# Abstracts from Hydrocephalus 2023—the fifteenth meeting of the Hydrocephalus Society

**DOI:** 10.1186/s12987-023-00498-1

**Published:** 2024-02-02

## A1 Idiopathic Normal Pressure Hydrocephalus in a Klinefelter Syndrome the first case in the literature

### Gianpaolo Petrella, Silvia Ciarlo, Graziano Taddei, Angelo Pompucci, Alessandro Pesce

#### ^1^A.O. “Santa Maria Goretti” General Hospital, Neurosurgery Division, Latina, Italy

##### **Correspondence**: Gianpaolo Petrella, gianpaolo_p@hotmail.com

*Fluids and Barriers of the CNS* 2023, **20(Suppl 2)**: A1

**Introduction**: Idiopathic Normal Pressure Hydrocephalus (iNPH) shows a typical clinical triad consisting of gait disturbance, dementia, and urinary incontinence, often combined with ventriculomegaly. Fortunately, these clinical manifestations are potentially reversible by adequate surgical treatment. Men affected by Klinefelter Syndrome (KS) harbor cognitive problems, those include impairments in both verbal and nonverbal memory, and in executive functions, which could be related to Idiopathic Normal Pressure Hydrocephalus. Although correlation between chromosomic disorders and iNPH has been described in Down Syndrome, to our knowledge direct association between iNPH and KS has never been discussed in the literature. We first described a case of iNPH in a patient with KS. The relevant literature and clinical implications are also discussed.

**Case Report**: A 62-year-old-man genetically diagnosed with KS had 1 year history of gait ataxia determining recurrent falls. He also developed progressive cognitive impairment leading to the impossibility to perform everyday activities. His magnetic resonance (MR) scan showed ventriculomegaly.

A positive lumbar infusion test was then obtained. With this evidence the patient underwent shunt surgery. At one month follow-up the patient had a significant improvement of the ataxia, and he was able to walk with help.

**Conclusion**: KS is the most frequent chromosomal aberration in men, and besides infertility, it can manifest also with cognitive and neurological disfunctions. When these symptoms appear, even if at a younger age than usual, diagnosis of iNPH in those patients should be suspected and investigated. Indeed, its treatment can lead to an opportunity to improve their life quality. Informed consent to publish has been obtained by the patient.

## A2 Is nausea during lumbar drainage a useful predictor of shunt responsiveness?

### Linda D’Antona^1,2^, Zeid Abussuud^1^, David Rowland^1^, Eleanor Moncour^1,2^, Kanza Tariq^1,2^, Lucia Darie^1^, Aleksandra Lasica^2^, Lewis Thorne^1^, Laurence Watkins^1,2^, Ahmed Toma^1,2^

#### ^1^Victor Horsley Department of Neurosurgery, National Hospital for Neurology and Neurosurgery, London, WC1N3BG, UK; ^2^Queen Square Institute of Neurology, University College London, London, WC1N3BG, UK

##### **Correspondence**: Linda D’Antona, linda.d’antona@nhs.net

*Fluids and Barriers of the CNS* 2023, **20(Suppl 2)**: A2

**Introduction**: Extended lumbar drainage (LD) is used to identify normal pressure hydrocephalus (NPH) patients who are likely to benefit from the insertion of a permanent cerebrospinal fluid (CSF) shunt system, also referred to as ‘shunt-responsive’ patients. One of the most common complaints of patients undergoing 3-days LD is nausea, it is possible that excessive drainage of CSF could be a leading cause. This study tested the hypothesis that nausea during LD may have a good negative predictive value (NPV) for shunt-responsiveness in NPH.

**Methods**: Retrospective single-centre observational study. Patients admitted for extended LD for NPH since January 2020 were identified. Patients who followed a standard CSF drainage protocol with complete information regarding walking test results, signs and symptoms during drainage, administration of medications (including possible sedatives during procedure) were included. Shunt-responsiveness was defined as a 10% improvement in walking test results after LD (either in number of steps or speed). Fisher’s exact test was used to test the association between shunt-responsiveness and nausea.

**Results**: Fifty-six patients (34M, 22F, mean age 75 ± 7 years) were included. Forty-five patients (80%) were ‘shunt-responsive’ and had average improvement in post-LD walking test of 25% in terms of speed and 21% in terms of number of steps. The remaining 11 patients (20%) did not have a significant improvement in the walking test. Overall, 15 patients (27%) complained of nausea during the lumbar drainage period, of these 11 were in the ‘shunt-responsive’ group and 4 in the ‘non-shunt-responsive’ group. The Fisher’s exact test did not demonstrate an association between nausea and shunt-responsiveness (p = 0.46). No statistical association was found between nausea and other factors tested (age, sex, sedatives, BMI, pain, comorbidity index).

**Conclusions**: Nausea is a common finding in NPH patients undergoing LD and, according to the results of this study, it is not a useful indicator of shunt-responsiveness.

## A3 Packaging designs for medical device drug combination products - impact on required storage conditions and shelf-life

### Lucas Thieme, Christoph Miethke

#### ^1^Christoph Miethke GmbH & Co. KG, 14469 Potsdam, Germany

##### **Correspondence**: Lucas Thieme, Lucas.Thieme@Miethke.com

*Fluids and Barriers of the CNS* 2023, **20(Suppl 2)**: A3

**Introduction**: Drug combination products pose the chance to overcome limitations of conventional medical devices. For example, active pharmaceutical ingredients (APIs) coated on stents, help to prevent stent restenosis. While medical devices mainly made of polymeric, metal or ceramic materials can be considered robust, many APIs are of limited stability because of sensitivity to heat, oxygen and moisture. The limited stability of the APIs limits the shelf life of the medical device itself and poses a challenge during transportation and storage. Sterile barrier systems for conventional medical devices mainly have to make the sterilization process possible and afterwards assure sterility. Additionally, a sterile barrier of a medical device drug combination product has to protect the APIs over shelf life.

Methods: Various packaging designs for medical devices are available today. Different sterile barrier systems and suitable sterilization methods have been evaluated and compared to each other, regarding their potential to improve the stability of APIs incorporated in medical devices.

**Results**: There is no perfect sterile barrier available that fulfils all requirements and should be recommended in general, today. Each packaging design is associated with individual limitations as well as individual advantages. Depending on the requirements as defined by the medical device and the incorporated APIs, it is possible to recommend a suitable sterile barrier system and sterilization method.

**Conclusions**: Adding an API to an established medical device is likely to change the requirements regarding a suitable sterile barrier. By selecting a suitable combination of sterile packaging and sterilization process, the requirements of sterilizability and product protection of a medical device drug combinations product can be met.

## A4 Theophylline a drug efficient to increase intracranial pressure: case report and review of literature

### Aoife Curran^1^, Laurence Watkins^2^, Lucia Darie^2^

#### ^1^Medical Student at University College Dublin, Ireland; ^2^Department of Neurosurgery, The National Hospital for Neurology and Neurosurgery, University College Hospitals London, United Kingdom

##### **Correspondence**: Lucia Darie, darielucia@yahoo.com

*Fluids and Barriers of the CNS* 2023, **20(Suppl 2)**: A4

**Introduction**: Limited information is available regarding the direct effect of drugs prescribed to elevate intracranial pressure in contrast to the abundance of evidence in support of medication utilized to lower intracranial pressure. Theophylline is a methylxanthine derivate used in the treatment of respiratory diseases with only a few randomized control trials or case reports describing pain improvement in low CSF pressure states or spontaneous intracranial hypotension (SIH). We present the case of a recorded increase in intracranial pressure under Theophylline.

**Case Report**: A 23-year-old female with a complex history of hydrocephalus and syringomyelia was experiencing debilitating symptoms due to refractory intracranial hypotension. Medical management with oral theophylline was attempted for a period of three months. Intracranial pressure measurements were obtained via the telemetric pressure sensor reservoir (Miethke®, M.scio®) incorporated in the patient’s ventriculoperitoneal shunt system.

**Results**: A significant increase in intracranial pressure was recorded at therapeutic drug levels.

**Conclusion**: This is the first report of an increase in intracranial pressure under oral theophylline medication. Informed consent to publish has been obtained by the patient.

**Keywords**: intracranial pressure, theophylline, hydrocephalus, low pressure CSF states

## A5 An investigation of the usefulness of the Rey-15 cognitive performance validity test, to detect suboptimal effort on patients with suspected idiopathic Normal Pressure Hydrocephalus (iNPH)

### Aishah Hannan^1^, Cathryn Harries^1^, Emma King^1^, Abbie Brockway^1^, Beth Wheeler^1^, Elizabeth Cray^2^, Samuel Jeffery^2^, Samiul Muquit^2^, Thomas Davis^1^, Rupert Noad^1^

#### ^1^Department of Neuropsychology, University Hospital Plymouth, Plymouth, United Kingdom; ^2^Department of Neurosurgery, University Hospital Plymouth, Plymouth, United Kingdom

##### **Correspondence**: Aishah Hannan, aishah.hannan@nhs.net

*Fluids and Barriers of the CNS* 2023, **20(Suppl 2)**: A5

**Introduction**: Cognitive decline is a feature of iNPH and is routinely assessed by neuropsychological testing. Although cognitive tests are sensitive to iNPH related cognitive change, a patient’s performance is also at risk of being influenced by non-organic variables such as anxiety and lack of motivation. It is therefore common practice to administer performance validity tests (PVTs) to determine data reliability and insight into a patient’s cognitive difficulties. The present study evaluates the use of the Rey-15 Item Test (Rey, 1964) in patients suspected of iNPH.

**Methods**: A retrospective record review of 38 brief, diagnostic cognitive assessments administered between November 2021 to February 2023 was conducted. Raw scores from the Rey-15 Item Test were obtained and categorised into two groups: valid effort or suspect effort. A cut off score of < 21 was used to indicate effort validity. Bayesian Chi-squared tests were used to analyse the relationship between effort outcome and clinical intervention.

**Results**: Thirty-eight patients (Female *n* = 16; Male *n* = 22; age *M* = 75 years), referred for suspected iNPH, underwent a brief cognitive assessment during a diagnostic iNPH clinic. After administration, 71% of patients showed suspect effort, 29% showed valid effort. Of the 38 patients, 66% of patients (*n* = 25) were clinically deemed to have iNPH and warranted further investigation independent of their PVT outcome. Chi square values did not show a significant relationship between effort outcome and clinical recommendation, BF = 0.88, χ^2^ (1)  = 0.91, p = 0.34.

**Conclusions**: Differences in PVT performance in patients suspected of iNPH is not likely due to effort alone. The recommended cut-off score of < 21 may be sensitive to disease-specific factors such as cognitive dysfunction and apathy. Future research might examine these findings by incorporating PVTs with optimal cut-off scores for disease-specific groups, such as the Dot Counting Test (Boone, 2002).

## A6 Hydroflex: The Use of Patient Reported Outcomes in a Clinical Setting Among Patients with Hydrocephalus

### Arnar Astradsson^1^, Nana Txvig Sorensen^2^, Anne Sofie Graversen^1^, Ivona Nemeiko^1^, Arzu Bilgin-Freiert^1^, Liz Marit Valen^3^, Marianne Juhler^1^, Toben Skovbo Hansen^1^

#### ^1^Aarhus University Hospital; ^2^AmbuFlex - Center for Patient-reported Outcomes, Central Denmark Region, Gødstrup Hospital, Herning, Denmark; ^3^Center for Patient-reported Outcomes, Central Denmark Region, Gødstrup Hospital, Herning, Denmark

##### **Correspondence**: Arnar Astradsson, arnar.astradsson@gmail.com

*Fluids and Barriers of the CNS* 2023, **20(Suppl 2)**: A6

**Introduction**: A patient-reported outcome (PRO) measure is defined as “any report of the status of a patient’s health condition coming directly from the patient without interpretation of the patient’s response by a clinician or anyone else”. PRO data are increasingly being used in healthcare to monitor symptoms and identify problems early, and reduce outpatient clinic appointments.

**Objectives**: Hydrocephalus is a common neurosurgical condition. We have designed a PRO system specifically for hydrocephalus patients, a program named Hydroflex. The primary objective of Hydroflex is to utilize the PRO responses to evaluate whether the patient needs a contact. The secondary objective is the use of the patients’ responses to support patient involvement, communication, and education.

**Methods**: Patients receive questionnaires online at home. Based on an automated algorithm, the patients’ PRO measures are ranked to guide clinical decision making. PRO responses are automatically assigned a given color code of “green”, “yellow”, or “red”. “Red” answers indicate that the patient needs contact, "yellow” indicates that the patient may need contact and “green” indicates that there is no need for attention currently.

**Results**: A total of 355 patients, 172 men and 183 women, have been enrolled in Hydroflex since it was launched in October 2017. In total, 1385 responses have been recorded, with 139 (10%) all green responses, 651 (47%) with one or more yellow responses and 594 (43%) with one or more red responses. Decisions in all instances could be made based on the questionnaires; 683 (50%) patients needing to be contacted and 690 (50%) not needing to be contacted.

**Conclusions**: We believe, that Hydroflex provides more continuity in the treatment of patients with hydrocephalus. It provides for a more standardized follow-up scheme, and we postulate that this will in turn lead to improved patient satisfaction and involvement and lead to fewer outpatient appointments.

## A7 Spinal tumours as cause of normal pressure hydrocephalus: a case series

### Zeid A. Abussuud, Lucia Darie, Ahmed Toma, Laurence Watkins

#### ^1^Department of Neurosurgery, National Hospital for Neurology and Neurosurgery, University College London Hospitals, United Kingdom

##### **Correspondence**: Zeid A Abussuud, z.abussuud@nhs.net

*Fluids and Barriers of the CNS* 2023, **20(Suppl 2)**: A7

**Introduction**: Normal pressure hydrocephalus (NPH) is a cerebrospinal fluid (CSF) disorder characterised by ventriculomegaly, cognitive impairment, urinary incontinence, and gait apraxia. The role spinal pathology and CSF circulation in this disease remains elusive. Whilst NPH is known to coexist with other comorbidities there are only a few case reports up to date describing spinal tumors causing NPH like symptoms independently. We aim to report a small case series.

**Methods**: This is a retrospective case series study of patients presenting with NPH-like symptoms attributed to spinal pathology. Demographic, clinical, and radiological data were collected from each patient.

**Results**: Four (3 males and 1 female) patients, aged 50–80 years, with spinal tumors presenting as NPH were identified. Amongst these cases two underwent tumor resection only, one spinal and CSF diversion surgery and the last patient was recommended conservative treatment due to comorbidities. In both cases requiring only spinal surgery the histological diagnosis was schwannoma, with one of the patients showing significant objective improvement in neurocognitive testing and reduction in the ventricular size on subsequent imaging.

**Conclusion**: Spinal tumours are a rare cause of NPH and are often overlooked as a potential differential diagnosis. This case series serves as a reminder that rare causes of NPH should be considered. Nevertheless, it equally highlights a need for better understanding of the role of spinal CSF dynamics in the pathophysiology of this disease.

## A8 Distal Catheter Migration In Ventriculoperitoneal Shunt: Gastric Perforation and Scrotal Migration – Report of two uncommon cases

### Cristian Leonardo Ortiz Alonso, Juan Rial Basalo, Patricia Barrio Fernandez, Jenny Leon Rivera, Noelia Miron Jimenez, Cristina Ferreras Garcia, Belen Alvarez Fernandez

#### ^1^Hospital Universitario Central de Asturias, Oviedo, Spain

##### **Correspondence**: Cristian Leonardo Ortiz Alonso, cristian.or9202@gmail.com

*Fluids and Barriers of the CNS* 2023, **20(Suppl 2)**: A8

**Introduction**: The ventriculoperitoneal shunt (VPS) is the most commonly used and preferred procedure in the management of hydrocephalus. Various methods for the introduction and fixation of the distal catheter exist, and possible complications include malposition, obstruction, and less frequently, migration, which can be internal, external, or combined. In this report, we present two cases of internal migration.

**Method**: In the first case (C1), a 70-year-old patient with a history of Von Hippel Lindau disease underwent multiple surgeries for cerebellar and suprasellar hemangioblastomas. Twelve years after the placement of a VPS, the patient presented to the emergency department with mammary cellulitis adjacent to the distal catheter of the VPS, which did not improve with antibiotics. Attempted removal and relocation of the distal catheter to the left side proved challenging due to adhesions.

In the second case (C2), a 2-year-old patient with a history of bilateral communicating hydrocele and congenital hydrocephalus associated with a left retrocerebellar cyst underwent uneventful VPS placement. After 10 months, the patient presented to the emergency department with an exacerbation of the hydroceles, and a radiographic examination confirmed the presence of the distal catheter in the right scrotum.

**Results**: In C1, a thoracoabdominal tomography was performed, revealing intragastric positioning of the catheter. Endoscopic removal was necessary, and the patient received targeted antibiotic treatment based on commensal microbiota and P. mirabilis cultures, resulting in improvement. In C2, surgical intervention was performed for laparoscopic relocation of the distal catheter along with bilateral hydrocelectomy.

**Conclusions**: It is important to highlight that internal migration of the distal catheter in VPS is a rare but potentially serious complication due to the risk of intracranial infection, including meningitis, encephalitis, and/or ventriculitis. These two cases illustrate its clinical presentation and the challenges associated with its management. Informed consent to publish has been obtained.

## A9 Gait improvement following cerebrospinal fluid tap test in normal pressure hydrocephalus patients with striatal dopaminergic deficit: a preliminary study

### Minju Kim^1^, Young Ho Park^1^, Yoo Sung Song^2^, SangYun Kim^1^

#### ^1^Department of Neurology, Seoul National University College of Medicine and Department of Neurology, Seoul National University Bundang Hospital, Seongnam, Korea; ^2^Department of Nuclear Medicine, Seoul National University College of Medicine and Department of Nuclear Medicine, Seoul National University Bundang Hospital, Seongnam, Korea

##### **Correspondence**: Young Ho Park, kumimesy@snubh.org

*Fluids and Barriers of the CNS* 2023, **20(Suppl 2)**: A9

**Introduction**: Although dopamine transporter imaging is considered to show no striatal dopaminergic deficit in patients with normal pressure hydrocephalus (NPH), there have been reported cases of NPH patients exhibiting striatal dopaminergic deficit on dopamine transporter imaging. However, the extent of gait improvement following a cerebrospinal fluid (CSF) tap test in NPH patients with striatal dopaminergic deficit has not been comprehensively investigated.

**Methods**: We assessed the walking speed of eight consecutive NPH patients, who had striatal dopaminergic deficit as determined by ^18^F-N-(3-fluoropropyl)-2β-carboxymethoxy-3β-(4-iodophenyl) nortropane (^18^F-FP-CIT) positron emission tomography (PET), before and after the CSF tap test using 3D gait analysis. A response to the CSF tap test was defined as an improvement of 10% or more in walking speed.

**Results**: In the study population, a total of five patients (62.5%) demonstrated a positive response in walking speed, after undergoing the CSF tap test.

**Conclusions**: The findings from our preliminary study suggest that gait improvement can be achieved in more than half of NPH patients with striatal dopaminergic deficit as determined by ^18^F-FP-CIT PET. Further studies with larger sample sizes are needed to validate these results.

## A10 Does the intensive care monitor show the correct intracranial pressure? Comparison of monitor output values with raw data from high-frequency recordings

### C. B. Bluemel^1^, C. Raak^2^, L. Wistorf^2^, B. Petzold^1^, D. Cysarz^2^, W. Scharbrodt^1,2^

#### ^1^Department of Neurosurgery, GKH Herdecke, 58313 Herdecke, Germany; ^2^University of Witten/Herdecke, 58455 Witten, Germany

##### **Correspondence**: Carl Benjamin Blümel, c.bluemel@gemeinschaftskrankenhaus.de

*Fluids and Barriers of the CNS* 2023, **20(Suppl 2)**: A10

**Introduction**: Monitoring of intracranial pressure is a standard procedure in the intensive care treatment of patients with certain morbidities. The processing of the underlying raw data is not standardized. Post-processing of the data in monitor systems by averaging and/or filtering may lead to significant deviations. The purpose of this study is to compare monitor output and raw data and to examine the suitability of conventional values to serve as a basis for clinical decisions.

**Methods**: 39 patients from our ICU with intracranial hypertension (ICH) who had a complete 60-minute recording from 2 a.m. on the first day after probe implantation were included. ICP, BP, ECG, pulse oximetry, and various ventilation parameters were collected as raw data. Data were recorded at 100 Hz and automatically stored on a scientific server system. Similarly, the ICP output from the Draeger Infinity Delta XL system was recorded.

The starting point of each ICP signal for all 39 data sets was identified and synchronized. Then, ICP was determined for each heartbeat from each wave area integral. The data collected was then compared with the monitor output data.

**Results**: In 36 patients, significant differences are shown. The median differences are smaller than − 2 mmHg in 8 of 39 patients (20.5%), which means that the ICP carrier monitor data are greater than the ICP area calculations by at least 2 mmHg in these patients. If we consider a limit of − 1 mmHg, then 13 of 39 patients (33.3%) are found in whom the monitor output data are at least 1 mmHg larger than the ICP area calculation.

**Conclusions**: The determination of the intracranial pressure using the area integral seems reasonable. The deviations shown in this study are probably due to the morphology of the pressure waves and the way they are processed by conventional monitoring systems.

## A11 Slit Ventricle Without Subdural Hematoma After Ventriculo-atrial Shunt (VAS) For idiopathic normal pressure hydrocephalus (INPH)

### Kiyoshi Takagi^1,2,3^, Shuichiro Asano^4^, Ryosuke Takagi^5^, Takashi Kawahara^3^, Masamichi Atsuchi^3^

#### ^1^NPH Center, Abiko Seijinkai Hospital, Abiko, Japan; ^2^Department of Neurosurgery, Fujita Health University, Nagoya, Japan; ^3^NPH Center, Jifukai Atsuchi Neurosurgical Hospital, Kagoshima, Japan; ^4^Department of Neurosurgery, Kashiwatanaka Hospital, Kashiwa, Japan; ^5^Department of Neurosurgery, Yokohama City University, Yokohama, Japan

##### **Correspondence**: Kiyoshi Takagi, paulktkg@mac.com

*Fluids and Barriers of the CNS* 2023, **20(Suppl 2)**: A11

**Introduction**: INPH can be treated only by CSF shunting. One of the most common complications of CSF shunt is overdrainage. The overdrainage results in slit ventricles (SV) in the pediatric CSF shunting, whereas it usually causes subdural hematoma (SDH) in shunting for iNPH. We retrospectively investigated the incidence and symptoms of SV without SDH after ventriculo-atrial shunt (VAS) for iNPH.

**Methods**: We performed 1,023 VASs for iNPH from April, 2004 to March, 2021. Post-operative neuroimaging studies and symptoms were retrospectively investigated to find SV without SDH. Data were shown in mean (SD).

**Results**: Mean age was 78.3 (6.8) years old (male: 78.1 (6.9), n = 545, female: 78.5 (6.7), n = 478). Only four cases with SV without SDH were found. Case 1: Sixty-nine years old woman developed SV 1 year after the shunt (Codman Hakim Programmable Valve with siphon guard (CHPV-SG)) with excellent outcome. Case 2: Seventy-three years old woman developed SV one week after the shunt (CHPV-SG) without any deterioration. Case 3: Seventy-six years old woman developed SV six months after the shunt (Polaris SPVA-140) with excellent outcome. Case 4: Fifty-eight years old woman received ventriculo-peritoneal shunt (VPS) (Certas-plus) developed SV three months after the VPS with severe headache, involuntary movement, and consciousness disturbance. She received VAS (CHPV-SG) with abdominal catheter ligation 1 year after the VPS. Although she developed SV six months after the VAS she showed no neurological deterioration.

**Conclusions**: Despite male dominancy in our VAS series, all four cases were women. Slit ventricle is a serious complication in the pediatric shunting and in the VPS for iNPH as suggested by case 4. However, no serious deterioration was observed in SV after VAS for iNPH. This observation may suggest that VAS is safer than VPS for iNPH at least regarding slit ventricles.

## A12 Early emergence of isolated fourth ventricle following meningitis in adult: a case report

### Han-Lin Yen^1^, I-i Chen^2^

#### ^1^Department of Neurosurgery, Tainan Municipal Hospital (Managed by Show Chwan Medical Care Corporation), Tainan, Taiwan; ^2^Department of hepatogastroenterology, Tainan Municipal Hospital (Managed by Show Chwan Medical Care Corporation), Tainan, Taiwan

##### **Correspondence**: Dr. Han-Lin Yen, hanlin.yen@me.com

*Fluids and Barriers of the CNS* 2023, **20(Suppl 2)**: A12

**Introduction**: An isolated fourth ventricle (IFV) is an uncommon phenomenon primarily described in children. Usually, it occurs with ventricular shunts placed for communicating hydrocephalus following hemorrhage or meningitis. We report an early emergence of IFV in an adult following hypervirulent Klebsiella pneumoniae (hvKP) meningitis, the approach to management, and the neurological outcome.

**Methods**: We report the case of a 54-year-old woman with hvKP infection caused liver abscess, urinary tract infection, pneumonia, meningitis, and brain abscess. The central neurological system (CNS) infection management includes antibiotics for meningitis, external ventricle drainage for post-meningitis hydrocephalus, and suboccipital craniectomy for removed cerebellar abscesses. She developed early entrapment of the fourth ventricle and compartmentalized hydrocephalus. After controlling the CNS infection, we treated the patient with multiple shunt catheters, including fourth ventriculoperitoneal shunting.

**Results**: She could go back home, but her daily activity was dependent. She was drowsy; Glasgow Coma Scale was 12/15 (E3, M6, V3), and was bedridden most of the time at six months after her discharge.

**Conclusions**: This rare case report serves as a reminder that IFV can emerge early in adults following hvKP CNS infection. Early identification of IFV following CNS infection requires high clinical vigilance. More frequent brain computed tomography can provide valuable information about the posterior fossa and ventricular system in high-risk patients. In general, surgical treatment of an isolated fourth ventricle requires the placement of a shunt catheter into the fourth ventricle. Informed consent to publish has been obtained by the patient.

## A13 Ability for Basic Movement Scale 2 (ABMS-2) Evaluation in iNPH Diagnosis

### Masamichi Atsuchi

#### Atsuchi Neurosurgical Hospital, Kagoshima, 892-0842, Japan

##### **Correspondence**: Masamichi Atsuchi, atsuchi0824@yahoo.co.jp

*Fluids and Barriers of the CNS* 2023, **20(Suppl 2)**: A13

**Introduction**: Indicators that demonstrate high improbability in the treatment of idiopathic normal pressure hydrocephalus (iNPH) are required. There are cases where disproportionately enlarged subarachnoid-space hydrocephalus (DESH) is observed but does not improve even with lumboperitoneal (LP) shunt, so we consider ABMS-2, which quantifies basic motor ability, as a simple and useful indicator in addition to DESH findings. Based on our experience using ABMS-2 as an evaluation before and after the tap test, we discuss the usefulness of ABMS-2 in iNPH diagnosis.

**Methods**: We conducted ABMS-2 evaluations on all 130 cases with DESH who underwent tap test between January 1 and December 31, 2018. We reviewed the outcomes of LP shunt in 92 cases with ABMS-2 scores of 30 points and in 38 cases with scores below 30 points and discussed the usefulness of ABMS-2.

**Results**: Of the 92 cases who underwent LP shunt with ABMS-2 score of 30 points, 45/47 (95.7%) were diagnosed with definite iNPH. Of the 38 cases who underwent LP shunt with ABMS-2 score of less than 30 points, 21/24 (87.5%) were diagnosed with definite iNPH.

**Conclusions**: iNPH is a disease of "treatable gait disturbance" and shunt surgery is a treatment aimed at "walking in daily life." ABMS-2 is a measure of the remaining motor ability and effort put into rehabilitation, and we consider it a useful indicator for analyzing movement in iNPH diagnosis, in addition to DESH, an image indicator that demonstrates high improbability.

## A14 The impact of a standardized surgical protocol on shunt revision in people with Idiopathic Intracranial Hypertension and a pre-existing shunt

### Yousra Rasool^1^, Joseph Welch^2^, Sheikh M. B. Momin^1^, Marian Byrne^1^, Alexandra J. Sinclair^3,4^, Susan P. Mollan^4,5^, Georgios Tsermoulas^1,4^

#### ^1^Department of Neurosurgery, Queen Elizabeth Hospital Birmingham, United Kingdom; ^2^Department of Emergency Medicine, City Hospital, Birmingham, United Kingdom; ^3^Department of Neurology, Queen Elizabeth Hospital Birmingham, United Kingdom; ^4^Institute of Metabolism and Systems Research, University of Birmingham, United Kingdom; ^5^Birmingham Neuro-Ophthalmology, Queen Elizabeth Hospital Birmingham, United Kingdom

##### **Correspondence**: Georgios Tsermoulas, georgios.tsermoulas@nhs.net

*Fluids and Barriers of the CNS* 2023, **20(Suppl 2)**: A14

**Introduction**: Insertion of a ventriculoperitoneal (VP) shunt in people with idiopathic intracranial hypertension (IIH) is associated with high revision rates. A standardised surgical protocol at our centre significantly reduced revision rates for primary shunts. In this study, we examined the impact of the protocol in people with IIH who had a pre-existing shunt and underwent revision as per the protocol.

**Methods**: We analysed data from consecutive patients who underwent VP shunt insertion for IIH since the implementation of the protocol in July 2019. The cohort was divided into two groups: those who underwent shunt insertion for the first time (primary group) and those with a pre-existing shunt that underwent revision (secondary group). We compared shunt failure between the groups, in order to assess the effectiveness of the protocol in revision surgery.

**Results**: Eighty-eight patients underwent VP shunt insertion during 45 months. Seventy-four had primary shunt and 14 had a pre-existing shunt and underwent shunt surgery based on the protocol. The thirty-day revision rate was 5.4% in the primary vs. 7.1% in the secondary group (p = 0.6). For patients that had completed 1 year follow up, 5/62 in the primary and 4/11 in the secondary group underwent revision (8.1% vs 36.4%, p = 0.02). Overall, 8 patients from the primary (10.8%) and 4 from the secondary group (29%) required revision (p = 0.09) during the study period. In the primary group half of the revisions were due to proximal underdrainage and in the secondary group the majority was due to distal catheter complications.

**Conclusions**: Revision surgery is more challenging and our standardized IIH shunt protocol demonstrated favourable revision rates in IIH patients with a pre-existing shunt requiring revision. Our study supports the use of a standardized surgical protocol when inserting shunts for IIH.

## A15 Risk factors for slit ventricles in hydrocephalic children treated with ventriculo-peritoneal shunt

### Sadahiro Nomura^1^, Natsumi Fujii^1^, Hideyuki Ishihara^1^

#### ^1^Department of Neurosurgery, Yamaguchi University School of Medicine, Ube, Yamaguchi, 7558505, Japan

##### **Correspondence**: Sadahiro Nomura, snomura@yamaguchi-u.ac.jp

*Fluids and Barriers of the CNS* 2023, **20(Suppl 2)**: A15

**Introduction**: The study analyzes the reasons for slit ventricle (SV)-related ventriculo-peritoneal (VP) shunt failure in pediatric patients with hydrocephalus.

**Methods**: The study included patients treated at Yamaguchi University Hospital from 1991 – 2022. The initial VP shunts were placed in patients aged 6 days to 14 years (mean age: 1.6 ± 3.0 years). Follow-up periods ranged from 6 months to 27 years. The VP shunts were revised due to SV syndrome or SV-related catheter obstruction in 19 patients (SV group). The shunts functioned until the final follow-up day in 39 patients (Control group). The patients with shunt malfunction not related to SV (n = 40) were excluded. Age at surgery, type of hydrocephalus (communicating or non-communicating), absence of brain atrophy, pressure setting of the shunt valve, and implantation of an anti-siphon device were compared between the groups.

**Results**: The age at surgery in the SV group was 0.7 ± 1.1 years which was significantly younger than that in the Control group at 2.6 ± 4.3 years (p < 0.05). The absence of brain atrophy was 94.7% in SV and 51.3% in the Control groups (p < 0.01). None of the non-communicating hydrocephalus, low-pressure valve, or absence of anti-siphon device significantly affected the shunt malfunction. Among the 19 patients in SV group, 3 patients became free from shunt due to compensated hydrocephalus. The other 16 patients received shunt revision with anti-siphon devices, and no shunt malfunction recurred for 12.8 ± 7.6 years.

**Conclusions**: Shunts implanted in infants with no atrophic brain were the risk factors for SV related shunt malfunction. The type of hydrocephalus and shunt system did not significantly influence shunt malfunction. Anti-siphon device did not prevent SV; however, it did prevent recurrence of SV-related shunt malfunction.

## A16 Syndromic validity of idiopathic normal pressure hydrocephalus (iNPH)

### Eric A. Schmidt^1^, Lubin Klotz^1^, Fabienne Ory Magne^2^, Margherita Fabbri^2^

#### ^1^Department of Neurosurgery, University Hospital, Toulouse, France; ^2^Department of Neurology, University Hospital, Toulouse, France

##### **Correspondence**: Eric A Schmidt: schmidt.e@chu-toulouse.fr

*Fluids and Barriers of the CNS* 2023, **20(Suppl 2)**: A16

**Introduction**: Hydrocephalus is a disease due to active distension of brain’s ventricles resulting from altered CSF circulation. Idiopathic normal pressure hydrocephalus (iNPH) is characterized by a set of core clinical and imaging features (gait, cognitive, urinary dysfunctions and enlarged ventricles). But the evaluation of iNPH is anchored in the description of clinical and imaging phenomena (*i.e.* phenomenology) that frame the diagnostic process, rather than the characterization of actual root-causes (*i.e.* aetiology) that ascertain disease identification. iNPH as a disease is often misleading due to the lack of standardized assessments for positive and differential diagnoses. iNPH should be regarded as a syndrome, a set of symptoms suggesting the presence of a certain disease, that also takes into account heterogeneity and temporal complexity of neurodegenerative diseases. We would like to provide arguments about the syndromic validity of iNPH.

**Methods**: We reviewed published literature about comorbidities of iNPH in the landscape of neurodegenerative disease and movement disorders.

**Results**: We identified consistent literature-based evidence of:

1. High prevalence of cardiovascular disease in iNPH patients, suggesting an overlap between iNPH syndrome and vascular dementia.
2. High prevalence of Alzheimer disease in iNPH patients, suggesting an overlap between iNPH syndrome and Alzheimer disease.
3. High prevalence of bradykinesia, akinetic symptoms and sometimes dopaminergic dysfunction in iNPH patients, suggesting an overlap between iNPH syndrome and Parkinson syndrome.
4. The presence of other diseases does not significantly influence the short-term clinical response to shunt.
5. But the presence of other diseases may significantly influence the long-term clinical response to shunt.

This figure emphasizes the required combinatorial framework for identifying commonalities and diversification within the subtypes of neurodegenerative diseases.
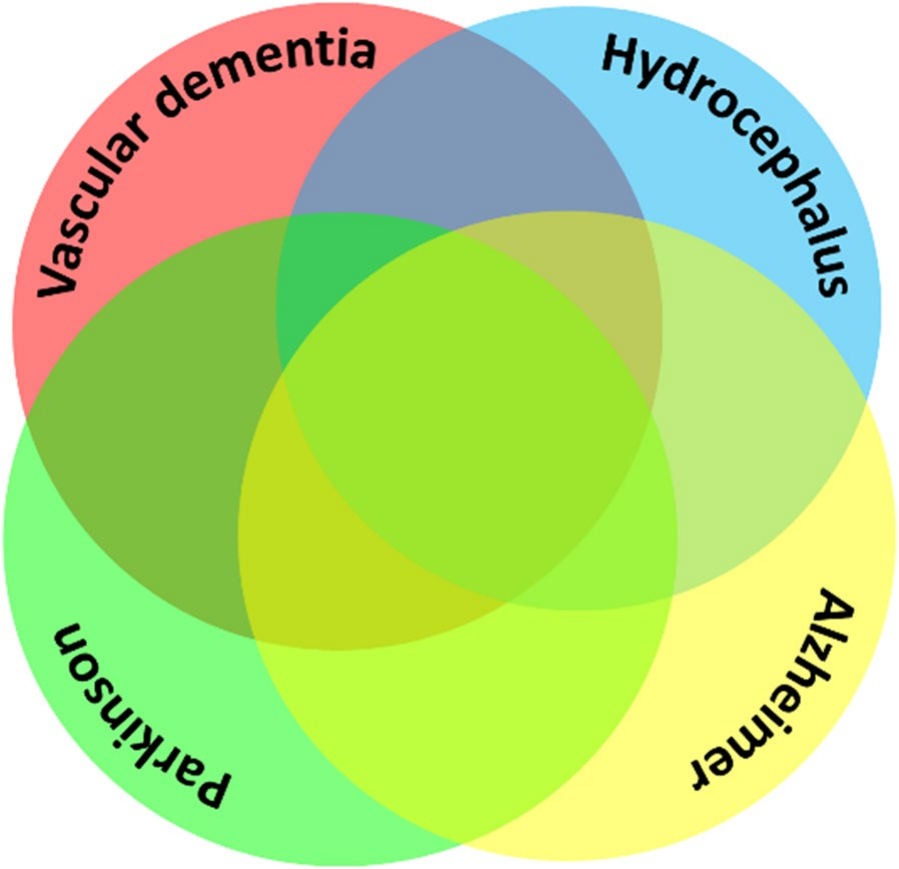


**Conclusions**: Despite decades of research, the etiopathogenesis underlying iNPH as a disease is still poorly understood. We provide a rationale for partial validation of the syndromic validity of iNPH.

## A17 Key Concepts in Minimally Invasive Neurosurgery (MIN): Application in Complex Hydrocephalus

### Klaus D. M. Resch^1^, Susanna M. Antal^2^

#### ^1^MIN/Univ. Guadalajara, Mexico; ^2^LKH Feldkirch Ophthalmology, Feldkirch, Austria

##### **Correspondence**: Klaus Resch, klausdmresch@ens-surgery.com

*Fluids and Barriers of the CNS* 2023, **20(Suppl 2)**: A17

**Introduction**: In the „Key-Concept“, we ask for the keys in MIN rather than for the key-holes. Complex hydrocephaly are excellent indications for MIN. The pathology is not incurable, but surgical procedure is very difficult, needing the spectrum of key-techniques of MIN. The extreme case is the preterm infant with very small space conditions and in addition immature tissue already traumatized by birth and bleedings.

**Methods**: This MIN concept combined 5 MIN-key techniques to assist microneurosurgery: high-end neuro-sonography with small probes („burr-hole-probe 8x8mm, ALOKA/ Hitachi) and mouth-tracking of the microscope, both mandatory. Additionally, we added endoscopy (Wolf, Aesculap, Storz) and LASER (Th-YAG Revolix). Sealing technique (Tachosil/ Takeda) is always used. A combination of techniques was used in each case.

**Results**: This observational series presents 6 cases which each needed a different combination of MIN-Key-Techniques. Most of the major conditions for complex hydrocephalus were represented by one case. In all cases a shunt could be avoided in the preterm infants at least during the first 6 months. In all cases the combination of techniques enabled very precise individually adapted MIN approaches. The two preterm infants with fatal prognosis also went home finally.

**Conclusions**: Application of MIN-Key-Concepts enabled good results in difficult complex hydrocephalus cases**. **Combination of Key-Techniques individually tailored for each case allow application in many cases of complex hydrocephaly**. **This MIN concept and techniques increased therapeutic options and need to be further examined by research and future studies.

Ref.: KDM Resch; Key Concepts in MIN, Vol. 1+2, 2020,2022/ Springer

## A18 Normal Pressure Hydrocephalus and Cerebral Atrophy: A Scoping review

### Afroditi D. Lalou*^1,2,3^, Thanasis Paschalis*^1,^ Zofia H. Czosnyka^1^, Marek Czosnyka^1^

#### ^1^Department of Clinical Neurosciences, Division of Neurosurgery, University of Cambridge & Cambridge University Hospital NHS Foundation Trust, United Kingdom; ^2^Department of Radiation Sciences, Umea University, Sweden; ^3^Department of Neurology, King’s College Hospital NHS Foundation Trust, London, United Kingdom

##### **Correspondence**: Afroditi Lalou, adl43@cam.ac.uk

*the authors contributed equally

*Fluids and Barriers of the CNS* 2023, **20(Suppl 2)**: A18

**Introduction**: This scoping review aims to investigate the diagnostic tools and evidence available in differentiating Normal Pressure Hydrocephalus (NPH) from cerebral atrophy. The variable aetiology of NPH, as well as the higher incidence of comorbidities in the ageing population has posed long-term challenges in diagnosing and managing those patients. A scoping review on studies that mention NPH, atrophy and relevant clinic-radiological comorbidities will provide an overview and structure for future research priorities.

**Methods**: We conducted a scoping review following the PRISMA January-June 2022. We searched Pubmed, Cochrane, Web of Science, Embase and Scopus. Search terms included normal pressure hydrocephalus and cerebral atrophy, biomarkers and magnetic resonance imaging. We included studies of adult humans investigated for idiopathic or secondary NPH in all languages.

**Results**: We assessed 13391 articles and included 6. Cerebral Blood Flow has been reviewed separately. CSF biomarkers, fluid dynamics, structural and diffusion imaging have been used to characterise NPH, atrophy and other dementias. Reporting of criteria, clinical presentation and factors influencing diagnosis and shunt treatment showed high variability in reporting and may pose significant challenges in future evidence synthesis.

**Conclusions**: Emphasis on a combination of structural and mechanistic studies in assessing NPH with current clinical and scientific tools is essential. Separating surgical treatment and outcomes from pathophysiological and diagnostic investigations as an initial approach may help expand availability of patient cohorts and decrease heterogeneity in reporting.

## A19 Minimal Invasive Neurosurgery (MIN) and Ophthalmology: Observation Series of Complex Hydrocephalus Cases

### Susanna Antal^1^, LKHF, Klaus D. M. Resch^2^

#### ^1^Ophthalmology, Feldkirch; Austria; ^2^MIN, Univ. Guadalajara; Mexico

##### **Correspondence**: Susanna Antal, zsuzsinak@yahoo.de

*Fluids and Barriers of the CNS* 2023, **20(Suppl 2)**: A19

**Introduction**: Preservation of visual function can be optimized in selective cases only by close cooperation of ophthalmology and MIN. The cases are an in-homogenous and complex series. The analysis of the ophthalmological outcomes to prove the effect of functional recovery by minimal invasive neurosurgical procedures (MIN) included 11 cases of tumor-/ liquor dynamics-diseases, in all cases causing disturbance of visual function.

**Methods**: This concept combined 5 MIN-key techniques to assist microneurosurgery: high-end neuro-sonography with small probes, mouth-tracking of the microscope -both mandatory-, endoscopy and LASER. Sealing technique was always used. Ophthalmological standard techniques were perioperatively used to meticulously document ophthalmological functions. Visual acuity, 30°-visual field, RNFL and fundoscopy were examined as the patients´ condition did allow so.

**Results**: In the tumor-/ liquor dynamics-disease group were 4 males and 7 females. Reasons of the liquor- pathway-obstruction were: 7 tumors, 2 cyst, 6 obstructive hydrocephaly, 2 post-ICH cases and 4 very complex cases. Seven of them were emergency cases regarding “last minute cases” in preservation of visual function. In both groups, fast visual function recovery could be documented. MIN techniques and ophthalmological examinations differed in relation to the patients´ individual conditions.

**Conclusions**: Close cooperation of neurosurgery and ophthalmology can preserve visual functions even in emergency cases. Ophthalmological examination-techniques may support the indication making as well as outcomes analysis as an excellent model to show functional recovery after MIN procedures. Ophthalmology plays in this context the rule of an emergency indicator. A close to the patients and an individual management came out to be necessary.

## A20 Technical analysis of Hydrocephalus Shunt Revisions: How MIETHKE investigates? What do we learn? The importance of data for patients, clinics & industry!

### August V. Hardenberg, Jörg Knebel, Christoph Miethke

#### Christoph Miethke GmbH & Co. KG, Potsdam, 14469, Germany

##### **Correspondence**: August v. Hardenberg, august.hardenberg@miethke.com

*Fluids and Barriers of the CNS* 2023, **20(Suppl 2)**: A20

**Introduction**: The importance of data is globally obvious. The aim of this work is to highlight the learning outcome and importance of data collection regarding technical analysis of revised shunts for the Hydrocephalus society (patients, clinics and industry).

**Methods**: The methods of technical analysis and data collection of examination results of returned products have been redesigned over the last years. In addition, the data collected has been analyzed.

**Results**: Derived from feedback of neurosurgeons and technical analysis of revised shunts MIETHKE started research projects and developed the valve generation 2.0. Technical analysis showed a significant improvement for the MIETHKE valve generation 2.0. But still highlights side effects of the treatment of hydrocephalus by shunting. Thus analyses are driving forces for innovation but also point out a further need for innovation. Results show a correlation regarding patient age and deposits in revised valves. Furthermore 48% of suspected reasons for the malfunction were not confirmed and 32% of the products investigated worked within all product specifications.

**Conclusions**: Technical analysis of revised shunts opens another part of the black box of shunt revisions. This work highlights the importance of looking at all possible aspects to provide further insights for the hydrocephalus community, particularly patients, neurosurgeons and engineers. A more complete data set of shunt revisions including patient’s quality of life on a European (better global) basis is needed. Insights from shunt revision can raise learning curves for neurosurgeons and engineers. In addition, further innovations can be driven by newly gained knowledge.

## A21 Idiopathic Normal Pressure Hydrocephalus (iNPH) Is Common among Nonagenarians in the Takahata Cohort

### Chifumi Iseki^1,2^, Yoshimi Takahashi^3^, Michito Adachi^4^, Shigenori Kanno^1^, Ryosuke Igari^2^, Hiroyasu Sato^2^, Kyoko Suzuki^1^, Takeo Kato^5^, Yasuyuki Ohta^2^

#### ^1^Department of Behavioral Neurology and Cognitive Neuroscience, Tohoku University Graduate School of Medicine, Sendai-city, Miyagi, 980-8575, Japan;^2^Division of Neurology and Clinical Neuroscience, Department of Internal Medicine III, Yamagata University School of Medicine, Yamagata-city, Yamagata, 990-3221, Japan; ^3^Haneda clinic, Yamagata, Japan; ^4^Oshima clinic, Yamagata, Japan; ^5^Yamagata University, Yamagata, Japan

##### **Correspondence**: Chifumi Iseki email: chi-iseki@hyper.ocn.ne.jp

*Fluids and Barriers of the CNS* 2023, **20(Suppl 2)**: A21

**Introduction**: We aimed to discover the epidemiology of iNPH in nonagenarians.

**Methods**: The cohort study, which analyzed gait, cognitive functions, and brain MRI or CT for all participants, started in 2000 when 272 at 70 in Takahata town participated. In 2022/2023, at age 92, of 102 living individuals, 78 participated in at least one interview alone or with their families to evaluate their clinical dementia rating (CDR) and modified Rankin scale (mRS). iNPH is diagnosed as having “possible iNPH with brain MRI support (defined by Japanese guidelines),” dementia of CDR > 1, and gait disturbance. The asymptomatic individual with findings of DESH (disproportionately enlarged subarachnoid space hydrocephalus) was called AVIM—asymptomatic ventriculomegaly with features of iNPH on MRI. DESH was defined as lateral ventricular enlargement (VE) plus tight high convexity (THC). When THC was not complete, we called it non-DESH iNPH. Imaging was retrospectively checked.

**Results**: Sixteen individuals (16/78, 20.5%) were considered as iNPH positive—nine of DESH iNPH, five of non-DESH iNPH, and two suspected of the transitional periods from AVIM. Of the cohort, twenty individuals lived in nursing homes; ten (10/20) were iNPH, and all except one presented the CDR of 3 and mRS of 5. Two individuals had Alzheimer’s dementia several years before iNPH. In progression styles of iNPH, the THC-preceding, the VE-preceding type, and the unclassified were four, nine, and three individuals, respectively. Almost all individuals with AVIM in the study progressed, although some were asymptomatic for at least 20 years.

**Conclusions**: The disorder of the ventricular system may be common in people in their 90s. The major style of iNPH progression at ages > 85 was not from AVIM but from the VE-preceding type, which was difficult to recognize for both symptoms and brain imaging in each person.

## A22 Idiopathic Normal Pressure Hydrocephalus (iNPH) in advanced age: profiling patients through quantitative variables

### Liliana Mazza^1^, Sevil Yaşar^2^, Alessandro Pirina^3^, Giulia Giannini^4^, David Milletti^5^, Fabiola Maioli^1^, Giorgio Palandri^3^

#### ^1^U.O.C. Percorsi Geriatrici Integrati, Dipartimento dell’Integrazione, Azienda USL di Bologna, Italia; ^2^Johns Hopkins School of Medicine, Department of Medicine and Neurology, Baltimore, USA; ^3^IRCSS Istituto delle Scienze Neurologiche di Bologna, UOC Neurochirurgia, Bologna, Italia; ^4^IRCCS Istituto delle Scienze Neurologiche di Bologna, Clinica Neurologica Metropolitana NEUROMET, Bologna, Italia; ^5^IRCCS Istituto delle Scienze Neurologiche, UOSI Medicina Riabilitativa, Bologna, Italia

##### **Correspondence**: Liliana Mazza, liliana.mazza3@gmail.com

*Fluids and Barriers of the CNS* 2023, **20(Suppl 2)**: A22

**Introduction**: Idiopathic Normal Pressure Hydrocephalus (iNPH) frequently affects people of advanced age. Gait and/or balance disturbance often occurs as the first symptom. For this reason, multiple scores are used to evaluate functional disability. The aim of the present study is to explore the gait and balance characteristics of iNPH subjects to define profiles of patients.

**Methods**: Data for the present analysis were provided by the Bologna PRO-Hydro multidisciplinary team and included patients with diagnosed iNPH from 2014 to 2023. Variables of interest include demographic (age, education), clinical (Rankin score, iNPH Grading Scale), and functional (Tinetti, Timed Up and Go score - TUG) data collected for a subset of patients. Principal Component Analysis (PCA) was adopted to unveil the latent traits of these individuals, while a clustering technique (K-means) was carried out on the two main dimensions of PCA (“tandem” approach) to distinguish two different groups of subjects.

**Results**: 138 patients with iNPH were included. The mean age was 75.4 years old (SD = 5.7) and 85 were males (62%). Gait disturbance was the first symptom in 57% of cases (79 patients), while balance disturbance was the first symptom in 20 patients (14%). Two main dimensions of PCA carried out on the six quantitative variables explained 63.5% of the total variability. The first dimension (45.1%) was mainly driven by clinical and functional scores (TUG, Tinetti, Rankin, iNPHGS) while, in the second dimension (18.4%), age and education were well represented. Cluster analysis designed two profiles of iNPH patients: those older, with worse functional scores, and those younger and/or educated with better performances.

**Conclusions**: Gait, balance and disability are the most relevant characteristics of subjects with iNPH. Nonetheless, age itself plays a non-negligible role, especially when combined with levels of education, to define iNPH patients’ profiles.

## A23 In 2023 is NPH still underdiagnosed? The social and economic burden of the problem

### Gianpaolo Petrella, Silvia Ciarlo, Graziano Taddei, Angelo Pompucci, Alessandro Pesce

#### ^1^A.O. “Santa Maria Goretti” General Hospital, Neurosurgery Department, Latina, Italy

##### **Correspondence**: Gianpaolo Petrella, gianpaolo_p@hotmail.com

*Fluids and Barriers of the CNS* 2023, **20(Suppl 2)**: A23

**Introduction**: Normal Pressure Hydrocephalus (iNPH) typically affects the elderly and, by leading to a cognitive decline, it enters in a differential diagnosis with other neurodegenerative conditions. However, it is considered to be underdiagnosed: this does not allow the patient to receive the right treatment and significantly affects quality of life and life expectancy.

**Methods**: The present investigation is an in-depth analysis of the real incidence of iNPH in the population of the province of our hospital (circa 580000 individuals). The first phase of this study was conducted by visualizing a brain CT done in a week in the emergency department. We visualized a total of 308 brain CT scans performed on patients accessing for different complaints in the Emergency Departments of the four hospitals of our network and screened those suspicious for iNPH. Subsequently, the corresponding Emergency Department medical records were investigated, with the aim of understanding the medical history of each patient in search of elements attributable to an alteration of the CSF dynamics.

**Results**: The cohort of positive CT scans, according to the radiological and clinical inclusion criteria included 48 patients. Among the reasons for acute medical care, “Fall” was the most common. The prevalence of CT scans suggestive of iNPH among the patients undergoing CT scans was as high as 15.58% and the incidence calculated on the basis of the total number of patients accessing the emergency departments was 1.084%.

**Conclusions**: The real incidence of iNPH in the population may be underestimated, and the social burden linked to the assistance of patients suffering from such untreated condition could be significantly relieved.

## A24 Levels of inflammatory cytokines MCP-1, CCL4 and PD-L1 in CSF differentiate idiopathic normal pressure hydrocephalus from neurodegenerative diseases

### Madelene Braun^1^, Gustaf Boström^2^, Martin Ingelsson^2^, Lena Kilander^2^, Malin Löwenmark^2^, Dag Nyholm^1^, Joachim Burman^1^, Valter Niemelä^1^, Eva Freyhult^3,4^, Kim Kultima^5^, Johan Virhammar^1^

#### ^1^Department of Medical Sciences, Neurology, Uppsala University, Sweden; ^2^Department of Public Health and Caring Sciences, Geriatrics, Uppsala University, Sweden; ^3^Department of Medical Sciences, National Bioinformatics Infrastructure Sweden, Science for Life Laboratory, Uppsala University, Sweden; ^4^Department of Cell and Molecular biology, Uppsala University, Sweden; ^5^Department of Medical Sciences, Clinical Chemistry, Uppsala University, Sweden

##### **Correspondence**: Madelene Braun, madelen.braun@akademiska.se

*Fluids and Barriers of the CNS* 2023, **20(Suppl 2)**: A24

**Introduction**: Neuroinflammation has been suggested to play a role in the pathophysiology of neurodegenerative diseases and posthemorrhagic hydrocephalus but has rarely been investigated in patients with idiopathic normal pressure hydrocephalus (iNPH). The aim of this study was to investigate whether the levels of inflammatory proteins in CSF are different in iNPH compared to healthy controls and patients with selected neurodegenerative disorders and whether any of these markers can contribute to the differential diagnosis of iNPH.

**Methods**: Lumbar CSF was collected from 172 patients from a single center and represented iNPH (n = 74), Alzheimer's disease (AD) (n = 21), mild cognitive impairment (MCI) due to AD (n = 21), stable MCI (n = 22), frontotemporal dementia (n = 13) and healthy controls (HC) (n = 21). Levels of 92 inflammatory proteins were analyzed by proximity extension assay. Protein differences between iNPH and HC were compared in a second step with the other groups. The linear regressions were adjusted for age, sex, and plate number.

**Results**: Three proteins showed higher (MCP-1, p = 0.0013; CCL4, p = 0.00082; CCL11, p = 0.0022) and one lower (PD-L1, p = 0.0051) levels in patients with iNPH compared with controls. Further MCP-1 was higher in iNPH than in all other groups. CCL4 was higher in iNPH than in all other groups except in MCI due to AD. PD-L1 was lower in iNPH compared with all other groups except in stable MCI. Levels of CCL11 did not differ between iNPH and the differential diagnoses. In a model based on the four proteins mentioned above, the area under the curve to discriminate between iNPH and the other disorders was 0.91.

**Conclusions**: The inflammatory cytokines MCP-1 and CCL4 are present at higher, and PD-L1 at lower, levels in iNPH than in the other investigated diagnoses. These three cytokines may have diagnostic potential in the diagnostic work-up of patients with iNPH.

## A25 Directed Topographical Changes to Reduce Cellular Adhesion and Ventricular Wall Pull in

### Aaron A. Gonzales, Carolyn A. Harris

#### ^1^Chemical Engineering and Materials Science, Wayne State University, Detroit, MI, 48201, USA

##### **Correspondence**: Aaron A Gonzales, AaronGonzales@wayne.edu

*Fluids and Barriers of the CNS* 2023, **20(Suppl 2)**: A25

**Introduction**: Ventricular shunt design has remained largely stagnant since its early development. Perhaps the most widely used advancement is the implementation of antibiotic impregnated catheters. Although this application has helped to improve device functionality, our biobank of explanted catheters still suggests that cellular attachment, growth, and tissue infiltration are prominent issues within the hydrocephalus community. Herein, we discuss the effects of nano to macro scale topographical features of human astrocyte adhesion and ventricular wall pull which can easily work in conjunction with anti-microbial agents to further reduce revisions rates.

**Methods**: The relationship between topographical features and cell adhesion, proliferation, and activation was determined using a high-throughput design that allowed the testing of unique patterns under static environments. Human astrocytes were cultured on the surfaces and analyzed using immunofluorescence and ELISA. Additionally, the effect of macroscopic changes to the catheter surface was analyzed. The likelihood of tissue pull in to occur was investigated in vitro and with computer simulations.

**Results**: The results showed that the topographical changes had an effect on astrocyte adhesion, proliferation, and activation. The degree of pull in of large ventricular wall tissue masses was manipulated by macroscopic size changes in surface structure. It was found that the likelihood of tissue pull in to occur was lower when these structures were utilized compared to the control.

**Conclusions**: The use of nano- to microscale topographical features to manipulate cellular effects to achieve a desired outcome is new to the field of hydrocephalus. We believe that this emerging field of mechanobiology and functional macrostructures can be used to create a new generation of highly engineered catheters that reduce adhesion and pull in. Most promising of all is that this technology can be used with antibiotic coating technologies with hardly any difference to the naked eye.

## A26 An In-vitro Setup for Testing of Ventricular Catheters under Physiologic and Pathophysiologic Flow Rates

### Ahmad Faryami^1^, Adam Menkara^1^, Shaheer Ajaz^1^, Christopher Roberts^2^, Carolyn A. Harris^2^

#### ^1^Biomedical Engineering, Wayne State University, Detroit, Michigan,48202 USA; ^2^Chemical Engineering and Material Science, Wayne State University, Detroit, Michigan,48202 USA

##### **Correspondence**: Ahmad Faryami: gw7895@wayne.edu

*Fluids and Barriers of the CNS* 2023, **20(Suppl 2)**: A26

**Introduction**: The mechanism of shunt failure is still not fully understood; A variety of methods including in-vitro experiments, in-vivo animal studies, and clinical studies have been utilized to identify the mechanism of obstruction formation that preludes shunt failure. These studies have primarily focused on endpoint analysis of the catheter without incorporating any dynamic, quantitative, or qualitative outcomes throughout the study. The absence of a tested and validated, long-term in-vitro model has significantly limited progress in understanding the pathogenesis of obstruction formation on ventricular catheters and improving treatment. Therefore, a novel setup was developed to recapitulate the relevant shear stresses and to investigate the interaction between immune cells and shunt hardware.

**Methods**: The setup consisted of three main components: A control unit, positive displacement pumps, and bioreactor chambers. Computational fluid dynamics (CFD) simulations were utilized to design the bioreactor chambers. The capacity, consistency, and long-term fluidic performance of 135 purpose-built bioreactor chambers were investigated. Physiologic and pathophysiologic CSF flow rates were simulated through the bioreactor chambers and the flow of CSF through a partially obstructed explanted catheter was visualized using confocal microscopy.

**Results**: No leaks or ruptures were observed in bioreactor chambers. All chambers withstood 10 pressurization cycles to 140.7 cmH2O gauge pressure: significantly beyond the reported ICP domain (0–25 cmH2O) in most pediatric and adult hydrocephalus patients. There were no statistically significant differences between the bulk flow output of the setup at 0 Days, 15 Days, and 30 Days respectively.

**Conclusions**: the use of bioreactors as an intermediary between computer models and complex biological systems in the context of hydrocephalus treatment was previously proposed. This in-vitro setup is the first high-throughput testing platform for recapitulating patient-specific in-vivo conditions and rigorous real-time data collection across up to 50 concurrent channels.

## A27 Novel 3D Printed Microfluidic Chip for Hydrocephalus Treatment: Fabrication and In vitro Evaluation

### Seunghyun Lee^1^, Leandro Castaneyra-Ruiz^1^, Jenna Ledbetter^1^, Michael Le^1^, Michael Muhonen^2^

#### ^1^CHOC Children’s Research Institute, Orange, CA 92868, USA; ^2^Neurosurgery department at CHOC Children’s Hospital, Orange, CA 92868, USA

##### **Correspondence**: Seunghyun Lee, Seunghyun.Lee@choc.org

*Fluids and Barriers of the CNS* 2023, **20(Suppl 2)**: A27

**Introduction**: To overcome the limitations of current shunt systems, which are prone to catheter-related infections and blockages, innovative solutions are required. To this end, we introduce a new microfluidic chip that utilizes 3D printing technology constructed of biocompatible flexible resin, eliminating the need for catheters, and offering an alternative approach for the treatment of communicating hydrocephalus.

**Method**: The microfluidic chip is designed to shunt CSF from the subarachnoid space to a venous sinus. It has a ring-shaped structure at the inlet to prevent CSF backflow, sealing the inlet hole at low ICP and deflecting upwards when ICP surpasses the target chip opening pressure determined by device dimensions. Flow/pressure performance was measured across the chip under various inlet pressures (-100 to 100 cmH_2_O) with a flow rate of 450 µl/min. Astrocytes were cultured on the device to assess potential cell obstructions.

**Results**: The microfluidic chip effectively served as an alternative shunt system in benchtop functional tests. In response to pressure changes due to input flow accumulation, the chip effectively drained excess flow with an opening pressure of 50 to 100 mmH_2_O and prevents backflow to at least − 400 mmH_2_O. The chip also showed few cellular attachments and obstructions.

**Conclusion**: The study presents a promising microfluidic chip for the treatment of hydrocephalus, fabricated by 3D printing technology. The chip provides a solution to the limitations of current shunts with following novel functions: i) Enabling CSF shunting without catheters ii) Minimizing cellular blockage, and iii) Simplicity in design/fabrication process. Future studies will focus on in vivo experiments to validate the efficacy and safety of the chip in the treatment of hydrocephalus.

## A28. A model of normal pressure hydrocephalus (NPH) as a disorder of intracranial thermodynamics

### Michael Egnor^1^, Liu Yang^2^, Racheed Mani^1^, Susan Fiore^1^, Peter Djuric^2^

#### ^1^Department of Neurological Surgery, Stony Brook University Hospital, Stony Brook, New York, USA; ^2^Department of Electrical and Computer Engineering, Stony Brook University, Stony Brook, New York,USA..

##### **Correspondence**: Michael Egnor, michael.egnor@stonybrookmedicine.edu

*Fluids and Barriers of the CNS* 2023, **20(Suppl 2)**: A28

**Introduction**: The etiology of NPH remains obscure. Using flow MRI, investigators have shown that NPH is characterized by diminished CSF pulsatility in the subarachnoid space, increased CSF pulsatility in the ventricles, and increased pulsatility in the brain parenchyma compared with controls. This redistribution of CSF pulsatility is caused by restriction of wall motion in the large subarachnoid arteries due to arteriosclerosis, and ventriculomegaly is caused by increased pulse pressure on the periventricular white matter. The increase in brain pulsatility implies that NPH is a disorder of the cerebral windkessel.

We have developed a thermodynamic model of the windkessel as a tuned band stop filter centered at the heart rate, and we apply this model to a new understanding of NPH based on MRI flow evidence.

**Methods**: We use a simple tuned electrical tank circuit model of the cerebral windkessel. The effectiveness of the windkessel is (W is windkessel effectiveness, I is inertance which is the size of the pulse, E is the elastance of the CSF space, and R is the resistance to the pulse):

$$W\hspace{0.17em}=\hspace{0.17em}\frac{IE}{R}$$

We model NPH as a disorder of the cerebral windkessel by decreasing the windkessel inertance (which corresponds to diminished CSF pulsatility in the subarachnoid space caused by arteriosclerosis of large subarachnoid arteries) and increasing brain compliance (due to age-related brain atrophy)

**Results**: Simulation shows decreased subarachnoid pulsatility, increased ventricular pulsatility, and increased brain parenchymal pulsatility.

**Conclusions**: NPH is characterized by high impedance to AC power in the subarachnoid CSF path due to arteriosclerosis and high subarachnoid CSF compliance due to brain atrophy, with redistribution of AC power to the ventricular CSF path and high amplitude pulsatile stress on the periventricular white matter. This can be simulated in a circuit model of the cerebral windkessel. We propose that NPH is a disorder of intracranial thermodynamics.

## A29 Computer vision pipeline to synchronize cranio-spinal pressures with posture in chronic in-vivo trials

### Anthony Podgorsak^1^, Fabian Flürenbrock^2^, Andreas Kaufmann^3^, Nina Eva Trimmel^4^, Margarete Arras^4^, Markus Florian Oertel^5^, Miriam Weisskopf^4^, Marianne Schmid Daners^2^

#### ^1^Product Development Group Zurich, Department of Mechanical and Process Engineering, ETH Zurich, Zurich, 8092, Switzerland; ^2^Institute for Dynamic Systems and Control, Department of Mechanical and Process Engineering, ETH Zurich, Zurich, 8092, Switzerland; ^3^Department of Computer Science, ETH Zurich, Zurich, 8092, Switzerland; ^4^Center for Preclinical Development, University Hospital Zurich, University of Zurich, Zurich, 8092, Switzerland; ^5^Department of Neurosurgery, University Hospital Zurich, University of Zurich, Zurich, 8092, Switzerland

##### **Correspondence**: Marianne Schmid Daners, marischm@ethz.ch

*Fluids and Barriers of the CNS* 2023, **20(Suppl 2)**: A29

**Introduction**: Animal studies allow multimodal data to be acquired under more diverse conditions when compared to humans. Postural impacts on physiological pressures have been studied, however, a unified pipeline to synchronize, quantify, and analyze pressures to posture in chronic settings is not yet developed.

**Methods**: A custom computer vision pipeline was developed. After pressure (via telemetric sensors) and video (via dual-camera stereoscopic setup) data are acquired, 15 2-Dimensional keypoints are detected on each frame and triangulated into 3-Dimensions. Three anatomical angles, (a) abdominal and (b) neck both relative to ground and (c) angle between neck and abdomen are calculated and synchronized with pressure data into a single dataset. Finally, the sheep’s position is classified into seven buckets (standing, laying, walkup, walkdown, kneeling, sitting, other). 2/8 pressures are evaluated in three sheep: intracranial (ICP) and lumbar intrathecal (ITP) pressure. Average precision (AP) and percent correct classifications were used to evaluate the pipeline’s performance.

**Results**: The pipeline detected key points with an AP of 97.5. The position classifier classified positions with 95.2% accuracy (465/488 positions). Of the evaluated sheep, a Pearson correlation of −0.78 (0.74) was found between ICP (ITP) and angle (a) of 96.2±1.4° during chair postures. Similarly, correlations of −0.77 (0.39) were found between ICP (ITP) and angle (b) of 59.7±4.8° during walkup postures. During walkup and kneeling positions, angle (b) influenced ICP more strongly than angles (a) and (c) (−0.77 (b) vs. −0.33 (a) and −0.28 (c) during walkup and −0.62 (b) vs. −0.43 (a) and 0.13 (c) during kneeling).

**Conclusions**: The pipeline shows great promise and flexibility as an all-in-one analysis tool for multimodal datasets in chronic in-vivo trials. Insights gained from this pipeline can not only improve our understanding of cranio-spinal and adjacent physiology but also support the development of novel hydrocephalus shunts.

## A30 Prolonged Spaceflight Alters Intracranial Tissues, Fluids and Ocular Morphology

### Katherine G. Warthen^1^, Stuart H. Sater^1^, Gabryel Conley Natividad^1^, Larry A. Kramer^2^, Khader M. Hasan^2^, Michael A. Williams^3^, Brandon R. Macias^4^, Steven S. Laurie^5^, Bryn A. Martin^1^

#### ^1^Alcyone Therapeutics, Moscow, ID, USA; ^2^University of Texas Health Science Center, Houston, TX, USA; ^3^University of Washington School of Medicine, Seattle, Washington, USA; ^4^NASA Johnson Space Center, Houston, TX, USA; ^5^KBR, Houston, TX, USA

##### **Correspondence**: Bryn Martin, bryn@alcyonetx.com

*Fluids and Barriers of the CNS* 2023, **20(Suppl 2)**: A30

**Introduction**: Long duration spaceflight (LDSF) is known to affect both the eye and brain, although exact effects are still being quantified. Astronauts experience prolonged reduced gravitational pull, which can result in increased ventricular cerebrospinal fluid (CSF) volume, displacement of the posterior optic globe, and optic disc edema and choroidal engorgement along with brain structure alterations. We analyzed ophthalmic globe displacement, brain and CSF volumes, and brain and CSF shifts within the skull in a group of 11 astronauts (~6 months in space) compared to a group of healthy controls (n = 10).

**Methods**: We used a previously validated in-house method to automatically segment the eye. Brain and CSF tissues were automatically segmented using Charm. FSL was used to calculate volumes and center of gravity of each tissue type. A control group was used to determine both the reliability of the technique as well as how much natural variation may exist in healthy adults.

**Results**: We found a significant increase in optic globe volume displacement among astronauts (p = 0.002, mean = 8.97mm^3^), and a lack of change among controls. Astronauts also showed significant increases in gray matter volume (p = 0.007, mean = 7.97 cm^3^), trending increases in white matter volume (p = 0.083, mean = 5.96 cm^3^) and an increase in lateral ventricular volume in all but one astronaut case (p = 0.002, mean = 3.2mL). We also observed a dorsal brain shift (p = 0.04, mean = 0.62mm) and ventral (p = 0.001, mean = -2.24mm) and anterior total intracranial CSF shift (p = 0.002, mean = 1.03mm) as calculated by center of gravity. None of these changes were observed in the control group.

**Conclusions**: Long duration spaceflight was found to be associated with significant increases in ophthalmic globe displacement, brain volume and ventricle increases, as well as brain and CSF shift within the skull. This methodology could be applied to quantify brain and CSF shifts in a multitude of disorders, including hydrocephalus and idiopathic intracranial hypertension.

## A31 Modeling congenital hydrocephalus genes SMARCC1 and TRIM71 in the Xenopus model system

### Stephen Viviano^1^, Amrita Singh^2^, Engin Deniz^3^

#### ^1^Department of Pediatrics, Yale University, New Haven, CT, 06525, USA; ^2^Department of Neurosurgery, Yale University, New Haven, CT, 06525, USA

##### **Correspondence**: Engin Deniz, engin.deniz@yale.edu

*Fluids and Barriers of the CNS* 2023, **20(Suppl 2)**: A31

**Introduction**: Congenital hydrocephalus (CH), characterized by the pathological expansion of the brain ventricles, is a common birth defect affecting ~ 1/1000 births. Studies estimate that ~40% of cases have a genetic etiology, yet only a few candidate genes have been studied. TRIM71 and SMARRC1 have recently been identified as CH-risk genes. To analyze their role in hydrocephalus, we developed an innovative approach that leverages the genetically tractable frog *Xenopus*. The tadpole brain is semi-transparent, enabling Optical Coherence Tomography (OCT) imaging, which allows us to visualize the entire embryonic CSF circulation in 3D in real-time and can demonstrate the hallmarks of human hydrocephalus including aqueduct stenosis and ventriculomegaly. By combining CRISPR-based gene depletion with the *Xenopus* system's speed, low cost, and efficiency, we have transformed our ability to investigate candidate CH genes. Using the *Xenopus* model, we evaluated TRIM71 and SMARCC1 for hydrocephalus phenotypes.

**Methods**: We generated *trim71* and *smarcc1* G0-*Xenopus* mutants using CRISPR/Cas9. Starting post-neurulation, we performed OCT imaging to analyze brain morphology to determine precisely when CH phenotypes develop and examined pathogenesis using *in situ* hybridization and immunofluorescence.

**Results**: *smarcc1* mutant tadpoles exhibited aqueduct stenosis that was transmitted to G1 mutant progeny. In contrast, when we depleted *trim71,* tadpoles developed severe ventriculomegaly. Both results phenocopied the core brain pathology of the patients. Neither gene affected CSF circulation, and further analysis revealed cortical and midbrain dysgenesis in both cases resulting from alteration of the expression of key transcriptional regulators involved in neural progenitor growth and proliferation.

**Conclusions**: We show that OCT imaging coupled with the *Xenopus* model system is an efficient platform to functionally screen candidate CH genes and improve our understanding of hydrocephalus pathogenesis. Our data further provide evidence that *Xenopus* SMARCC1 and TRIM71 affect early neurogenesis rather than CSF circulation, which may not be the primary defect in these subtypes.

## A32 Genetic determinants and molecular mechanisms of human cerebral ventricular size and congenital hydrocephalus

### Garrett Allington^1,2^, Kedous Y. Mekbib^2^, Evan Dennis^2^, Emre Kiziltug^2^, Stephen McGee^3^, Sheng Chih Jin^4,5^, Kristopher T. Kahle^2,6-8^

#### ^1^Department of Pathology, Yale University School of Medicine, New Haven, CT, 06511, USA; ^2^Department of Neurosurgery, Massachusetts General Hospital, Boston, MA, 02114, USA; ^3^GeneDx, Gaithersburg, MD, 20697, USA; ^4^Department of Genetics, Washington University School of Medicine, St. Louis, MO, 63130, USA; ^5^Department of Pediatrics, Washington University School of Medicine, St. Louis, MO, 63130, USA; ^6^Division of Genetics and Genomics, Manton Center for Orphan Disease Research, Department of Pediatrics, and Howard Hughes Medical Institute, Boston Children's Hospital, Boston, MA, 02115, USA; ^7^Broad Institute of MIT and Harvard, Cambridge, MA, 02142, USA; ^8^Harvard Center for Hydrocephalus and Neurodevelopmental Disorders, Massachusetts General Hospital, Boston, MA, 02114, USA

##### **Correspondence**: Kristopher T. Kahle, Kahle.Kristopher@mgh.harvard.edu

*Fluids and Barriers of the CNS* 2023, **20(Suppl 2)**: A32

**Introduction**: The leading cause for pediatric neurosurgical intervention, congenital hydrocephalus (CH), affects 1/1,000 live births and is associated with neurodevelopmental disorders, but CH etiology remains mysterious. Currently, surgical cerebrospinal fluid (CSF) shunting or endoscopic third ventriculostomy procedures aimed at reducing CSF accumulation, are the mainstay of treatment for patients with CH. While these procedures could be lifesaving, they carry high rates of morbidity, complication, and failure, often requiring lifelong neurosurgical management. These outcomes reflect a poor understanding of the underlying pathogenic mechanisms.

**Methods**: To advance our understanding of CH pathogenesis we performed an integrative analysis of exomes from 2,697 CH patient-parent trios as well as 1,798 control trio exomes, 848,020 single cell transcriptomes of the developing human brain, 432 histologically verified developing brain tissue samples from post-conception week 9 to post-natal age 3, and artificial intelligence-assisted clinical phenotype profiling of proband medical records.

**Results**: We found damaging, de novo variants were significantly enriched in the CH cohort relative to control (P = 3.03 × 10^−122^) with 35 genes reaching the threshold for genome-wide significance. Weighted genome correlation network analysis revealed significant enrichment of CH genes in processes related to epigenetic regulation and neuro-gliogenesis (P = 7.36 × 10^−48^) in early/mid-fetal development (P = 1.26 × 10^−11^). AI-assisted clinical phenomic and complementary risk gene homology analysis found significant enrichment for neurodevelopmental disorders (P = 3.16 × 10^−73^) and hypotonia (P = 7.72 × 10^−71^), with phenotypic frequency associated with variants in specific genes.

**Conclusions **Together, these analyses suggest CH exists as a manifestation of a deeper insult to neurodevelopment, rather than simply a disease of CSF malabsorption. Moreover, in some cases correlated to specific risk genes, CH phenotypes may arise from impaired mid-fetal cortical neuro-gliogenesis and cell migration. Better patient outcomes can be realized by considering genomic factors in neurosurgical decision-making.

## A33 Cerebrospinal fluid oscillations and cerebral blood flows in hydrocephalic premature infants who have had intraventricular hemorrhage

### Olivier Balédent^1,2^, Margaux Aye^3^, Cyrille Capel^2,4^, Catherine Gondry-Jouet^2,3^

#### ^1^Image processing, University hospital, Amiens, France; ^2^CHIMERE UR7516, Jules Verne University , Amiens, France; ^3^Radiology, University hospital, Amiens, France; ^4^Neurosurgery, University hospital, Amiens, France

##### **Correspondence**: Olivier Balédent, olivier.baledent@chu-amiens.fr

*Fluids and Barriers of the CNS* 2023, **20(Suppl 2)**: A33

**Introduction**: Intraventricular hemorrhage (IVH) in newborns induces hydrocephalus that must be treated surgically in the worst situations. Morphological MRI can describe the morphology of the brain and the bleeding location but cannot describe how active is the hydrocephalus. We previously have shown that Phase contrast MRI (PCMRI) can quantify neurofluids in newborn. Today our objective is to evaluate the IVH impact on CSF oscillations and blood flows in hydrocephalic premature infants.

**Methods**: 15 preterm infants born between 24 and 34 weeks of amenorrhea who had IVH underwent morphological MRI and PCMRI between 34 weeks of amenorrhea and 6 years. We quantified CSF flows in the aqueduct of Sylvius and in the spinal canal. In addition, we quantified cerebral arterial and venous blood flow.

**Results**: Dilatations were bi, tri or tetra ventricular. The aqueduct was stenotic in 1 patient of normal caliber in 5 patients and was widened in 9 patients. The CSF flow in the spinal canal was very low in 8 subjects, hyper dynamic in 1 and normal for the others. CSF flow in the aqueduct was null in 7 patients, very low in 4, normal and hyper dynamic respectively in 2. Morphological analysis of the spinal canal did not allow apprehending the PCMRI results. For the aqueduct the concordance was present in only 3 cases. Cerebral arterial blood flow varied as a function of age between 33 ml/min to 1162 ml/min and was correlated (R2 = 0.77) with jugular veins flow. Oscillatory spinal CSF volume correlated (R2 = 0.91) with cerebral volume expansion during cardiac cycle.

**Conclusions**: PC-MRI can quantify CSF and blood flows in the newborn within 5 min. It highlights CSF blockage or hyper dynamism when morphologic analysis does not. It provides complementary information to the morphological analysis that could be useful to understand the pathophysiology of IVH and help in surgical decisions.

## A34 CSF-based extracellular vesicle signaling and related T-cell activation mediate the pathogenesis of post-hemorrhagic hydrocephalus

### Maria Garcia Bonilla^1^, David Giles^1^, Emre Kiziltug^2^, Diego Morales^1^, Corrine Gardner^4^, Kirill Shumilov Bartenev^1^, Pat MacAllister^1^, Kristopher Kahle^3^, David Limbrick^1^

#### ^1^Washington University in St. Louis School of Medicine, Missouri, USA; ^2^Yale School of Medicine, Connecticut, USA; ^3^Pediatric Neurosurgery at the Massachusetts General Hospital and the Harvard Center for Hydrocephalus and Neurodevelopment, Massachusetts, USA; ^4^St. Louis Children's Hospital, Missouri, USA

##### **Correspondence**: Maria Garcia Bonilla, mariag@wustl.edu

*Fluids and Barriers of the CNS* 2023, **20(Suppl 2)**: A34

**Introduction**: Preterm intraventricular haemorrhage (IVH) is closely associated with the development of post-hemorrhagic hydrocephalus (PHH), the most common cause of paediatric hydrocephalus in the industrialized world. PHH neuropathology is multifactorial and includes ventricular/subventricular zone (V/SVZ) disruption, inflammation, and subsequent eruption of cells into the cerebrospinal fluid (CSF). Extracellular vesicles (EVs) are involved in intercellular communication and may contribute to the neuropathogenesis of the disease. We hypothesized that CSF-based EV signaling, and related CSF T-cell activation mediate the pathogenesis of PHH.

**Methods**: EV and cell profiles from CSF of human neonates with PHH were compared to samples from IVH grade 1–2, congenital hydrocephalus (CH), and controls (no known neurological injury). EVs were isolated and analyzed by mass spectrometry-based high-throughput proteomics. Cells were analyzed by single-cell RNA sequencing (scRNAseq) and flow cytometry. T-cell activation after EV exposure was studied *in vitro* by RNAseq, flow cytometry, and ELISA; and in post-mortem brain samples by immunofluorescence.

**Results**: PHH CSF samples contained a significant increase in EV pro-inflammatory proteins compared to control and CH. Furthermore, monocytes/macrophages as well as a robust population of activated T-cells were detected in the CSF of PHH cases. EVs activated T-cells to produce the pro-inflammatory cytokines interleukin 1 beta (IL1β), IL6, and tumor necrosis factor-alpha (TNFα) in PHH. EV-mediated activation in T-cells occurred through the Nf-kB pathway. Finally, T-cell recruitment and the same cytokine production were detected in the choroid plexus of post-mortem IVH/PHH samples.

**Conclusions**: PHH is associated with increased pro-inflammatory EVs that can activate T-cells to produce pro-inflammatory cytokines, exacerbating the inflammatory milieu in the CSF and choroid plexus. Thus, neuroinflammation and modulation of stem cell biology for neural injury and repair after PHH may be mediated through EV signals and T-cells. Defining EV and cell profiling will lead to new directions to improve outcomes for patients with hydrocephalus.

## A35 Patterns of Clinical Presentation of Pediatrics Hydrocephalus at The National Center for Neurological Sciences . 2017–2021

### Nidaa Ahmed^1^, Arwa Nasr^1^, Asma Abdalla^2^, Safa Hamid^1,3^

#### ^1^Department of Surgery, University of Khartoum, 11111, Sudan; ^2^Department of Community, University of Khartoum, 11111, Sudan; ^3^Department of Neurosurgery, National Center for Neurological Sciences, Khartoum, 11111, Sudan

##### **Correspondence**: Nidaa Munir Osman Ahmed, nidaamunir98@gmail.com

*Fluids and Barriers of the CNS* 2023, **20(Suppl 2)**: A35

**Introduction**: Hydrocephalus is an increase in CSF (Cerebrospinal Fluid) consequent to physical or functional obstruction, or increased CSF production leading to ventricular system enlargement. The worldwide incidence of HCP (Hydrocephalus) is more than 380,000, mainly in Africa, Latin America, and Southeast Asia. The disease can be classified as congenital and acquired (or secondary to e.g. hemorrhage, infection, or neoplasms), with the post-infective HCP being the most frequent etiology in pediatrics. Clinical presentation varies; in infants includes abnormally increasing head size, irritability, vomiting, bulging anterior fontanelle, and splaying of the cranial sutures, while beyond infancy, comprises headache, vomiting, loss of developmental milestones, diplopia, and papilledema. The principal investigation is brain imaging. The treatment involves CSF diversion, e.g. temporal with the use of external drains and permanent using the Ventriculoperitoneal Shunts.

Methods: Hospital-based retrospective descriptive cross-sectional study at the National Center for Neurological Sciences (NCNS) in Khartoum, Sudan, including all pediatric cases aged 1 day to 18 years with HCP from years 2017 to 2021.

Results: The study included 555 cases. The male-to-female ratio was 1.2:1. 471 cases (84.86%) presented between the age of 1 day to 3 years, mostly 148 were between 2 and 3 months. The commonest presenting symptom was abnormally increasing head size in 367 cases (66.13%), with a maximum head circumference of 81 centimeters. The most common type was congenital in 384 cases (69.19%), while the acquired was 160 cases (29%) with 74 obstructive due to neoplasms and 72 post-infective.

Conclusions: Hydrocephalus is considered to be a serious problem in pediatrics in Sudan, with a distinctive clinical picture due to late presentation; significantly the enlarged head sizes, and in divergence from the literature, the dominant type was congenital suggesting the prospect of the genetic predisposition of this disease or the successful prevention and treatment efforts for the post-infective cases.

## A37 Adjustable ventriculoperitoneal shunt valve for the treatment of hydrocephalus in newborns

### T. Freiman^1^, A. Cattani^2^, A. Spyrantis^2^, S. Schubert-Bast^3^

#### Departments of ^1^Neurosurgery Medical Centre Rostock, Schillingallee 35, 18057 Rostock, Germany; ^2^Neurosurgery; ^3^Neuropediatrics, Frankfurt University Hospital, Theodor Stern Kai 7, 60590 Frankfurt am Main, Germany

##### **Correspondence**: Thomas Freiman thomas.freiman@med.uni-rostock.de

*Fluids and Barriers of the CNS* 2023, **20(Suppl 2)**: A37

**Introduction**: Ventriculoperitoneal shunts (VPS) with adjustable differential pressure valves are commonly used to treat hydrocephalus in infants under 6 months old. However, determining the optimal pressure valve values for these patients can be challenging. The aim of this study was to analyze the influence of VPS adjustable differential pressure valves on head circumference (HC) and ventricular size (VS) stabilization in infants with post intraventricular hemorrhage, acquired and congenital hydrocephalus.

**Methods**: In this prospective study conducted for 4 years, 43 hydrocephalic infants under 6 months old were treated with a VPS with an adjustable differential pressure valve. HC and transfontanelle ultrasonographic VS measurements were regularly performed, and valve modifications were made when HC measurement was below the 10th percentile or exceeded the 90th percentile. The patients were divided into two groups based on the cause of hydrocephalus: intraventricular hemorrhage (IVH) and other etiologies (OE).

**Results**: The results showed that the mean pressure valve modification per patient was 4 cm H_2_O in the IVH group and 3 cm H_2_O in the OE group. The median of the last pressure valve value was higher at 8.5 cm H_2_O in the IVH group compared to 5 cm H_2_O in the OE group. This suggests that infants with IVH may need a higher pressure valve value compared to infants with OE. After a mean follow-up of 18 months and several pressure valve modifications, normalizing HC and VS was possible.

**Conclusion**: In conclusion, determining the optimal pressure valve value for infants with hydrocephalus can be challenging. However, this study suggests that long-term follow-up and several pressure valve modifications can lead to the normalization of HC and VS. Infants with IVH may require a higher-pressure valve value compared to infants with OE.

## A38 What can be learned from hydrocephalus induction failures?

### Pat MacAllister^1^, Maria Garcia Bonilla^1^, Jason Moore^1^, Michael Talcott^1^, Albert Isaacs^2^, Leandro Castaneyra Ruiz^3^, Diego Morales^1^, David Limbrick^1^

#### ^1^Washington University in St. Louis School of Medicine, Missouri, USA; ^2^Ohio State University and Nationwide Hospital, Ohio, USA; ^3^Children's Hospital of Orange County, California, USA

##### **Correspondence**: Maria Garcia Bonilla mariag@wustl.edu

*Fluids and Barriers of the CNS* 2023, **20(Suppl 2)**: A38

**Introduction**: For more than a century, preclinical models of acquired hydrocephalus have been developed using a wide variety of induction methods and vertebrate species. Practically all are designed to promote ventriculomegaly. The most popular have been chosen in part to optimize the severity of ventriculomegaly, the yield of animals that exhibit hydrocephalus, and the overall cost of the experiments.

**Methods**: This review seeks to summarize experimental induction methods and the extent of ventriculomegaly achieved, as well as the possible neurobiological mechanisms for the lack of ventriculomegaly.

**Results**: The most widely-used induction methods include mechanical and/or inflammatory impairment of cerebrospinal fluid pathways (e.g., intracisternal kaolin) and intraventricular or intracerebral injections of blood or blood products. No induction methods produce ventriculomegaly in 100% of attempts, although many achieve 70–95% success rates. These “induction failures” are usually omitted from further analyses, and no reports have emerged that include these “negative” results, even though these brains have been subjected, at least for a short time, to perturbations that usually cause hydrocephalus. This is unfortunate, because much can be learned about the threshold for ventriculomegaly and the potential endogenous mechanisms that may counteract the cause and progression of hydrocephalus. Furthermore, the lack of research on induction failures avoids the fact that, depending on the primary event, a relevant number of patients subjected to the main causes of hydrocephalus do not develop this disorder. For example, only about 30% (published estimates vary) of children with documented intraventricular hemorrhage (IVH) develop post-hemorrhagic hydrocephalus. This correlates well with our 10–15% rate for infant ferrets with IVH that develop ventriculomegaly, and the 15% of juvenile pigs that do not develop ventriculomegaly post-kaolin.

**Conclusions**: Research into hydrocephalus “induction failures” is timely and should provide new insights into the causes and treatments for hydrocephalus.

## A39 Modulation of Toll-like receptor (TLR)-calpain pathway may prevent acquired hydrocephalus

### Leandro Castaneyra-Ruiz^1^, Jenna Ledbetter^1^, Seunghyun Lee^1^, Anthony Rangel^1^, Evelyn Torres^1^, Michael Muhonen^2^

#### ^1^CHOC Children’s Research Institute, Orange, CA 92868, USA; ^2^Neurosurgery department at CHOC Children’s Hospital, Orange, CA 92868, USA

##### **Correspondence**: Leandro Castaneyra-Ruiz, Leandro.castaneyra.ruiz@choc.org

*Fluids and Barriers of the CNS* 2023, **20(Suppl 2)**: A39

**Introduction**: Adherens junctions (AJ) disruptions are crucial in the pathogenesis of congenital hydrocephalus, as they affect the stability of the ventricular zone (VZ) and the endothelial cells. It has been reported in vitro, in vivo, and in postmortem human subjects that alterations in AJ are associated with the origin of acquired hydrocephalus. Recently, several authors have proposed inflammatory mechanisms as the primary trigger of acquired hydrocephalus. Thus, Toll-like receptors (TLR) recognize pathogen-associated molecular patterns (PAMPs) such as toxins or bacteria and damage-associated molecular patterns (DAMPs) like blood or blood subproducts as inflammatory triggers. Therefore, PAMPs and DAMPs are involved in the origin of postinfectious (PIH) and posthemorrhagic hydrocephalus (PHH). Calpains are intracellular proteases that activate when TLR detects DAMPS or PAMPS. Preliminary data support the hypothesis that calpains may underlie AJ cleavage under both conditions, PAMPs or DAMPs, as a fundamental trigger in the origin of hydrocephalus. This project aimed to modulate the TLR-Calpain pathway as a possible treatment to prevent acquired hydrocephalus.

**Methods**: Calpeptin (calpain inhibitor) was used to prevent VZ, endothelial disruption, and ventriculomegaly. The activity of this inhibitor was tested in 1–3 days old C57BL mice inoculated intraventricularly with LPS. After 15 days of treatment, histology was performed to evaluate the integrity of AJ and the viability of the VZ and endothelial cells. MRI was used to quantify ventricular volumes.

**Results**: The inhibition of the TLR-Calpain pathway after LPS intraventricular injection significantly increased the animals’ survival and reduced the ventricular volumes compared to untreated mice (p < 0.05). Calpeptin also contributed to maintaining the integrity of the endothelial cells and the VZ.

**Conclusions**: This research opens the possibility of using calpeptin to treat acquired hydrocephalus.

## A40 Brain pathologic change due to ciliary dysfunction in chronic hydrocephalus model mice

### Kaito Kawamura^1,2^, Madoka Nakajima^2^, Masakazu Miyajima ^3^, Chihiro Akiba^3^, Koichiro Sakamoto^2^, Kou Horikoshi^1,2^, Ryo Miyahara^2^, Chihiro Kamohara^2^, Ikuko Ogino^2^, Shinya Yamada^2,4^, Kostadin Karagiozov^2^, Akihide Kondo^2^

#### ^1^Department of Neurosurgery, Saiseikai Kawaguchi General Hospital, Saitama, Japan; ^2^Department of Neurosurgery, Juntendo University School of Medicine, Tokyo, Japan; ^3^Department of Neurosurgery, Juntendo Koto Geriatric Medical Center, Tokyo, Japan; ^4^Department of Neurosurgery, Kugayama Hospital, Tokyo, Japan

##### **Correspondence**: Kaito Kawamura, k-kawamu@juntendo.ac.jp

*Fluids and Barriers of the CNS* 2023, **20(Suppl 2)**: A40

**Introduction**: We have previously reported the DNAH14-/- mice model of a chronic hydrocephalus harboring ciliary dysfunction, that demonstrated chronic progression of ventriculomegaly followed by cognitive decline. Although ciliary dysfunction is known as a possible cause of hydrocephalus, its pathology remains unclear. The present study aims to evaluate brain pathologic changes in DNAH14-/- mice caused by ciliary dysfunction.

**Methods**: To evaluate brain pathologic change in DNAH14-/- mice, we performed microarray analysis using hippocampus collected from 16 week-old mice, followed by reverse transcription qPCR (RTqPCR) using 8, 16, 24 and 48 weeks old mice for validation. To evaluate neuro inflammatory changes in hippocampus (CA1/2, CA3, hilus), we performed immunohistochemistry (GFAP and Iba1) to measure the number of astrocytes and microglia using 48 weeks old mice.

**Results**: Microarray analysis showed hypo-expression of RNA related to dopamine receptor (GPR88, DRD2) in DNAH14-/- mice compared with WT. RTqPCR showed decreased expression in both GPR88 and DRD2 at 24 and 48 weeks, while not at 8 and 16 weeks. Immunohistochemistry showed increased number of GFAP or Iba1 positive cells in DNAH14-/- mice compared with WT in each region (p = 0.006, 0.006, 0.003 for GFAP, < 0.001, < 0.001, 0.044 for Iba1, respectively).

**Conclusions**: DNAH14-/- mice showed hypo-expression of RNA related to dopamine receptors in 24-week-old mice. Immunohistochemistry showed neuro-inflammation in hippocampus of DNAH14-/- mice. As it was reported that hippocampus-entorhinal cortex network is engaged in spatial memory, these results are matching the cognitive decline in 24-week-old DNAH14-/- mice. From these results, impaired neuro-transmission due to neuro-inflammation in hippocampus can be considered a key pathology for cognitive decline in chronic hydrocephalus. The results from RTqPCR imply that impairment of neurotransmission in DNAH14-/- mice is observed later in life and not congenitally, and has still unclear relation to ciliary dysfunction that will be clarified in future.

## A41 Exploring the Influence of Catheter Materials on Neuroinflammation and the Intracranial Microbiome

### Michael Meggyesy^1^, Yinghao Li^2^, Dipankar Biswas^1^, Gwendolyn Williams^1^, Ryan P. Lee^1^, Enoch Kim^3^, Jun Hua^2,4,5^, Horst A. von Recum^6^, Jeffrey R Capadona^6^, Mark G. Luciano^1^

#### ^1^Department of Neurosurgery, Johns Hopkins University School of Medicine, Baltimore, MD, 21205, USA; ^2^F.M. Kirby Research Center for Functional Brain Imaging, Kennedy Krieger Institute, Baltimore, MD, USA; ^3^Nova Southeastern University Dr. Kiran C. Patel College of Allopathic Medicine, Fort Lauderdale, FL, USA; ^4^Department of Biomedical Engineering, Johns Hopkins, Baltimore, MD, USA; ^5^Neurosection, Division of MRI Research, Department of Radiology, Johns Hopkins, Baltimore, MD, USA; ^6^Department of Biomedical Engineering, Case Western Reserve University, Cleveland, OH, USA

##### **Correspondence**: Mark Luciano, markluciano@jhu.edu

*Fluids and Barriers of the CNS* 2023, **20(Suppl 2)**: A41

**Introduction**: Ventricular catheter obstruction remains one of the most frequent causes of shunt failure. Standard silicone or antibiotic infused (AI) – for infection reduction - catheter materials are commonly used, with unknown impact on obstruction mechanisms. Recent discovery of the brain’s microbiome using intracranial 16sRNA detection allows evaluation of another potential modulator of the neuroinflammatory reaction. In this study we investigated the level of neuroinflammation in response to standard silicone and AI catheters using MRI and 16sRNA analysis.

**Methods**: A total of 29 rodents were used in this study. Eight rodents in each group were implanted with either silicone or AI catheter fragments. Six rodents received sham-surgery where the catheter material was immediately removed. Seven controls without surgery were used. Two AI, two silicone-implanted and one control subject were scanned on an 11.7 Tesla MRI with intravenous ferumoxytol contrast agent to stain activated macrophages at 1-, 4-, 8-, and 16-weeks post implantation. Analysis was performed on T2, flair and SWI sequences. Six rodents per group were perfused and sacrificed at 1- and 4-weeks post implantation for 16sRNA analysis.

**Results**: Peri-implantation edema on MRI was similar in both implanted groups and resolved by week 4. AI implanted rodents had less macrophage activity on MRI than the silicone implant group after week 4. 16sRNA analysis showed an increase in the microbial class of Bacterioides (+ 50% in dominance), and a decrease of Beta- and Gammaproteobacteria (− 18.7 and − 17%) over the post-implant time. Negative controls and sham-surgery showed an inverse pattern, with a decrease of Bacterioides (-18%) and increase of Gammaproteobacteria (+ 22%).

**Conclusions**: In this pilot antibiotic impregnation demonstrated overall decreased levels of neuroinflammation, as indicated by macrophage activity, and an alteration of the brain microbiome. The relationship of these changes to incidence of catheter obstruction and infection rates is currently unknown.

## A42 Acute hydrocephalus and cortical neurodegeneration in Dnah5 knockout mice: exploring mechanisms and pathogenesis

### Koichiro Sakamoto^1^, Kaito Kawamura^1,2^, Madoka Nakajima^1^, Masakazu Miyajima^3^, Chihiro Akiba.^3^, Kou Horikoshi^1,2^, Ryo Miyahara^1^, Chihiro Kamohara^1^, Ikuko Ogino^1^, Shinya Yamada^1,4^, Kostadin Karagiozov^1^, Akihide Kondo^1^

#### ^1^Department of Neurosurgery, Juntendo University School of Medicine, Tokyo, Japan; ^2^Department of Neurosurgery, Saiseikai Kawaguchi General Hospital, Saitama, Japan; ^3^Department of Neurosurgery, Juntendo Koto Geriatric Medical Center, Tokyo, Japan; ^4^Department of Neurosurgery, Kugayama Hospital, Tokyo, Japan

##### **Correspondence**: Koichiro Sakamoto, k-sakamo@juntendo.ac.jp

*Fluids and Barriers of the CNS* 2023, **20(Suppl 2)**: A42

**Introduction**: Hydrocephalus has been reported to occur due to dysfunction of ependymal cilia, as cilia are thought to contribute to the maintenance of cerebrospinal fluid circulation and ventricular morphology. Mice lacking Dynein axonemal heavy chain 5 (Dnah5), which is expressed in the outer arm of the cilia, develop cerebral aqueduct occlusion and acute hydrocephalus early after birth, leading to death within one month. We have created a Dnah5 knockout (KO) mice, observed the ventricles and cerebral parenchyma, and aimed to elucidate the mechanism of congenital hydrocephalus based on the morphological changes and cellular differences.

**Methods**: Microarray analysis was performed on mouse brain tissue from 3-day-old mice before cerebral aqueduct occlusion to extract Target genes. We also performed pathological and histological evaluation of Dnah5 KO mice 　brain and analyzed ciliary movement.

**Results**: We found that the number of neurons in the selected cortical area of the Dnah5 KO mouse brain was reduced by approximately 25%. Microarray analysis showed that mutations in the NeuroD6 gene pathway, which is involved in neural development, were strongly reduced. The expression of the dync1h1 gene encoding dynein, a motor protein involved in ciliary motility, was also found to be downregulated. Cytoplasmic dynein plays an important role in axon elongation and neuronal migration and development, but Dnah5KO mice showed significant increase in neuronal development defects. Fluorescent immunostaining showed the decrease of dync1h1 expression and the reduction of mature neurons.

**Conclusions**: The Dnah5 gene is involved not only in ciliary movement but also in the formation of brain parenchyma. Dnah5 KO mice are considered to have abnormalities in neuronal cells, with reduced neuronal density and enlarged ventricles from birth.

## A43 In-Vitro Evaluation of Shunt Valve Flow Dynamics

### Gwendolyn Williams^1^, Michael Meggyesy^1^, Kenae Thompson^2^, Enoch Kim^3^, Dipankar Biswas^1^, Mark Luciano^1^

#### ^1^Department of Neurosurgery, Johns Hopkins University School of Medicine, Baltimore, MD, 21287, USA; ^2^Krieger School of Arts and Sciences, Johns Hopkins University, Baltimore, MD, 21287, USA; ^3^Nova Southeastern University Dr. Kiran C. Patel College of Allopathic Medicine, Fort Lauderdale, FL, 33314, USA

##### **Correspondence**: Mark Luciano, markluciano@jh.edu

*Fluids and Barriers of the CNS* 2023, **20(Suppl 2)**: A43

**Introduction**: Hydrocephalus leads to over 30,000 shunt implantations in the US annually in both pediatric and adult populations. When valves are implanted in series, the expectation being that the opening pressures of each valve can be combined in an additive fashion to determine the overall equivalent resistance of the valves in series, though this hasn’t been confirmed experimentally. Here, we developed an *in-vitro* shunt system to investigate flow dynamics of commonly implanted valves in isolation and in series.

**Methods**: A gravity-driven *in-vitro* flow system at 37°C with a simulated ICP of 22 cmH_2_O and 60 cmH_2_O from valve to peritoneal cavity was built. Differential pressure (DPV) and gravitational (GV) valves were tested in isolation and series at various settings. The relationship between flow rate and the pressure drop across a valve is expressed with a valve coefficient (K_v_). Results of isolated valve trials were used to calculated K_v_ for each valve, which were then used to calculate combined valve coefficients (K_vv_) to predict flowrate of valves in series. Flowrate predictions were compared to experimental results.

**Results**: In isolation, DP and GV valves had low intra- and inter-valve variability (p > 0.05). Valves in series had highly variable flowrates across trials and sets of valves in both supine and upright positions (p < 0.05). Using calculated K_vv_ to predict flowrates of valves in series, the average percent error was 15 ± 7% and 23 ± 18% in the supine and upright positions, respectively.

**Conclusions**: These results indicate low flowrate variability of isolated valves, but high variability of valves placed in series.

The unpredictable nature of valves in series seen here makes the theoretical models of additive opening pressures and K_vv_ seem insufficient. These findings underscore the difficulties faced by physicians in determination of optimal valve settings for shunted patients.

## A44 Quantitative Analysis of Flow and Pressure Changes Through Explanted Biobank Ventricular Catheters

### Ahmad Faryami^1^, Adam Menkara^1^, Shaheer Ajaz^1^, Christopher Roberts^2^, Carolyn A. Harris^2^

#### ^1^Biomedical Engineering, Wayne State University, Detroit, Michigan,48202 USA; ^2^Chemical Engineering and Material Science, Wayne State University, Detroit, Michigan,48202 USA

##### **Correspondence**: Ahmad Faryami: gw7895@wayne.edu

*Fluids and Barriers of the CNS* 2023, **20(Suppl 2)**: A44

**Introduction**: Although shunts are still the neurological devices with the highest failure rate, there are a limited number of methods that yield quick, quantifiable measures of catheter obstruction. A significant degree of inter-patient variation is observed in the frequency of shunt replacement surgeries. And intrinsic and extrinsic variables that impact the degree of shunt obstruction are unknown. The proposed setup is a simple and rapid method to measure the degree of obstruction in an explanted ventricular catheter.

**Methods**: The setup is composed of two main components: Fluidic circuit and data acquisition. The data acquisition unit is controlled by a microcontroller and a custom-built program. The flow of distilled water or saline through the catheter is measured with pressure and flow sensors in real-time. The slope of the linear function indicates the resistance value for each test run. The obstruction of the catheters could also be defined as the time elapsed to run each experiment. The degree of obstruction of 80 explanted catheters was quantified and correlations between patient variables and measured resistance were investigated using machine learning. A predictive model was developed to estimate the relative resistance of the explanted catheters.

**Results**: strong correlation was also observed between semi-quantitative analysis of lateral hole obstructions and relative resistance. A higher relative resistance was measured in catheters removed from neonatal patients compared to pediatric and adult patients. The difference in the relative resistance of catheters received from different centers was also statistically significant.

**Conclusions**: this study investigated the variation in the degree of shunt obstructions between explanted catheters in Harris Lab biobank. A significant degree of variation was observed among explanted catheters such that a considerable portion of explanted catheters were comparable to relative resistance of unused catheters. However, a few catheters were also completely obstructed, and no flow was observed.

## A45 A Flow Optimized Ventricular Catheter; Flow Redistribution, Shear Reduction, and Reduced Astrocytic Activation

### Ahmad Faryami^1^, Nathan G. Tappen^2^, Chris Roberts^2^, Carolyn A. Harris^2^

#### ^1^Biomedical Engineering, Wayne State University, Detroit, Michigan,48202 USA; ^2^Chemical Engineering and Material Science, Wayne State University, Detroit, Michigan,48202 USA

##### **Correspondence**: Ahmad Faryami, GW7895@wayne.edu

*Fluids and Barriers of the CNS* 2023, **20(Suppl 2)**: A45

**Introduction**: Despite 60 years of research and development, shunts are still the neurological devices with the highest failure rate: more than 50% of the shunts fail only after 2 years of implantation. Our recent analysis of astrocyte cytokine secretion under shear stress revealed a statistically significant increase in pro-inflammatory IL-6 cytokine secretion. These data have pointed us toward attempts to reduce shear with a new shunt design.

**Methods**: A catheter optimized to minimize shear was designed and evaluated *in-silico*. A batch of 10 PDMS catheters were produced, and the surface properties of the manufactured catheters were investigated using scanning electron microscopy. Protein adsorption was investigated in our *in-vitro* setup under physiologic flow rates.

**Results**: The shear-optimized catheters demonstrated significantly more uniform distribution of flow through all the lateral holes *in silico*. Higher protein adsorption was observed in the proximity of the distal holes of catheters with the standard architecture compared to the shear-optimized catheter.

**Conclusions**: A truly optimized catheter is the implementation of various modifications to current catheters. This is a brief description of catheter profile with future computational optimization.

## A46 Vascular Risk Factors, Causes of Death and 10-year Mortality in idiopathic normal pressure hydrocephalus (INPH)

### Sanna A. Eklund^1^, Hanna Israelsson^2^, Mattias Brunström^3^, Karin Forsberg^1^, Jan Malm^1^

#### ^1^Department of Clinical Science, Neurosciences, Umeå University, Umeå, Sweden; ^2^Department of Health, Medicine and Caring Sciences, Linköping University, Linköping, Sweden; ^3^Department of Public Health and Clinical Medicine, Umeå University, Umeå, Sweden

##### **Correspondence**: Sanna Andrea Eklund, sanna.eklund@umu.se

*Fluids and Barriers of the CNS* 2023, **20(Suppl 2)**: A46

**Introduction**: Idiopathic Normal Pressure Hydrocephalus (iNPH) have both higher mortality and higher prevalence of vascular risk factors (VRFs) compared to the population. INPH mortality studies deviate regarding which VRF that are most important for avoiding premature death. The aim of this study was to investigate 10-year mortality, cause of death and association between individual VRFs and mortality in iNPH.

**Methods**: This prospective cohort study included 176 iNPH undergoing CSF shunt surgery, and 368 age- and sex-matched controls. At inclusion, participants were medically examined, had blood analysed for lipids, creatinine and glucose and answered a questionnaire. The VRFs investigated were smoking, diabetes, body mass index (BMI), blood pressure (BP), hyperlipidemia, kidney function, atrial fibrillation and cerebro- and cardiovascular disease. Survival after surgery was followed for 10.3±0.84 years. Causes of death were categorised into disease groups using ICD-10. COX-regression and independent sample t-test were used for analysis.

**Results**: iNPH had an increased risk of death compared to controls (hazard ratio (HR)  = 2.5, 95% CI 1.86–3.36; p < 0.001). After 10 years, 50% (n = 88) of INPH patients and 24% (n = 88) of the controls were dead (p-value < 0.001). Systolic BP (HR = 0.985 95% CI 0.972–0.997, p = 0.018), atrial fibrillation (HR = 2.652, 95% CI 1.506–4.872, p < 0.001) and creatinine (HR = 1.018, 95% CI1.010–1.027, p < 0.001) were independently associated with mortality for iNPH. The most common cause of death in iNPH were diseases of the circulatory system (14%) and for controls neoplasm (7%) and diseases of the circulatory system (7%). Compared to controls, cerebrovascular disease, dementia, and falls were overrepresented causes of death in iNPH (p < 0.05).

**Conclusions**: INPH have a higher mortality than a matched population and the risk of lethal falls is imminent. Overrepresented causes of death in INPH are diseases of the circulatory system, cerebrovascular diseases and dementia, highlighting why vascular risk factors should be systematically assessed and treated in iNPH.

## A47 Smartphone monitoring of multidomain gait parameters to facilitate remote monitoring of gait in Normal Pressure Hydrocephalus

### Aida Kamalian, Abhay Moghekar

#### ^1^Neurology Department, Johns Hopkins University School of Medicine, Baltimore, MD, 21224, USA

##### **Correspondence**: Abhay Moghekar, am@jh.edu

*Fluids and Barriers of the CNS* 2023, **20(Suppl 2)**: A47

**Introduction**: Gait dysfunction is a classic symptom of Normal Pressure Hydrocephalus (NPH). While current instrumented measures have focused on gait speed, we aimed to establish the reliability of smartphone monitoring of gait in NPH patients in three domains of gait using CDC recommended measures of gait function.

**Methods**: We recruited 15 (mean age 73.5 ± 7.1) probable NPH patients undergoing large volume lumbar puncture (LVLP) or extended lumbar drainage (ELD) at Johns Hopkins to determine their eligibility for ventriculoperitoneal shunt surgery. We recorded their gait tests before and after the procedure using Mon4t application concurrent to the physiotherapists’ (PT) assessments. The gait tests covered all three domains: Speed (Timed-Up-Go), balance (30-second sit-to-stand and 4-stage-balance test), and endurance (2-Minute-Walk Test). We used Pearson correlation and ANOVA tests for data analysis.

**Results**: Results obtained with Mon4t app showed a significantly positive correlation with the PT results both in the absolute measures and the magnitude of change before and after procedure in each patient. Correlation coefficient for different gait tests included: Timed-Up-Go = 0.96, change in Timed-Up-Go = 0.87, 30-second sit-to-stand = 0.97, change in 30-second sit-to-stand = 0.73, 2-Minute-Walk test = 0.99 in ELD and 0.96 in LVLP settings, change in 2-Minute-Walk test = 0.8 (P-value < 0.05). Moreover, anteroposterior sway in the first stage of the balance test as measured by Mon4t increased in patients who were able to successfully finish more stages in the balance test (ANOVA p-value = 0.053).

**Conclusions**: The results of this pilot study suggest that smartphone monitoring of gait using the Mon4t Clinic application is a reliable and valid tool for assessing gait in NPH. The strong correlation between the results indicates that the app can be used as an adjunct to clinical assessment in the management of NPH if validated in an ongoing larger cohort.

## A48 Endoscopic third ventriculostomy versus Ventriculoperitoneal shunt in the treatment of normal pressure hydrocephalus: Preliminary results of a randomized approach

### Loay Shoubash, Ina Lange, Maximilian Schilling, Sascha Marx, Henry H. W. Schroeder

#### ^1^Department of Neurosurgery, University Medicine of Greifswald, Greifswald, Germany

##### **Correspondence**: Loay Shoubash, E-mail: loay.shoubash@uni-greifswald.de

*Fluids and Barriers of the CNS* 2023, **20(Suppl 2)**: A48

**Introduction**: Diagnosis and selecting the best treatment option for idiopathic normal pressure hydrocephalus (iNPH) is challenging. Ventriculoperitoneal shunt (VPS) and endoscopic third ventriculostomy (ETV) are part of the surgical options. Very little evidence is available for ETV outcomes. We report a randomized study to explore both treatment outcomes.

**Methodology**: Prospective randomized, parallel, open-label trial included patients over 60 years with iNPH, and with positive responses to the tap test (TT) or lumbar drain trial (LDT) from 2016 to 2022. The functional outcomes were assessed according to the Kiefer index (KI). Standard gait exam and mini-mental state exam (MMSE) were also used. The minimum follow-up time was set at 2 years. ETV was performed with a rigid endoscope with a 30° lens (KARL STORZ SE & Co. KG), and VPS was performed with a fixed-pressure valve (ProGAV 1.0/2.0 Christoph Miethke GmbH & Co. KG).

**Results**: 31 Patients were included, and the randomization elicited 16 ETV Group and 15 VPS Shunt. Nine patients from the ETV Group received VP Shunt due to failure of improvement after the procedure.

The outcome in VPS Group showed statistically significant improvement compared to ETV within the first year. At 2 years follow-up (still not completed by all patients), there was no significant difference due to secondary worsening or return to baseline of VPS group (35%). Interestingly, the patients who received both treatments had the best outcome. This study is currently being stopped.

**Conclusion**: VPS was significantly better in treatment than ETV, especially in the early phase after treatment. However, some patients treated with VPS showed a secondary worsening in outcome after a long follow-up. This could be part of the progressive nature of the disease.

## A49 Cerebrospinal fluid and intracranial blood rapid interactions in normal pressure hydrocephalus

### Cyrille Capel^1,2^, Kimi Owashi^2^, Serge Metanbou^1^, Alexis Joannides^3,^ Zofia Czosnyka^3^, Marek Czosnyka^3^, Peter Smielewski^3^, Olivier Balédent^1,2^

#### ^1^University hospital, Amiens, France.; ^2^CHIMERE UR7516, Jules Verne University , Amiens, France; ^3^Division of Neurosurgery, Department of Clinical Neurosciences, University of Cambridge, Cambridge, UK

##### **Correspondence**: Olivier Balédent, olivier.baledent@chu-amiens.fr

*Fluids and Barriers of the CNS* 2023, **20(Suppl 2)**: A49

**Study supported by**: Revert Project, Interreg, France (Channel Manche) England, funded by European Regional Development Fund.

**Introduction**: Changes in intracranial pressure amplitude within the cardiac cycle are dependent on cerebral arteriovenous volume change and intracranial compliance. Cerebrospinal fluid (CSF) oscillation from the cranial compartment to the spinal compartment is a factor determining intracranial compliance. Our objective was to quantify how CSF oscillations apply to balance intracranial blood volume changes during cardiac cycle in suspected hydrocephalus patients.

**Method**: Patients with suspected normal pressure hydrocephalus (NPH) undergoing an infusion test and MRI were included. Based on Rout, patients were divided into an NPH+ group with Rout > 12 mmHg/mL/min, and an NPH- group with Rout < 12 mmHg/mL/min. Cerebral arterial blood flows, cerebral venous blood flows and CSF oscillations in the cervical spaces during cardiac cycle were quantified using phase-contrast MRI. From these dynamic curves, tailored software automatically calculated arteriovenous volume change (SV_AV_) and intracranial CSF volume change (SV_CSF_) during cardiac cycle.

**Results**: We included a total of 23 patients (73±8 years), with 13 patients in the NPH+ group and 10 in the NPH- group. SV_AV_ values were heterogeneous and not significantly different between the groups (SV_AV_ = 0.71+/−0.17 in NPH+ and 0.82+/−0.29 NPH-). Similar results were found for the SV_CSF_ (SV_CSF_ = 0.42+/−0.20 in NPH+ and 0.53+/−0.18 NPH-). We found a significant correlation (R2 = 0,76; p = 0,001) between SV_AV_ and SV_CSF_ in NPH- patients whereas this correlation was not significant (R2 = 0,17, p = 0,16) in NPH+ patients.

**Conclusions**: Intracranial compliance is a complex concept resulting from dynamic CSF and tissues compartments volume change. Two mechanisms co-exist: one to compensate large but slow intracranial volume change (infusion test) and one to compensate small but rapid intracranial volume change (systolic arterial flow, as revealed by PC-MRI). Our preliminary results show that, during the short time of cardiac cycle, the mobile CSF, which is part of rapid intracranial compliance, is altered in suspected NPH with elevated Rout.

## A50 Characteristics of human meningeal lymphatic vessels

### César Luis Vera Quesada^1,2^, Shreyas Balachandra Rao^3^, Reidun Torp^3^, Per Kristian Eide^1,2^

#### ^1^Department of Neurosurgery, Oslo University Hospital-Rikshospitalet, Oslo, Norway; ^2^Institute of Clinical Medicine, Faculty of Medicine, University of Oslo, Oslo, Norway; ^3^Division of Anatomy, Department of Molecular Medicine, Institute of Basic Medical Sciences, University of Oslo, Oslo, Norway

##### **Correspondence**: Per Kristian Eide, p.k.eide@medisin.uio.no

*Fluids and Barriers of the CNS* 2023, **20(Suppl 2)**: A50

**Introduction**: In recent years, renewed interest in meningeal lymphatic vessels and their role in health and disease has led to numerous studies, primarily studies in animal models and from human autopsy material. Meningeal lymphatic vessels are found mainly along the walls of dural sinus veins. The present work addressed i) methodological aspects of visualization of lymphatic vessels in the dura of humans, and ii) characteristics of meningeal lymphatic vessels in various locations.

**Methods**: Dura biopsies were obtained during neurosurgical procedures in varying locations, including shunt surgery for idiopathic normal pressure hydrocephalus (iNPH), and craniotomies for meningiomas, aneurysms, and other pathologies. The dura mater samples were examined through immunohistochemistry using the lymphatic cell markers lymphatic vessel endothelial hyaluronan receptor 1 (LYVE-1), podoplanin, (PDPN) and vascular endothelial growth factor receptor 3 (VEGFr3).

**Results**: We have characterized three types of meningeal lymphatic vessels in human dura: 1) Lymphatic vessels in near proximity to blood vessels. 2) Lymphatic vessels without nearby blood vessels. 3) Clusters of lymphatic structures. Generally, lymphatic vessels were observed most frequently towards the arachnoid membrane. Furthermore, positive immunoreactive lymphatic endothelial cells were found in the dura mater at the temporal skull base, frontal convexity, posterior fossa and craniocervical junction far from dural sinus veins.

**Conclusions**: We conclude that visualization of meningeal lymphatic vessels in live humans seems to be highly sensitive to the tissue processing method. Our observations also show that meningeal lymphatic vessels in humans can be found spread throughout the dura mater and not solely lining the dural sinuses. Further studies need to explore how meningeal lymphatic vessels are affected in disease.

## A51 Urinary Incontinence and Normal Pressure Hydrocephalus: Some clues for what is still uncertain

### Julián Azuero^1^, Alexandra Ramos^2^, Diana Valentina Garrido^2^, Isabella Mejía^3^, Diego F. Gómez^3^, Juan A. Mejía^3^, Mauricio Plata^1^, Fernando Hakim^3^, Juan F. Ramón^3^

#### ^1^Department of Urology, Hospital Universitario Fundación Santa Fe de Bogotá, Colombia; ^2^Faculty of Medicine, Universidad de los Andes, Bogotá, Colombia; ^3^Department of Neurosurgery, Hospital Universitario Fundación Santa Fe de Bogotá, Colombia

##### **Correspondence**: Juan F. Ramón, juanfernandoramon@yahoo.com

*Fluids and Barriers of the CNS* 2023, **20(Suppl 2)**: A51

**Introduction**: Urinary incontinence is part of the classical symptom triad in normal pressure hydrocephalus (NPH), and greatly affects patient and caretaker quality of life. 45–95% of patients with NPH report urinary symptoms. However, data for characterization of urinary incontinence in NPH, improvement after shunt surgery and associations with demographic and clinical factors is scarce. Research in this area is important due to the prevalence of other causes of urinary incontinence in older adults, which may impact urinary symptoms at presentation and after surgical management.

**Methods**: This study aimed to evaluate the prevalence of urinary incontinence in patients with NPH, as well as symptom improvement after shunt surgery, and associations with demographic and clinical factors. We reviewed the medical records of all patients diagnosed with NPH at our institution between 2016 and 2022, where the International Consultation on Incontinence Questionnaire-Urinary Incontinence Short Form questionnaire (ICIQ-UI-SF) was applied at diagnosis and post-operatively.

**Results**: We found 117 patients diagnosed with NPH; 75 underwent shunt surgery. The average age was 78.7 years, and 69.2% were male. 82% had urinary symptoms at diagnosis, with a mean ICIQ score of 10.6/21 (SD 6.5). 46.9% reported only urge incontinence; 5.2%, only stress incontinence; and 25% reported mixed symptoms. For 83.6% of patients, ICIQ score improved in postoperative follow-up; however, 10.8% of these patients deteriorated to baseline in further follow-up. Symptoms of urge incontinence (OR = 7.2, p = 0.0214), body mass index > 25 (OR = 14.4, p = 0.0036), and age < 80 years (OR = 1.28, p = 0.7510) were associated with increased chance of post-operative improvement.

**Conclusion**: We found a post-operative improvement rate similar to that reported in literature. 30% of patients with NPH and urinary symptoms were either mixed or stress incontinence, highlighting the importance of a multidisciplinary approach for management of these symptoms. We found few statistically significant associations, signaling the need for continued research in this field with larger, prospective studies.

## A52 Multi-Domain Assessment of Gait at Baseline Visit in patients with Idiopathic Normal Pressure Hydrocephalus (iNPH)

### Megha Patel, Abhay Moghekar

#### ^1^Department of Neurology, Johns Hopkins University School of Medicine, Baltimore, MD, 21231, USA

##### **Correspondence**: Abhay Moghekar, am@jhmi.edu

*Fluids and Barriers of the CNS* 2023, **20(Suppl 2)**: A52

**Introduction**: Though gait dysfunction in iNPH has been investigated intensively, most studies have focused on gait speed, a few studies on components of balance, and none on endurance. While cognitive assessments are often analyzed in terms of the domains affected, similar domain-based assessment of gait in iNPH has been lacking.

**Methods**: Probable iNPH patients (n = 322) referred to the Center for CSF Disorders for assessment of shunt candidacy underwent multi-domain gait evaluation at baseline visit. Speed was assessed using the Timed Up and Go (TUG) test and the 10-Meter Walk (10MW) test. Static and dynamic balance was assessed using the 4 Stage Balance (4SB) test and the 30-Second Sit to Stand (30STS) test. Endurance was assessed using the 2-Minute Walk (2MW) test. The performance on each of these tests was compared to age and sex adjusted norms.

**Results**: There were 186 males and 136 females with a mean age of 74.4 (SD: 8.46) years. The most affected domain was balance, with 95% of patients testing abnormally on the 30STS test compared to 92% of patients when tested using the 4SB test. The TUG test was abnormal in 92 % while the more widely used 10MW was abnormal only in 64%. Endurance was abnormal in 68%. 238 patients had all three domains tested. Of these, 70% performed abnormally in at least one of the tests in each domain.

**Conclusions**: Gait speed, balance and endurance of gait are impacted differentially in iNPH. Therefore, it is important to evaluate each domain independently. Assessment of gait speed just by the 10MW test is insufficient to capture the burden of gait dysfunction in iNPH. More emphasis needs to be placed on balance measures in iNPH assessment.

## A53 Audiovestibular Symptoms in Shunted Idiopathic Normal Pressure Hydrocephalus

### Enoch Kim^1,2^, Michael Meggyesy^1^, Ryan P. Lee^1^, Christina Ritter^1^, Sevil Yasar^3^, Mark G. Luciano^1^

#### ^1^Department of Neurosurgery, Johns Hopkins University School of Medicine, Baltimore, MD, 21205, USA; ^2^Nova Southeastern University Dr. Kiran C. Patel College of Allopathic Medicine, Fort Lauderdale, FL, 33328, USA; ^3^Division of Geriatric Medicine & Gerontology and Department of Neurology, Johns Hopkins University School of Medicine, Baltimore, MD, 21205, USA

##### **Correspondence**: Mark G. Luciano, markluciano@jhu.com

*Fluids and Barriers of the CNS* 2023, **20(Suppl 2)**: A53

**Introduction**: Patients with idiopathic normal pressure hydrocephalus (iNPH) can experience complications like headache and infection after ventriculoperitoneal (VP) shunt placement. However, atypical symptoms including tinnitus, vertigo, hearing loss (HL), and aural fullness can be significant debilitating symptoms and may be underrecognized and underreported. In this study, our main objective was to characterize the frequency, severity, and duration of audiovestibular (AV) symptoms in iNPH patients following VP shunt placement.

**Methods**: A retrospective chart review of iNPH patients who underwent VP shunt placement at the Johns Hopkins University, Department of Neurosurgery between June 2016 and December 2022 was conducted. Pre- and postoperative audiograms, when available, were analyzed.

**Results**: Of 390 patients with iNPH, 28 (7.2%) reported AV symptoms after surgery. Median +/− SD age was 71.5 +/− 7.2 years. Subjective HL (18, 64%), vertigo (14, 50%), tinnitus (11, 39%), and aural fullness (3, 11%) were noted postoperatively. Only 9 patients (32%) experienced resolution of at least one postoperative symptom, namely tinnitus, vertigo, or dizziness. Spontaneous resolution was noted in 2 patients. Tinnitus (1, 14%) or dizziness (3, 43%) resolved with higher shunt settings, vertigo (1, 14%) or dizziness (2, 29%), with lower shunt settings. Postoperative audiograms were available for 5 of 18 patients reporting HL. In the postoperative period, hearing thresholds increased at frequencies ranging between 250 and 800 Hz, reaching statistical significance at 1000 and 2000 Hz for air conduction. The average rate of hearing loss for all frequencies was 5.23 dB per year, nearly four-fold greater than published rates of HL.

**Conclusions**: VP shunt placement in iNPH patients can lead to short- and long-term AV complications that may be insidious and more often do not resolve. Of those who reported symptom resolution, most did after shunt setting adjustments. Prompt recognition and appropriate adjustments may increase recovery rate.

## A54 AQP4 levels in CSF correlate with clinical severity in iNPH patients. A preliminary report

### Enrico Belgrado^1^, Yan Tereshko^2^, Adriana Cifù^4,5^, Martina Fabris^4,5^, Daniele Piccolo^3^, Mariarosaria Valente^2^, Francesco Tuniz^3^

#### ^1^Neurology Department, ASUFC, S.M della Misericordia, Udine Italy; ^2^ Neurologic Clinic, University of Udine, Italy; ^3^Neurosurgery Department, ASUFC, S.M della Misericordia, Udine Italy; ^4^Department of Laboratory Medicine, ASUFC, S.M della Misericordia, Udine, Italy; ^5^Institute of Clinical Pathology, DAME, University of Udine, Italy.

##### **Correspondence**: Fransesco Tuniz, tuniz.francesco@gmail.com

*Fluids and Barriers of the CNS* 2023, **20(Suppl 2)**: A54

**Introduction**: AQP4 (Aquaporin-4) is involved in the regulation of brain water homeostasis and in the function of the glymphatic system. In iNPH (idiopathic Normal Pressure Hydrocephalus), there is an altered parenchymal expression of AQP4, contributing to glymphatic dysfunction. This study aimed to test AQP4 and AQP1 (Aquaporin-1) levels in the Cerebro-Spinal Fluid (CSF) of iNPH, NOT-NPH, and controls to evaluate their role as a diagnostic tool and their correlation with clinical parameters.

**Methods**: CSF samples were collected at the time of the Tap test (TT). Patients who responded to the TT or with a R_out_ (Resistance to outflow) ≥ 12 mmHg/mL/min, underwent ventriculoperitoneal shunting (VPS); patients with a negative response to the TT and with a R_out_ < 12 mmHg/mL/min did not receive surgery (NOT-NPH group). 10 CSF samples from healthy Controls were collected from our biobank. We performed an ELISA test to measure levels of Aquaporin 1 (Human AQP-1 E-EL-H0487, Elabscience) and Aquaporin 4 (Human AQP-4 E-EL-H0490, Elabscience). Clinical parameters were collected before and after the TT. A total of 16 iNPH patients, 10 NOT-iNPH patients, and 10 controls were involved in this study.

**Results**: AQP-1 levels were higher in iNPH versus NOT-NPH and controls (489±97 vs 404±52 vs 372±54 pg/mL, p < 0.005); AQP-4 levels were significantly higher in iNPH patients when compared with controls (1.40±0.35 vs 1.16±0.09 ng/ml p = 0.07) only. In iNPH patients there is a significant correlation between AQP-4 CSF levels and mUPDRS-III (rho = 0.76, p < 0.001), TUG (TimeUp-and-Go) (rho = 0.58, p < 0.05) and an MMSE (Mini-Mental State Examination) (rho = -0.55, p = 0.05).

**Conclusion**: AQP-4 CSF levels correlate with the severity of the disease in iNPH patients and may be used as a possible surrogate marker of glymphatic dysfunction.

## A55 Does ventriculomegaly correlate with intelligence and cognition? Neuropsychological findings and profile in longstanding overt ventriculomegaly of adults (LOVA)

### Lucia Darie^1^, Marco Pitteri^2^, Olatomiwa Olukoya^1^, Linda D’Antona^1^, Lewis Thorne^1^, Ahmed Toma^1^, Laurence Watkins^1^

#### ^1^Department of Neurosurgery, The National Hospital for Neurology and Neurosurgery, University College London Hospitals, United Kingdom; ^2^Department of Neuropsychology, The National Hospital for Neurology and Neurosurgery, University College London Hospitals, United Kingdom

##### **Correspondence**: Lucia Darie, darielucia@yahoo.com

*Fluids and Barriers of the CNS * 2023, **20(Suppl 2)**: A55

**Introduction**: *Ventriculomegaly has been often associated *with cognitive impairment, nevertheless objective data defining this patient cohort is lacking, signaling a knowledge gap. With increasing availability of imaging comes a subsequent rise in the number of cases referred to hydrocephalus services. Our aim was to outline the neurocognitive findings in this patient group.

**Methods**: This is a prospective, single-center, case-series study of patients diagnosed with longstanding overt ventriculomegaly of adults (LOVA). Demographic and radiological data as well as symptoms at presentation, profession, employment and detailed neurocognitive profile was collected.

**Results**: A total of 30 both symptomatic and asymptomatic LOVA patients with a mean fronto-occipital horn ratio (FOHR) of 0.49 (SD+/−0.06) were included. The mean age was 47.9 years (SD+/−16.8). On imaging, Aqueduct stenosis was present in 18 patients (60%). 10 (33%) and 8 (27%) out of 30 patients were found to have an above average verbal intelligence quotient (IQ) and a performance IQ respectively. No significant correlation (p 0.42) was found between FOHR and IQ in t-test. Only two patients (6.7%) were unemployed. On focal testing 3 out of 7 patients with the highest IQ showed executive function impairment and slow speed of processing. The exact neurocognitive profile is yet to be completed. To date, a comparison between pre- and post-surgery could be drawn in 3 patients, with 7 additional patients expected to have imminently complete data.

**Conclusion**: A wide variability in terms of cognition was observed in patients with ventriculomegaly that could mirror the normal distribution in the general population, concluding that ventriculomegaly should not automatically be associated with cognitive dysfunction.

**Keywords**: LOVA, ventriculomegaly, neuropsychology, intelligence quotient

## A56 Reproducibility of The Timed 10-meter walking test in Normal Pressure Hydrocephalus

### Kanza Tariq, Ahmed Toma, Lewis Thorne, Laurence Watkins

#### ^1^National Hospital for Neurology and Neurosurgery, Queen Square, London, UK

##### **Correspondence**: Kanza Tariq kanza.tariq@nhs.net

*Fluids and Barriers of the CNS* 2023, **20(Suppl 2)**: A56

**Introduction**: Gait disturbance is one of the features of normal pressure hydrocephalus (NPH). The timed-10-meter-walking test (10MWT) is frequently used diagnostic and prognostic tools for gait and balance disturbances in NPH, along-side several other disorders. We aimed to demonstrate the reproducibility of the 10MWT in NPH patients.

**Methods**: 10MWT was performed with timed slow-pace and fast-pace in 67 NPH patients using both the smart-phone app ‘Watkins’ as well as with a clinical observer using a stopwatch. The patient was requested to perform each test twice and the tests were repeated after 2 weeks. Statistical analysis used SPSS (version 25.0, IBM) by paired t-test, comparing the results of the 10MWT using ‘Watkins app’ performed over 2 weeks, as well as comparing the 10MWT with the clinical observer performed over 2 weeks for individual patients. Finally the results of 10MWT using ‘Watkins app’ and a clinical observer were compared with each other using paired t-test for individual patients.

**Results**: No statistically significant difference was found between the results of timed slow-pace and fast-pace 10MWT performed by individual patients over 2 weeks using either ‘Watkins app’ (p = 0.1419) or with a clinical observer (p = 0.4512) individually. For an individual patient, comparison of results of 10MWT as performed by ‘Watkins app’ to those performed by a clinical observer with a stopwatch showed no statistically significant difference in the measure of whole number of seconds taken to cover the distance (p = 0.3910) and statistically significant difference in number of steps taken to cover the distance (p = 0.0163) with an average error of ± 1–3 steps.

**Conclusion**: 10MWT is reproducible with high accuracy in individual patients using either smart-phone app ‘Watkins’ or clinical observer with a stopwatch. These results may vary following an intervention or change in disease status.

## A57 Advances in 3D and 4D imaging of cerebrospinal fluid and AI-based diagnosis of DESH

### Shigeki Yamada^1,2^, Satoshi Ii^3^, Tomohiro Otani ^4^, Hirotaka Ito^5^, Motoki Tanikawa^1^, Chifumi Iseki^6^, Yoshiyuki Watanabe^7^, Shigeo Wada^4^, Marie Oshima^2^, Mitsuhito Mase^1^

#### ^1^Department of Neurosurgery, Nagoya City University Graduate School of Medical Science, Aichi, Japan; ^2^Interfaculty Initiative in Information Studies/Institute of Industrial Science, The University of Tokyo, Tokyo; ^3^Faculty of System Design, Tokyo Metropolitan University, Tokyo; ^4^Department of Mechanical Science and Bioengineering, Graduate School of Engineering Science, Osaka University, Osaka; ^5^Medical System Research & Development Center, FUJIFILM Corporation, Tokyo; ^6^Department of Behavioral Neurology and Cognitive Neuroscience, Tohoku University Graduate School of Medicine, Miyagi; ^7^Department of Radiology, Shiga University of Medical Science, Shiga

##### **Correspondence**: Shigeki Yamada, shigekiyamada393@gmail.com

*Fluids and Barriers of the CNS* 2023, **20(Suppl 2)**: A57

**Introduction**: Cerebrospinal fluid (CSF) moves pulsatile to smooth cerebral circulation in the closed space skull. Recent developments in magnetic resonance imaging (MRI) technology have made it possible to visualize CSF dynamics. However, it is difficult to accurately reproduce the CSF dynamics in the complicated 3D structure of ventricle and subarachnoid spaces. Therefore, we aimed to develop computational simulation model of brain aging and chronic hydrocephalus in adults. Moreover, we developed an AI model for automatic detection/diagnosis of disproportionately enlarged subarachnoid-space hydrocephalus (DESH) to detect patients with idiopathic normal pressure hydrocephalus (iNPH).

**Methods**: This study included 180 participants (42 iNPH patients and 138 healthy adults (20–80 years old). They underwent 3D T1- and T2-weighted MRI, 3D time-of-flight MRI, 4D flow MRI for CSF (venc:5 cm/s) and for the circle of Willis (venc:120 cm/s), and intravoxel incoherent motion (IVIM) MRI.

**Results**: We obtained 3D morphological information of the brain, intracranial arteries, ventricles, and subarachnoid spaces, and the pulsatile complex motion of the CSF in the whole intracranial CSF space and arterial flow in the circle of Willis using various MRI sequences in healthy adults and iNPH patients, to reproduce the 3D intracranial environment on a computer. Moreover, we successfully developed an automatic detection/diagnosis of DESH with deep learning.

**Conclusions**: 3D digital information will be integrated to predict environmental changes in the brain and cerebrospinal fluid space with aging and simulate the development of iNPH. Accurate segmentation of the regional volumes and automatic determination of DESH from 3D T1- and T2-weighted MRI can improve the precision of iNPH diagnosis.

## A58 Distinct cerebral cortical microstructural changes in idiopathic normal-pressure hydrocephalus

### Kyunghun Kang^1^, Myong Hun Hahm^2^, Uicheul Yoon^3^, Ki-Su Park^4^, Eunhee Park^5^, Sang-Woo Lee^6^, Shin Young Jeong^6^

#### ^1^Department of Neurology, School of Medicine, Kyungpook National University, Daegu, 41404, South Korea; ^2^Department of Radiology, School of Medicine, Kyungpook National University, Daegu, 41404, South Korea; ^3^Department of Biomedical Engineering, Daegu Catholic University, Gyeongsan-si, 38430, South Korea; ^4^Department of Neurosurgery, School of Medicine, Kyungpook National University, Daegu, 41404, South Korea; ^5^Department of Rehabilitation Medicine, School of Medicine, Kyungpook National University, Daegu, 41404, South Korea; ^6^Department of Nuclear Medicine, School of Medicine, Kyungpook National University, Daegu, 41404, South Korea

##### **Correspondence**: Kyunghun Kang, Kyunghun.Kang@hotmail.com

*Fluids and Barriers of the CNS* 2023, **20(Suppl 2)**: A58

**Introduction**: The aim of the study is to evaluate idiopathic normal-pressure hydrocephalus (INPH)-related cortical mean diffusivity (MD) abnormalities.

**Methods**: We investigated cortical MD utilizing surface-based diffusion tensor imaging analysis in three groups: INPH patients, Alzheimer’s disease (AD) patients, and healthy controls. Forty‐two INPH patients, 51 AD patients, and 23 healthy controls were imaged with MRI, including three-dimensional T1-weighted MR images, for automated surface-based analysis across the entire brain.

**Results**: Compared with age- and gender-matched healthy controls, INPH patients showed a statistically significant reduction in MD in the high convexity of the frontal, parietal, and occipital cortical regions. In clusters of lower MD in INPH patients, INPH patients when compared to AD and control groups, showed a statistically significant decrease in average MD values. Additionally, a significant increase in MD, mainly in the ventromedial frontal cortex, ventrolateral frontal cortex, supramarginal gyrus, and temporal cortical regions, was observed in the INPH group relative to the control group. In clusters of higher MDs in INPH patients, INPH patients, when compared to AD and control groups, showed a statistically significant increase in average MD values. In clusters of higher MD in INPH patients, AD patients, when compared to controls, showed a statistically significant increase in average MD values. The mean MD of clusters of lower MDs in INPH patients compared with healthy controls yielded an area under the curve of 0.857, differentiating INPH from AD.

**Conclusions**: A distinctive pattern of cortical MD changes was found in INPH patients. The mean MD for clusters of lower MD in INPH patients compared with healthy controls distinguishes INPH from AD with good diagnostic sensitivity and specificity. Our findings suggest microstructural changes in cortical integrity can help differentiate INPH and AD in elderly patients.

## A59 Reversibility of the Radiological Signs of Raised Intracranial Pressure Following Intraparenchymal Brain Tumour Resection

### Kanza Tariq, James Yeomans, Ahmed Toma, Laurence Watkins, Lewis Thorne

#### ^1^National Hospital for Neurology and Neurosurgery, Queen Square, London, UK

##### **Correspondence**: Kanza Tariq, kanza.tariq@nhs.net

*Fluids and Barriers of the CNS* 2023, **20(Suppl 2)**: A59

**Introduction**: Intraparenchymal brain tumours are often associated with raised intracranial pressure (ICP), which can be identified by radiological signs of raised ICP. Surgical evacuation of the tumour in most cases leads to normalisation of the ICP. In this single-centre study we aimed to examine the rate of reversibility of radiological signs of raised ICP following resection of intraparenchymal brain tumours.

**Methods**: A retrospective-observational study was performed in 42 patients who underwent excision of intraparenchymal brain tumours in the National Hospital for Neurology and Neurosurgery during 2021 and 2022. The electronic-health-records (EHRs) of the patients were evaluated. Basic demographic information (age, gender, underlying pathology) was recorded. Pre and post-operative magnetic resonance imaging (MRI) scans of the patients were studied for signs of raised ICP, including optic-nerve sheath diameter (ONSD) and pituitary thickness. Heed was paid to the time duration between surgery and the amelioration of radiological signs of raised ICP. Statistical analysis was done by SPSS (version 25.0, IBM) by paired t-test comparing the radiological features of raised ICP in pre and post-operative MRI scans of the patients.

**Results**: 33 patients demonstrated radiological features of raised ICP. 6 patients showed tortious optic nerve, optic-nerve sheath distension and empty sella. 15 patients featured tortious optic nerve and optic-nerve sheath distension. 12 patients showed optic-nerve sheath distension and empty sella. Post tumour excision 9 patients showed complete resolution of radiological features of raised ICP within 1–6 months (p < 0.0001). 22 patients showed progressive amelioration in the signs of raised ICP from 1 month post-op onwards, with tortuosity of optic-nerves being first thing to correct, followed by improvement in ONSD, followed-by optic nerve-sheath distension and lastly pituitary thickness (p < 0.0001). 2 patients showed no improvement in radiological features on account of recurrence of disease.

**Conclusion**: Radiological features of raised ICP can be reversed following excision of intraparenchymal brain tumours.

## A60 Respiratory and cardiac signal analysis of CSF dynamics in normal pressure hydrocephalus and Alzheimer’s disease

### Pragalv Karki^1^, Matthew C. Murphy^1^, Sandeep Ganji^1^, Jeffrey L. Gunter^1^, Jonathan Graff-Radford^2^, David T. Jones^3^, Hugo Botha^3^, Jeremy K. Cutsforth-Gregory^3^, Benjamin D. Elder^3-5^, Clifford R. Jack Jr^1^, John Huston III^1^, Petrice M. Cogswell^1^

#### ^1^Department of Radiology, Mayo Clinic, Rochester, MN, 55905, USA; ^2^Department of Neurology; ^3^Department of Neurologic Surgery; ^4^Department of Biomedical Engineering; ^5^Department of Orthopedics

##### **Correspondence**: Pragalv Karki, karki.pragalv@mayo.edu

*Fluids and Barriers of the CNS* 2023, **20(Suppl 2)**: A60

**Introduction**: Normal pressure hydrocephalus (NPH) is a CSF dynamics disorder, which in some patients is associated with elevated flow through the cerebral aqueduct. CSF flow is usually evaluated with a cardiac-gated 2D phase contrast (PC) acquisition through the cerebral aqueduct. This approach is limited by evaluation of a single location and does not account for respiration effects on flow. In this study we apply a real-time 2D PC acquisition at multiple locations to evaluate both the cardiac and respiratory contributions to flow in patients with NPH compared to cognitive unimpaired controls (CU) and patients with Alzheimer’s disease (AD).

**Methods**: The study included 25 participants, 6 NPH, 9 AD, and 10 CU. Imaging was performed on a 3T Philips system. A 2D real-time echo-planar imaging-based PC acquisition was performed at the foramen magnum, fourth ventricle, and cerebral aqueduct. The standard deviation of the velocity vs time curve was used as a metric of flow excursion/magnitude of flow. The cardiac and respiratory contributions to flow were estimated by the area under the curve of the power spectral density (PSD) around the cardiac and respiratory frequencies, respectively.

**Results**: Standard deviation of velocity did not show statistically significant differences between groups. Area under the curve of the PSD around the cardiac frequency was higher in AD than CU ($p\hspace{0.17em}=\hspace{0.17em}0.03$ Welch’s t-test, $p\hspace{0.17em}=\hspace{0.17em}0.04$ Wilcoxon rank sum test) at the fourth ventricle. Area under the respiratory frequency was higher in NPH than CU at the aqueduct with $p\hspace{0.17em}=\hspace{0.17em}0.02$ based on the Wilcoxon rank sum test.

**Conclusions**: The 2D phase contrast imaging provides information about respiratory and cardiac contributions to the CSF flow. Imaging of CSF flow should not be limited to the cerebral aqueduct only. Flow in other locations may differ between groups and provide further insight into the mechanism of CSF dynamics disorders.

## A61 Structural Volumetric and Periodic Table DTI Patterns in Complex Normal Pressure Hydrocephalus – Towards the Principles of a Translational Taxonomy

### Christine Lock^1^, Nicole C. H. Keong^1,2^, for the Alzheimer's Disease Neuroimaging Initiative*

#### ^1^Department of Neurosurgery, National Neuroscience Institute, 308433, Singapore; ^2^Duke-NUS Medical School, 169857, Singapore

*Data used in preparation of this article were obtained from the Alzheimer's Disease Neuroimaging Initiative (ADNI) database (adni.loni.usc.edu). As such, the investigators within the ADNI contributed to the design and implementation of ADNI and/or provided data but did not participate in analysis or writing of this report. A complete listing of ADNI investigators can be found at: http://adni.loni.usc.edu/wp-content/uploads/how_to_apply/ADNI_Acknowledgement_List.pdf

##### **Correspondence**: Nicole CH Keong, nchkeong@cantab.net

*Fluids and Barriers of the CNS* 2023, **20(Suppl 2)**: A61

**Introduction**: Diffusion tensor imaging (DTI) measures are highly dependent on technical factors and not comparable between study sites/ across groups, limiting interpretation in small cohorts, such as Normal Pressure Hydrocephalus (NPH). To address this gap, we previously proposed a novel strategy – a periodic table of DTI elements; a taxonomic framework to describe white matter tracts by their diffusivity and neural properties. In this iteration of the periodic table, we examined patterns of tissue distortion in Complex Normal Pressure Hydrocephalus (CoNPH) and validated the methodology of the periodic table against an open-access dataset of healthy control subjects to expand its accessibility to a larger community of users.

**Methods**: Structural volumetric and DTI measures for 12 patients with CoNPH with multiple comorbidities and 45 healthy controls from the ADNI database were derived using image processing pipelines on the brainlife.io cloud computing platform (Freesurfer 7.1.1, FSL and MRTrix3). Statistical analyses were performed for comparisons between cohorts. Differences in FA, MD, L1, and L2and3 between CoNPH vs. controls were then mapped according to the periodic table algorithm.

**Results**: We found widespread and significant reductions in subcortical deep grey matter structures, in comparison to healthy controls (left thalamus, left putamen, bilateral hippocampi, p = 0.007, 0.030, 0.004 and 0.028 respectively). DTI tissue signatures in the cerebral and cerebellar white matter demonstrated more potential reversibility of injury as compared to cerebral and cerebellar cortices. The use of the periodic table algorithm allowed for greater consistency in the interpretation of DTI results and aided in areas of concurrent but conflicting profiles.

**Conclusions**: Our study findings support the hypothesis that Complex NPH cohorts retain imaging features of Classic NPH. Our aim, in evolving towards controls-in-common, is to provide a prototype that could be refined and improved for an approach towards the concept of a “translational taxonomy”.

## A62 The relationship between dual-task cost and cognitive performance among patients with probable idiopathic Normal Pressure Hydrocephalus

### Elizabeth Cray^1^, Rupert Noad^2^, Samiul Muquit^1^, Samuel Jeffery^1^

#### ^1^Department of Neurosurgery, Southwest Neurosurgery Centre, University Hospital Plymouth, Plymouth, PL6 8DH;^2^Department of Neuropsychology, University Hospital Plymouth, Plymouth, PL6 8DH

##### **Correspondence**: Elizabeth Cray, elizabethcray@nhs.net

*Fluids and Barriers of the CNS* 2023, **20(Suppl 2)**: A62

**Objective**: The dual-task gait assessment is frequently used to highlight cognitive inefficiency in idiopathic Normal Pressure Hydrocephalus (iNPH). There is, however, little evidence demonstrating its utility as a clinical marker of treatment responsiveness in these patients. The objective was to investigate the relationship between dual-task cost (DTC) during gait assessment and overall cognitive performance following cerebrospinal fluid (CSF) drainage in patients with iNPH.

**Method**: Patients with probable iNPH who underwent CSF drainage with either high-volume lumbar puncture (LP) or 3-day lumbar drain (LD) between May 2020 and December 2022 were included. Prospectively collected data from 10-metre single and 10-metre dual-task gait assessments, along with tests for phonemic and semantic fluency before and after CSF drainage were analysed. The DTC, which is the difference between single and dual task performance, was calculated and expressed as a percentage. Spearman’s correlation was used to analyse the relationship between this DTC and overall cognitive performance.

**Results**: 26 patients with probable iNPH, as defined by an improvement in gait velocity following CSF drainage were included. Median age was 75 (SD±8.85) years. Before CSF drainage the mean single and dual-task times were 26.0 and 30.0 s respectively, improving to 17.0 and 25.4 s after drainage. The mean DTC before CSF drainage was 27.1% and this increased to 54.4% after drainage. In this cohort there was a significant improvement in tests of phonemic and semantic fluency (p < 0.001) following CSF drainage, however there was no correlation between improved cognitive performance and DTC.

**Conclusion**: Although walking speed during both single and dual tasks improved following CSF drainage, the dual-task cost deteriorated despite an overall improvement in cognitive performance. The dual-task gait assessment is a useful clinical marker of the effect of cognitive loading in iNPH but provides little value during supplementary testing in our diagnostic pathway.

## A63 Long-term care in older persons suspected of idiopathic normal pressure hydrocephalus (iNPH)

### Eric A Schmidt^1^, Laurent Balardy^2^, Hélène Villars^2^

#### ^1^Department of Neurosurgery, University Hospital, Toulouse, France; ^2^Department of Geriatrics, University Hospital, Toulouse, France

##### **Correspondence**: Hélène Villars, villars.h@chu-toulouse.fr

*Fluids and Barriers of the CNS* 2023, **20(Suppl 2)**: A63

**Introduction**: Older persons with clinical and imaging signs of iNPH have excess mortality and increased risk of dementia. Shunt surgery for iNPH improves gait, functional independence and seems to normalize survival.

Long-term care involves a variety of services designed to meet older person care needs, in order to live as independently and safely as possible when they can no longer perform daily living activities on their own.

We want to understand whether identification and treatment of iNPH promote functional autonomy and, consequently, independent living at home and has an impact on long-term care. In this preliminary study, we hypothesize that shunt surgery improves survival and reduces the probability of admission in long-term care facility (LTCF).

**Methods**: 100 older patients suspected of iNPH (46W/54M mean age 74.6 years) were included in our geriatric prospective cohort. Inclusion criteria were clinical signs (gait or balance disorder, cognitive impairment or urinary incontinence) and ventriculomegaly (*i.e. *Evans Index > 0.3). Multidimensional markers of aging and neurodegenerative pathologies were analyzed, including CSF outflow resistance (Rcsf) measured with lumbar infusion test. Patients with clinical signs, ventriculomegaly and Rcsf ≥ 12 mmHg/mL/min were shunted. In this geriatric cohort, we analyzed 10 year survival rate and the percentage of patient admitted to LTCF.

**Results**: 42 patients were shunted (resp. 58 non-shunted). The 10-year survival rate was 71% in the shunted group (30 patients alive) and 48% in the non-shunted group (28 patients alive). 10 years after inclusion, 29% of the shunted patients were in LTCF (12 patients) and 34% of the non-shunted patients were in LTCF (20 patients). Kaplan Meier graphs are provided.
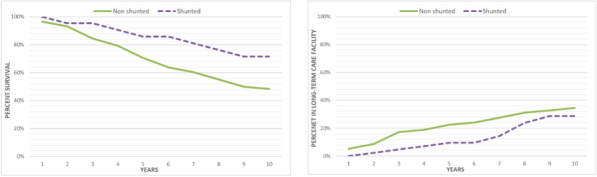


**Conclusions**: In our geriatric cohort, identification and treatment of iNPH was associated with higher 10-years survival rate and lower admission rate in LTCF. Exploring and treating older persons suspected of iNPH seems to have an impact on long-term care.

## A64 Thirty years of experience in CSF dynamics testing in adult hydrocephalus

### Zofia Czosnyka, Marek Czosnyka, Afroditi Lalou, Peter Smielewski, Alexis Joannides, John D. Pickard

#### ^1^Division of Neurosurgery, Cambridge University Hospital, Cambridge Biomedical Campus, cb20qq, Cambridge UK

##### **Correspondence**: Zofia Czosnyka, zc200@medschl.cam.ac.uk

*Fluids and Barriers of the CNS* 2023, **20(Suppl 2)**: A64

**Introduction**: Abnormal CSF dynamics is an important component of hydrocephalus. It may manifest with increased resistance to CSF outflow (Rout), abnormal elasticity, excessive ICP waves, distortion of pulse amplitude of ICP, etc.

**Methods**: Lumbar infusion test supported with computer identification of Marmarou's CSF dynamics model was designed in Poland in 1985–87 and later implemented in ICM+ software. Since 1992 over 6000 infusion studies have been performed in both shunted and non-shunted hydrocephalus patients in Cambridge.

**Results**: The infusion test is safe. There were no serious complications. Infection rate was less than 1%

1. Knowledge of compensatory parameters helps in making decisions about patients' management. Increased Rout is positively correlated with better outcome following shunting (p < 0.004; N = 352)).
2. In adults, Rout increases with age, while estimated CSF production rate decreases (p < 0.01).
3. In shunted patients, reservoir infusion study helps to objectively assess shunt function and avoid unnecessary revisions. Yearly savings are estimated to amount to £1 million in our hospital.
4. Defective CSF dynamics may overlap existing cerebrovascular disease contributing to poor clinical status. In patients with normal CSF circulation autoregulation of cerebral blood Flow (CBF) measurements estimated with transcranial Doppler was worse (p < 0.002).
5. In adults with idiopathic NPH, white matter *CBF *measurements using positron emission tomography decreases towards surface of ventricles. Also, autoregulation of CBF is worse in this region. This may illustrate transependymal route of CSF absorption and its interference with regional CBF around ventricles.
6. Even moderate rise in ICP during infusion study slightly but significantly increases heart rate variability, suggesting possible existence of 'brain baroreceptors’.

**Conclusion**: Continuing studies in CSF dynamics elucidate pathophysiology of hydrocephalus and other CSF disorders and helps in better management of patients.

**Acknowledgement**: Mr Eric Schmidt, Mrs Nicole Keong, Mr Shahan Momjian, Mrs Eva Nabbanja, Mr Brian Owler, Prof Magdalena Kasprowicz, Dr Olivier Baledent

## A65 Use of non-invasive ICP waveform monitoring in a csf collection laboratory: experience and learning points

### Carlos O. Brandão^1^, Ricardo C. Benesi^1^, Celina de Oliveira^1^, Cristiane S. Casanova^1^, Francisjane de J. Lopes^1^, Ana L. L. Diblasi^1^, Léo F. Corrêa^1^, Dalton L. D. Ferreira^1^, Maria P. F. dos Santos^1^, Maria F. G. D Dubourqc^1^, Raphael Bertani^2^

#### ^1^Neurolife, laboratório especializado no estudo do líquido cefalorraquiano (Neurolife, CSF laboratory); ^2^Cerebral Hydrodynamics Group, University of São Paulo (USP), São Paulo, SP, Brazil

##### **Correspondence**: Raphael Bertani, contato@rbertani.com

*Fluids and Barriers of the CNS* 2023, **20(Suppl 2)**: A65

**Introduction**: Intracranial compliance (IC) is the ability of the central nervous system to absorb changes in volume without increasing the intracranial pressure (ICP), it is associated with the ICP waveform (ICPw) and predicts intracranial hypertension (IH). Noninvasive technology for IC monitoring was incorporated into the laboratory routine for a trial period to evaluate synergy with cerebrospinal fluid puncture and TAP Test exams.

**Methods**: The brain4care (b4C) sensor was positioned on the patient's scalp, in the frontotemporal region. Monitoring of 330 patients was done before lumbar punctures and the TAP tests, for 5 min in the supine and standing position. Through artificial intelligence, b4c has automatically generated indicators related to intracranial compliance. The relationship between the amplitude of the subcomponents of the noninvasive ICP waveform (nICPw) P2 and P1 (P2/P1 ratio) and the time to the highest point of the wave (Time to peak - TTP) were calculated and inserted into the patient's report.

**Results**: nICPw monitoring provided results consistent with patient’s clinical symptoms and history, as well as other exams showing potential to be used as a screening method for cerebrospinal fluid puncture and adjunct to the TAP test analysis.

**Conclusions**: The monitoring of nICPw demonstrated safety, usability, and reproducibility, presenting the required characteristics for incorporation into the diagnostic routine.

## A66 In vivo performance of the Kitea intracranial pressure (ICP) System

### Sarah-Jane Guild^1,2^, Abdel-Hamed Dabbour^1,2^, Natalia Lopez^2^, Bryon Wright^2^, Robert Gallichan^2^, Dixon Leung^2^, Daniel McCormick^1,2^, Masahiro Kondo^2^, Peter Heppner^3^, Simon Malpas^1,2^

#### ^1^University of Auckland, New Zealand; ^2^Kitea Health Ltd, Auckland, New Zealand; ^3^Auckland City Hospital, New Zealand

##### **Correspondence**: Sarah-Jane Guild, s.guild@auckland.ac.nz

*Fluids and Barriers of the CNS* 2023, **20(Suppl 2)**: A66

**Introduction**: The Kitea ICP System is designed to allow long-term home monitoring of ICP in patients with hydrocephalus. A discrete micro-implant is placed within the cortex at the same time as a shunt. ICP values are obtained by passing a Wand over the implant. The system is designed to provide not only the mean ICP level but also ICP waveforms. This study investigated the stability and performance of the Kitea ICP system over a 4-week implantation period and examined the ICP waveforms obtained.

**Methods**: Six female sheep were implanted with the Kitea Implant next to ventricular shunt (Medtronic) via a burr hole in the skull. Additionally, a MEMS based pressure catheter (Millar Inc/Integra) inserted into the cortex on the opposite side of the midline allowed comparison of the Kitea ICP data to a gold-standard wired catheter. ICP measurements were made using both pressure systems three times a week for the next four weeks. At the end of 4 weeks, the sheep were anesthetized and a second Millar catheter was placed in the cortex to check the accuracy of the Kitea ICP Implant and the original Millar catheter. To assess the ability to measure a range of ICP, an intracerebroventricular canula was inserted and sterile saline infused into the ventricle to increase ICP.

**Results**: In all 6 sheep, ICP measurements with successfully made using the Kitea ICP system throughout the entire 4-week period. Comparison of the Millar and Kitea ICP data shows the Kitea System can accurately measure ICP in the conscious sheep both at baseline and in response to induced increased in ICP. Waveform analysis showed that the Kitea ICP System accurately captures features such as the cardiac and respiratory components of the ICP waveform.

**Conclusions**: We are confident that the Kitea ICP System can accurately measure ICP.

## A67 Validation of the Kitea intracranial pressure (ICP) monitoring system over 6 months in sheep

### Sang Ho Kim^1,3^, Sarah-Jane Guild^1,2^, Abdel-Hamed Dabbour^1,2^, Natalia Lopez^2^, Bryon Wright^2^, Robert Gallichan^2^, Dixon Leung^2^, Daniel McCormick^1,2^, Masahiro Kondo^2^, Mark Oliver^1,4^, Eric Ai^1,4^, Gregg Pardoe^1,4^, Simon Malpas^1,2^

#### ^1^University of Auckland, New Zealand; ^2^Kitea Health Ltd, Auckland, New Zealand; ^3^Auckland City Hospital, New Zealand; ^4^Ngapouri Research Farm Laboratory, University of Auckland, New Zealand

##### **Correspondence**: Sarah-Jane Guild, s.guild@auckland.ac.nz

*Fluids and Barriers of the CNS* 2023, **20(Suppl 2)**: A67

**Introduction**: The Kitea ICP System is designed to allow long-term home monitoring of ICP (intracranial pressure) in patients with hydrocephalus. The system is comprised of a discrete micro-implant placed within the cortex at the same time as a shunt. ICP values are obtained by passing a wand over the implant. The wand provides power wirelessly at a depth of up to 12 cm. The implant size is 2 x 3 x 20 mm. Prior to first clinical use it is important to demonstrate the ability to obtain ICP recordings in animals over an extended period.

**Methods**: 10 adult female sheep were implanted with a Kitea ICP Implant placed next to a ventricular shunt catheter (Medtronic). The sheep were allowed to recover from surgery indoors for 2 weeks before being moved to the paddock for the remainder of the 6-month study period. ICP recordings were made weekly. Three ICP recordings were collected in each sheep at each weekly session and were recorded along with the head position for each reading.

**Results**: Data were obtained on 100% of the occasions that a reading was attempted. On every occasion, values of ICP were within normal range and there was no sign of drift. Over 700 discrete measurements of ICP were made.

**Conclusions**: Along with separate histological evidence of safety and bench testing we are confident that the Kitea ICP system can be used to obtain ICP over an extended period. The next phase of this program will see the first clinical use.

## A68 Safety of the Kitea intracranial pressure (ICP) implant in the growing brain

### Sarah-Jane Guild^1,2^, Sheryl Tan^1^, Abdel-Hamed Dabbour^1,2^, Natalia Lopez^2^, Bryon Wright^2^, Robert Gallichan^2^, Dixon Leung^2^, Daniel McCormick^1,2^, Masahiro Kondo^2^, Mark Oliver^1,3^, Eric Ai^1,3^, Gregg Pardoe^1,3^, Simon Malpas^1,2^

#### ^1^University of Auckland, New Zealand; ^2^Kitea Health Ltd, Auckland, New Zealand; ^3^Ngapouri Research Farm Laboratory, University of Auckland, New Zealand

##### **Correspondence**: Sarah-Jane Guild, s.guild@auckland.ac.nz

*Fluids and Barriers of the CNS* 2023, **20(Suppl 2)**: A68

**Introduction**: The purpose of the Kitea ICP System is to allow better management of hydrocephalus in paediatric patients through home monitoring of ICP. The system is comprised of a discrete micro-implant placed within the cortex at the same time as a shunt. The external brain contacting parts of the Kitea implant are all glass. We have shown previously that the Kitea Implant does not cause significant tissue damage and does not move significantly in the adult sheep brain during implantation for 6 months. The purpose of this study was to assess the safety of the Kitea Implant in the growing brain using a lamb model.

**Methods**: Twenty 4-week-old lambs were implanted with the Kitea Implant next to a piece of shunt tubing (Medtronic). Ten of the lambs were euthanized after 1 week to assess the acute response to the implant placement and the other half were kept for 6 months. Lateral radiographs were taken to document the position of the implants after surgery and before euthanasia.

**Results**: Histological analysis of the brain tissue was used to characterize the response of the tissue around the implant and shunt. Stains for cellular and tissue structure (H&E), astrocytes (GFAP), microglia activation (IBA-1), myelinated axons, (LFB) and degenerating neurons (FJB). No signs of implant migration were seen in either the histology or analysis of the radiographs.

**Conclusions**: Using the lamb as a model of brain growth we have shown that the Kitea Implant does not migrate within the brain and has a stable tissue response.

## A69 AI-trained mixed reality head-mounted display to place ventricular catheters -preliminary data

### Martin Mersch^1^, Patrick House^2,3^, Erke Can Tellal^4^, Sirko Pelzl^4^, Uwe Kehler^1^

#### ^1^Department of Neurosurgery, Asklepios Klinik Altona, Hamburg, 22763, Germany; ^2^theBlue.ai GmbH, Hamburg, 20095, Germany; ^3^Epileptologicum Hamburg, Specialist´s Practice for Epileptology, 22299, Germany; ^4^apoQlar GmbH, Hamburg, 20095, Germany

##### **Correspondence**: Martin Mersch, Martin.Mersch@outlook.com

*Fluids and Barriers of the CNS* 2023, **20(Suppl 2)**: A69

**Introduction**: Artificial intelligence (AI) and mixed reality holographic navigation technology are currently a much-researched topic in surgery. In neurosurgery, there is little data on practical clinical application. The purpose of our study is to test the applicability of a mixed reality head-mounted display (HMD) for the placement of ventricular catheters in ventriculoperitoneal shunt surgery. The ethical committee allowed a series with 10 patients.

**Methods**: In advance, we trained an AI with 71 MRI T2 data sets to automatically recognize the ventricles. Thereafter, we used the HMD to visualize the lateral ventricles on the head of five patients with normal pressure hydrocephalus while performing ventriculoperitoneal shunt surgery. An evaluation was carried out regarding the number of ventricular punctures, the surgeon's subjective comfort and the safety gain.

**Results**: We applied this technique in 5 patients so far. One patient dropped out for a reason unrelated to the HMD. In 50 % (2 of 4) of the cases, the ventricle could be punctured the first time, in 50 % (2 of 4) the 2nd or the following times, the surgeon rated the comfort as good in 50 % (2 of 4) of the cases. An increase in safety due to the HMD was recorded in 25 % (1 of 4) of the cases, no loss of security was reported.

**Conclusions**: First results show that the HMD for ventricles imaging has a high potential for safe ventricular puncture. To date, however, the rate of first-time ventricular punctures is low. Inaccuracies, especially regarding referencing and stability during surgeries due to movements of the operating table and patient’s head, need to be improved by intraoperative real-time adjustments of preoperative imaging. However, developments already underway will take these requirements into account.

## A70 Comparison between conventional surgery and robotic endoscopic third ventriculostomy in occlusive hydrocephalus

### Thomas M. Freiman^1^, Lennard Spanehl^1^, Daniel Dubinski^1^, Florian Gessler^1^, Johannes Buchmann^2^, Sae-Yeon Won^1^

#### Departments of ^1^Neurosurgery and ^2^Child- and Adolescence Neuropsychiatry, University Medical Centre Rostock, Schillingallee 35, D-18057 Rostock, Germany

##### **Correspondence**: Thomas Freiman thomas.freiman@med.uni-rostock.de

*Fluids and Barriers of the CNS* 2023, **20(Suppl 2)**: A70

**Introduction**: Causes for occlusive hydrocephalus are congenital aqueduct stenosis, cerebellar-, skull base- or pineal tumors. Endoscopic third ventriculostomy (ETV) is considered as the primary therapy in occlusive hydrocephalus.

**Methods**: A neurosurgical robot (Robotic Surgery Assistant, ROSA) has been used for several years for stereotaxic procedures and was adapted for the endoscopic procedure. Since the selection of an ideal trajectory through the foramen of Monro towards the prepontine cistern is crucial, robotic technology may be beneficial. In this study 11 robotic procedures in the last 2 years and the previous 11 conventional ETV procedures were compared. The method of conventional and robotic procedure consisted of a preoperative three-dimensional (3D) MRI with T1 and T2 weighted images. Imaging was programmed into the robotic planning software, referencing with the head-clamp-fixed patient was achieved by a semi-robotic laser scan of the face. In the conventional group the trajectory and burr hole were identified by measurements from imaging. Patients were controlled clinical and by MRI T2 imaging straight-, three- and twelve months after surgery.

**Results**: In the robotic group the mean age was 39 years (Range 4–80), in the conventional 36 years (Range 9–73), In the age matched robotic group, only one female was identified, versus seven in the conventional group. The duration of surgery amounted to 117 min (range 63–177) in the robotic- and 79 min (Range 54–150) in the conventional group. The surgery was successful in ten of eleven patients in the robotic group- and in all patients in the conventional group. One patient from the robotic group required a ventriculo-peritoneal shunt after two months, one patient in the conventional group had a thalamic lesion related to the endoscope.

**Conclusion**: In conclusion, the robotic procedure takes more time, however it results in an optimal trajectory avoiding friction in the foramen of Monro and lesions in the surrounding ventricular structures.

## A71 Unravelling mechanisms driving ventricular catheter obstruction - a multicenter shunt biobank approach

### Hariharan Prashant^1^, Carolyn Harris^1,2^

#### ^1^Department of Biomedical Engineering, Wayne State University, Detroit, MI, United States; ^2^Department of Chemical Engineering and Materials Science, Wayne State University, Detroit, MI, United States;

##### **Correspondence**: Prashant Hariharan, fj1852@wayne.edu

*Fluids and Barriers of the CNS* 2023, **20(Suppl 2)**: A71

**Abstract**: Hydrocephalus patients have a severely diminished quality of life because of the failure and ineffectiveness of current treatments, which involve diversion of cerebrospinal fluid (CSF) with shunts. To investigate factors that affect shunt failure, we established a multicenter biobank. Our project aims to create a comprehensive dataset that can help us understand the mechanisms driving shunt obstruction and the factors that may help predict the performance of a shunt in a specific patient.

**Methods**: To date our biobank has recruited 6 centers across the United States and collected over 500 failed ventricular catheters (VCs) with relevant clinical data. Each VCs was analyzed using bright field and confocal microscopy, immunofluorescent labelling, histology, and immunohistochemistry.

**Results**: Of the VCs determined intraoperatively to be obstructed by surgeons, 36% were found to be unobstructed on microscopic analysis. Of the obstructed VCs, 61.5% had tissue aggregates occluding at least one hole (n  =  211) however most holes (70%) showed no tissue aggregates. Contrary to previous belief, choroid plexus (in 24% of VCs) and microglia (2–6% of the cells in obstructing tissue) are not major contributors to obstruction. Large tissue aggregates with comparable cell density were observed in VCs after different durations of implantation suggesting mechanisms that may not increase in severity over time. VCs from patients with 0 to 2 lifetime revisions had a larger fraction of VC holes obstructed than VCs from patients with 10+ revisions (p = 0.0484). VCs contacting the ventricular wall were more likely to have holes with protruding tissue aggregates as compared to VCs that did not (p = 0.005).

**Conclusions**: VC obstruction appears to be a multifactorial problem with no single factor independently predicting the degree of obstruction or the potential duration of patency. Grouping patients by multiple factors may improve our ability to predict and prevent obstruction.

## A72 Blood GFAP, NFL and abeta42/40 Correlate Modestly with CSF levels in idiopathic normal pressure hydrocephalus (iNPH) But Do Not Predict Short Term Response To CSF Diversion

### Alexandria Lewis, Abhay Moghekar

#### ^1^Department of Neurology, Johns Hopkins University School of Medicine, Baltimore, MD, 21231, USA

##### **Correspondence**: Abhay Moghekar, am@jhmi.edu

*Fluids and Barriers of the CNS* 2023, **20(Suppl 2)**: A72

**Introduction** : The role of biomarkers in the selection of idiopathic normal pressure hydrocephalus (iNPH) patients for shunt surgery has been studied in small populations and mainly in CSF. In this study, we evaluated CSF and plasma from a large cohort of iNPH patients referred for shunt surgery. The study aimed to evaluate the correlation of plasma and CSF biomarkers and determine if plasma measures could help in selecting patients for shunt surgery.

**Methods** : CSF and plasma were obtained from patients referred for a CSF diversion procedure after a baseline assessment at the Johns Hopkins Center for CSF Disorders. All patients had a battery of cognitive and gait testing prior to their procedure. Patients deemed to be responders were referred for surgery based on iNPH guidelines. Plasma and CSF [Neurofilament Light (NF-L) and Glial fibrillary acidic protein (GFAP)] and canonical Alzheimer’s disease-related biomarkers [abeta-42 (aβ42), abeta-40 (aβ40), were analyzed on the Quanterix HDX platform.

**Results** : 338 patients (211 male, 127 female) underwent a lumbar puncture or extended CSF drainage as part of their INPH assessment. 135 patients were selected for shunt surgery. Plasma NFL correlated modestly with CSF NFL (R = 0.42; p < 0.001). Plasma GFAP correlated modestly but significantly with CSF GFAP (R = 0.34; p < 0.001). Plasma aβ42/aβ40 correlated weakly with CSF aβ42/aβ40 (R = 0.2; p < 0.001). The median plasma biomarker levels were not statistically different in those selected for surgery. Plasma measures could not reliably predict who improved short term after the diversion procedure.

**Conclusions** : Similarly, CSFand plasma measures could not predict who would improve after a CSF diversion and cannot be used in selection for shunt surgery. Plasma levels of GFAP and NFL along with the aβ42/aβ40 ratio did correlate modestly with respective CSF levels and an ongoing study will determine if they predict long term improvement.

## A73 Watkins 2.0: The Next Generation In Gait-Assessment Apps For Normal Pressure Hydrocephalus And Decompensated Long-Standing Overt Ventriculomegaly Patients

### Kanza Tariq, Ahmed Toma, Lewis Thorne, Laurence Watkins

#### ^1^National Hospital for Neurology and Neurosurgery, Queen Square, London, UK

##### **Correspondence**: Kanza Tariq kanza.tariq@nhs.net

*Fluids and Barriers of the CNS* 2023, **20(Suppl 2)**: A73

**Introduction**: The timed-up-and-go (TUG) test and the timed-10-meter-walking test (10MWT) are frequently used assessments tools for gait and balance disturbances in normal pressure hydrocephalus (NPH) and long-standing overt ventriculomegaly (LOVA). We aimed to make a smart-phone app which performs both the 10MWT and the TUG-test, thus making it possible for patients to perform repeat assessments in their home environment and have an objective measure of their progress for themselves and for their clinical team.

**Methods**: 10MWT and TUG-test were performed by 50 healthy adults, 67 NPH, 10 LOVA and 5 elderly patients with other conditions, using the Watkins2.0 app. The 10MWT was assessed with timed slow-pace and fast-pace. Statistical analysis used SPSS (version25.0, IBM) by paired t-test, comparing the healthy and the NPH cohorts. Level of precision of the app (as compared to a clinical observer using a stopwatch) was evaluated using a receiver operating characteristic (ROC) curve.

**Results**: As compared to a clinical observer using a stopwatch:

10MWT the app showed 100% agreement to the number of whole seconds in the time taken to cover distance, 95% accuracy in the number of steps taken (error ± 1–3 steps), and 97% accuracy in the measure of total distance covered (error ± 0.25–0.50 m).

The TUG test has 100% agreement to the number of whole seconds in the time taken to complete the test, 97% accuracy in the number of steps (error of ± 1–2 steps) and 87.5% accuracy in the distance covered with error of ± 0.50 meter. In the measure of time, the app was found to have equal sensitivity as a clinical observer. In measure of number of steps and distance, the app demonstrated high sensitivity and precision (AUC > 0.9). The app also showed significant level of discrimination between healthy and gait-impaired individuals.

**Conclusion**: Watkins2.0 is an efficient app for objective performance of 10MWT and the TUG-test.

## A74 Quantitative measurement of finger tapping before and after the tap test in idiopathic normal pressure hydrocephalus

### Yoko Shimizu^1,2^, Mitsuya Horiba^1^, Kento Sahashi^1^, Shoji Kawashima^3^, Akihiko Kandori^4^, Motoki Tanikawa^2^, Shigeki Yamada^2^, Noriyuki Matsukawa^3^, Hideki Okamoto^1^, Yoshino Ueki^1^, Mitsuhito Mase^2^

#### ^1^Department of Rehabilitation Medicine, Nagoya City University Graduate School of Medical Science, Nagoya, Japan; ^2^Department of Neurosurgery, Nagoya City University Graduate School of Medical Sciences, Nagoya, Japan; ^3^Department of Neurology and Neuroscience, Nagoya City University Graduate School of Medical Science, Nagoya, Japan; ^4^Hitachi, Ltd, Research and Development Group, Center for Exploratory Research, Tokyo, Japan

##### **Correspondence**: Yoko Shimizu, otyokos@med.nagoya-cu.ac.jp

*Fluids and Barriers of the CNS* 2023, **20(Suppl 2)**: A74

**Introduction**: Several previous studies reported that patients with idiopathic normal pressure hydrocephalus (iNPH) have not only lower limb dysfunction but also the upper limb.

We have already reported that finger dexterity of iNPH patients is obviously impaired by quantitative assessment of finger tapping (F-T) with auditory stimuli. Based on this, we analyzed how the finger dexterity of iNPH patients changed before and after tap test (T-T).

**Methods**: Subjects were 51 possible iNPH patients that underwent T-T, 18 T-T non-responders (mean age 79.6±7.1 years; male 9/18) and 33 T-T responders that were probable iNPH (76.3 ± 7.8 years; male 15/18) according to the Japanese Guidelines for NPH.

Twenty of the 33 probable iNPH underwent shunt surgery and were diagnosed with definite iNPH. We performed F-T measurements before and after T-T as well as after shunt surgery.

F-T performance of the index finger and thumb was quantified using magnetic sensing device.

The participants performed repetitive tapping , following to the pace at the rate of 2.0Hz. We evaluated F-T parameters including the mean of maximum amplitude (M-Amplitude), and closing velocity (cl-Velocity).

**Results**: In non-responders of T-T, there was no significant difference in both M-Amplitude and cl-Velocity between before and after the T-T. In patients with probable iNPH, M-Amplitude (P = 0.04) and cl- Velocity (P = 0.01) were significantly increased after T-T.In difinite iNPH patients, M-Amplitude (P = 0.006) and cl-Velocity (P = 0.001) showed significantly much larger incresed after T-T. (These increases reflect improvements in finger dexterity.)

**Conclusions**: The paced F-T test could become a new tool for evaluating the upper extremities function in iNPH before and after T-T.

## A75 Machine Learning Analysis of Voice Data from Patients with Normal Pressure Hydrocephalus

### Ki-Su Park^1^, Jiho Lee^2^, Janghyeok Yoon^2^, Ji-Wan Ha^3^, Kyunghun Kang^4^, Eunhee Park^5^

#### ^1^Department of Neurosurgery, School of Medicine, Kyungpook National University, Daegu, Republic of Korea; ^2^Department of Industrial Engineering, Konkuk University, Seoul, Republic of Korea; ^3^Department of Speech Pathology, Daegu University, Gyeongsan, Republic of Korea; ^4^Department of Neurology, School of Medicine, Kyungpook National University, Daegu, Republic of Korea; ^5^Department of Physical and Rehabilitation Medicine, Kyungpook National University Medical Center, Daegu, Republic of Korea.

##### **Correspondence**: Janghyeok Yoon, janghyoon@gmail.com

*Fluids and Barriers of the CNS* 2023, **20(Suppl 2)**: A75

**Introduction**: Several studies have reported an association between normal pressure hydrocephalus (NPH) and speech impairment. However, there has been no analysis of the relationship between voice and NPH using artificial intelligence (AI) techniques. The study aimed to explore the possibility of identifying acoustic features that can differentiate NPH from other neurodegenerative diseases by analyzing the voice of patients with NPH using machine learning (ML).

**Methods**: We enrolled 54 patients with NPH from a total of 196 patients with cognitive impairment who had a score of 24 or less on the Korean Mini-Mental State Examination. We analyzed the following acoustic parameters using a boosting ML algorithm: (a) the prolongation of vowels as /a/, /i/, and /u/; (b) the alternative motion rate (AMR) as /puh-puh-puh/, /tuh-tuh-tuh/, and /kuh-kuh-kuh/; (c) the sequential motion rate as /puh-tuh-kuh/; (d) the maximum phonation time (MPT); (e) voice quality; (f) voice variability; and (g) speech rate.

**Results**: First, we found that the meaningful speech tasks related to the discrimination between neurodegenerative brain diseases and other diseases were the MPT (precision: 0.643 and recall: 1) and the AMR as /kuh-kuh-kuh/ (precision: 1.000 and recall: 0.667). Next, the speech tasks that can differentiate NPH from other neurodegenerative brain diseases were /tuh-tuh-tuh/ (precision: 1.000 and recall: 0.875) and /kuh-kuh-kuh/ (precision: 0.889 and recall: 1.000) of AMR.

**Conclusions**: Although this was an artificial intelligence analysis using a small number of voice data, it suggests that there may be acoustic features that can differentiate NPH from other diseases.

## A76 Optimizing Gait and Balance Metric Acquisition from the “Tap Test” in Normal Pressure Hydrocephalus

### Philip W. Tipton^1^, Olga P. Fermo^1^, Kaisorn L. Chaichana^2^, Christian Lachner^1,3^, Gregory S. Day^1^, Sanjeet S. Grewal^2^, Bjorn E. Oskarsson^1^, Nilufer Ertekin-Taner^1,4^, Zbigniew K. Wszolek^1^, Neill R. Graff-Radford^1^

#### ^1^Department of Neurology, Mayo Clinic, Jacksonville, Florida, USA; ^2^Department of Neurosurgery, Mayo Clinic, Jacksonville, Florida, USA; ^3^Department of Psychiatry and Psychology, Mayo Clinic, Jacksonville, Florida, USA; ^4^Department of Neurosurgery, Mayo Clinic, Jacksonville, Florida, USA

##### **Correspondence**: Philip W Tipton, tipton.philip@mayo.edu

*Fluids and Barriers of the CNS* 2023, **20(Suppl 2)**: A76

**Introduction**: Gait and balance impairments are hallmarks of normal pressure hydrocephalus (NPH). Ambulatory and balance improvement following high-volume lumbar puncture (HVLP) predicts shunt responsiveness, the so-called “Tap Test.” However, it is uncertain which and when gait and balance measures should be acquired after the HVLP.

**Methods**: This pilot study identified 12, of 14 shunted patients whose ambulation improved after shunting. All patients underwent gait/balance assessments prior to (Pre), within 1 h of (Post1), and within 24 h (Post2) of a HVLP. We assessed changes in the time to complete a Timed Up and Go (TUG), 25-feet extended Timed Up and Go (ETUG), and 360-degree turn and counted the steps needed to turn 360 degrees. We acquired truncal sway metrics from a lumbar-positioned wearable inertial measurement unit (Opal^TM^, APDM) while patients completed brief quiet standing tasks including feet apart/eyes open (FAEO), feet apart/eyes closed (FAEC), feet together/eyes closed (FTEC). We used paired t-tests to assess changes from Pre to Post measures.

**Results**: We observed that ≥ 92% of subjects improved on Post2 TUG time, ETUG time, 360-degree turn time, and 360-degree turn step number. Fewer patients improved on Post1 assessments (p > 0.05). FAEO centroidal frequency in the sagittal plane significantly increased by 12–16% in 83% and 75% of subjects at Post1 and Post2 assessments, respectively. FAEC frequency dispersion in the sagittal plane significantly decreased by 5–6% in 67% and 75% of subjects at Post1 and Post2 assessments, respectively.

**Conclusions**: These pilot data suggest that longer measures of gait (ETUG) and turning at 24 h, rather than within 1 h, of a HVLP may be more predictive of surgical outcomes. Balance measures require additional study. With these preliminary data, we can design a prospective study to show which of these metrics, or combinations, is the best predictor of surgical outcomes.

## A77 Lumboperitoneal (LP) shunt in the patients with idiopathic normal pressure hydrocephalus (iNPH): Surgical technique

### Naoyuki Samejima, Nobmasa Kuwana, Akira Watanabe

#### ^1^NPH Center, Department of Neurosurgery, Tokyo Kyosai Hospital, Tokyo, Japan

##### **Correspondence**: Naoyuki Samejima samejima@tkh.meguro.tokyo.jp

*Fluids and Barriers of the CNS* 2023, **20(Suppl 2)**: A77

**Introduction**: The SINPHONI-2 study (Japanese prospective multicenter cohort studies) was carried out and showed the safety and efficacy of LP shunt surgery for iNPH. Although our high level of success with surgery may be a minor point, it is worth reporting, as minor differences in technique and know-how can markedly affect the efficacy of shunt surgery. We showed a video of our LP shunt procedure.

**Methods**: A total of 758 probable iNPH patients underwent LP shunt surgery at our NPH center between April 2009 and December 2021 (mean age 77.5 ± 6.5). Aspects of our surgical technique include: 1) General anesthesia, 2) Use of the original drape, 3) Upward insertion of the spinal tube through L2/3 via a paramedian puncture for highly deformed lumbar spine patients, using the intraoperative C-arm imaging. 4) Placement of a Codman-Hakim programmable valve with Siphonguard™ in the back, 5) Inclination of the table at 30°angle. 6) Laparotomy via rectal muscle splitting, and 7) Running the peritoneal tube obliquely from upper lateral to lower medial (to eliminate the space permitting tube expulsion)

**Results**: Of the 693 patients followed up at our NPH Center for 1 year after LP shunt surgery. During the first year after surgery, 68 of 693 patients (9.8%) developed postoperative complications including tube occlusion in 24(3.5%), chronic subdural hematoma requiring evacuation in 18(2.6%), migration of the spinal or abdominal tube in 12(1.7%), lower limb numbness in 7, rupture of the spinal tube in 5, and shunt infection in 2.

**Conclusions**: Our LP shunt procedure generally seems to be acceptable from the viewpoint of complications. The low-invasive LP shunt that does not require ventricular puncture is preferred and has become the first-line procedure for iNPH in Japan. We would like to popularize the use of this surgical procedure worldwide in the future.

## A78 Neuralgia after Lumboperitoneal Shunt Can Be Reduced with Surgery under Local Anesthesia

### Yasuaki Inoue, Ryo Oike

#### ^1^Department of Neurosurgery, Nadogaya Hospital, Kashiwa, Japan

##### **Correspondence**: Yasuaki Inoue, Inoue.yasuaki@gmail.com

*Fluids and Barriers of the CNS* 2023, **20(Suppl 2)**: A78

**Introduction**: Controlling complications after shunt surgeries for idiopathic normal pressure hydrocephalus (iNPH) is essential. We report the data of complications and the preventive measures from a facility where lumboperitoneal shunt (LPS) is the first-line treatment for iNPH. We initiated LPS under local anesthesia, believing that asking the patient if they feel any pain while inserting the spinal catheter can decrease the incidence of neuralgia. The result is discussed.

**Methods**: We reviewed the medical records of consecutive iNPH patients for whom a shunt surgery was provided from 5/1/2020 to 3/16/2023. The incidence and the characteristics of postoperative complications were compared between LPS and ventriculoperitoneal shunt (VPS) and between LPS under local anesthesia and LPS under general anesthesia.

**Results**: A total of 218 patients were included, with 152 LPS (69.7%), 60 VPS (27.5%), and six ventriculoatrial shunts (0.8%). The overall rates of complication were 16.6% (36/217) 1 month after surgery (POM1), 9.8% (19/193) 3 months after surgery (POM3), 4.4% (7/159) 6 months after surgery (POM6), and 1.9% (2/106) 12 months after surgery (POM12). Complications after LPS were reported in 18.5% (28/151) POM1, 12.9% (17/132) POM3, 4.9% (5/103) POM6, and 3.1% (2/64) POM12, respectively.

In the LPS group, CSDH was seen in 10 (8.1%) patients POM1 and 5 (3.8%) POM3, while headache was experienced in 8 (5.3%) POM1 and 2 (1.5%) POM3. These conditions likely related to intracranial hypotension were statistically more significant in LPS than in VPS (13.9 % vs. 3.3%, p = 0.026).

A significantly smaller number of patients complained of neuralgia after LPS under local anesthesia than general anesthesia (1.2% vs. 7.7%, p = 0.042).

**Conclusions**: LPS may carry a greater risk of reversible intracranial hypotension than VPS, while neuralgia after LPS can be reduced with surgery under local anesthesia.

## A79 Impact of shunt placement in CSF dynamics

### Cyrille Capel^1,2^, Kimi Owashi^2,3^, Serge Metanbou^4^, Olivier Balédent^2,3^

#### ^1^Neurosurgery, University hospital, Amiens, France; ^2^CHIMERE UR7516, Jules Verne University , Amiens, France; ^3^Image processing, University hospital, Amiens, France; ^4^Radiology, University hospital, Amiens, France

##### **Correspondence**: Olivier Balédent, olivier.baledent@chu-amiens.fr

*Fluids and Barriers of the CNS* 2023, **20(Suppl 2)**: A79

**Introduction**: In so-called “normal pressure hydrocephalus” paradoxically Intracranial pressure and CSF dynamics are not normal. We also know that there are hyper dynamic CSF flows in the aqueduct and flow alterations in the spinal compartment. The aim is to investigate how CSF dynamics change after ventricular shunt surgery in hydrocephalus patients who have improved clinically.

**Methods**: We included 14 patients with hydrocephalus who had improved after ventriculoperitoneal shunting. We referred to these patients as patients with active reversible hydrocephalus (ARH). These patients underwent phase contrast MRI preoperatively, at 6 and 12 months postoperatively. We quantified the oscillating CSF (stroke volume) in the aqueduct (SV_aqu_) and in the cervical canal (SV_cerv_). We calculated the ratio of SV_aqu_ to SV_cerv_ called CSF_ratio_, which reflects the participation of the intraventricular compartment in the overall pulsatility of intracranial CSF through the spinal canal.

**Results**: 6 months after shunt placement SV_AQU_ significantly (p = 0.003) decreased from 240±114μL/cc to 214±157μL/cc. (In two cases, SV_aqu_ increased). 12 months after shunt placement SV_aqu_ volume continued to decrease non significantly (p = 0.12) to 193±145μL/cc. (In two cases, SV_aqu_ increased)

SV_cerv_ significantly (p = 0.007) decreased from 627±229μL/cc to 557±234μL/cc and 12 months after shunt placement SV_cerv_ continued to significantly (p = 0.001) decrease to 496±234μL/cc. CSF_ratio_ was not changed by surgery. It was measured at 40±20%.

**Conclusion**: Our study showed that intraventricular and spinal CSF dynamics increased in patients with ARH and decreased only slightly after shunt placement. Shunt placement primarily treats symptoms rather than CSF dynamics. CSF dynamics may help to diagnose shunt dysfunction only if CSF dynamics begin to increase again compared with previous measurements. In these patients who have improved after shunt but continue to have elevated CSF ventricular dynamics, such CSF dynamics do not appear aggressive but instead it is important to ensure a secondary compliance to the one probably altered.

## A80 Influence of ventricular size and surgical approach on flow distribution in ventricular catheters

### Cristopher Roberts, Prashant Hariharan, Carolyn Harris, Bryn Martin

#### ^1^Wayne State University, Michigan, USA

##### **Correspondence**: Cristopher Roberts bb9561@wayne.edu

*Fluids and Barriers of the CNS* 2023, **20(Suppl 2)**: A80

**Introduction**: Incremental progression has been made with ventricular catheter (VC) design apart from antibiotic impregnation. Prior work using computational fluid dynamics (CFD) has used a cylinder to replicate a hydrocephalic ventricle to study CSF behavior in VCs. Accurate representation of cerebrospinal fluid (CSF) flow patterns is difficult in these models because of the lack of dynamic ventricular shape. Capturing the complex ventricular morphology is imperative to investigate CSF flow patterns. Using patient-specific ventricles generated from MRIs, we investigate the impact of ventricular size and shape with various VC designs, including CSF parameters of mass flow rate, shear stress, and pressure gradient. We aim to elucidate the influence of ventricular size and surgical trajectory on these parameters to optimize VC design.

**Methods**: Enlarged lateral ventricles were extracted from magnetic resonance images to create computational 3D renders. Catheters were inserted into the lateral ventricles using anterior and posterior trajectories. Physiological boundary conditions were placed at the choroid plexus and catheter outlet. Flow parameters, including mass flow rate, shear stress, and pressure gradients, were quantified in the catheter drainage holes and lumen under steady-state conditions.

**Results**: These data suggest that the mass flow rate, shear stress, and pressure gradient within the catheter holes display no significant variation across different ventricular volumes, irrespective of the surgical approach employed. Nevertheless, a discernible variation in the ventricular pressure is observed, which may be attributed to the different resistances to flow among the catheters, causing variations in ventricular pressure without affecting the mass flow rate, shear stress, and pressure gradients in the catheter.

**Conclusions**: The present findings suggest that the current commercial catheters exhibit similar flow characteristics across all tested parameters concerning surgical approach and ventricular size. However, the overall resistance to flow of the catheters appears to vary depending on the surgical approach rather than the ventricular size.

## A81 Shunt testing in vivo: Performance of Certas Valve integrated with SiphonGuard

### Marek Czosnyka, Benjamin Dias-Dougan, Indu Lawes, Zofia Czosnyka, Alexis Joannides

#### ^1^Division of Neurosurgery, Cambridge University Hospital, Cambridge, UK

##### **Correspondence**: Marek Czosnyka , mc141@medschl.cam.ac.uk

*Fluids and Barriers of the CNS* 2023, **20(Suppl 2)**: A81

**Introduction**: Testing of hydrocephalus shunt using infusion test through a prechamber has proved to be both precise and objective. Its most important advantage is avoidance of unnecessary revision of a properly working shunt in patients presenting with symptoms similar to those caused by blockage. But not all shunts can be tested in this way. We recently discovered that testing Certas Valve integrated with SiphonGuard (siphon controlling device) should be interpreted with special caution.

**Methods**: Since finishing of first COVID lockdown (2020) 12 patients with implanted Certas Valve were tested. Two 25G butterfly needles were inserted through triple-cleaned skin in shunt prechamber. One needle was connected to pressure transducer, another to infusion pump. Baseline pressure with present pulsatile waveform was recorded over a 10 min period. Then infusion of Hartmann’s solution with a rate of 1.5 ml/min started and pressure increased gradually to plateau value. If the plateau is lower than shunt performance level plus infusion rate times hydrodynamic resistance of shunt system, the system is judged to drain properly.

**Results**: In 7 cases, tests revealed properly working shunts. But in 5 cases, after reaching plateau at 12–18 mm Hg (depending on shunt setting) during infusion, pressure suddenly increased to 40 mm Hg or above, showing shape of ICP resembling plateau wave. Infusion was discontinued immediately, and the test was reported as indicating inability of shunt to stabilize ICP at safe level. Further inspection and in one case using infusion with decreasing rate (1.5, 1 and 0.5 ml/min) showed that ‘plateau wave’ disappears with lower infusion rate.

**Conclusion**: SiphonGuard works as flow regulator. During infusion through the shunt prechamber switching from low to high resistance state may happen. It results in sudden, plateau wave -like rise in ICP. For testing Certas valve with SiphonGuard lower infusion rate should be used.

## A82 Prognostic Significance Of Cerebrospinal Fluid Production Rate In Idiopathic Intracranial Hypertension And The Impact Of Medical Therapy

### Kanza Tariq, Ahmed Toma, Sogha Khawari, Lewis Thorne, Laurence Watkins

#### ^1^National Hospital for Neurology and Neurosurgery, Queen Square, London, U.K

##### **Correspondence**: Kanza Tariq kanza.tariq@nhs.net

*Fluids and Barriers of the CNS* 2023, **20(Suppl 2)**: A82

**Introduction**: The management of idiopathic intracranial hypertension (IIH) is quite varied. While the Idiopathic Intracranial Hypertension Treatment Trial advocates the efficacy of acetazolamide in the treatment of IIH, the VISION study is comparing the effectiveness of venous stenting to CSF shunting. Our study aimed to demonstrate net cerebrospinal fluid production rate (PRcsf) as a potential prognostic tool for the management of IIH.

**Methods**: A prospective observational study was performed in all IIH patients in our hospital who required CSF drainage as part of their ongoing management. The drain was connected to a LiquoGuard7 (Möller-Medical, Germany) with the intracranial pressure sensor at the level of the external auditory meatus. The internal software and flow-rate data of the LiquoGuard7 pump was used to calculate net PRcsf in the patients. Patients were flat for 30 min during the measurement. Patient’s notes and electronic health records were reviewed daily. The events occurring during the course of hospital stay for each patient, and prognosis and patient outcome was recorded as well as resolution of papilledema. Statistical analysis used SPSS (version 25.0, IBM) by multivariate multiple regression, comparing the resolution of papilledema to the measured PRcsf, age, gender, body-mass-index, co-morbidities, and the type of medical treatment received.

**Results**: 15 IIH patients were studied. IIH patients not using acetazolamide (n = 9) demonstrated average PRcsf of 86 ml/h±10SD. These patients benefitted from CSF-shunting, even if they had previously undergone stenting (n = 2). Acetazolamide (AZM) influenced PRcsf in a dose-dependent manner: AZM-Dose 250mg thrice daily (n = 5), average PRcsf of 45 ml/h ± 3SD, papilledema resolved with stenting; AZM-Dose 1000 mg twice daily (n = 1), average PRcsf of 22 ml/h ± 3SD, no additional treatment required.

**Conclusion**: PRcsf may hold prognostic value towards treatment-response in patients with IIH.

## A83 The Peking University Medical College Hospital (PUMCH) Evaluation System of Idiopathic Normal Pressure Hydrocephalus and Clinical Practice

### Rui Yin^1*^, Junxian Wen^1*^, Jianbo Chang^1^, Xiao Zhang^1^, Caiyan Liu^2^, Jing Gao^2^, Yihao Chen^1^, Lang Yang^1^, Xiying Dong^1^, Feng Feng^3^, Hui You^3^, Wei Zuo^4^, Junji Wei^1^

#### ^1^Department of Neurosurgery, Peking Union Medical College Hospital, Chinese Academy of Medical Science & Peking Union Medical College, 100730 Beijing, China; ^2^Department of Neurology, Peking Union Medical College Hospital, Chinese Academy of Medical Science & Peking Union Medical College, 100730 Beijing, China; ^3^Department of Radiology, Peking Union Medical College Hospital, Chinese Academy of Medical Science & Peking Union Medical College, 100730 Beijing, China; ^4^Department of Pharmacy, Peking Union Medical College Hospital, Chinese Academy of Medical Science & Peking Union Medical College, 100730 Beijing, China

##### **Correspondence**: Junji Wei, weijj1999@163.com

*the authors contributed equally to this work

*Fluids and Barriers of the CNS* 2023, **20(Suppl 2)**: A83

**Introduction**: Differentiating idiopathic normal pressure hydrocephalus (iNPH) from other neurodegenerative diseases is challenging. Only a portion of the patients clinically suspected of iNPH would respond to surgical intervention. CSF tap test is usually used to predict surgery outcomes and hence aid clinical decision-making, but the work-up varies from center to center.

**Objective**: To introduce the PUMCH evaluation system which we conducted at our center during CSF tap test and examine its power by analyzing data from a series of iNPH cases that underwent shunt placement. To analyze common features in the past medical history of our patients and investigate whether they are related to the etiology of iNPH.

**Methods**: Data from 20 patients who were positive in the tap tests pre-operatively with

PUMCH evaluation system and received ventriculoperitoneal shunting were retrospectively analyzed. Pre-operative and post-operative performance data were analyzed. History of any underlying medical conditions was also taken into consideration.

**Results**: We performed VP shunt placement in 20 NPH patients from October 2019 to February 2022. 80% of them exhibited improvement in at least 1 of the clinical triad, proving the predictive power of the PUMCH test workflow. The underlying conditions like hypertension, diabetes and insufficiency in cerebral blood supply were also found to be associated with the onset of NPH.

**Conclusion**: Our PUMCH evaluation system is a valid tool for NPH assessment and can guide clinical decision-making. Comorbidities should be taken into consideration as they contribute to the pathogenesis and progression of NPH. Better identification of potential iNPH patients will lower the burden exerted on the family and the aging society.

## A84 Consistency and sensitivity analysis of instrumental and traditional methods for evaluating the CSF tap test (CSF-TT) responsiveness in patients with iNPH

### Chunyan Liu^a^, Hongliang Li^a^, Qiong Yang^a^, Yanfeng Li^b^, Yan Xing^a*^

#### ^a^Department of Neurology, Aviation General Hospital, 100012 Beijing, China; ^b^Department of Neurology, Peking Union Medical College Hospital, Beijing, China 100730

##### **Correspondence**: Yan Xing drxingyan@163.com

*Fluids and Barriers of the CNS* 2023, **20(Suppl 2)**: A84

**Introduction**: Idiopathic normal pressure hydrocephalus (iNPH) is characterized by gait, cognition and urinary dysfunction. Here we evaluate the consistency and sensitivity of the instrumental （ReadyGo motor function quantitative evaluation system）and traditional manual gait measurement methods (10-meter walk test (10MWT) and 3-meter timed up and go test (3m-TUG)) for evaluating the CSF tap test (CSF-TT) responsiveness in patients with iNPH.

**Method**: 40 iNPH patients (21 males and 19 females, with an average age of 71.71 ± 4.43 years) were prospectively enrolled and subjected to CST-TT. ReadyGo and 10MWT and TUG were done simultaneously.

**Results**: Instrumental gait analysis parameters were highly linearly correlated with manually measured parameters: ReadyGo was highly linearly correlated with 10MWT (speed correlation coefficient is 0.81, step number correlation coefficient is 0.70, and step frequency correlation coefficient is 0.77); ReadyGo was highly linearly correlated with 3m-TUG (r = 0.89). The sensitivity of gait analysis recorded by instrumental recorders was higher than that of traditional manual gait measurements, and can provide more dimensional parameters for gait evaluation.

**Conclusion**: The ReadyGo quantitative motor function assessment system showed more advantages in objectivity, quantitation, and automated calculation, and has the potential for clinical application.

**Keywords**: CSF tap test (CSF-TT); idiopathic normal pressure hydrocephalus (iNPH);10-meter walk test (10MWT) ; 3-meter timed up and go test (3m-TUG)

## A85 Effectiveness of the Clock Drawing test in the Tap Test

### Koichi Miyazaki

#### ^1^Department of Neurosurgery, Osaka Kaisei Hospital, Osaka, Osaka, 532-0003, Japan

##### **Correspondence**: Koichi Miyazaki, rpmy69126@nike.eonet.ne.jp

*Fluids and Barriers of the CNS* 2023, **20(Suppl 2)**: A85

**Introduction**: The Clock Drawing Test (CDT) is a simple and effective test to evaluate cognitive function, often used for diagnosing dementia. We have been applying the CDT in addition to the Mini-Mental State Exam (MMSE) and the Frontal Assessment Battery (FAB) in the Tap Test (TT). In this study, we retrospectively examined the effectiveness of the CDT in the TT.

**Methods**: We analyzed the data of consecutive 56 patients with iNPH who underwent TT at our hospital. The CDT score was assessed using Freedman's 15-point scoring system, and we evaluated the change in values before and after the tap. The value after tap was the highest score observed between the day of tap and the 7th day. We also similarly compared the changes in MMSE and FAB. Furthermore, we compared the change among three groups according to comorbidities: iNPH without comorbidities (iNPH group), iNPH with Alzheimer's disease (AD-iNPH group), and iNPH with vascular dementia (VaD-iNPH group).

**Results**: In the analysis of all subjects, the CDT score before tap was 10.2 ± 3.2, and we observed an increase of 2.6 ± 2.0 points after tap (P < 0.0001). In the analysis by comorbidity, the AD-iNPH group showed a smaller increase in MMSE score after tap than the iNPH group (2.0 ± 2.3 points, P = 0.099) but showed a significantly larger increase in CDT score (3.6 ± 2.3 points, P = 0.022) similar to the iNPH group (2.7 ± 2.0 points, P < 0.001). The VaD-iNPH group showed a tendency to have a smaller increase in CDT score than the iNPH group, with an increase of 1.9 ± 1.7 points (P = 0.013).

**Conclusions**: The CDT is an effective useful indicator as an evaluation item for the TT, especially in cases of comorbid AD.

## A86 Efficacy and safety of a novel programmable valve (Sphera Pro^®^) in the treatment of idiopathic normal pressure hydrocephalus: a multicenter study

### Rodolfo C. Reis^1^, Marina M. Melo^1^, Matheus M. Mendes^2^, Gabriel A. da S. Mendes^3^, Camila S. Cechi^4^, Manoel J. Teixeira^2^, Fernando C. G. Pinto^2^, José M. Rotta^1^

#### ^1^Department of Neurosurgery, IAMSPE, São Paulo, SP, Brazil; ^2^Department of Neurosurgery, Hospital das Clínicas, University of São Paulo, SP, Brazil; ^3^Physiotherapy Section, IAMSPE, São Paulo, SP, Brazil; ^4^Neuropsychology Section, Hospital das Clínicas, SP, Brazil

##### **Correspondence**: Rodolfo Casimiro Reis, rodolfocr84@yahoo.com.br

*Fluids and Barriers of the CNS* 2023, **20(Suppl 2)**: A86

**Introduction**: Idiopathic normal pressure hydrocephalus (iNPH) is a syndrome characterized by gait disturbance, cognitive impairment, and urinary incontinence, affecting mainly the elderly. Recently we showed in a pilot study that treating iNPH with a novel programmable valve (Sphera Pro®) is cost-effective. Here we aim to describe the efficacy and safety of Sphera Pro® valve in a large cohort of patients.

**Methods**: A prospective cohort of iNPH patients treated with Sphera Pro® programmable valve with gravitational unit was followed for 1 year in two tertiary public hospitals. Clinical improvement, measured by NPH Japanese Scale and compared pre- and postoperatively (3, 6 and 12 months), and complications were registered. Differences in the evolution of the NPH Japanese scale score over time were assessed using generalized estimating equations (GEE).

**Results**: A total of 30 patients with probable iNPH were prospectively recruited. Mean age of patients was 75.7 (±7.87) years-old, 53.3% were female. Cognitive impairment was present in 90% of patients, meanwhile gait disturbance and urinary incontinence in 96.6%. Time from the onset of symptoms to surgery was 37.2 (±13.2) months. NPH Japanese Scale score decreased over time (p < 0.001) from 6.1 (±2.2) preoperatively to: 4.0 (±2.9), 3.8 (±2.6) and 3.8 (±2.5), 3, 6 and 12 months postoperatively (respectively). 86.6% of patients had a sustained decrease in NPH Japanese Scale score during follow-up. Only two patients (6.6%) had complications during follow-up, which were chronic subdural effusions needing surgery. There were no deaths during the study.

**Conclusions**: In this prospective multicenter study, Sphera Pro® valve with gravitational unit was effective in treating iNPH patients, with few complications.

## A87 Placebo–controlled effectiveness in INPH shunting (PENS) – Study Update

### Mark Luciano^1^, Richard Holubkov^2^, Sean Nagel^3^, Jeffrey Wisoff^4^, Guy McKhann, II^5^, Thomas Zwimpfer^6^, Richard Edwards^7^, Michael Williams^8^, Jan Malm^9^, Abhay Moghekar^10^, Anders Eklund^9^, James Golomb^11^, Heather Katzen^12^, Nickolas Dasher^13^, Michael Meggyesy^1^, Mark Hamilton^14^

#### ^1^Neurosurgery, ^10^Neurology, Johns Hopkins School of Medicine, Baltimore, USA; ^2^University of Utah, Salt Lake City, USA; ^3^Neurosurgery, Cleveland Clinic Foundation, Cleveland, USA; ^4^Pediatric Neurosurgery, 11Neurology, NYU Langone Health, New York City, USA; ^5^Neurological Surgery, Columbia University School of Medicine, ^6^Neurosurgery, University of British Columbia, Vancouver, Canada; ^7^Neurosurgery, Southmead Hospital, Birstol, UK; ^8^Neurology, ^13^Rehabilitation Medicine, University of Washington School of Medicine, Seattle, USA; ^9^Neurology, Umeå University, Umea, Sweden, ^12^Neurology, University of Miami Miller School of Medicine, Florida, USA; ^14^Neurosurgery, University of Calgary School of Medicine, Calgary, Canada

##### **Correspondence**: Mark Luciano, markluciano@jhmi.edu

*Fluids and Barriers of the CNS* 2023, **20(Suppl 2)**: A87

**Introduction**: The Placebo–controlled effectiveness, in INPH shunting (PENS) trial is now in its second year. We will give an update of the trial’s clinical site development and early accrual. Issues in the development of the project will be discussed including screening, criteria for capacity and issues of obtaining consent. Exclusion criteria, either designed to decrease surgical risk, such as anticoagulation, or to optimize shunting, such as gait speed and co-morbidities, will be discussed in the context of an effectiveness versus an efficacy study.

**Methods**: 19 study sites are currently enrolling patients scheduled for ventriculoperitoneal shunting based on response to CSF drainage. Patients are randomized to a Codman® Certas® Plus valve with SiphonGuard® at either setting 4 (Active, N = 9) or setting 8 /”virtual off” (Placebo, N = 9). Patients and assessors are blinded to the shunt setting. Outcomes include 10-meter gait velocity (primary outcome), cognitive function, bladder activity, depression, Modified Rankin scale, and Lawton ADL/IADL scores. The primary analysis is gait velocity improvement in the Active versus Placebo groups at 4-months. Afterwards all shunts are blindly adjusted to the active setting. The long-term evaluation of active shunting in all patients is ongoing.

**Conclusions**: While the trial is moving forward, multiple issues based on site differences and local PI judgment are critical to maintain the validity of the trial and will be presented.

## A88 Safety of Ventriculoperitoneal Shunt Placement in Patients with Idiopathic Normal Pressure Hydrocephalus in the Setting of Systemic Anticoagulation

### Abdelrahman Hamouda^1^, Mahnoor Shafi^1^, Zach Pennington^1^, Hannah Hallak^1^, Jonathan Graff-Radford^2^, David T Jones^2^, Hugo Botha^2^, Jeremy K. Cutsforth-Gregory^2^, Petrice M Cogswell^3^, Benjamin D. Elder^1^

#### ^1^Department of Neurologic Surgery, Mayo Clinic, Rochester, MN, 55905, USA; ^2^Department of Neurology; ^3^Department of Radiology

##### **Correspondence**: Benjamin Elder, elder.benjamin@mayo.edu

*Fluids and Barriers of the CNS* 2023, **20(Suppl 2)**: A88

**Introduction**: Since the iNPH patient population is elderly, many patients have significant medical comorbidities that may require treatment with systemic anticoagulation or antiplatelet therapy. Therefore, the objective of this study was to determine the safety of shunting iNPH patients on systemic anticoagulation.

**Methods**: A total of 232 patients > 60 years of age diagnosed with iNPH underwent shunt placement from 2018–2022. Patients were included in this study if they were treated with anticoagulation preoperatively including warfarin, direct Xa inhibitors (apixaban and rivaroxaban), direct thrombin inhibitors (dabigatran), or antiplatelet therapy with clopidogrel. The patients were retrospectively reviewed for baseline demographics, perioperative complications, and overdrainage.

**Results**: A total of 47 patients were identified who underwent VP shunt placement in the setting of systemic anticoagulation or antiplatelet medication use. The mean modified frailty index was 1.45, and Charles comorbidity Index was 2.98. Seven patients had a history of myocardial infarction, and 18 patients were anticoagulated for treatment of atrial fibrillation. 15 patients were treated with apixaban, 15 patients with coumadin, 1 patient with dabigatran, 9 patients with rivaroxaban, and 6 patients with clopidogrel. Two patients had a minor postoperative tract hemorrhage and five patients had small intraventricular hemorrhages, though none were clinically symptomatic or required intervention. At 1-month follow-up, seven patients (14.9%) had evidence of subdural hematoma formation, with 1 patient in the setting of a traumatic head injury. Two patients (4.3%) required further surgery for treatment of the subdural hematoma, though one of these patients was due to the traumatic injury.

**Conclusions**: Patients with iNPH on long-term anticoagulation or antiplatelet treatment can be safely treated with VP shunt placement, though there is a potentially higher risk of overdrainage and subdural hematoma formation. However, only 1 patient out of the series of 47 required surgical treatment of a subdural hematoma from the shunt.

## A89 Frailty and medium-term overall survival following ventriculoperitoneal shunting for patients with idiopathic Normal Pressure Hydrocephalus

### Elizabeth Cray^1^, Rupert Noad^2^, Samiul Muquit^1^, Samuel Jeffery^1^

#### ^1^Department of Neurosurgery, Southwest Neurosurgery Centre, University Hospital Plymouth, Plymouth, PL6 8DH. UK; ^2^Department of Neuropsychology, University Hospital Plymouth, Plymouth, PL6 8DH, UK

##### **Correspondence**: Elizabeth Cray, elizabethcray@nhs.net

*Fluids and Barriers of the CNS* 2023, **20(Suppl 2)**: A89

**Introduction**: The aim was to evaluate the utility of the modified Frailty Index-11 (mFI-11) in predicting overall survival following ventriculoperitoneal shunt (VPS) surgery for idiopathic Normal Pressure Hydrocephalus (iNPH).

**Methods**: Patients, 65 years and above who underwent VPS for iNPH between 2013 and 2022 were included. Patients were retrospectively scored using the mFI-11 to categorise frailty as low (mF-11 score 0–2), moderate (mFI-11 score 3–5) or high risk (mFI-11 score 6–7). Kaplan–Meier survival curve analysis and log-rank test were used to estimate overall survival and to test for differences amongst frailty groups following surgical intervention.

**Results**: A total of 109 patients (109 procedures) were included; 72 (66%) were males and the mean age was 75.17 (±5.25). The median length of hospital stay was 3.56 days. The mean mFI-11 score was 1.91 (SD 1.259). 75/109 (69%) were categorised as low risk and 34/109 (31%) as moderate risk. There were no patients with a high frailty risk score. In this cohort, univariate analysis showed no significant association between level of frailty and length of stay. Median length of follow up was 55 months and at the time of analysis 86/109 had reached 2 years and 49/109 had reached 5 years of follow up. The estimated median survival following shunt insertion was 6 years and the 5-year cumulative survival was 75%. There was no difference in survival between frailty risk groups (p = 0.66).

**Conclusion**: The mFI-11 is a useful tool that can be readily calculated using available patient data. Frailty is prevalent in this patient group and can be a valuable predictor of mortality. In our patient cohort however, those with low and moderate frailty risk scores had similar medium-term survival. Correlation with prospective assessment of frailty and with functional outcomes following shunt surgery is needed.

## A90 Analysis of Intracranial Pressure Pulse Shape Index in Normal Pressure Hydrocephalus

### Magdalena Kasprowicz^1^, Agnieszka Kazimierska^1^, Eric Schmidt^2^, Olivier Balédent^3^, Agata Pudełko^1^, Marek Czosnyka^4^, Zofia Czosnyka^4^

#### ^1^Department of Biomedical Engineering, Faculty of Fundamental Problems of Technology, Wroclaw University of Science and Technology, Wroclaw, Poland; ^2^Department of Neurosurgery, University Hospital of Toulouse, Toulouse, France; ^3^Department of Imaging and Biophysics, Teaching Hospitals, Jules Verne University of Picardie, Amiens, France; ^4^Division of Neurosurgery, Department of Clinical Neurosciences, Addenbrooke’s Hospital, University of Cambridge, Cambridge, United Kingdom

##### **Correspondence**: Magdalena Kasprowicz, magdalena.kasprowicz@pwr.edu.pl

*Fluids and Barriers of the CNS* 2023, **20(Suppl 2)**: A90

**Introduction**: Years ago, it was observed that as craniospinal compliance (C) decreases, the shape of intracranial pressure (ICP) pulse waveforms gradually shifts from a three-phasic to a sinusoidal-like morphology. However, analysis of ICP pulse shape has not been widely used in clinical practice until recently, mainly due to the challenges with processing large amounts of high-resolution ICP data. With the recent developments in artificial intelligence (AI), this limitation no longer exists. In this study, we applied an AI-based metric called the Pulse Shape Index (PSI) to automatically analyze the morphology of ICP pulses recorded in normal pressure hydrocephalus (NPH) patients. Additionally, we assessed the clinical usefulness of ICP pulse shape analysis by comparing the PSI with the elastance coefficient (E) and resistance to cerebrospinal fluid outflow (Rout).

**Methods**: We retrospectively analyzed ICP recordings performed in 40 NPH patients during intraventricular infusion tests. A residual network model was applied to classify ICP pulse waveforms into four classes: from 1 (normal) to 4 (pathological). Pulse Sape Index (PSI) was calculated as the weighted sum of class numbers in moving 30-sec windows. Compensatory parameters E and Rout were calculated based on the infusion test. The Wilcoxon signed-rank test was used to compare PSI at baseline and during the plateau phase of the infusion test. Results are presented as median ± IQR.

**Results**: Pre-infusion PSI was elevated in comparison to values previously reported in patients after head injury without deep intracranial hypertension. PSI increased slightly during the infusion test (2.96±1.26 vs. 3.05±0.84, p < 0.0001). Pre-infusion PSI was significantly correlated with E (R = 0.41, p < 0.008) but did not correlate with either Rout or baseline ICP.

**Conclusions**: In general, the shape of ICP pulse is altered in NPH. Patients with higher E demonstrate more pathological ICP pulse morphology. Further studies are required to confirm the usefulness of PSI in clinical practice.

## A91 Oscillation of Intracranial Pressure (ICP) during walking is locked to head motion

### Matthew J. Bancroft^1,2,3^, Eleanor M. Moncur^1,3^, Lewis Thorne^1,3^, Laurence D. Watkins^1,3^, Ahmed K. Toma^1,3^

#### ^1^Department of Brain Repair and Rehabilitation, UCL Queen Square Institute of Neurology, London, WC1N 3BG, UK; ^2^Department of Clinical and Movement Neurosciences, UCL Queen Square Institute of Neurology, London, WC1N 3BG, UK; ^3^Victor Horsley Department of Neurosurgery, National Hospital for Neurology and Neurosurgery, Queen Square, London, WC1N 3BG, UK

##### **Correspondence**: Matthew J Bancroft, matthew.bancroft.13@ucl.ac.uk

*Fluids and Barriers of the CNS* 2023, **20(Suppl 2)**: A91

**Introduction**: Patients with ICP disturbances often report worsening symptoms upon active movement, such as walking. However, the effect of movement on ICP has received little attention, and instead is typically studied whilst a patient is stationary. We recently reported that ICP is modulated systematically over the stepping cycle when walking. Here we investigate potential explanations of the effect of walking on ICP.

**Methods**: Ambulatory patients undergoing routine ICP monitoring (n = 27) stood upright and still for 2 min, then walked back and forth along the hospital ward at a self-selected pace for 2 min, followed by another 2 min of standing. A subset of patients (n = 4) completed two further recordings where they walked faster and slower than their self-selected pace. ICP was measured by intraparenchymal probe (Raumedic Neurovent-P). Body kinematics were measured from inertial measurement units attached to the head, chest, pelvis and bilateral feet, upper and lower legs. ICP and kinematic data were both recorded at 100Hz and synchronised precisely.

**Results**: The classically-shaped, cardiac cycle-driven ICP waveform observed during standing was immediately abolished upon initiation of walking and replaced by an ICP waveform with greater mean, and oscillations with larger amplitude and higher frequency than when standing. ICP oscillations during walking were tightly linked to body kinematics such that their frequency corresponded to the stepping rate and their magnitude corresponded to the degree of vertical head motion. The temporal lag between head motion and ICP oscillation was small ( < 25 ms on average) further suggesting a tight relationship. On average, vertical head displacement and acceleration each explained ~60% of variation in the magnitude of ICP oscillation. Both step rate and vertical head motion increased with walking speed which subsequently increased the frequency and magnitude of ICP oscillations, respectively.

**Conclusions**: We demonstrate that ICP oscillations during walking are locked to head motion.

## A92 Cut-off point of the noninvasive measurement of intracranial compliance for the diagnosis of idiopathic Normal Pressure Hydrocephalus

### Gabriel A. S. Mendes^1,2^, Raphael Bertani^2^, Cintya Hayashi^2^, Gustavo H. Frigieri³, Rodolfo C. Reis^4^, Manoel J. Teixeira^2^, Fernando C. G. Pinto^2^

#### ^1^Physiotherapy Nucleous, Hospital of the State Public Servant of São Paulo, São Paulo, SP, Brazil; ^2^Cerebral Hydrodynamics Group, Department of Neurosurgery, Hospital das Clínicas, University of São Paulo, SP, Brazil; ^3^Brain4Care, São Carlos, SP, Brazil; ^4^Neurosurgery Department, Hospital of the State Public Servant of São Paulo, São Paulo, SP, Brazil.

##### **Correspondence**: Gabriel André da Silva Mendes, mendes1986@yahoo.com.br

*Fluids and Barriers of the CNS* 2023, **20(Suppl 2)**: A92

**Introduction**: Normal Pressure Hydrocephalus (NPH) is a disease directly related to changes in intracranial compliance. Non-invasive measurement of the intracranial pressure curve may provide a new reliable way of assessing brain compliance and reduce costs with imaging tests and invasive procedures. The present study proposes to evaluate the performance of the P2/P1 ratio (provided through the non-invasive device from the company Brain4care®) as a diagnostic criterion and define a cutoff point for NPH.

**Methods**: Twenty-five patients were evaluated and submitted to TAP tests. Non-invasive evaluation of intracranial compliance was performed before and after puncture of 50mL of CSF. All evaluations were performed in the lying position. The performance of P2/P1 was evaluated taking into account the timing (pre- and post-puncture), in addition to the evaluation of the cut-off points from 0.8 to 1.2.

**Results**: The results obtained show that pre-puncture, a P2/P1 ratio of 1.1 is the best cut-off point, with a sensitivity of 83%, a sensitivity of 71%, an accuracy of 80%, a positive predictive value of 88%, and a negative predictive value of 63%.

**Conclusions**: A value of 1.1 with the patient lying down before the puncture is the appropriate cut-off point for the variable P2/P1 ratio in the noninvasive assessment of intracranial compliance.

## A93 Prediction of optimal Intracranial Pressure (ICP) level to achieve normalization of pulse amplitude

### Leon Gramss^1,2^, Linda D’Antona^1,2^, Eleanor Moncour^1,2^, Kanza Tariq^1,2^, Lucia Darie^1^, Lewis Thorne^1^, Laurence Watkins^1,2^, Ahmed Toma^1,2^

#### ^1^Victor Horsley Department of Neurosurgery, National Hospital for Neurology and Neurosurgery, London, WC1N3BG, UK; ^2^Queen Square Institute of Neurology, University College London, London, WC1N3BG, UK

##### **Correspondence**: Linda D’Antona, linda.d’antona@nhs.net

*Fluids and Barriers of the CNS* 2023, **20(Suppl 2)**: A93

**Introduction**: Shunt setting adjustments are based on patients’ signs and symptoms, and/or intracranial pressure (ICP) readings. Pulse amplitude (PA) is a useful marker of brain compliance. Valve setting adjustments usually aim at the normalization ICP, but this does not necessarily correspond to normal PA. It would be useful to be able to predict the level of ICP that results in normalization of PA. This pilot study describes the prediction of PA from ICP values in adult patients undergoing elective 24-h intraparenchymal ICP monitoring.

**Methods**: Single-centre retrospective observational study. Patients undergoing valve setting adjustments guided by 24-h intraparenchymal ICP monitoring were included. Patients with suspected shunt blockage were excluded. For each patient, the minute-by-minute ICP and PA measurements taken in the first 24 h (‘baseline’ dataset) were used to build linear regression models to predict PA from ICP. This model was then tested against the 24-h ICP monitoring recording performed after valve setting adjustment (‘test’ dataset) and used to calculate a ‘predicted PA’. Differences between ‘predicted PA’ and actual PA measurements are described.

**Results**: Seven patients (6F, 1M, mean age 37±11 years) were selected. They had heterogenous diagnoses and shunt types. The ‘baseline’ minute-by-minute data included a total of 16114 observations (average 2302 per patient), the ‘test’ minute-by-minute data included 19546 observations (average 2792 per patient). For all the patients, the linear regression models demonstrated a significant association between PA (dependent variable) and ICP (independent variable) (p < 0.05). The difference between ‘predicted PA’ and actual PA measurements was negligible (range 0.05–0.68 mmHg, mean 0.02). The optimal mean ICP, achieving PA < 5 mmHg was 6±3 mmHg but varied among patients (ranging 0–10 mmHg).

**Conclusions**: It is possible to predict optimal ICP levels that would achieve normalization of PA, this is potentially useful when planning valve setting adjustments. Larger studies will be needed to confirm these findings.

## A94 Characteristics of the M.scio Telemetric ICP Measurement System with Lumbar Catheter

### Anders Eklund^1^, Tomas Bäcklund^1^, Michael A. Williams^2^, Jan Malm^3^

#### ^1^Department of Radiation Sciences, Biomedical Engineering, Umeå University, Umeå, Sweden; ^2^University of Washington, Seattle, USA; ^3^Department of Clinical Science, Neurosciences, Umeå University, Umeå, Sweden

##### **Correspondence**: Anders Eklund, anders.eklund@umu.se

*Fluids and Barriers of the CNS* 2023, **20(Suppl 2)**: A94

**Introduction**: Astronauts in microgravity develop symptoms that raise suspicion of elevated ICP. One option to investigate this is to surgically implant a lumbar catheter attached to a Telemetric Intracranial Pressure Monitoring (M.scio, Miethke) in astronauts and measure ICP. We evaluated the capability of this assembly to detect mean ICP and ICP pulsatility.

**Methods**: We bench tested six M.scio sensor and Medtronic lumbar catheters for sinusoidal frequency response, including the effect of different catheter lengths, including evaluation with an ICP-waveform signal, and accuracy of measuring static mean pressure.

**Results**: The variable frequency measurements showed that pressure amplitude dampening increased with both frequency and catheter length. With a 44 cm catheter, the amplitude transfer drops from 79% to 20% when going from 1 to 5 Hz. The corresponding change for an 84 cm catheter was from 59% to 13%. The dampening was not affected by the mean pressure. The detected amplitude for a 3 mmHg ICP waveform was reduced by 28% with heart rate increase from 56 to 112 bpm. Static measurements showed that the within-sensor response to changes from − 5 to 20 mmHg was, on average, 92% of the reference change and the bias was − 2.0 ± 1.1 mmHg at zero ref pressure. The M.Scio system had a noise level of SD = 0.4 mmHg, which affects the amplitude assessment.

**Conclusions**: A lumbar approach with the M.scio for investigating astronaut CSF dynamic changes in microgravity is feasible, but considering the system characteristics, the ICP could be underestimated. Our findings suggest that one should use the shortest possible catheter, apply post processing to reduce the effect of noise, and adjust for changes in HR.

Supported by the Baylor College of Medicine Center for Space Medicine through a gift from the Polaris Dawn Program.

## A95 Early feasibility study of implantation of a telemetric Intracranial Pressure (ICP) sensor in a commercial spaceflight participant

### Michael A. Williams^1^, Karen N. Adams^1^, Elisa McGee^1^, Robert Bonow^1^, Andrew Ko^1^, Danial Hallam^1^, Courtney Gomez^1^, Jan Malm^2^, Anders Eklund^2^, Tomas Bäcklund^2^, J. E. Schmitt^3^, Eric M. Bershad^4^, Christoph Miethke^5^, Andreas Bunge^5^

#### ^1^University of Washington, Seattle, USA; ^2^University of Umeå, Sweden; ^3^University of Pennsylvania, Philadelphia, USA; ^4^Baylor College of Medicine, Houston, USA; ^5^Christoph Miethke GmbH & Co. KG, Potsdam, Germany

##### **Correspondence**: Michael A. Williams, maw99@uw.edu

*Fluids and Barriers of the CNS* 2023, **20(Suppl 2)**: A95

**Introduction**: Spaceflight Associated Neuro-ocular Syndrome (SANS) may be caused by abnormal ICP. Non-invasive ICP methods researched by NASA lack the accuracy to make this determination. Lumbar puncture is considered unsafe for spaceflight. Methods to assess ICP directly during spaceflight are needed.

**Methods**: To obtain U.S. Food and Drug Administration (FDA) approval to surgically implant a telemetric ICP sensor (Miethke M.scio) attached to a lumbar catheter to evaluate its safety and effectiveness for ICP monitoring in a commercial spaceflight participant (SFP), we: 1) designed the surgical and research protocol and safety evaluations for high G forces; 2) submitted an FDA Investigational Device Exemption (IDE) application; 3) collaborated with Miethke GmbH and SpaceX to test the equipment for spaceflight conditions; and 4) conducted bench testing of the implant assembly.

**Results**:

- April 18, 2022 – FDA pre‐application submitted
- July 5, 2022 – Teleconference with FDA reviewers
- August 19, 2022 – Teleconference with FDA/NASA liaisons, and TRISH and NASA leaders
- October 24, 2022 – Teleconference with FDA reviewers
- November 2022 –M.scio system preclinical quality testing by Miethke GmbH
- January 3, 2023 – IDE early feasibility study and research protocol with safety assessments submitted
- February 14, 2023 – IDE approved by FDA
- March 2023 – Bench testing of M.scio implant assembly by Umeå
- March 16, 2023 – Baylor College of Medicine IRB approved the protocol
- May 1, 2023 – Awaiting spaceflight opportunity

**Conclusions**: If the research protocol proceeds as planned, it will be the first-ever direct ICP recording in an SFP before, during, and after spaceflight, yielding significant insight into the role of ICP and craniospinal compliance in the early development of SANS.

Supported by the Translational Research Institute for Space Health through NASA Cooperative Agreement NNX16AO69A.

We wish to thank our SpaceX collaborators Marissa Rosenberg, Amran Asadi, and Jaime Mateus

## A96 Effect of shunt adjustment on short term telemetric Intracranial Pressure (ICP) readings

### Karina Hoffmann^1^, Linda D’Antona^1,2^, Zeid Abussuud^2^, Eleanor Moncour^1,2^, Kanza Tariq^1,2^, Lucia Darie^2^, Lewis Thorne^2^, Laurence Watkins^1,2^, Ahmed Toma^1,2^

#### ^1^Queen Square Institute of Neurology, University College London, London, WC1N3BG, UK; ^2^Victor Horsley Department of Neurosurgery, National Hospital for Neurology and Neurosurgery, London, WC1N3BG, UK

##### **Correspondence**: Linda D’Antona, linda.d’antona@nhs.net

*Fluids and Barriers of the CNS* 2023, **20(Suppl 2)**: A96

**Introduction**: The advent of telemetric intracranial pressure (ICP) reading shunt components (M.Scio, Miethke), has improved the management of patients with cerebrospinal fluid (CSF) dynamic disturbances, creating the opportunity of frequent non-invasive ICP measurements. These measurements are typically short in time (seconds to few minutes) and are often used to guide shunt adjustments. The effect of shunt adjustments on ICP in the short term is unclear, as the utility of performing immediate post-adjustment ICP measurements. If the human craniospinal compartment behaves as a simple fluid filled compartment, the ICP should be expected to reduce within 2 min of a valve setting reduction (tested hypothesis). This ongoing pilot study describes the effect of valve adjustments on post-adjustment telemetric ICP readings.

**Methods**: Prospective, single-centre observational study. Patients with a shunt incorporating M.Scio telemetric reader who underwent shunt setting adjustment since March 2023 were included. Patients with suspected shunt blockage were excluded. Baseline (pre-adjustment) and post-adjustment (at various time intervals) ICP was recorded for 30 s in sitting, standing and supine position. Mann-Whitney U test was used to compare ICP results.

**Results**: Five patients (3F, 2M, mean age 67±17 years) with different CSF dynamics disorders were included. All patients underwent downwards valve setting changes. The mean baseline ICP was − 11.1 mmHg (4.4SD) in sitting, − 8.3 mmHg (5.5SD) in standing and 18.4 mmHg (7.5SD) in supine position. The mean post-adjustment ICP was − 10.5 mmHg (5.6SD) in sitting, − 9.5 mmHg (5.9SD) in standing and 15.0 mmHg (6.5SD) in supine position. There was no statistically significant difference comparing baseline and post-adjustment ICPs (p > 0.05 for all 3 positions, Mann-Whitney U). Differences in ICP at later post-adjustment time intervals will be discussed.

**Conclusions**: The initial results of this ongoing study showed that ICP does not significantly change 2 min after shunt adjustment. These results suggest that CSF dynamics are much more complex than those of a simple fluid filled container.

## A97 Age-stratified presentation and outcomes of ventriculoperitoneal shunt implantation in a cohort of normal pressure hydrocephalus (NPH) patients

### Aleksandra B. Lasica^1^, Zeid A. Abussuud^1^, Christos Soumpasis^1^, Astri M. V. Luoma^2^, Ahmed K. Toma^1^

#### ^1^Victor Horsley Department of Neurosurgery, National Hospital for Neurology and Neurosurgery, University College London Hospitals, NHS Foundation Trust, Queen Square, London, UK; ^2^Department of Neuroanaesthesia and Neurocritical Care, National Hospital for Neurology and Neurosurgery, University College London Hospitals, NHS Foundation Trust, Queen Square, London, UK

##### **Correspondence**: Alexandra B. Lasica, a.lasica@nhs.net , Zeid A. Abussuud, z.abussuud@nhs.net

*Fluids and Barriers of the CNS* 2023, **20(Suppl 2)**: A97

**Introduction**: NPH is a disorder of cerebrospinal fluid dynamics. The condition is partially reversed by ventriculoperitoneal shunt (VPS) implantation. The majority of patients affected are above the age of 65. NPH patients are often affected by multiple comorbidities; increasing the surgical risk in this population. This study investigates the effects of advanced age on the presentation of NPH and post-operative VPS outcomes by comparing individuals over 80 and below 80 years of age.

**Methods**: Patients admitted to the National Hospital for Neurology and Neurosurgery (NHNN), London to undergo VPS implantation for NPH between April 2019–2022 were screened. We recorded standard demographics, symptoms at presentation, pre-operative co-morbidities, medication burden and frailty and co-morbidity markers. The admission length, early post-operative morbidity, VPS mobility outcomes and VPS revision rates were recorded. Pearson’s chi-square test and point-biserial correlations were employed to assess the associations between age and investigated factors.

**Results**: 126 NPH patients, 89 (70.6%) below 80 years and 37 (29.4%) above 80 years were included. There were no significant differences in symptoms duration, symptoms at presentation, comorbidities and medication burden between the two groups. Patients above 80 years of age had higher baseline frailty levels and greater surgical risk parameters as reflected by various anaesthetic, surgical outcome and frailty scores. The older group had post-VPS mobility outcomes comparable to the younger group, with 88.9% and 85.7% improvement rates, respectively (*p* = *1.000*). No significant differences in the immediate post-operative morbidity, post-operative hospital stay (*p* = *0.512*) or shunt revision rates (*p* = *0.279*) were demonstrated. Older NPH patients received more input from the occupational and physiotherapy teams post-operatively (*p* = *0.038*).

**Conclusion**: Age alone should not be a factor precluding elderly NPH patients from surgical intervention. Incorporation of comprehensive anaesthetic assessment, adequate pre-operative optimisation and post-operative therapy input should be promoted to maximise the benefits of VPS for older adults.

## A98 Effective shunt surgery for elderly patients after a fall with definite idiopathic normal pressure hydrocephalus can prevent the following fall

### Ryo Oike, Yasuaki Inoue

#### ^1^Department of Neurosurgery, Nadogaya Hospital, Kashiwa, Japan

##### **Correspondence**: Ryo Oike, ryo.st2b@gmail.com

*Fluids and Barriers of the CNS* 2023, **20(Suppl 2)**: A98

**Introduction**: We previously reported the benefit of screening for idiopathic normal pressure hydrocephalus (iNPH) in older adults after falls. We assessed whether the shunt surgeries could prevent another fall in those iNPH patients postoperatively.

**Methods**: We retrospectively evaluated the consecutive cases diagnosed with probable iNPH after falls to eventually get a shunt surgery between May 2020 and January 2022. We included the patients with the modified Rankin scale (mRS) score of 0–3 1 year after the shunt surgery. Patients who dropped out from our postoperative follow-up were excluded. We compared the time of the Timed-Up Go test (TUG), the Mini-Mental State Evaluation (MMSE), the utility weighted-mRS (UW-mRS), the iNPH grading scale, and the history of postoperative falls between the shunt responders and the non-responders 1 year after the surgery. We defined shunt responders 1 year after the surgery as patients improved in mRS score by at least 1.

**Results**: Fifty-one patients were included; 40 shunt responders and 11 non-responders. The number of patients who had never fallen up to 1 year after the surgery was statistically more significant in the shunt responders than in the non-responders (33 patients [82.5%] vs. 5 [45.5%]; 95% confidence interval [CI], p = 0.0273). There was no statistically significant difference except for the preoperative UW-mRS (Median [Interquartile range], 6.5 [3.3–7.6] vs. 7.6 [6.5–9.1]; 95% CI, p = 0.021).

**Conclusions**: Shunt surgeries for elderly patients with definite iNPH after falls can prevent the following fall.

## A99 Comparison of female and male outcomes in a cohort of normal pressure hydrocephalus (NPH) patients undergoing ventriculoperitoneal shunt implantation

### Aleksandra B. Lasica^1^, Zeid A. Abussuud^1^, Christos Soumpasis^1^, Astri M. V. Luoma^2^, Ahmed K. Toma^1^

#### ^1^Victor Horsley Department of Neurosurgery, National Hospital for Neurology and Neurosurgery, University College London Hospitals, NHS Foundation Trust, Queen Square, London, UK; ^2^Department of Neuroanaesthesia and Neurocritical Care, National Hospital for Neurology and Neurosurgery, University College London Hospitals, NHS Foundation Trust, Queen Square, London, UK

##### **Correspondence**: Alexandra B. Lasica, a.lasica@nhs.net, Zeid A. Abussuud, z.abussuud@nhs.net

*Fluids and Barriers of the CNS* 2023, **20(Suppl 2)**: A99

**Introduction**: NPH is a disease of the elderly classically characterized by a triad of gait disturbance, cognitive impairment and urinary symptoms. Symptoms can be partially reversed by ventriculoperitoneal shunt implantation (VPS). Little is known about the disparities between female and male NPH patients. This study aimed to characterise the differences between male and female NPH patients undergoing VPS implantation and their post-operative outcomes.

**Methods**: Patients admitted to the National Hospital for Neurology and Neurosurgery (NHNN), London to undergo VPS implantation for NPH between April 2019–2022 were screened. We recorded standard demographics, symptoms at presentation, pre-operative co-morbidities, medication burden and frailty and co-morbidity markers. Additionally, the admission length, early post-operative morbidity, VPS mobility outcomes and VPS revision rates were recorded. The associations between sex and investigated factors were assessed by Pearson’s chi-square test and point-biserial correlations.

**Results**: 126 NPH patients, 80 (63.5%) males and 46 (36.5%) females were included. Gait disturbance and urinary symptoms were more common among females than males (*p* = *0.038*). Gait disturbance and cognitive impairment were more prevalent among males (*p* = *0.021*). Females experienced a greater burden of mental health illness compared to males (*p* = *0.073*). No significant differences in the age of presentation, symptom duration, co-morbidities, medication burden, multiple frailty markers, VPS outcomes or revision rates were found. Females reported severe post-operative pain more than males did ) (*p* < *0.001*) which may have accounted for a longer post-operative stay in females (4.9 versus 3.7 days; *p* = *0.102*).

**Conclusion**: Symptomatic differences between sexes and the routine use of objective assessments of cognition, urinary symptoms, and gait disturbance should be considered during the diagnostic process. This may facilitate early NPH treatment and, thus, better outcomes. Additionally, limited data on female and male differences in the NPH cohort calls for further research, including immediate and long-term shunting outcomes.

## A100 Baseline PROMIS Score Characteristics and Temporal Changes Following Shunt Placement in Patients with Idiopathic Normal Pressure Hydrocephalus (iNPH)

### Abdelrahman Hamouda^1^, Mahnoor Shafi^1^, Zach Pennington^1^, Hannah Hallak^1^, Maria Astudillo^1^, Jonathan Graff-Radford^2^, David T. Jones^2^, Hugo Botha^2^, Jeremy K. Cutsforth-Gregory^2^, Petrice M. Cogswell^3^, Benjamin D. Elder^1^

#### ^1^Department of Neurologic Surgery, Mayo Clinic, Rochester, MN, 55905, USA; ^2^Department of Neurology; ^3^Department of Radiology

##### **Correspondence**: Benjamin Elder, MD/PhD, elder.benjamin@mayo.edu

*Fluids and Barriers of the CNS* 2023, **20(Suppl 2)**: A100

**Introduction**: Patient reported-outcomes Measurement information system (PROMIS) is currently a widely used tool to provide an overview of baseline quality of life characteristics. The objective was to describe the baseline PROMIS scores in idiopathic Normal Pressure Hydrocephalus patients, and to determine the feasibility of PROMIS to document improved quality of life in iNPH patients.

**Methods**: 222 patients > 60 years of age were diagnosed with iNPH and underwent shunt placement from 2018 to 2022, were retrospectively reviewed. PROMIS questionnaires were administered preoperatively and during short- and long-term clinic visits. We primarily evaluated seven PROMIS computerized adaptive testing (CAT) domains including anxiety, depression, fatigue, sleep, pain interference, social ability, and physical function. Additionally, we evaluated four secondary PROMIS domains including upper extremity function CAT, short form pain intensity, and short form global and mental health.

**Results**: A total of 174 patients completed the main seven PROMIS CAT domains before shunt placement. The average baseline scores were: anxiety (55.85), depression (52.82), fatigue (57.41), sleep (49.15), pain interference (55.51), social ability (42.92) and physical function (33.54). 90 patients completed the PROMIS questionnaire at mean 20 months postoperatively. There was a significant decrease in the mean score for anxiety (p < 0.007), and fatigue (p < 0.001), and a significant increase in the mean score for social domain (p < 0.001) and physical function (p < 0.001). However, no significant differences were observed following shunt placement for the other PROMIS CATS domains.

**Conclusions**: The data indicate that shunt placement results in significant improvement in patient reported outcomes for depression, fatigue, social ability, and physical function, and indicates that these PROMIS CAT domains may be useful in the iNPH population.

## A101 Long-term Cognitive and Gait Outcomes after Primary Endoscopic Third Ventriculostomy (ETV) in Adults from the AHCRN with Chronic Obstructive Hydrocephalus

### Thomas J. Zwimpfer^1^, Richard Holubkov^2^, Heather Katzen^3^, Mark G. Luciano^4^, Sean J. Nagel^5^, Michael A. Williams^6^, Jeffrey H. Wisoff^7^, Guy M. McKhann^8^, Richard Edwards^9^, Abhay Moghekar^10^, Nickolas Dasher^11^, Mark G. Hamilton^12^

#### ^1^Department of Surgery, University of British Columbia, Vancouver, V5Z 1M9, Canada; ^2^Department of Pediatrics, University of Utah School of Medicine, Salt Lake City, USA; ^3^Department of Neurology, University of Miami Miller School of Medicine, USA; ^4^Department of Neurosurgery, Johns Hopkins University, Baltimore, USA; ^5^Department of Neurosurgery, Center for Neurological Restoration, Cleveland Clinic, USA; ^6^Departments of Neurology and Neurological Surgery, University of Washington School of Medicine, Seattle, USA; ^7^Department of Neurosurgery, New York University School of Medicine, USA; ^8^Department of Neurological Surgery, Columbia University Irving Medical Center, New York, USA; ^9^Department of Neurosurgery, Southmead Hospital, Bristol, United Kingdom; ^10^Department of Neurology, Johns Hopkins University, Baltimore, USA; ^11^Department of Rehabilitation Medicine, University of Washington School of Medicine, Seattle, USA; ^12^Department of Clinical Neurosciences, Division of Neurosurgery, University of Calgary Cumming School of Medicine, Canada

##### **Correspondence**: Thomas J. Zwimpfer, thomas.zwimpfer@ubc.ca

*Fluids and Barriers of the CNS* 2023, **20(Suppl 2)**: A101

**Introduction**: This study investigated the long-term efficacy of primary ETV on cognition and gait in adults with chronic obstructive hydrocephalus.

**Methods**: Patients from the multicenter Adult Hydrocephalus Clinical Research Network (AHCRN) registry, undergoing ETV for untreated congenital or acquired obstructive hydrocephalus, were prospectively accrued. Outcomes were gait velocity (10-meter walk test) and Montreal Cognitive assessment (MoCA) score. Median within-patient change, from pre-ETV to post-ETV evaluation, was assessed (via Signed Rank Test).

**Results**: 61 patients with both pre- and post-ETV assessments were analyzed. Mean age was 58.1 years and 34 (55.7%) were female. Etiology of CSF obstruction was either congenital (n = 37, 60.7%) or acquired (n = 24, 39.3%). Pre- and ≤ 6-month post-ETV gait was assessed in 43 patients: Baseline median gait velocity was 0.9 m/s, improving to 1.3 m/s at ≤ 6-mo post-ETV. This improvement was statistically significant with median within-patient change of + 0.3 m/s (*p* < 0.001). Gait was assessed ≥ 12-month post ETV (12–51 months) in 21 of 43 patients (49%) and improvement persisted: Baseline median gait velocity of 1.1 m/s, improved to 1.4 m/s at ≥ 12-mo post-ETV and the median within-patient change was + 0.2 m/s (*p* < 0.001). Pre- and ≤ 6-month post-ETV MoCA was conducted in 46 patients: Baseline median MoCA was 24/30, which improved to 26/30. This improvement was statistically significant with the median within-patient MoCA change of + 1 (*p* = 0.002). MoCA was performed ≥ 12-month post-ETV (12 to 50 months) in 22 of 46 patients (48%) and improvement persisted: Baseline median MoCA was 23/30, which improved to 25/30 at ≥ 12-month post-ETV, while the median within-patient MoCA change was + 1 (*p* = 0.03). However, a ≥ + 2 change in MoCA is probably necessary to be clinically meaningful.

**Conclusion**: ETV results in long-term improvement in gait and cognition in adults with chronic obstructive hydrocephalus.

## A102 Safety, sequelae, and efficacy of nerve root clipping in patients with spontaneous spinal CSF leaks

### A. El Rahal, B. Haupt, F, Volz, K. Wolf, B. Blass, L. M. Kraus, I, Vassilikos, N, Lützen, L. Häni, J. Beck, C. Fung

#### ^1^Medical Center University of Freiburg, Freiburg, Germany

##### **Correspondence**: Amir El Rahal, amir.elrahal@uniklinik-freiburg.de

*Fluids and Barriers of the CNS* 2023, **20(Suppl 2)**: A102

**Introduction**: Spinal CSF leaks may cause a myriad of clinical symptoms, the most common being orthostatic headache. Leaking cysts (Type II) and direct CSF venous fistulas (Type III) are a subgroup of spinal CSF leaks representing about 1/3 of spinal CSF leaks. To seal the diverticula or the CSF venous fistula the respective nerve root is microsurgically ligated using aneurysm clips.

**Objectives**: The study aimed to analyze the incidence of sensory-motor deficits, neuropathic pain, and patient-reported outcome after nerve root clipping.

**Methods**: All consecutive patients with Type II and Type III spinal CSF leaks treated with nerve root clipping at our Neurosurgical institution from May 2018 to November 2022 were included. Patients were evaluated for post-operative sensory-motor deficits. The incidence of neuropathic pain and patient-reported outcome was assessed via DN4 (Douleurs Neuropathique 4) and PGIC (Patients' Global Impression of Change), respectively.

**Results**: A total of 40 patients were included in the study (Type II n = 31; Type III n = 9). In total, 47 spinal CSF leaks were treated via nerve root clipping; the mean age was 46,6. The mean follow-up time was 22 months. Over 80% of patients reported significant recovery. Almost 90% of patients either fully or partially returned to their employment. One patient clipped in the upper thoracic region developed a regressive motor deficit. The incidence of neuropathic pain was 7,5%. Over 80% of patients developed dermatomal hypoesthesia, with no reported effect on life quality.

**Conclusion**: Nerve root clipping is a safe and effective surgical strategy for patients with Type II and Type III spinal CSF leaks. Only 7.5% of patients developed post-operative neuropathic pain, and only 2.5% needed medical treatment. In terms of efficacy, over 98% of patients reported recovery, according to the PGIC score and close to 90% of patients either fully or partially returned to their employment.

## A103 Prognostic Significance of Cerebrospinal Fluid Production Rate in the Management of Cerebrospinal Fluid Leak

### Kanza Tariq, Ahmed Toma, Sogha Khawari, Lewis Thorne, Laurence Watkins

#### ^1^National Hospital for Neurology and Neurosurgery, Queen Square, London, UK

##### **Correspondence**: Kanza Tariq kanza.tariq@nhs.net

*Fluids and Barriers of the CNS* 2023, **20(Suppl 2)**: A103

**Introduction**: The management of cerebrospinal fluid (CSF) leak is quite varied. Traditionally, CSF leaks are managed by primary repair (with or without augmentation), tissue glue, and CSF diversion (most commonly lumbar drain placement). Our study aimed to demonstrate net cerebrospinal fluid production rate (PRcsf) as a potential prognostic tool for the management of CSF-leaks.

**Methods**: A prospective observational study was performed on all CSF leak patients in our hospital who required CSF drainage as part of their ongoing management. The drain was connected to a LiquoGuard7 (Möller-Medical, Germany) with the intracranial pressure sensor at the level of the external auditory meatus. The internal software and flow-rate data of the LiquoGuard7 pump was used to calculate net PRcsf in the patients. Patients were supine for 30 min during the measurement. Patient notes and electronic health records were reviewed daily. The events occurring during the hospital stay for each patient and the treatment received was noted. Patients were followed-up at 6 months post-hospital discharge. Prognosis and patient outcome were recorded as complete resolution of symptoms at 6 months post-hospital discharge. Statistical analysis used SPSS (version 25.0, IBM) by multivariate multiple regression, comparing the resolution of symptoms to the measured PRcsf, age, gender, co-morbidities, and the type of surgical treatment received.

**Results**: 26 CSF leak patients were studied. CSF leak patients with average PRcsf of 55ml/h ± 10SD (n = 9) improved with lumbar drain (average 7-days, average age < 40 years). CSF Leak patients demonstrating average PRcsf of 86ml/h ± 10SD (n = 10) improved following CSF-shunting with no further CSF leak (average age 48 years). Repair ± augmentation ± CSF-shunt was required in patients with PRcsf of 146ml/h ± 5SD (n = 7, average age 52years). All CSF-leak patients on average demonstrated high intracranial-pressure.

**Conclusion**: PRcsf may hold prognostic value towards treatment-response in patients with CSF-leak.

## A104 Surgical closure of spinal CSF leaks improves symptoms in patients with superficial siderosis

### Amir El Rahal^1,2^, Benedikt Haupt^1^, Christian Fung^1^, Debora Cipriani^1^, Levin Häni^1^ , Niklas Lützen^1^, Tomas Dobrocky^2^, Eike Piechowiak^2^, Oliver Schnell^1^, Andreas Raabe^3^, Katharina Wolf^1^, Horst Urbach^1^, Luisa Mona Kraus^1^, Florian Volz^1^, Jürgen Beck^1^

#### ^1^Medical Center University of Freiburg, Freiburg, Germany; ^2^Inselspital Bern, Bern, Switzerland

##### **Correspondence**: Amir El Rahal amir.elrahal@uniklinik-freiburg.de

*Fluids and Barriers of the CNS* 2023, **20(Suppl 2)**: A104

**Background**: Spinal CSF leaks may cause a myriad of clinical symptoms, the most common being orthostatic headache. In addition, ventral spinal CSF leaks are a possible etiology of superficial siderosis (SS), a rare condition characterised by hemosiderin deposits in the CNS. It is caused by chronic or repeated subarachnoid bleeding. It classically presents as progressive cerebellar ataxia, bilateral hearing loss, and myelopathy, for which effective therapy is limited, and symptoms are considered irreversible.

**Objective**: To evaluate if microsurgical closure of the CSF leak can prevent further clinical deterioration or even improve symptoms of superficial siderosis.

**Methods**: This cohort study was conducted using data from a prospectively maintained database in 2 large SIH referral centers in Germany and Switzerland of patients who met the modified International Classification of Headache Disorders, 3rd edition criteria for SIH. Patients with spinal CSF leaks were screened for the presence of idiopathic infratentorial symmetric superficial siderosis of the CNS.

**Results**: In total, 12 patients with a ventral spinal CSF leak and SS were identified. The median latency between the onset of orthostatic headaches, if present, and symptoms attributed to superficial siderosis was 9.5 years. After surgical closure of the underlying spinal CSF leak, symptoms attributed to superficial siderosis improved in 70% and remained stable in 30%. Patients who presented within 1 year after the onset of superficial siderosis symptoms improved, but those who presented within 8–12 years did not improve at the follow-up time.

**Conclusion**: We conclude that long-standing untreated ventral spinal CSF leaks can lead to superficial siderosis of the CNS and that microsurgical sealing of spinal CSF leaks might stop progression and improve symptoms in patients with superficial siderosis in a time-dependent manner.

## A105 Recovery and long-term outcome after neurosurgical closure of spinal CSF leaks in patients with spontaneous intracranial hypotension (SIH)

### Florian Volz^1^ Christian Fung^1^, Katharina Wolf^1^, Niklas Lützen^2^, Horst Urbach^2^, Luisa Mona Kraus^1^, Mazin Omer^1^, Jürgen Beck^1^, Amir El Rahal^1,3^

#### ^1^Department of Neurosurgery, Medical Center University of Freiburg, Freiburg im Breisgau, Germany; ^2^Department of Neuroradiology, Medical Center University of Freiburg, Freiburg im Breisgau, Germany; ^3^Faculty of Medicine, University of Geneva, Geneva, Switzerland

##### **Correspondence**: Amir El Rahal amir.elrahal@uniklinik-freiburg.de

*Fluids and Barriers of the CNS* 2023, **20(Suppl 2)**: A105

**Introduction**: Spontaneous intracranial hypotension (SIH), caused by a spinal CSF leak, may cause many clinical symptoms, the most common being disabling headaches with a high impact on patients’ quality of life. While successful closure of the leak is often reported, long-term outcome results regarding patients’ symptoms are still scarce. This study aimed to analyze the postoperative course and long-term outcome in patients treated surgically for a spinal CSF leak.

**Methods**: Using patient reported outcome measures (PROM), between April 2020 and December 2022, SIH patients treated surgically for a spinal CSF leak completed the HIT-6 questionnaire on a tablet before surgery, and post-operative at 14 days, 3, 6 and 12 months, respectively, via an automated follow-up system.

**Results**: In total, 80 patients were included. The median HIT-6 score preoperatively was 65, IQR (61–69) representing severely disabling headaches. The score improved to 49 (IQR 44–58) 3 months postoperatively and to 48 (IQR 40–56) 12 months postoperatively, considered to have little to no impact on patients’ quality of life.

**Conclusions**: Surgical closure of the CSF spinal leak in SIH patients significantly improves headaches in the long term. At least three months should be expected for recovery. PROMs are reliable and applicable in a daily surgical routine in order to assess clinical improvement. Despite these encouraging results, 1/4 of patients still have a relevant long-term impairment, indicating the need for further research on its cause and possible treatment.

## A106 Impact of Ventriculoperitoneal Shunt on Systemic Hypertension

### Kanza Tariq, Ahmed Toma, Lewis Thorne, Laurence Watkins

#### ^1^National Hospital for Neurology and Neurosurgery, Queen Square, London, UK

##### **Correspondence**: Kanza Tariq: kanza.tariq@nhs.net

*Fluids and Barriers of the CNS* 2023, **20(Suppl 2)**: A106

**Introduction**: Several studies have supported the hypothesis that rise in systemic hypertension (HTN) leads to a subsequent rise in intracranial pressure (ICP). Systemic hypertension is an established cause of secondary intracranial hypertension. Insertion of ventriculoperitoneal shunt (VPS) is a common neurosurgical intervention to remedy raised ICP. This single-centre study aimed to evaluate whether insertion of VPS led to a subsequent decrease in systemic blood pressure (BP) in known hypertensive patients.

**Methods**: A retrospective-observational study was performed in 100 patients who had undergone VPS insertion in the National Hospital for Neurology and Neurosurgery in 2019. The electronic-health-records (EHRs) of the patients were evaluated in depth. After recording demographic information (age, gender, underlying pathology, BMI), heed was paid to past medical history (PMH) of hypertension and drug history including various antihypertensive medication and the doses of these medications. EHRs were studied to note trends in BP measurements prior to and following VPS insertion, and the subsequent need for antihypertensive medication. Statistical analysis was done by SPSS (version 25.0, IBM) by paired t-test comparing BP measurements prior to and following VPS insertion.

**Results**: 44 patients undergoing VPS insertion had PMH of HTN (average systolic BP 150–200 mmHg) with drug history of antihypertensive agents. Of these 28 exhibited normotensive BP immediately following VPS insertion, which was maintained 3 years following VPS insertion. These patients did not require antihypertensive agents any further. On paired t-test this result was statistically very significant (p < 0.0001). 16 patients continued to suffer from hypertension following VPS insertion. 100% of these patients (16/16) demonstrated better control of their BP, with less frequent hypertensive episodes (average systolic BP 130–150 mmHg) and 25%(4/16) had a subsequent reduction in the dosage of antihypertensive medication. These results were maintained at 3 years following VPS insertion (p < 0.0001).

**Conclusion**: VPS insertion may lead to decrease in HTN, possibly by decrease in ICP. Validation of the results is required in larger cohorts.

## A107 Cognitive Improvement after Endoscopic Third Ventriculostomy Surgery in Long-standing Overt Ventriculomegaly in Adults

### Fabio Campanella^1^, Daniele Piccolo^1,2,3^, Giulia Sebastianutto^1,4^, Francesca Marotta^1,2^, Miran Skrap^1^, Francesco Tuniz^1^

#### ^1^Neurosurgery Unit, Udine University Hospital, Udine (UD), 33100, Italy; ^2^Department of Neuroscience, University of Padua, Padova (PD), 35121, Italy; ^3^Department of Clinical, Diagnostic and Pediatric Sciences, University of Pavia, Pavia (PV), 27100, Italy; ^4^Department of General Psychology, University of Padua, Padova (PD), 35131, Italy

##### **Correspondence**: Daniele Piccolo, ing.daniele@gmail.com

*Fluids and Barriers of the CNS* 2023, **20(Suppl 2)**: A107

**Introduction**: Long-standing overt ventriculomegaly in adults (LOVA) is a chronic form of hydrocephalus that may lead to cognitive decline. This study aimed to evaluate the cognitive outcomes of endoscopic third ventriculostomy (ETV) surgery in patients with LOVA hydrocephalus.

**Methods**: Twenty consecutive patients with LOVA hydrocephalus underwent ETV surgery, and their cognitive status was assessed before surgery, immediately after surgery, and at four months follow-up. Cognitive function was assessed using a neuropsychological battery that measured six cognitive domains: general cognitive status, attention/executive function, language, visuospatial ability, short-term memory, and long-term memory (LTM). Cognitive reserve was also measured using the Italian version of the National Adult Reading Test (NART).

**Results**: **L**TM was the only cognitive domain that was significantly impaired in patients with LOVA hydrocephalus, and immediate postoperative improvement was observed. The amount of immediate improvement in LTM was directly correlated with cognitive reserve, as measured by the NART. Improvement in LTM was maintained at the 4-month follow-up evaluation.

**Conclusions**: ETV surgery may lead to immediate improvement in LTM in patients with LOVA hydrocephalus. These findings suggest that ETV surgery may be an effective treatment for LOVA hydrocephalus, and that cognitive reserve may be an important factor in predicting outcomes after surgery. Further studies with larger sample sizes are needed to confirm these findings and to determine the long-term effects of ETV surgery on cognitive function in patients with LOVA hydrocephalus.

## A108 Estimation of ventricular volume changes for smart shunt systems

### Fabian Flürenbrock^1^, Leonie Korn^1^, Anthony Podgoršak^2^, Melanie Zeilinger^1^, Marianne Schmid Daners^1^

#### ^1^Institute for Dynamic Systems and Control, Department of Mechanical and Process Engineering, ETH Zurich, Switzerland; ^2^Product Development Group Zurich, Department of Mechanical and Process Engineering, ETH Zurich, Switzerland

##### **Correspondence**: Fabian Flürenbrock, ffluerenb@ethz.ch

*Fluids and Barriers of the CNS* 2023, **20(Suppl 2)**: A108

**Introduction**: Shunt therapy for hydrocephalus aims to regulate the volume of cerebrospinal fluid (CSF) within the cranial ventricles. Presently, however, monitoring focuses mainly on intracranial pressure (ICP) and actual CSF drainage rate is controlled by passive differential pressure valves. To narrow the gap between therapy objective and contemporary practice, a sensor technology for continuous estimation of changes in ventricular volume (VV) is developed.

**Methods**: A mechatronic test-bench that can simulate VV changes in a saline-filled silicone-carbon brain phantom was used to mimic CSF dynamics and brain tissue properties *in-vitro*. A measurement catheter with integrated electrodes was inserted into the ventricle. ICP and bioimpedance (BI) were measured simultaneously. A physiological test scenario, including respiratory and cardiac effects, with VV changes up to 9 mL was simulated and the recording split into training and testing data. Different process and measurement models were fitted to the training data and investigated to design Kalman filter (KF) that estimate the unknown VV changes in the testing data.

**Results**: Changes in ICP and VV are strongly positive correlated (r = 0.954), whereas changes in BI and VV are strongly negative correlated (r = -0.996). Affine functions suffice to approximate the dynamic changes of ICP and BI in the training data with a root-mean-squared-error (RMSE) of 0.423 mmHg and 0.057 Ω, respectively. A simple KF that uses a random walk process model and the affine functions as the measurement model can estimate VV changes in the testing data with an RMSE of 0.137 mL.

**Conclusions**: The introduced sensor technology demonstrates how multimodal sensor data can be fused to estimate VV changes that otherwise can only be observed via imaging. The *in-vitro* test result underlines its potential to improve hydrocephalus patient monitoring and to enable physiological control of CSF drainage for smart shunt systems that could reduce complications like over- and under-drainage.

## A109 Transcranial Direct Current Stimulation for Cognition Improvement in Postoperative Normal Pressure Hydrocephalus Patients with Programmable Pressure Valves: A Pilot Study

### Camila Santos Cechi^1^, Raphael Bertani^1^, Savio Batista^2^, Matheus Miranda^1^, Caio Perret^3^, Stefan Koester^4^, Tamires Guimarães Cavalcante Carlos de Carvalho^5^, Gabriel A. S. Mendes^1,6^, Fernando C. G. Pinto^1^

#### ^1^Cerebral Hydrodynamics Group, Department of Neurosurgery, Hospital das Clínicas, University of São Paulo, SP, Brazil; ^2^Medical School, Federal University of Rio de Janeiro (UFRJ), Rio de Janeiro, RJ, Brazil; ^3^Department of Neurosurgery, Hospital Municipal Miguel Couto, Rio de Janeiro, RJ, Brazil; ^4^Medical School, Universidade Nove de Julho (UNINOVE), São Paulo, SP, Brazil; ^5^Medical School, University of Vanderbilt, Nashville, TN, USA; ^6^Physiotherapy Nucleous,Hospital of the State Public Servant of São Paulo, São Paulo, SP, Brazil

##### **Correspondence**: Raphael Bertani, contato@rbertani.com

*Fluids and Barriers of the CNS* 2023, **20(Suppl 2)**: A109

**Introduction**: The aim of this study was to investigate the impact of transcranial direct current stimulation (tDCS) on cognition in late postoperative patients who had been treated with programmable pressure valves for Normal Pressure Hydrocephalus (NPH) for at least twelve months. tDCS is a non-invasive brain stimulation technique that uses low-intensity continuous current to modulate cortical excitability and influence cognitive functions.

**Methods**: Neuropsychological assessments were conducted to examine cognitive profiles of 5 (five) patients before and after tDCS sessions, . Three assessments were carried out: one before treatment initiation, one three days after the 15th tDCS session, and another 30 days after the last tDCS session. Fifteen tDCS sessions were administered over three consecutive weeks. Patients completed an Adverse Effects Scale after the first and fifth tDCS sessions, and a second neuropsychological assessment three days after the 15th session. Active or sham maintenance sessions were conducted four and six weeks after the second assessment, followed by a third neuropsychological evaluation 30 days after the last tDCS session. The primary outcome was assessed through post-intervention neuropsychological evaluations.

**Results**: The study employed a comprehensive battery of neuropsychological tests and scales to assess emotional aspects and quality of life. However, this abstract focused only on the Addenbrooke's Cognitive Examination (ACE), a brief cognitive assessment scale. The Kruskal-Wallis test and multiple Tukey comparisons were applied to the ACE scores at pre-treatment, three days post-treatment, and 30 days post-treatment.

**Conclusions**: Preliminary analysis of the ACE scores suggested a significant improvement in cognitive performance 30 days after tDCS treatment compared to pre-treatment scores. However, no significant differences were found between pre-treatment and three days post-treatment or between three days and 30 days post-treatment. Further research with larger samples and additional cognitive tests are warranted to confirm findings and explore the potential benefits of tDCS in such patients.

## A110 Shunt readmission rates and factors influencing them in hydrocephalus of all etiologies across the age spectrum in the Nationwide Readmissions Database

### Jenna Koschnitzky^1^, Monica Chau^2^, Yifan Zhang^3^, Abhay Moghekar^3^

#### ^1^Rhaeos, Inc., Chicago, IL, USA; ^2^Hydrocephalus Association, Bethesda, MD, USA; ^3^Department of Neurology, Johns Hopkins University School of Medicine, Baltimore, MD, 21231, USA

##### **Correspondence**: Abhay Moghekar, am@jhmi.edu

*Fluids and Barriers of the CNS* 2023, **20(Suppl 2)**: A110

**Introduction**: A significant portion of the healthcare burden in hydrocephalus is related to readmissions and reoperations. These rates have been described for multiple timeframes from 30 days to 20 years in single-site, multi-site, and population-based studies. However, only a few studies have analyzed population-based data across the entire age spectrum and none with data from the last 8 years.

**Methods**: A retrospective analysis based on data from the Agency for Healthcare Research and Quality (AHRQ) National Readmission Database (NRD) for the year 2019. The main outcomes analyzed were all-cause and shunt-related readmission rates at 30, 60, 90, and 180 days. Clinical and demographic variables described include hydrocephalus etiology, age, elective versus nonelective admission, sex, income level, readmission diagnosis, expected primary payer, hospital bed size, and hospital type.

**Results**: In 2019, for hospital admissions related to initial shunt placement and shunt revision, the 30-day all-cause readmission rate was 17.0% (16.2–17.8%) and the 30-day shunt-related readmission rate was 6.4% (5.8–7.0%). Both all-cause and shunt-related readmission rates were twice as high for non-elective admissions compared to elective admissions, 20.6% versus 10.9%, and 7.9% versus 3.8%, respectively (both p < 0.001). All-cause readmission rates were highest for admissions with a diagnosis of meningitis, tumor, or spina bifida w/hydrocephalus (27.3%, 23.3%, and 19.4%, respectively), and shunt-related readmission rates were highest for admissions with a diagnosis of spina bifida w/hydrocephalus and congenital hydrocephalus (both 10.5%). 10% of all shunts needed to be revised in the first 180 days after shunt surgery.

**Conclusions**: Readmissions related to shunt surgery continue to be a major burden on patients and the health care system despite advances in shunt technology. Hydrocephalus related to infections, neoplasms and spina-bifida are associated with higher readmission rates than other etiologies. Emergency shunt surgery irrespective of etiology continues to be the single most important determinant of readmissions.

## A111 Mechanical complications of Sophysa SM8 shunt in adult hydrocephalus: a monocentric experience

### Arrighi Marta, Berton Quentin, Coll Guillaume

#### ^1^Service de Neurochirurgie, CHU Clermont-Ferrand, Clermont-Ferrand, F-63000, France ;

##### **Correspondence**: Guillaume Coll, gcol@chu-clermontferrand.fr

*Fluids and Barriers of the CNS* 2023, **20(Suppl 2)**: A111

**Introduction**: The Sophysa SM8 valve is commonly used by neurosurgeons in France, but previous studies on shunt malfunctions in adults included multiple valve types, making it difficult to draw conclusions. This study aimed to specifically determine the incidence of Sophysa SM8 valve dysfunction in adults, focusing on complications related to its opening pressure regulation mechanism.

**Methods**: A retrospective analysis of 599 adult patients who underwent CSF shunt placement with Sophysa SM8 between 2000 and 2013 was conducted.

**Results**: The study included 599 patients, with a mean age at surgery of 64.15 years. The most common causes of hydrocephalus were normal pressure hydrocephalus (49%), traumatic hemorrhages (26.5%), and tumors (15.7%). The overall rate of complications was 22.04%, with disconnection (25%), migration (12.9%), overdrainage (9.1%), and proximal obstruction (6.8%) being the most frequent causes. There were also seven reported cases of infection (5.3%). The risk of shunt failure was 36% at 10 years, with 17% of revisions occurring within the first year. When analyzing mechanical dysfunction related only to the opening pressure regulation mechanism, the intrinsic complication rate was 3.5% for proximal obstructions and overdrainage and 5.3% for complications related to the double connector.

**Conclusions**: The study found that disconnections were a frequent complication of the Sophysa SM8 valve and were related to its two-connector system. Based on these results, the authors recommend using a one-piece device to reduce the risk of disconnection.

## A112 Incidence and Nature of Complications Associated with Ventriculoatrial Shunt Placement: A Meta-Analysis

### Oliveira Leonardo^1^, Bertani Raphael^2^, Batista Sávio^3^, Wouters Kim^4^, Ribas Luiz, Machinski Elcio^1^, Wesselovicz Rubens^1^, Dos Santos Gabriel^1^, Viegas Fabio^1^, C. G. Pinto Fernando^2^

#### ^1^State University of Ponta Grossa, Faculty of Medicine, Paraná, Brazil; ^2^Cerebral Hydrodinamics Group, Department of Neurosurgery, University of São Paulo, São Paulo, Brazil; ^3^Federal University of Rio de Janeiro, Faculty of Medicine, Rio de Janeiro, Brazil; ^4^Open University of the Netherlands, Faculty of psychology, Heerle, The Netherlands

##### **Correspondence**: Wouters Kim, k.wouters82@gmail.com

*Fluids and Barriers of the CNS* 2023, **20(Suppl 2)**: A112

**Introduction**: Ventriculoatrial shunts (VAS) are utilized as a medical device to manage conditions such as hydrocephalus, pseudotumor cerebri, and arachnoid cysts, by diverting excessive cerebrospinal fluid from the brain's ventricles into the right atrium of the heart. This helps relieve symptoms associated with these conditions. However, the use of VAS carries a significant risk of potential complications. Here we conducted a systematic review and meta-analysis to evaluate the complications associated with VAS placement.

**Methods**: We conducted a systematic review of observational studies on complications related to VAS placement, following the PRISMA guidelines. Our search encompassed several databases, including PUBMED, Cochrane Library, and Embase, up to the present day. For the purpose of this review, complications were defined as any adverse clinical event occurring after VAS placement. Studies were excluded if they did not report on the presence or absence of postoperative complications or if they had a sample size of less than four patients.

**Results**: Out of 2.832 articles that were reviewed, 79 were ultimately included in the analysis. The study involved 4416 patients who underwent VAS placement, and the follow-up period ranged from 2 to 308 months. The total rate of complications was 11.7%, with the most common being infections (6.2%) and intracranial hemorrhage (1.8%). Cardiac complications were observed in 0.8% of the cases, followed by hygroma (0.5%), glomerulonephritis (0.5%), and pulmonary complications (0.4%). Mortality related to VAS placement was observed in 0.4% of cases.

**Conclusions**: Our findings suggest that the overall rate of complications associated with VAS placement is relatively low, with infections and intracranial hemorrhages being the most commonly observed complications, and cardiac complications being rare.

## A113 After 15 years, what is changed in the knowledge of hydrocephalus? Survey of Harold O. Conn 15 years later

### Gianpaolo Petrella, Silvia Ciarlo, Graziano Taddei, Angelo Pompucci, Alessandro Pesce

#### ^1^A.O. “Santa Maria Goretti” General Hospital, Neurosurgery Division, Latina, Italy

##### **Correspondence**: Gianpaolo Petrella, gianpaolo_p@hotmail.com

*Fluids and Barriers of the CNS* 2023, **20(Suppl 2)**: A113

**Introduction**: This work is based on the experience of Harold O. Conn, a Yale Medical School faculty member, who developed NPH and for 10 years was erroneously diagnosed as cerebral atrophy and/or Parkinson’s disease. In 2008, on recognizing the lack of awareness of NPH by physicians, he initiated a survey to explore this problem.

**Methods**: We translated his survey into Italian, with the help of a licensed translator, of 10 – points questionnaire, and submitted it to medical doctors, both specialists and family physicians belonging to the Latina Local Medical Council, via email.

**Results**: A total of 134 Physicians answered the survey. Impressively, almost one-fifth of the answering physicians had never heard of NPH. The first half of them had learned about NPH in medical school, and the other half learned after medical school. A total of 75% of physicians declared to have never seen patients suffering from NPH in the past five years.

**Conclusions**: Although the number of physicians aware of NPH appears to have increased globally, 75% of them still do not recognize/treat NPH patients. Therefore, this condition still remains widely unrecognized and unfortunately, undertreated.

## A114 Reducing Over-Drainage Complications in Idiopathic Normal Pressure Hydrocephalus

### Adam Nunn, Antonina Dembinska-Kenner, Cristina Cernei, Kelly McManus, Olivier Sluijters, Richard J. Edwards

#### ^1^Department of Neurosurgery, Southmead Hospital, Bristol, UK

##### **Correspondence**: Richard Edwards, richard.edwards@nbt.nhs.uk

*Fluids and Barriers of the CNS* 2023, **20(Suppl 2)**: A114

**Introduction**: Subdural effusion/haematoma (CSDH) is one of the most common complications of shunt surgery in the older population but estimates of prevalence vary widely. Here, we investigate the relationship between shunt valve setting, risk of CSDH and outcome from shunting.

**Methods**: Complication data was collected retrospectively for all patients shunted for idiopathic NPH in the period April 2004 to April 2019. Patients were excluded if they received a fixed pressure valve. Total follow-up was median 4.0 years. Early in the series, valves were initially set at 120 or 140, but later in the series, settings of 160 or 180 were more common.

**Results**: A total of 387 patients received programmable valves for suspected iNPH in the study period. 47 patients (12.1%) developed a CSDH which occurred a median 3.0 months after shunt insertion. Age (HR 1.08, p = 0.007) and concomitant antiplatelet (HR 2.7, p = 0.001) were associated with a higher risk of CSDH but not concomitant anticoagulant (HR 1.8, p = 0.16). Shunt valve settings of 120 or 140 were significantly more likely to result in a CSDH compared to settings of 160 or 180 (HR 2.7 [p = 0.005]). Patients set at 120 or 140 were also more likely to require evacuation (5.8% vs 0.5%; HR 11.9 [p = 0.02]). Among the 240 patients whose shunt valve was not adjusted over 3 months of follow-up, those with a shunt setting of 120 or 140 were similarly more likely to have a CDSH compared to those set at 160 or 180 (HR 3.6, p = 0.03) yet there was no difference in the proportion improved at 3 months follow-up (92% vs 88% [p = 0.36].

**Conclusions**: Valve settings of 160 or 180 are associated with a lower risk of CSDH formation and no significant reduction in treatment effect. Antiplatelet agents increase the risk of CSDH more significantly than oral anticoagulants.

## A115 Time to Resolution of Normal Pressure Hydrocephalus (NPH)-associated Subdural Hematomas

### Michael Meggyesy^1^, Gwendolyn Williams^1^, Ryan P. Lee^1^, Jheesoo Ahn^1^, Christina Ritter^1^, Enoch Kim^2^, Mark G. Luciano^1^

#### ^1^Department of Neurosurgery, Johns Hopkins University School of Medicine, Baltimore, MD, 21205, USA; ^2^Nova Southeastern University Dr. Kiran C. Patel College of Allopathic Medicine, Fort Lauderdale, FL, 33314, USA

##### **Correspondence**: Michael Meggyesy, mmeggye1@jhmi.edu

*Fluids and Barriers of the CNS* 2023, **20(Suppl 2)**: A115

**Introduction**: Ventriculoperitoneal shunting (VPS) introduces a risk of overdrainage-associated subdural hygroma and hematoma (SDH/H). Since chronic SDH/H (cSDH/H) reverses and delays idiopathic normal pressure hydrocephalus (iNPH) treatment, we investigate this “unnatural history” of their resolution and the effectiveness of drainage and/or shunt adjustments.

**Methods**: This is a retrospective medical record review of all patients treated for cSDH/H with shunted iNPH between 2017 and 2022. We reviewed patient demographics, symptoms, shunt settings, head-CT (HCT) and cSDH/H characteristics to evaluate risk factors, time to resolution and time to reinitiate hydrocephalus treatment. CSDH/H treatment consisted generally of shunt adjustment to maximum opening pressure with drainage performed only with progression.

**Results**: Of 82 patients identified, 65 patients had an adequate available HCT series for evaluation. Unilateral cSDH/H was found in 35, bilateral in 30 patients. The average hematoma size was 1.28 cm (± 0.85) for unilateral, 1.01 cm (± 0.7) for bilateral. Signs of rebleed were identified in 43% (15) of unilateral and 67% (20) of bilateral cSDH. Median days (±IQR) to resolution in unilateral cSDH/H was 103 (±54.5) days, and 113 (±166) days for bilateral. The most used valve was a Certas+ with Siphonguard. The use of Siphonguard did not specifically seem to protect from cSDH in our cohort. Neither the occurrence of rebleeds, nor the initial use of anti-siphon/gravitational devices influenced time to resolution. Similarly, surgically related (7 day perioperative) versus delayed onset cSDH/H had similar resolution time. Unilateral cSDH/H resolved quicker than bilateral (p = 0.01).

**Conclusions**: Given the current treatment of cSDH/H, utilizing adjustments, hydrocephalus treatment is delayed by a median (±IQR) of 106 (±110) days. While the effect of cSDH/H and treatment delay on ultimate outcome is uncertain, further evaluation of factors influencing occurrence and resolution is needed.

## A116 Abdominal pain after ventriculoperitoneal shunting for normal pressure hydrocephalus (NPH): prevalence, timeline, and impact of quality of life

### Lennard-Quirin Rohde^1^, Anita Ulrich^2^, Uwe Kehler^3^

#### ^1^Department of Neurosurgery, Asklepios Klinik Altona, Hamburg, 22763, Germany

##### **Correspondence**: Prof. Dr. Dr. Uwe Kehler, u.kehler@asklepios.com

*Fluids and Barriers of the CNS* 2023, **20(Suppl 2)**: A116

**Introduction**: The frequency of abdominal pain after ventriculoperitoneal (VP) shunt placement is still largely unknown and underreported. The aim of this study is to determine the prevalence, the time course and the impact to quality of life.

**Methods**: In our department 149 patients with NPH were treated with VP-shunts between 2020 and 2021. Data were collected by digital patient records and by a follow-up questionnaire, where patients were asked about pain intensity according to the visual analog scale (VAS), duration of pain (days to weeks, 2–12 months, > 12 months), the limitations in daily life (no limitation, barely disturbing, disturbing but tolerable, substantially disturbing), and the use of pain medication. 94 patients responded to the follow-up questionnaire and could be evaluated. Statistical analysis was performed using logistic regression model.

**Results**: Overall, 39% (n = 37) of patients reported abdominal pain. In 16% (n = 15), pain occurred during hospitalization. 15% (n = 14) reported pain for more than 12 months. The overall pain intensity was: no pain: 61% (n = 57), slight (VAS 1–3): 10,6% (n = 10), medium (VAS 4–6): 22.3% (n = 21), severe (VAS 7–8): 6.4% (n = 6). Abdominal pain never reached VAS 9–10. 12 months after surgery pain improved or resolved completely: no pain: 85,1% (n = 80), slight: 2,1% (n = 2), medium: 8,5% (n = 8), severe: 4,3% (n = 4). Only 7 patients used medication for abdominal discomfort. In 2.1% (n = 2) abdominal discomfort was reported as substantially bothersome. There was no correlation with age or sex.

**Conclusions**: Abdominal pain after VP-Shunt is a frequent finding (almost 40%), decreases over time and rarely shows severe impact on quality of life. However, abdominal pain after VP-Shunts can influence the postsurgical course and patients should be informed beforehand. Further studies regarding the cause are recommended for possible improvements.

## A117 Frequency Of Abdominal Pain Related To Types Of Shunt Tubing: The difference in the tubing

### Kanza Tariq, Ahmed Toma, Lewis Thorne, Simon Thompson, Celine Vicedo, Laurence Watkins

#### ^1^National Hospital for Neurology and Neurosurgery, Queen Square, London, UK

##### **Correspondence**: Kanza Tariq kanza.tariq@nhs.net

*Fluids and Barriers of the CNS* 2023, **20(Suppl 2)**: A117

**Introduction**: A retrospective comparative study looking at the incidence of unexplained abdominal pain relative to peritoneal shunt catheters during the period of 2012 to 2020 was performed in our single-centre unit. After evaluation of 426 patient records, it was concluded that Ares shunt tubing was associated with a higher incidence of abdominal pain and revision surgery following peritoneal shunt insertion, as compared to Bactiseal, Silverline or plain tubing. We, therefore, subsequently performed a prospective comparative study evaluating the different antibiotic impregnated peritoneal shunt catheters.

**Methods**: A uniform length of Ares, Bactiseal and B.Braun antibiotic impregnated peritoneal shunt catheters were connected to sterile drainage systems, running normal saline continuously through the catheters at 20 ml/h. Samples were collected from the catheters at 1 week, 3weeks, 5weeks and 8weeks and tested for antibiotic concentrations to discern elution rate. The catheters were examined by our mechanical engineering team, exploring the rigidity, elasticity and surface microstructure of each catheter using nano-indentation for elastic modulus and 3-dimentional computerised tomography scan.

**Results**: Preliminary results from the Bristol antimicrobial reference laboratory indicate highest level of rifampicin and clindamycin in samples collected from Ares catheter indicating a higher antibiotic elution rate. Definitive quantitative results for rifampicin and clindamycin levels from the week to week samples collected for all the catheters, as well as in depth study into the physical properties of the catheters by the engineering team will be available by mid-May 2023.

**Conclusion**: Based on preliminary results it can be concluded that Ares peritoneal shunt catheter is associated with a higher antibiotic elution rate.

## A118 Transendoscopic Ultrasound for Neuroendoscopy

### Klaus D. M. Resch

#### ^1^MIN Univ. Guadalajara; Mexico

##### **Correspondence**: Klaus D. M. Resch klausdmresch@ens-surgery.com

*Fluids and Barriers of the CNS* 2023, **20(Suppl 2)**: A118

**Introduction**: After trans-endoscopic sono-catheters had been tested in the laboratory for imaging characteristics and practicability, clinical application was studied with special reference to imaging and navigation capabilities, practicability, safety, and indications.

**Methods**: Intraoperative endo-neuro sonography (ENS) images prepared during surgery on 75 selected patients were examined. There were 35 female and 40 male patients, and their mean age was 42 years (range 2–69 years). Within this series, there were 28 cases of ventricular lesions (ventricular hematomas, tumors, and colloid cyst included, and hydrocephaly).

**Results**: Imaging: In clinical use, the sono-catheter has superior imaging and navigation abilities to those seen in anatomical laboratory work. Real-time and online characteristics represent changes such as shifting, pulsation, CSF flow, blood flow, and changes in size and form of structures. When confronted with clinical problems, this technique still has some limitations such as short penetration depth of 3-cm radius and lack of scanning anterior to the endoscope. Navigation: The scan is radial 360° and in an orthogonal plane to the axis of the endoscope. At the tip of the endoscope, it delivers an image that looks geometrically like a “brain-radar”. Because of its real-time characteristic, ENS has a navigation capacity that markedly differs from usual neuronavigation but is intuitively usable. Endo-Neuro-Sonography (ENS) was applied in 8 hydrocephaly, 3 colloid cysts, 5 intraventricular hematomas, 1 septostomy, 11 ETVs, 2 cystostomies, 4 multiple cysts, and 1 tumor biopsy case. Some illustrative cases are presented.

**Conclusion**: Endo-Neuro-Sonography (ENS) is a tool for intraoperative real-time and online high-resolution imaging, and neuronavigation of endoscopes with a working channel at least 2 mm in diameter; it also has application in a wide variety of ventricular lesions. ENS is limited by small penetration depth and not scanning ahead to the endoscope anteriorly.

## A119 Choroid plexus-on-a-chip: a microfluidic model to study how cerebrospinal fluid secretion and blood-cerebrospinal fluid barrier function are affected by inflammation associated with hydrocephalus

### Hariharan Prashant^1^, Schwerk Christian^4^, Schroten Horst^4^, Blazer-Yost Bonnie^3^, Harris A. Carolyn^1,2^

#### ^1^Department of Biomedical Engineering, Wayne State University, Detroit, MI, United States; ^2^Department of Chemical Engineering and Materials Science, Wayne State University, Detroit, MI, United States; ^3^Department of Biology, Indiana University - Purdue University Indianapolis, IN, United States; ^4^Mannheim Medical Faculty, University of Heidelberg, Children’s Hospital, Mannheim, Germany

##### **Correspondence**: Prashant Hariharan, fj1852@wayne.edu

*Fluids and Barriers of the CNS* 2023, **20(Suppl 2)**: A119

**Introduction**: Presently, there are no successful, non-surgical, pharmaceutical interventions for hydrocephalus, in part because targeted cerebrospinal fluid (CSF) regulation requires a better understanding of the mechanism of CSF secretion through the choroid plexus (CP). Conventional animal models used to study CSF secretion are expensive, plagued by variability, have limited translatability to humans and involve incredibly challenging surgical procedures. Existing in vitro models are not suited for long term dynamic culture and do not capture the complexity of human physiology. There is a critical need for a highly reproducible, cost-effective, human-relevant model with physiological relevance to study CSF secretion at the CP. To directly address this need we developed an organ-on-a-chip model of the CP that allows us to track and manipulate secretion and barrier function of the CP.

**Methods**: Using computational fluid dynamics, 3D printing, and soft lithography we designed and fabricated a 2-compartment microfluidic platform that mimics the luminal and abluminal regions of the CP. The model provides the mechanical cue of physiological shear and maintains physiologically accurate tissue-fluid ratios and fluid turnover times.

**Results**: Immunofluorescent labeling has confirmed that choroid plexus epithelial cells grown inside the abluminal compartment orient themselves correctly and express critical tight junction components, establishing a low permeability barrier. Barrier function has also been assessed using transepithelial electrical resistance. Lastly, we have also successfully simulated secretion of fluid across compartments.

**Conclusions**: The human CP-on-a-chip has the potential to replace currently used pre-clinical animal studies with human-relevant systems in translational research and reveal previously undiscovered transport mechanisms at the CP. Future work involves testing our hypothesis regarding inflammation-mediated barrier integrity loss and subsequent CSF hypersecretion at the CP as it pertains to hydrocephalus.

## A120 Patient-specific automated cerebrospinal fluid pressure control to augment spinal wound closure: a case series using the LIQUOGUARD® 

### Danyal Z. Khan^1,2^, Kanza Tariq^1,3^*, Keng Siang Lee^4^, Edward W. Dyson^1,3^, Vittorio Russo^1^, ^†^Laurence D. Watkins^1,3^, ^†^Antonino Russo^1^

#### ^1^Department of Neurosurgery, National Hospital for Neurology and Neurosurgery, London, UK; ^2^Wellcome/EPSRC Centre for Interventional and Surgical Sciences, University College London, London, UK; ^3^Department of Brain Repair & Rehabilitation, UCL Queen Square Institute of Neurology, London, UK; ^4^Bristol Medical School, Faculty of Health Sciences, University of Bristol, Bristol, UK

^*^Denotes joint first authorship

^†^Denotes joint senior authorship

##### **Correspondence**: Kanza Tariq kanza.tariq@nhs.net

*Fluids and Barriers of the CNS* 2023, **20(Suppl 2)**: A120

**Introduction**: Post-operative spinal cerebrospinal fluid (CSF) leaks are a common and potentially serious surgical complication. The management of intra- and post-operative leaks is heterogeneous. Numerous studies advocate for dural repair and CSF diversion. The LiquoGuard7 allows automated and precise CSF pressure and volume control, with the calculation of cerebrospinal fluid production rate (PRcsf), allowing for tailored CSF drainage. We sought to summarize our experience with patient-specific CSF automated drainage with layered spinal wound closure.

**Methods**: This single-centre case series included patients undergoing complex spinal surgery where: 1) a high-flow intra- and/or postoperative CSF leak was expected and 2) concurrent CSF diversion was performed via lumbar drain attached to a LiquoGuard7®. LiquoGuard7® was used to calculate net PRcsf in each patient. CSF diversion was tailored to calculated CSF production rates and other case factors to maintain a neutral pressure across the operative site.

**Results**: Three patients were included, with a variety of pathologies (T7/T8 disc prolapse; T8-T9; T4-T5 metastatic spinal cord compression). The first two patients underwent CSF diversion to prevent post-op CSF leak, whilst case 3 required this in response to post-op CSF leak. CSF hyperproduction (140–150 ml/h) was evident in all cases. With patient-specific CSF diversion regimes (50–150 ml/h/7 days), no cases required further intervention for CSF fistulae repair (including for pleural CSF effusion), wound breakdown or infection.

**Conclusions**: Automated patient-specific cerebrospinal fluid drainage, based on patient’s net CSF production rate, may have a role in the closure of complex spinal wounds with high-flow CSF leaks, with a smaller risk profile than traditional manual drainage. Further larger studies are needed to explore the comparative benefits and cost-effectiveness of these devices.

## A121 Validation and Application of In Silico and In Vitro Modelling to Optimize Cerebrospinal Fluid Drug Delivery to the Brain

### Bryn A. Martin^1,2^, Lucas Sass^1^, Mohammadreza Khani^1^, Ostin Arters^1^, Stuart Sater^1^, Gabryel Conley Natividad^1^, Omolola Bangudu^1^, Katie Warthen^1^, Howard Dobson^3^, Scott Haller^4^, Richard Watts^5^, Kathrin Meyer^1,6^, Deep Singh^1^

#### ^1^Alcyone Therapeutics, Lowell, USA; ^2^University of Idaho, Moscow, ID, USA; ^3^InVicro Research Organization; ^4^Charles River Laboratory; ^5^Dept of Psychology, Yale University, ^6^Center for Gene Therapy, The Abigail Wexner Research Institute at Nationwide Children’s Hospital, Department of Pediatrics, The Ohio State University, Ohio, USA

##### **Correspondence**: Bryn A. Martin, bryn@alcyonetx.com

*Fluids and Barriers of the CNS* 2023, **20(Suppl 2)**: A121

**Introduction**: Targeted, consistent, and safe CSF drug delivery of genetic therapies is critical for effective treatment of CNS disorders. There are currently no available validated tools for prediction of CSF-system wide solute transport in nonhuman primates (NHP) or transformation of protocols to humans. We developed and utilized in silico and in vitro model systems to formulate a lumbar-access automated intrathecal catheter infusion system (FalconTM) designed to target the brain only, spinal cord only or brain and spinal cord together while being safe and clinically scalable. The model system predictions were then validated within NHPs in vivo.

**Methods**: CSF geometry and flow was collected from NHPs via MRI to define in silico computational fluid dynamics simulations and in vitro 3D printed models of the CSF system. The models were used to optimize delivery device and infusion parameters for widespread brain and spinal cord targeting and compared to lumbar puncture (LP) drug injection. To validate model predictions, seronegative NHPs were co-infused with gadolinium and AAV9.CB.GFP via LP (n = 5) or the optimized FalconTM delivery (n = 5). Biodistribution was analyzed 3w later by immunohistochemistry, ddPCR and western blots. In vivo drug flow was compared via gadolinium concentration.

**Results**: We confirmed CSF model predictions with in vivo NHP biodistribution. FalconTM was significantly superior, reaching the cranial space 1–5 min post-injection, while LP required 30–60 min. At 30 mins post injection, gadolinium was significantly greater throughout the cortical gray matter and basal ganglia with FalconTM versus LP. FalconTM vector genome copies per diploid genome was significantly greater throughout brain regions versus LP.

**Conclusions**: We developed a novel state-of-the-art in silico and in vitro NHP modeling system and validated model predictions in vivo. The model was used to design and optimize the FalconTM intrathecal catheter delivery system that demonstrated faster and broader distribution of AAV9 to the CNS compared to LP.

## A122 Quantification of Regional Neural Tissue Strain in Type I Chiari Malformation

### Gwendolyn Williams^1^, Michael Meggyesy^1^, Dipankar Biswas^1^, Bryn Martin^2^, Mark Luciano^1^, Ari Blitz^3^, John Oshinski^4^

#### ^1^Department of Neurosurgery, Johns Hopkins University School of Medicine, Baltimore, MD, 21287, USA; ^2^Alycone Therapeutics, Inc., Lowell, MA, 01852, USA; ^3^Department of Radiology, School of Medicine, Case Western Reserve University, Cleveland, OH, 44106, USA; ^4^Department of Radiology & Imaging Science and Biomedical Engineering, Emory University, Atlanta, GA, 30322, USA

##### **Correspondence**: Gwendolyn Williams, gwilli85@jh.edu

*Fluids and Barriers of the CNS* 2023, **20(Suppl 2)**: A122

**Introduction**: Type I Chiari malformation (CMI) is believed to be a cerebrospinal fluid (CSF) related disorder, making it a subject of interest for hydrodynamic research. The unique anatomy of CMI at the cervicomedullary junction causes an impedance to CSF flow, resulting in abnormal tissue motion. This abnormal tissue motion and CSF flow may result in altered tissue strain. While it is known that brain tissue is sensitive to low magnitude strains, the exact role of brain tissue strain in CMI pathophysiology is yet to be defined. We hypothesized that tissue motion and strain resulting from the altered flow of CSF in symptomatic CMI patients would be different from healthy controls and asymptomatic CMI patients.

**Methods**: Rostral-caudal tissue motion was quantified using displacement-encoding with stimulated echoes (DENSE) MRI. Principle strain in four brain regions was derived from displacement: the pons, medulla, cerebellar tonsil, and upper spinal cord. Peak-to-peak displacement and mean principle compression and extension strains are reported.

**Results**: Average peak-to-peak rostral-caudal displacements ± STD of the upper spinal cord (SC) for the control, symptomatic, and asymptomatic groups were 0.14 ± 0.06, 0.18 ± 0.10, and 0.11 ± 0.03 mm, respectively and of the cerebellar tonsil were 0.07 ± 0.03, 0.11 ± 0.06, and 0.10 ± 0.03 mm, respectively. The average principles strains (extension % ± STD/compression % ± STD) in the SC for the control, symptomatic, and asymptomatic groups were 1.88 ± 0.71%/1.51 ± 0.60%, 2.18 ± 1.83%/1.66 ± 2.01%, and 2.05 ± 0.93%/1.64 ± 0.66%, respectively.

**Conclusions**: Preliminary results presented here indicate abnormal tissue motion and strain in symptomatic CMI patients compared to healthy controls and asymptomatic CMI. These findings indicate DENSE tissue strain measurements could provide utility in better understanding CMI and related pathologies, as well as aid in defining a novel biomarker for CMI.

## A123 Correlation of cerebrospinal fluid and plasma adipokines in obese versus non-obese idiopathic intracranial hypertension (IIH) patients

### Sara Ho, Aida Kamalian, Abhay Moghekar

#### ^1^Department of Neurology, Johns HopkinsUniversity, Baltimore, MD, 21224, USA

##### **Correspondence**: Abhay Moghekar, am@jhmi.edu

*Fluids and Barriers of the CNS* 2023, **20(Suppl 2)**: A123

**Introduction** : Determination of key markers of obesity and inflammation could potentially help elucidate IIH pathophysiology. The aim of this study was to measure adipokines and cytokines implicated in prior pilot studies, in CSF and plasma from IIH patients to determine correlations of these markers in plasma and CSF and assess group differences.

**Methods** : Patients seen at the Johns Hopkins Center for CSF Disorders were assessed for IIH using visual field testing and fundus perimetry, as well as lumbar puncture manometry to measure cerebrospinal fluid opening pressure in the left lateral decubitus position. CSF and plasma samples were analyzed for C-peptide, IL-6, insulin, leptin, resistin, tumor necrosis factor alpha (TNF-a), adiponectin, and retinol binding protein 4 (RBP4) by multiplex immunoassay (Luminex, Intelliflex). Pearson correlation was used to determine correlation between CSF and plasma analytes and comparisons between cohorts were determined by ANOVA.

**Results** : 244 patients (231F, 14M) ranging in age from 15 to 73 years (Mean 39 ± 12.74) were classified as obese IIH (N = 102, Mean BMI 40.11 ± 7.65), non-obese IIH (N = 24, Mean BMI 26.25 ± 2.65), obese normal (N = 68, Mean BMI 38.12 ± 5.98), and non-obese normal (N = 49, Mean BMI 26.29 ± 2.73). In matched CSF and plasma from all patients, leptin, C-peptide, resistin, RBP4, and insulin demonstrated moderate correlations (r = 0.26, p < 0.01; r = 0.35, p < 0.0005; r = 0.39, p < 0.00005; r = -0.31, p < 0.001; r = 0.28, p < 0.005 respectively). When stratified by cohort, plasma levels of C-peptide, insulin, leptin, and TNF-a were significantly higher in obese patients, regardless of disease status (p = 0.043; p = 0.012; p = 0.0067; p = 0.019). In CSF, insulin and leptin were elevated in obese IIH patients (p = 0.012; p < 0.0001) but not obese normals, whereas C-peptide was elevated in obese normals (p < 0.0001).

**Conclusions** : Brain-specific adipokines are altered in obese IIH patients but not obese healthy controls, suggesting that these pathways contribute to IIH development and may represent a potential characteristic profile.

## A124 A comparison of outcomes between pediatric and adult patients with idiopathic intracranial hypertension (IIH)

### Thaddeus Harbaugh, Manvita Mareboina, Derek Barnett, Elias Rizk

#### ^1^Department of Neurosurgery, Penn State Hershey Medical Center, Pennsylvania, 17033, USA

##### **Correspondence**: Thaddeus Daniel Harbaugh, tdh5264@psu.edu

*Fluids and Barriers of the CNS* 2023, **20(Suppl 2)**: A124

**Introduction**: Idiopathic intracranial hypertension (IIH) is a condition that occurs due to increased intracranial pressure without an identifiable cause. Symptoms of IIH include headaches, visual disturbances, tinnitus, and nausea. It is important to study this condition due to its serious and potentially permanent consequences, particularly for pediatric patients. Furthermore, the mechanism of this condition and its effects on certain populations are not quite understood.

**Methods**: This study aims to compare treatment types and outcomes across pediatric and adult populations. A total of 86,873 adult patients with IIH and 14,446 pediatric patients with IIH were assessed. Treatment types compared across cohorts included Ventriculoperitoneal Shunt (VPS), Endoscopic Third Ventriculostomy (ETV) and Stenting. Outcomes compared included death, infection, seizures, number of CT scans, and number of emergency department visits. Differences in sex, race, BMI were also determined.

**Results**: Results showed no significant difference in the number of Ventriculoperitoneal Shunt (VPS) placements between pediatric patients and adult IIH patients. However, pediatric patients were significantly more likely to have an ETV done and adult patients were significantly more likely to have stent placement. Additionally, adult patients were significantly more likely to die but were significantly less likely to have an infection or seizure when compared to pediatric patients.

**Conclusions**: In conclusion, this study provides valuable insights into the differences in treatment types and outcomes between pediatric and adult patients with IIH. These findings have important implications for clinicians and researchers, as they underscore the need for tailored approaches to the management of IIH in different patient populations. Ultimately, a better understanding of IIH and its management will help improve patient outcomes and quality of life.

## A125 Long-term outcomes after dural venous stenting in Idiopathic intracranial hypertension: a single centre experience

### Muhammad A. Kamal, Zeid Abussuud, Natasha Angadi, Ahmed Toma

#### ^1^Victor Horsley Department of Neurosurgery, National Hospital for Neurosurgery and Neurosurgery, London UK

##### **Correspondence**: Muhammad Ahmad Kamal, muhammadakamal@hotmail.com

*Fluids and Barriers of the CNS* 2023, **20(Suppl 2)**: A125

**Introduction**: Idiopathic Intracranial Hypertension (IIH) is a neurological disorder typically characterised with signs and symptoms of raised intracranial pressure (ICP). This includes high-pressure type headaches and visual disturbances. Dural venous stenosis is a potential cause of IIH . Increased pressure gradients caused by stenosis may contribute to elevated ICPs. Dural venous sinus stents can be used as a treatment to obliterate the pressure gradients. The aim of this study is to evaluate long-term outcomes after stenting for IIH.

**Methods**: Retrospective single centre review of case notes and imaging records of 42 patients with Idiopathic Intracranial Hypertension (IIH) and dural venous sinus stenosis who underwent dural venous stenting over an eight-year period (2015–2023) at our regional neurosurgical centre.

**Results**: 42 patients, 38 female and 4 males with a mean age of 32 years (range 19–52), were treated with dural venous stents. Almost all the patients had transverse sinus stenosis. 90% of patients presented with headaches and 73% had confirmed visual loss on ophthalmological examination. Immediately after stent placement, an improvement of pressure gradients was noted in 78% of patients with a corresponding reduction of intracranial pressures in 75% of patients. Following dural venous stent placement, 88% of patients had improved vision, whilst only 26% of patients reported a sustained improvement in headaches. 38% of patients required further management with cerebrospinal fluid diversion with a shunt. Two patients required re-stenting, and one patient had a partially occlusive thrombus.

**Conclusion**: We describe the use of dural venous stents in the management of patients with IIH. Treatment of IIH aims to reduce ICP and preserve visual loss. Results from this study demonstrated a subsequent reduction in ICP following stent placement and this reflected in objective and subjective improvements in visual symptoms. The long-term complication rate was low.

## A126 IIH Intervention: opening of a randomised clinical trial comparing Dural Venous Sinus Stenting with Cerebrospinal Fluid shunting in Idiopathic Intracranial Hypertension

### Gopiga Thanabalasundaram^1^, Ahmed Toma^2^, Fergus Robertson^3^, Phil White^4^, Susan P. Mollan^5,6^, Alexandra J. Sinclair^6,7^, Georgios Tsermoulas^1,6^

#### ^1^Department of Neurosurgery, Queen Elizabeth Hospital Birmingham, United Kingdom; ^2^Department of Neurosurgery, National Hospital for Neurology and Neurosurgery, London, United Kingdom; ^3^Department of Neuro-Radiology, National Hospital for Neurology and Neurosurgery, London, United Kingdom; ^4^Department of Neuro-Radiology, Royal Victoria Infirmary, Newcastle-Upon-Tyne, United Kingdom; ^5^Birmingham Neuro-Ophthalmology, Queen Elizabeth Hospital Birmingham, United Kingdom; ^6^Institute of Metabolism and Systems Research, University of Birmingham, United Kingdom; ^7^Department of Neurology, Queen Elizabeth Hospital Birmingham, United Kingdom

##### **Correspondence**: Georgios Tsermoulas, georgios.tsermoulas@nhs.net

*Fluids and Barriers of the CNS* 2023, **20(Suppl 2)**: A126

**Introduction**: The morbidity in Idiopathic Intracranial Hypertension (IIH) is primarily related to visual loss and headaches. Patients at risk of permanent visual loss require urgent intervention to reduce intracranial pressure (ICP). The most widely used surgical procedure is cerebrospinal fluid (CSF) shunting and over the last two decades dural venous sinus stenting (DVSS) has been used as an alternative intervention. Currently, there is no direct comparison of the two interventions or high quality evidence to guide clinical practice.

**Methods**: IIH intervention is a multicentre phase IIb randomised trial with integrated health economic evaluation that will compare the effectiveness of DVSS with CSF shunting to prevent visual loss. The target population is 138 people recruited in 15 sites and randomization will be 1:1 for the two interventions. The primary outcome will be visual function over 12 months and secondary outcomes include improvement in headaches, surgical complications and revisions, impact on quality of life and cost-effectiveness.

**Results**: The study has received regulatory approval and will open in the first sites in April 2023. Each patient will stay in the trial for 2 years, accrual period is 2.5 years and patient data will be collected from a national electronic database for 10 years. The primary outcome measure will be Humphrey Visual Field Perimetric Mean Deviation over 12 months. Secondary measures will assess the impact of the two interventions over 12 and 24 months on vision, headache, tinnitus, quality of life and economic cost.

**Conclusions**: IIH Intervention will compare DVSS with CSF shunting and will answer an important clinical question, not least because the incidence of IIH is rising in line with the global obesity epidemic. The results of this trial will change practice and inform development of clinical guidelines on the management of IIH.

## A127 ICP dynamics during movement in Chiari malformation and idiopathic intracranial hypertension

### Eleanor M. Moncur^1,2^, Matthew J. Bancroft^2^, Linda D’Antona^1,2^, Graziella Favarato^2^, Lewis Thorne^1^, Laurence D. Watkins^1^, Ahmed K. Toma^1,2^

#### ^1^Victor Horsley Department of Neurosurgery, National Hospital for Neurology and Neurosurgery, London, WC1N3BG, UK; ^2^Department of Brain Repair and Rehabilitation, UCL Queen Square Institute of Neurology, University College London, London, WC1N3BG, UK

##### **Correspondence**: Eleanor Moncur, e.moncur@nhs.net

*Fluids and Barriers of the CNS* 2023, **20(Suppl 2)**: A127

**Introduction**: Intracranial pressure (ICP) changes with body movement, however most ICP research has been performed in stationary patients. This is particularly relevant for patients with CSF dynamic disorders such as Chiari malformation and idiopathic intracranial hypertension (IIH) because symptoms and disease severity are affected by body movement and position. Here we investigate ICP dynamics *during* movement in these groups.

**Methods**: Single-centre prospective observational study. Patients undergoing continuous ICP monitoring were fitted with continuous position sensors and underwent set sequences of movements including lying, sitting, standing and head movements. A proportion of patients also underwent passive positional change using a tilt table whilst ICP and position monitoring was ongoing. ICP and postural data were recorded at 100Hz and synchronized. Data were analysed for ICP, PA and waveform ICP behaviour *during* movement using a regression model adjusted for sex, shunt status and BMI.

**Results**: Sixty-seven patients (16M, mean age 43±13) were recruited. 16 participants had CSF-diverting shunts in situ, 15 had Chiari malformation and 11 had IIH. A transient increase in ICP occurred during movement that varied in magnitude. After controlling for sex, shunt status and BMI, Chiari participants tend to have a smaller amplitude increase than non-Chiari participants during all active torso movements and participants with IIH tended to have a larger amplitude increase compared with non-IIH participants. In the IIH group, participants with higher BMI had higher increases than participants with normal BMI and this was more pronounced in lying-sitting movements. Differences were also observed between shunt statuses including whether an antisiphon device was present or not. Further analyses will be presented.

**Conclusions**: We present data on ICP dynamics during movement in Chiari malformation and IIH. This has implications for understanding the mechanisms of symptomatology and compliance abnormalities in these pathologies and is an avenue that requires more exploration.

## A128 Synthetic MRI: a fast and reliable method for ventricular volumetry

### Rafael T. Holmgren^1^, Anders Tisell^2^, Marcel Warntjes^3^, Charalampos Georgiopoulos^4^

#### ^1^Department of Neurosurgery, and Department of Biomedical and Clinical Sciences, Linköping University, Linköping, Sweden; ^2^Department of medical radiation physics, and department of caring sciences, Linköping University. Center for medical image science and visualization (CMIV), Linköping University, Sweden; ^3^Center for medical image science and visualization (CMIV), Linköping University. SyntheticMR AB, Linköping, Sweden; ^4^Diagnostic Radiology, Department of Clinical Sciences, Medical Faculty, Lund University, Lund, Sweden

##### **Correspondence**: Rafael T. Holmgren, Rafael.holmgren@gmail.com

*Fluids and Barriers of the CNS* 2023, **20(Suppl 2)**: A128

**Introduction**: Volumetry of cerebral ventricles is a far more sensitive measure for shunt-induced reduction of ventricular size than traditional 2D-measures, such as Evans index. However, available ventricle segmentation methods are time-consuming, which limits use in clinical practice.

Quantitative MRI (qMRI) obtains quantitative measurements of physical properties of tissues used for automatic segmentation of white and grey matter and intracranial CSF. With a 6-minute 3D qMRI scan all relevant data are acquired to perform tissue-aided ventricle segmentation. The aim of this study was to evaluate and reliability-test a qMRI-software (SyntheticMRI) for ventricle volumetry, including a semi-automated segmentation algorithm.

**Methods**: 45 3D-qMRI scans (15 healthy subjects, 15 iNPH-patients, 15 shunted iNPH-patients) were assessed twice for ventricular volumetry by two independent examiners (one neurosurgeon and one neuroradiologist). Total intraventricular CSF-volumes, extraventricular intracranial CSF and required time for manual segmentations were recorded. Segmentations generated by an automated ventricle segmentation algorithm (n = 15) were manually corrected by the neurosurgeon to obtain another set of data.

**Results**: Intra- and interobserver reliability for all segmentations was excellent (ICC 1.000). Ventricular volumes were on average 42 ml (range 17–82) in healthy subjects, 141 ml (range 82–194) in iNPH-patients and 113 ml (range 59–186) in shunted iNPH-patients. The learning curve of manual correction was steep, with an average 23% reduction time between segmentations no 1 and 2. The average time for examiners was 14.75 and 42.75 min, respectively. The time spent manually correcting the automated algorithm was significantly lower, on average by 5 min and 51 s.

**Conclusions**: SyntheticMRI is a reliable and efficient method to obtain relevant volumetric measures of intracranial CSF-spaces for both clinical and research purposes. Manual segmentation showed a steep learning curve and especially the manually corrected automated algorithm provides a feasible time expenditure for clinicians caring for patients with iNPH, advanced hydrocephalus and arachnoid cysts.

## A129 Non-invasive phase-contrast MRI in idiopathic intracranial hypertension - first promising data from a prospective study

### Katharina Wolf^1,2^, Wolf Lagrèze^3^, Marco Reisert^4^, Alexandra Camp^3^, Tim Bleul^3^, Mukesh Shah^2^, Hansjörg Mast^5^, Sebastian Küchlin^3^, Samer Elsheikh^5^, Niklas Lützen^5^, Jürgen Beck^2^, Horst Urbach^5^

#### ^1^Department of Neurology, Medical Center, University of Freiburg, 79106, Germany; ^2^Department of Neurosurgery, Medical Center, University of Freiburg, 79106, Germany; ^3^Department of Ophthalmology, Medical Center, University of Freiburg, 79106, Germany; ^4^Department of Radiology, Medical Physics, Medical Center, University of Freiburg, 79106, Germany; ^5^Department of Neuroradiology, Medical Center, University of Freiburg, 79106, Germany

##### **Correspondence**: Dr. Katharina Wolf, katharina.wolf@uniklinik-freiburg.de

*Fluids and Barriers of the CNS* 2023, **20(Suppl 2)**: A129

**Introduction**: Idiopathic intracranial hypertension (IIH) is a condition defined by increased pressure measured by lumbar puncture ≥ 25 cmH2O, papilloedema and the absence of a competing etiology. To date, the only method to measure intracranial hypertension must be invasive. In view of increasing evidence of IIH patients presenting with CSF leaks, for IIH without papilloedema, new therapeutic studies, and rebound hypertension after closing of CSF leaks a noninvasive monitoring of patients would be helpful. A previous study has demonstrated that MRI-based measurements of spinal cord motion at segment C2/C3 are increased in patients with CSF leaks . It is hypothesized that there is reduced motion in IIH patients due to increased resistance.

**Methods**: Prospective, controlled study on 15 IIH patients, 100% female, with proven elevated CSF pressure ≥ 25 cmH2O and papilloedema without loss of visual acuity, and 33 female healthy controls. All subjects received axial, ECG-triggered phase-contrast MRI measurements at level C2/C3. Analysis was fully automated (www.nora-imaging.org). The velocity range (mm/s) and the total displacement (mm) of the time-resolved velocity curve over the cardiac cycle was used as the main parameter. Pairwise comparisons were made by Mann-Whitney U test; correlation was determined using regression models.

**Results**: Mean opening pressure was 31 ± 5 cmH2O. Spinal cord velocity range and total displacement was significantly lower in IIH patients as compared to controls: 3.9 ± 1.4 mm/s vs. 5.3 ± 1.3 mm/s, p = 0.001; 0.5 ± 0.1 mm vs. 0.7 ± 0.2 mm, p = 0.002 (Figure 1). Opening pressure and BMI showed no significant impact on dynamic parameters in patients. Data corrected for age did not reveal any change.

**Conclusions**: We report first evidence, that in disorders with increased CSF pressure, the physiological oscillation of the spinal cord CSF is dampened. Thus, this method might be helpful to solve clinical and diagnostic ambiguity.

**Figure:**
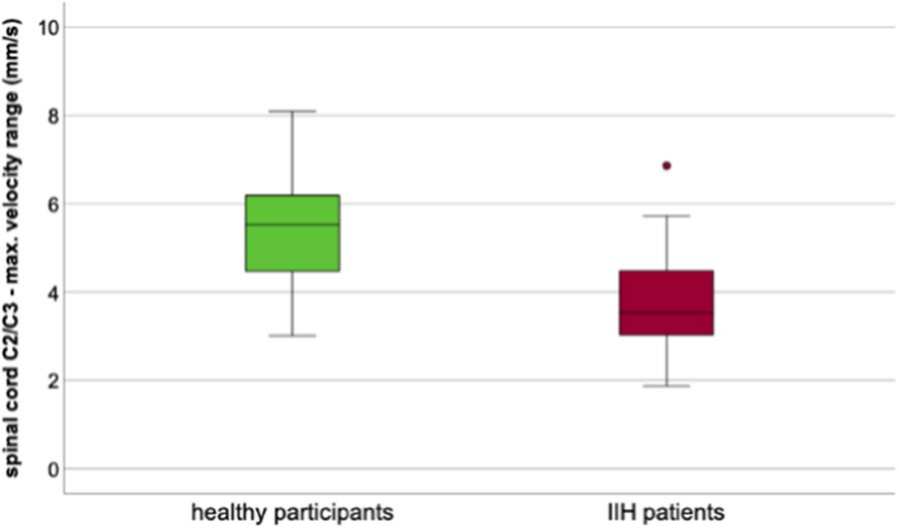
Figure 1: CSF flow velocities and spinal cord velocities in craniocaudal direction (velocity range – mm/s) is significantly increased in patients with SIH, p = 0.001

## A130 Cerebral arterial dynamic in hydrocephalus patients

### Kimi P. Owashi^1^, Cyrille Capel^1,2^, Serge Metanbou^3^, Zofia Czosnyka^5^, Marek Czosnyka^5^, Peter Smielewski^5^, Alexis Joannides^5^, Olivier Balédent^1,4^

#### ^1^CHIMERE UR7516, Jules Verne University, Amiens, France; ^2^Neurosurgery, University Hospital, Amiens, France; ^3^Radiology, University Hospital, Amiens, France; ^4^Image processing, University Hospital, Amiens, France; ^5^Department of Clinical Neurosciences, University of Cambridge, UK

##### **Correspondence**: Kimi Piedad Owashi, kimi.owashi@u-picardie.fr

*Fluids and Barriers of the CNS* 2023, **20(Suppl 2)**: A130

**Introduction**: Active reversible hydrocephalus (ARH) is a neurological condition mainly characterized by cerebrospinal fluid (CSF) flow disorders. However, it is known that CSF oscillations during the cardiac cycle are closely related to cerebral blood flow. Rapid intracranial arterial inflow initiates compensatory mechanisms, including CSF oscillations and venous outflow. Since arterial flow is the main driver of these neuro-fluids interactions, this study aims to compare cerebral arterial flow dynamics in healthy elderly volunteers (HEV) and suspected ARH patients as an appropriate short-term step in analyzing ARH physiopathology.

**Methods**: Twenty HEV (72±6 years) and thirty suspected ARH patients (73±8 years) underwent 3T MRI. From the value of resistance to CSF outflow (Rout), the patients were classified as suitable (ARH+: n = 14) or unsuitable (ARH-: n = 16) for shunting. Phase contrast MRI acquisitions were used to quantify the flow dynamics in cerebral arteries during the cardiac cycle. A MATLAB script was implemented to identify the main key points (time, amplitude) of the total cerebral arterial flow. Representative arterial profiles were estimated for each group. Moreover, we evaluated the effects of pathological conditions on flow times and amplitude-related parameters.

**Results**: The patients showed a significant decrease in the average cerebral arterial flow compared to HEV (ARH+: 452±116, ARH-: 456±97, HEV: 607±125 ml/min). Arterial profiles of ARH+ and ARH- presented very close morphologies; however, the intracranial pulsatility index (PI) of ARH+ was importantly higher than that of HEV (1.4±0.4 vs. 1.1±0.2), whereas the PI between ARH- and HEV did not differ significantly.

**Conclusions**: ARH+ patients appear to present higher PI values, which may reflect stiffer and less elastic arterial walls that could contribute to the development of the disease. Since the arterial flow is the leading actor in brain dynamics, impaired arterial blood flow could affect CSF oscillations and its distribution in the craniospinal compartments.

## A131 Intracranial CSF-ISF Flow Patterns in Large Animal Model

### Michael Meggyesy^1^, Di Cao^2,3,4^, Dipankar Biswas^1^, Gwendolyn Williams^1^, Enoch Kim^5^, Jun Hua^2,3^, Mark G. Luciano^1^

#### ^1^Department of Neurosurgery, Johns Hopkins University School of Medicine, Baltimore, MD, 21205, USA; ^2^F.M. Kirby Research Center for Functional Brain Imaging, Kennedy Krieger Institute, Baltimore, MD, USA; ^3^Neurosection, Division of MRI Research, Department of Radiology, Johns Hopkins, Baltimore, MD, USA; ^4^Department of Biomedical Engineering, Johns Hopkins, Baltimore, MD, USA; ^5^Nova Southeastern University Dr. Kiran C. Patel College of Allopathic Medicine, Fort Lauderdale, FL, 33314, USA

##### **Correspondence**: Michael Meggyesy, mmeggye1@jhmi.edu

*Fluids and Barriers of the CNS* 2023, **20(Suppl 2)**: A131

**Introduction**: Intracranial pathways of cerebrospinal fluid (CSF) are unclear to this date. Furthermore, interaction between intracranial fluids, molecules and solutes is ill-defined.

To better understand these complex interactions and fluid pathways, our goal was to create a large animal model to track movements of intracranially injected tracer at different locations and in iatrogenic induced pathophysiological states.

**Methods**: Six hounds received baseline MRI followed by a 0.1 mL cisternal or ventricular Gadoteridol injection. Injections were performed either through left ventricular catheter or cisterna magna. Follow-up MRIs were acquired every 30 min for five hours, and at 24 h post-injection. One subject had partial obstructive hydrocephalus induced by 4^th^ ventricular injection of cyanoacrylate glue, followed by the same MRI protocol. Regions of interest were identified at the aqueduct, 4^th^ ventricle, basal ganglia, parietal cortical and periventricular regions. Onset-time, time-to-peak, and clearance-time were estimated from the dynamic time courses.

**Results**: Cisternal baseline injections demonstrated fastest intracranial distribution of tracers along major arterial pathways. Signal change was noted among most cortical surfaces, but not at high parietal regions by hour five. Most uptake was noticed at the temporal lobe. Retrograde flow was noted into the ventricles. Ventricular injections showed trans-ependymal enhancement along the entirety of ventricles, with high uptake at the ipsilateral temporal horn, presumably due to gravity driven accumulation. Ventricular injections followed cisternal patterns once they reached extracranial CSF spaces.

The average time to peak was three hours for the lateral ventricle and temporal horn area, whereas uptake in basal nuclei, thalamus and 4^th^ ventricle was detectable but noticeably slower.

**Conclusions**: Solute distribution in the CNS follows major arterial pathways, and uptake into the parenchyma/interstitial space occurs through cortical as well as trans-ependymal routes. Stereotactical tracer injections may allow for better understanding of CSF interactions in specific brain regions in the future.

## A132 Optic nerve sheath diameter distension in normal pressure hydrocephalus: a potential marker for shunt responsiveness

### Linda D’Antona^1,2^, Zeid Abussuud^1^, David Rowland^1^, Eleanor Moncour^1,2^, Kanza Tariq^1,2^, Lucia Darie^1^, Aleksandra Lasica^2^, Lewis Thorne^1^, Laurence Watkins^1,2^, Ahmed Toma^1,2^

#### ^1^Victor Horsley Department of Neurosurgery, National Hospital for Neurology and Neurosurgery, London, WC1N3BG, UK; ^2^Queen Square Institute of Neurology, University College London, London, WC1N3BG, UK

##### **Correspondence**: Linda D’Antona, linda.d’antona@nhs.net

*Fluids and Barriers of the CNS* 2023, **20(Suppl 2)**: A132

**Introduction**: Optic nerve sheath diameter (ONSD) is a useful noninvasive marker for the identification of patients with abnormal cerebrospinal fluid (CSF) dynamics both in emergency and elective settings. Studies employing ultrasound and MRI techniques have confirmed the association between ONSD distension and raised ICP, however its association with Normal Pressure Hydrocephalus (NPH) has not been described before. This study investigated the prevalence of this sign in NPH and its utility in the identification of shunt-responsive NPH patients.

**Methods**: Retrospective single-centre study. Patients admitted for extended lumbar drainage (LD) for suspected NPH since January 2020 were identified. Inclusion criteria were (a) complete recording of pre- and post-LD walking test results; (b) brain MRI performed before treatment with ventriculoperitoneal shunt. Patients with 10% improvement in their post-LD walking test were considered shunt ‘responders’. ONSD was measured on axial T2 MRI sequences and considered abnormal or distended if  > 2mm in thickness in the area immediately behind the globe. Data on walking test results and ONSD were collected by independent assessors.

**Results**: Thirty-three patients (19M, 14F, mean age 74±8 years) met the inclusion criteria. Twelve patients (36%) had ONSD distension. Based on the post-LD walking test, 26 patients (79%) were classified as ‘responders’ and 7 patients (21%) were ‘non-responders’. ‘Responders’ had an average walking speed improvement of 26%. Amongst the ‘responders’, 38.5% had distension of the ONSD (sensitivity), while 71% of the ‘non-responders’ had normal ONSD (specificity). The patients with distended ONSD were classified as responders in 83% of the cases (positive predictive value).

**Conclusions**: These results suggest that despite having a ‘normal pressure’, NPH patients often have distension of the ONSD. In addition, this sign could be a useful non-invasive marker for the detection of shunt responsiveness. Larger studies will be needed to confirm these findings.

## A133 Potential impact of non-invasive phase-contrast MRI in the diagnosis of spontaneous intracranial hypotension (SIH)

### Katharina Wolf^1^, Niklas Lützen^2^, Marco Reisert^3^, Hansjörg Mast^2^, Florian Volz^1^, Amir El Rahal^1^, Christian Fung^1^, Jürgen Beck^1^, Horst Urbach^2^

#### ^1^Department of Neurosurgery, Medical Center, University of Freiburg, 79106, Germany; ^2^Department of Neuroradiology, Medical Center, University of Freiburg, 79106, Germany; ^3^Department of Radiology, Medical Physics, Medical Center, University of Freiburg, 79106, Germany

##### **Correspondence**: Dr. Katharina Wolf, katharina.wolf@uniklinik-freiburg.de Full

*Fluids and Barriers of the CNS* 2023, **20(Suppl 2)**: A133

**Introduction**: CSF flow velocities and spinal cord motion per heartbeat can be quantified by phase-contrast MRI. We have recently published a prospective proof-of-concept study showing increased CSF flow velocities and increased spinal cord velocities at the cervical segment C2/C3 in 20 patients suffering from spontaneous intracranial hypotension (SIH) with definite ventral or lateral spinal leaks. This new method might add to the diagnostic pathways. Meanwhile, the most challenging cohort among SIH patients are those with subtle leaks and/or CSF venous fistulas that require further invasive diagnostics. The main objective of this study was to test for the reproducibility of our previous findings.

**Methods**: Retrospective analysis of phase-contrast MRI measurements at C2/C3 that has been implemented in our current SIH workup since November 2021. All SIH patients with epidural fluid collections and/or localized leak between November 2021 to January 2023 were included (Type I, II, III; Schiewink et al. 2016). Segmentation and processing were fully automated (www.nora-imaging.org). Main parameter of interest was the velocity range (mm/s) in craniocaudal direction.

**Results**: We included 65 patients with SIH (52% with dural tears (Type I), 25% with meningeal diverticula (Type II), 20% CSF venous fistulas (Type III), 2% unknown leak site), and compared them to 68 healthy controls. CSF and spinal cord velocity ranges were significantly higher in SIH patients: CSF - 42 ± 10 mm/s vs. 58 ± 17 mm/s, p < 0.001; spinal cord 5.6 ± 1.4 mm/s vs. 7.7 ± 3 mm/s, p = 0.005 (Figure 1).

**Conclusions**: Our previous findings could be reproduced in a large cohort of patients with SIH that now represented a typical SIH cohort. Most importantly, current data included patients with CSF-venous fistulas that are most difficult to detect. These results underline the potential impact of this new diagnostic tool in CSF volume disorders.

**Figure:**
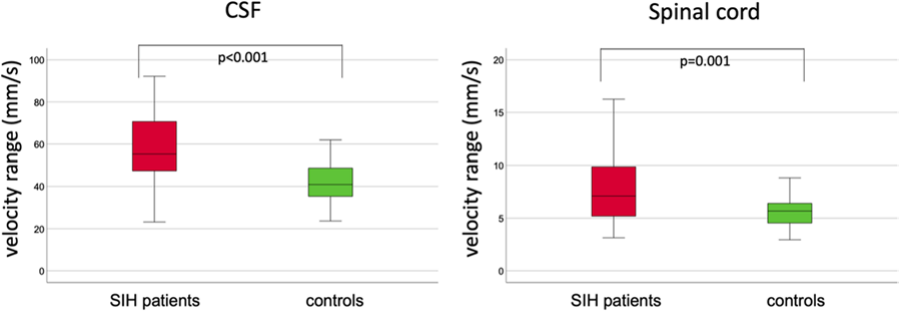
Figure 1: CSF flow velocities and spinal cord velocities in craniocaudal direction (velocity range – mm/s) is significantly increased in patients with SIH.

## A134 Reversible dementia project (REVERT): impact on improvement in awareness, diagnosis and management of normal pressure hydrocephalus

### Peter Smielewski^1^, Alexis J. Joannides^1,3^, Cyrille Capel^4^, Yael Fregier^5^, Michael Martin^6^, Zofia Czosnyka^1,3^, Olivier Peltre^5^, Michal Placek^1^, Kimi Owashi^2^, Pan Liu^4^, Serge Metanbo^4^, Rocio Fernandez-Mendez^3^, Toby Meek^3^, Lisa Healy^3^, Benjamin Dias-Dougan^3^, Rebecca Reece^3^, Romuald Seizeur^7^, Alin Bohra^8^, Marija Drinjakovic^1^, John Pickard^3^, Marek Czosnyka^1,3^, Olivier Baledent^2,4^

#### ^1^Brain Physics Laboratory, Department of Clinical Neurosciences, University of Cambridge, Cambridge UK; ^2^CHIMERE Laboratory, University of Picardie Jules Verne, Amiens, France; ^3^Division of Neurosurgery, Cambridge University Hospitals, Cambridge Biomedical Campus, Cambridge, UK; ^4^Jules Verne University hospital, Amiens, France; ^5^Lens Mathematics Laboratory, University of Artois, Arras, France; ^6^Obex Technologies, Cambridge, UK; ^7^Neurosurgery Dept, University Hospital of Brest, Brest, France; ^8^Neurosurgery Dept, University Hospital of Caen, Caen, France

##### **Correspondence**: Peter Smielewski, p10011@cam.ac.uk

*Fluids and Barriers of the CNS* 2023, **20(Suppl 2)**: A134

**Study supported by**: Revert Project, Interreg, France (Channel Manche) England, funded by European Regional Development Fund.

**Introduction**: REVERT, funded in October 2020 by ERDF, an Interreg France (Channel) England Programme, aimed to establish a common clinical and research network of excellence to transform the current management pathway of patients suspected of normal pressure hydrocephalus in the UK-French cross-border region. Following its official completion in March 2023, the goal of this paper is to summarise and discuss its main outputs.

**Methods**: A consortium of clinicians, physicists, mathematicians and software specialists from 3 Universities, 4 hospitals and an IT company worked towards addressing the following challenges in the management of NPH:

- insufficient referrals, due to low awareness of the disease in the context of dementia like symptoms
- lack of clear guidelines for optimal diagnostic workup
- incomplete understanding of physiology of pressure-flow-volume relationships in the cerebrospinal space
- inadequate understanding of the interplay between the cerebro-vascular and CSF circulation systems
- limited access and familiarity to advanced supplementary tests (CSF dynamics tests, PC MRI imaging)
- lack of consistent and appropriate outcomes measures to determine improvement after shunting

**Results**: The three key outputs of the project are: (1) establishment of a multidisciplinary one-stop clinic incorporating a core dataset of cognitive and gait measures for efficient screening and triage of new referrals, (2) refinement of infusion study and phase-contrast MRI mathematical models, and development of clinical protocols for the adoption of these supplementary tests within the routine care pathway, and (3) implementation of a digital portal for integrating data across the clinical pathway and for secondary linkage with national registries and research repositories.

**Conclusion**: Achieving impact from research translation in clinical practice necessitates an integrated approach to the entire patient pathway involving all relevant stakeholders. Novel approaches to the analysis and interpretation of the supplementary tests are essential for improving their predictive power, and require validation with appropriate clinical outcomes.

## A135 Bowel and Urinary Incontinence in Idiopathic Normal Pressure Hydrocephalus

### Andreas Eleftheriou^1^, Susanna Walter^2^, Johanna Rydja^3^, Katerina Owen^3^, Rafael Holmgren^4^, Fredrik Lundin^1^

#### ^1^Department of Neurology, and Department of Biomedical and Clinical Sciences, Linköping University, Linköping, Sweden; ^2^Department of Biomedical and Clinical Sciences (BVK), Division of Inflammation and Infection, Linköping University, Linköping, Sweden; ^3^Department of Activity and Health, and Department of Biomedical and Clinical Sciences, Linköping University, Linköping, Sweden; ^4^Department of Neurosurgery, and Department of Biomedical and Clinical Sciences, Linköping University, Linköping, Sweden

##### **Correspondence**: Andreas Eleftheriou, andelef2002@yahoo.gr

*Fluids and Barriers of the CNS* 2023, **20(Suppl 2)**: A135

**Introduction**: One of the key symptoms of Idiopathic Normal Pressure Hydrocephalus (iNPH) is urinary incontinence. However, there are very few studies focusing on this topic. The Japanese guidelines for management of iNPH describe that there is no sufficiently strong evidence to make recommendations regarding characteristics and evaluation of urinary incontinence. Furthermore, it is reported that patients with urinary incontinence also may have bowel incontinence. In this single-center prospective study we aimed to investigate urinary and bowel symptoms in patients with iNPH before and after the surgery and compared them with healthy individuals (HI).

**Methods**: Fifty-seven consecutive iNPH patients (27f, median age 77) were included. They underwent a pre- and 3-months clinical evaluation after shunt surgery. At the same time the following questionnaires were employed: the International Consultation on Incontinence Questionnaire – Urinary Incontinence Short Form (ICIQ-UI), the Wexner’s Fecal Incontinence Score (Wexner’s FI) and the Bowel Symptom Questionnaire. Forty-two healthy individuals, HI (25f, median age 71y) answered the questionnaires.

**Results**: The median disease duration was 24 months (6–120). Six patients (10.5%) had bladder disturbance as the first symptom, whereas 46 (80.7%) had gait/balance. The total Hellström scale score, the total ICIQ-UI score and the total bowel function satisfactory scale were significantly improved (p < 0.001, p = 0.01, p = 0.03). Significant differences were found in defecation time, occurrence of abdominal pain, soiling, stool retention time and bowel-related limitation in social life (p < 0.001, p = 0.04, p < 0.001, p = 0.04 and p = 0.03) between patients and HI. Eighty-three percent of patients released involuntary gas at least once per week (p < 0.001), while 12% used protection against stool leakage (p = 0.03) and 19% could not release gas without stool leakage (p = 0.04).

**Conclusion**: We expand the knowledge of urinary and bowel symptoms in iNPH with a more detailed description of the urinary symptoms. The patients also report several bowel symptoms, indicating bowel incontinence.

## A136 Temporo-spatial gait parameters alteration after tap test in patients with idiopathic normal pressure hydrocephalus (iNPH)

### Sunee Bovonsunthonchai^1^, Theerapol Witthiwej^2^, Roongtiwa Vachalathiti^1^, Sith Sathornsumetee^3^, Chanon Ngamsombat^4^, Orasa Chawalparit^4^, Weerasak Muangpisan^5^

#### ^1^Faculty of Physical Therapy, Mahidol University, Nakhon Pathom, Thailand; ^2^Division of Neurosurgery, Department of Surgery, Faculty of Medicine Siriraj Hospital, Mahidol University, Bangkok, Thailand; ^3^Division of Neurology, Department of Medicine, Faculty of Medicine Siriraj Hospital, Mahidol University, Bangkok, Thailand; NANOTEC-Mahidol University Center of Excellence in Nanotechnology for Cancer Diagnosis and Treatment, Faculty of Medicine Siriraj Hospital, Mahidol University, Bangkok, Thailand; ^4^Department of Radiology, Faculty of Medicine Siriraj Hospital, Mahidol University, Bangkok, Thailand; ^5^Department of Preventive and Social Medicine, Faculty of Medicine Siriraj Hospital, Mahidol University, Bangkok, Thailand

##### **Correspondence**: Sunee Bovonsunthonchai, sunee.bov@mahidol.edu

*Fluids and Barriers of the CNS* 2023, **20(Suppl 2)**: A136

**Introduction**: To investigate the alteration of gait parameters after CSF removal using a tap test in patients with idiopathic normal pressure hydrocephalus (iNPH).

**Methods**: Sixty-two patients with iNPH were recruited from the Orthopaedic Outpatient Clinic, Siriraj Hospital, Thailand. Eleven were excluded from unable to walk (n = 4), exhausted (n = 1), herpes zoster (n = 1), unable to communicate or follow commands (n = 2), gout (n = 1), severe back pain (n = 1), and unable to track the data (n = 1). Gait parameters were recorded at pre- and 24-h post-tap tests using an objective gait measurement platform with the self-selected speed for 3 trials. A physical therapist walked behind the patient to provide care or assistance as needed. To get rid of the effect of acceleration and deceleration, data at the middle part of the pressure mat were tracked and used in the analysis. The Paired t-test with p-value < 0.05 was used to compare data between pre- and post-tap tests.

**Results**: Fifty-one patients with iNPH completed the data collection protocol. Of these, 35 reported walking more easily, while the others felt the same (n = 10) or slightly diminished (n = 6). Comparisons of the data showed significant improvements in left and right step length and time, stride length and time, cadence, and velocity (p < 0.05) while there was no change in foot rotation angle, step width, stance phase, load response, single limb support, pre-swing, swing phase, and double limb support (p > 0.05).

**Conclusion**: Most patients enhanced their gait after the tap test as evidenced by the improvement of step/stride length and time, cadence, and velocity. However, the incremental improvements in stated variables are far from normal range values, and long-term effect is still needed in further study.

## A137 Expression analysis of motor activity related genes to predict idiopathic normal pressure hydrocephalus

### Madoka Nakajima^1^, Masakazu Miyajima^2^, Kaito Kawamura^1^, Chihiro Kamohara^1^, Ikuko Ogino^1^, Yumiko Motoi^3^

#### ^1^Department of Neurosurgery, Juntendo University, Tokyo, 113-0033, Japan; ^2^Department of Neurosurgery, Juntendo geriatric medical center, Tokyo, 136-0075, Japan; ^3^Department of Diagnosis, Prevention and Treatment of Dementia, Juntendo University Graduate School of Medicine, Tokyo, 113-0033, Japan

##### **Correspondence**: Madoka Nakajima, email address: madoka66@juntendo.ac.jp

*Fluids and Barriers of the CNS* 2023, **20(Suppl 2)**: A137

**Introduction**: The aim of this study is to investigate the expression of motor function related genes that can reflect motor activity intensity and detect pre-clinical stages of idiopathic normal pressure hydrocephalus (iNPH).

In a previous study, we performed an expression analysis of microRNAs (miR) in mouse hippocampus of an “exercise” group and found significantly increased miR-532-5P. We hypothesized that this gene may also be upregulated by exercise in humans, and investigated whether the expression of miR in plasma is affected. In addition, it can be predicted that early stages iNPH patients, who have significantly limited mobility, may have it as a biomarker for motor impairment.

**Method**: We measured miR-532-5P in plasma samples from iNPH patients (n = 32) and healthy elderly subjects (n = 20).

Next, in patients with iNPH who underwent shunt surgery (n = 10), we measured miR-532-5P expression before and at least 6 months after surgery in plasma and CSF samples, together with the more relevant Brain-Derived Neurotrophic Factor (BDNF), using ELISA.

**Results**: We found a significantly lower expression of miR-532-5P in plasma in the iNPH group than in the healthy elderly group, with a cutoff value of 11.2 in ΔCt value distinguishing between the two groups with 0.94 sensitivity and 0.8 specificity (AUC, 0.8). Furthermore, the expression of miR-532-5P in plasma increased after shunt surgery, indicating improved motor activity. miR-532-5P was increased after shunt treatment in iNPH patients, with pre- and postoperative BDNF increased 40% from 8.0 (ng/mL) to 10.8 in plasma and 70% from 0.7 to 0.94 in CSF.

**Conclusion**: Based on our findings, miR-532-5P can be a potential marker in iNPH patients for motor activity-induced upregulation. BDNF increased with miR-532-5P improvement and was thought to help improve activity and cognitive function.

## A138 Long-term Outcomes of Normal Pressure Hydrocephalus (NPH) Patients with CSF Shunt: A Systematic Literature Review and Meta-analysis

### Kim Wouters^1^, Savio Batista^2^, Luisa Glioche Gasparri^3^, Breno Rocha^2^, Raphael Bertani^4^, Fernando Pinto^4^

#### ^1^Open University of the Netherlands, Faculty of psychology, Heerle, the Netherlands; ^2^Federal University of Rio de Janeiro, Faculty of Medicine, Rio de Janeiro, Brazil; ^3^University Estácio de Sá, Faculty of Medicine, Rio de Janeiro, Brazil; ^4^Cerebral Hydrodinamics Group, Department of Neurosurgery, University of São Paulo, São Paulo, Brazil

##### **Correspondence**: Wouters Kim, k.wouters82@gmail.com

*Fluids and Barriers of the CNS* 2023, **20(Suppl 2)**: A138

**Introduction**: CSF shunt is the standard treatment for NPH and has been used for over 50 years with excellent results in the treatment of NPH but is not free of complications. The aim of this study was to review the long-term outcomes in patients treated with CSF shunt. Our second objective was to determine possible improvements in quality of life after shunt surgeries in patients with NPH.

**Methods**: We reviewed literature regarding long-term outcomes after shunt surgery in patients with NPH, following the PRISMA guidelines. Our search included PUBMED, ScienceDirect, Google scholar and Cochrane library databases. We did not include papers that report secondary causes of NPH and ages younger than 60 years.

**Results**: Out of 17000 articles, 12 were selected for analysis. The study involved 1402 treated patients who underwent CSF shunt surgery. The long-term outcomes were evaluated in 995 patients with a median time after surgery of 4 years and 2 months. The most reported initial symptoms were gait disorders (97%), followed by complaints related to cognitive impairment, (85%) and urinary incontinence (76%). After 12 months, global improvement was present in 80% of the patients. The most important complications listed in the studies are shunt dysfunction (22%), infections (7%), and subdural hematomas (3%).

**Conclusions**: Patients with clinical symptoms of NPH may benefit from a CSF shunt procedure for up to 6 years and 80% of the patients seem to experience an improvement in quality of life, even those with significant co-morbidities. Studies such as this one, are extremely relevant to further understanding the role of surgery for NPH patients. More studies are necessary to serve as basis for larger reviews and metanalysis on this.

**Keywords**: Normal pressure hydrocephalus, long-term, follow-up, clinical outcome, shunt surgery

## A139 The incidence of postdural puncture headache by lumbar puncture in patients with hydrocephalus is lower than in younger patients

### Masayuki Yamagishi, Takashi Kawahara, Masamichi Atsuchi

#### ^1^Department of Neurosurgery, Normal Pressure Hydrocephalus Center, Atsuchi Neurosurgical Hospital, Kagoshima, Japan

##### **Correspondence**: Masayuki Yamagishi, g.mira.2756@gmail.com

*Fluids and Barriers of the CNS* 2023, **20(Suppl 2)**: A139

**Introduction**: Postdural puncture headache (PDPH) is a complication that occurs in 10.9–29.5% of patients after lumbar puncture. Young age, female age, low body mass index, and history of headache have been reported as risk factors, but the pathogenesis is controversial. In our previous study, we suggested that younger patients have a larger intervertebral foramen cross-sectional area than older patients and are therefore more prone to cerebrospinal fluid (CSF) exudation. In the present study, we discussed PDPH in hydrocephalus patients, whose population is older.

**Methods**: We examined 283 patients (average age 78.7 years) who had undergone lumbar puncture at our hospital over a 3-year period, and selected those who presented with post-puncture headache. In addition, the cross-sectional area of the intervertebral foramen was measured by computed tomography (CT) in each case.

**Results**: PDPH had a low incidence in 4 of 283 patients (1.41%), all of whom were female, and the mean age was 76.8 years. When compared to the association between age and intervertebral foramen cross-sectional area in previous studies, the area tended to be smaller, again due to older age.

**Conclusions**: Few clinical studies focusing exclusively on elderly patients have been reported. Lumbar punctures in hydrocephalus patients, who are predominantly elderly, have a low incidence of PDPH. This suggests that CSF extravasation from the epidural space of the spinal canal through the intervertebral foramen into the paravertebral space is the mechanism that causes PDPH.

## A140 Contemporary Management of patients with Normal Pressure Hydrocephalus (NPH) – an International Survey

### Ganesalingam Narenthiran

#### ^1^Department of Neurosurgery, Manchester Centre for Clinical Neurosciences, Salford Royal Hospital, Greater Manchester, M6 8HD, United Kingdom

##### **Correspondence**: Ganesalingam Narenthiran, g_narenthiran@hotmail.com

*Fluids and Barriers of the CNS* 2023, **20(Suppl 2)**: A140

**Introduction**: The management of patients with suspected NPH has been controversial. The aim of the study was to ascertain current practice and provide data set for identifying future trials.

**Methods**: An online survey was created using SurveyMonkey with 60 questions. The questions included open , multiple choice and multiple response questions and logical branching. The survey distributed through Neurosurgery Research Listserv, British Neurosurgery Research Group Mailing list, neurosurgery Facebook groups (Neurosurgery Research Listserv, Neurosurgery Cocktail) and notice posted on LinkedIn and Twitter and emailed to leading neurosurgeons in the care of NPH around the world. The survey ran from 1/9/2022 to 9/12/2022. The data was downloaded from SurveyMonkey in .xlxs file. Then it was cleaned with MS Excel PowerQuery and MS Excel and imported into FileMaker 19 and then analysed with statistical software: Wizard 2 and Stata 15.

**Results**: There were 64 responses in total. Of the 44 (2/3) from 21 countries provided their localities. Of the 64 respondents, 60 (94%) obtained MRI of the head. Eleven percent restricted ancillary tests to ‘DESH negative’ patients. 75% respondents undertook Lumbar-tap test routinely and 31% undertook lumbar-drainage test routinely. Gait test was performed by neurosurgical staff in 44% of the patients. Regular NPH MDT was practiced in 20% of the practices. In 41% of the cases the ventricular catheter was inserted free-hand. Of those responded 45 inserted only VP shunts and 2 inserted only LP shunts for NPH.

**Conclusions**: The survey identifies important current trends in the management of NPH

## A141 Our preliminary experience on the combination of neuropsychological assessment and intrathecal pressure measurement to settle the diagnostic dilemma of the normal pressure hydrocephalus

### Gianpaolo Petrella, Silvia Ciarlo, Graziano Taddei, Angelo Pompucci, Alessandro Pesce

#### ^1^A.O. “Santa Maria Goretti” General Hospital, Neurosurgery Division, Latina, Italy

##### **Correspondence**: Gianpaolo Petrella, gianpaolo_p@hotmail.com

*Fluids and Barriers of the CNS* 2023, **20(Suppl 2)**: A141

**Introduction**: Normal-pressure hydrocephalus (NPH) is a common condition associated with cognitive deterioration and possibly involving 9% - 14% of all nursing home residents older than 65 years. The purpose of the present paper is to introduce an inclusive study protocol aimed at increasing the diagnostic precision and follow-up accuracy.

**Methods**: A total of 28 patients were operated on for NPH in our institution in the period ranging between January 2015 and December 2019. All the patients underwent MRI of the brain with standard sequences, calculation of the Evans index and corpus callosum angle, and evaluations by means of Montreal Cognitive Assessment (MOCA), Mini-Mental State Examination, and Frontal Assessment Battery (FAB) neuropsychological tests preoperatively and at 1 and 6 months. A preoperative lumbar test infusion (LIT) was performed.

**Results**: MOCA and FAB showed an overall improvement in the neurocognitive conditions at 1 month postoperatively. The mean pressure at the beginning of the LIT, was negatively associated with the neuropsychological outcome variables (Mini-Mental State Examination, FAB, and MOCA) in the 3 different evaluations, with FAB and MOCA at 6 months. We found a strong positive correlation between the Evans index as measured on the first magnetic resonance imaging scan with both the diastolic and systolic pressure at the beginning of the test.

**Conclusions**: Neuropsychological assessment, combined with LIT with intrathecal pressure management aids the diagnostic process in patients affected by NPH. It allows standardization in a rigorous fashion in the follow-up evaluation of patients undergoing surgery for a ventriculoperitoneal shunt.

## A142 A multidisciplinary team approach to managing normal pressure hydrocephalus: insights from a new service at the Royal Victoria Infirmary

### I. Coulter, L. Gourley, S. Surash, D. Butteriss, N. Warren

#### ^1^Royal Victoria Infirmary, Newcastle, UK

##### **Correspondence**: Ian Coulter, ian.coulter@doctors.org.uk

*Fluids and Barriers of the CNS* 2023, **20(Suppl 2)**: A142

**Introduction**: Determining the diagnosis of normal pressure hydrocephalus (NPH) and subsequently, which patients may respond to CSF diversion, can prove challenging due to the relatively high prevalence of mimicking conditions. Herein, we present our initial experience of delivering a multidisciplinary team approach to managing patients referred to the service in Newcastle upon Tyne.

**Methods**: We have performed an evaluation study in order to describe how the service has evolved. We have interrogated a prospectively maintained a database of patients assessed and managed since commencement of the service.

**Results**: The service was initiated in June 2021. Our NPH team consists of a neurologist, a neuroradiologist, CSF specialist nurse and a neurosurgeon. We conduct at least one MDT meeting per month as well as joint clinics bimonthly. 165 patients have been discussed in our MDT meeting. Radiological evaluation has included the assessment of the iNPH Radscale score for most of the patients referred. 54 new patients have attended our joint clinic for face-to-face assessment. We have employed a CSF tap test, which involves a) a videoed timed walking test and b) the Addenbrooke’s cognitive assessment, pre- and post-lumbar puncture, to try and determine which patients might respond to CSF diversion, in 40 cases. At the time of writing, 14 patients have undergone ventriculo-peritoneal shunt (VPS) insertion (which represents the treatment of choice at our centre) since inception of this service. Of these, 12 patients (86%) have experienced a positive neurological outcome (based on clinical assessment of gait and patient reported outcome) during a follow-up period of 1–18 months (one patient has yet to receive their postoperative review).

**Conclusions**: Our MDT approach allows the careful consideration of the patient’s diagnosis and suitability for CSF diversion treatment. This approach has led to a high rate of positive neurological outcomes following VPS insertion.

## A143 Variation of intracranial pressure and pulse amplitude amongst different types of hydrocephalic conditions and cerebrospinal fluid dynamic disorders

### Lucia Darie^1^, Jessica Mendall^2^, Nimeshan Chandra-Segaran^2^, Mary Thomas^2^, Aswin Chari^1^, Debayan Dasgupta^1^,Alexander Smedley^3^, Linda D’Antona^1^, Lewis Thorne^1^, Ahmed Toma^1^, Laurence Watkins^1^

#### ^1^Department of Neurosurgery, The National Hospital for Neurology and Neurosurgery, University College London Hospitals, London, United Kingdom; ^2^University College London Medical School, London, United Kingdom; ^3^Department of Neurosurgery, Leeds General Infirmary, Leeds, United Kingdom

##### **Correspondence**: Lucia Darie, darielucia@yahoo.com

*Fluids and Barriers of the CNS* 2023, **20(Suppl 2)**: A143

**Introduction**: Intraparenchymal intracranial pressure (ICP) monitoring is a useful took to provide insight and treatment guidance into various types of hydrocephalus and cerebrospinal fluid dynamic (CSF) disorder conditions. This study’s intention is to explore the variance of ICP and pulse amplitude (PA) amongst these different conditions and to investigate the correlation between ICP and PA.

**Methods**: This is single institution retrospective study analysing various parameters included in our departmental standard 24 h ICP monitoring. Demographic and radiological data were derived from the electronical charts. Meticulous recordings with a sampling frequency up to 40Hz included ICP, PA (separated into day, night and overall) however also peak ICP, percentage of time spend in negative and compliance. Principle component analysis was utilized to detect the key factors that contribute to the variability in data dispersion.

**Results**: Over 1200 individual 24 h recordings were analysed. These originated from over 900 patients, with additional 300 24 h data obtained during valve setting adjustments in the same cohort. A wide range of conditions were included such as: idiopathic intracranial hypertension, longstanding overt ventriculomegaly of adults, Chiari Malformation with or without previous surgery, low pressure states, and patients investigated with ICP monitoring for shunt dysfunction and arachnoid cysts. The mean age was 42.7 years (SD+/−14.91) with a good representation of both male and female patients. Significant variations were observed among the diagnostic groups when conducting exploratory comparisons of PA and ICP.

**Conclusion**: This research offers valuable information regarding intracranial pressure parameters observed among various patient groups diagnosed with CSF hydrodynamic disorders.

**Keywords**: hydrocephalus, CSF dynamic disorders, intracranial pressure, pulse amplitude

## A144 A decade of academic publications on NPH and IIH

### Linda D’Antona^1,2^, Eleanor Moncour^1,2^, Kanza Tariq^1,2^, Lucia Darie^1^, Lewis Thorne^1^, Laurence Watkins^1,2^, Ahmed Toma^1,2^

#### ^1^Victor Horsley Department of Neurosurgery, National Hospital for Neurology and Neurosurgery, London, WC1N3BG, UK; ^2^Queen Square Institute of Neurology, University College London, London, WC1N3BG, UK

##### **Correspondence**: Linda D’Antona, linda.d’antona@nhs.net

*Fluids and Barriers of the CNS* 2023, **20(Suppl 2)**: A144

**Introduction**: Normal Pressure Hydrocephalus (NPH) and Idiopathic Intracranial Hypertension (IIH) are disorders of the cerebrospinal fluid (CSF) dynamics that can be encountered by clinicians in neurology and neurosurgery clinical settings. The successful management of these diseases heavily relies on multidisciplinary efforts, with the neurologists often being in the position of making the initial diagnoses and referring to the neurosurgeons when appropriate. Academic publications in peer-reviewed journals should reflect this communal interest. This study is an analysis of peer-reviewed publications on NPH and IIH in the last decade.

**Methods**: Analysis of peer-reviewed literature. Standardised literature searches were conducted on PubMed to identify the number of publications that used the MeSH terms “Normal Pressure Hydrocephalus” or “Idiopathic Intracranial Hypertension” (or “Benign Intracranial Hypertension”) since 2012. The searches were conducted in predefined neurology and neurosurgery journals. The number of articles retrieved was quantified and compared to the total volume of papers published by the journals in the same years. A comparison in the proportion of publications about these diseases between neurology and neurosurgery journals was conducted.

**Results**: Publications in 7 neurosurgery journals and 8 neurology journals were analysed. A total of 89616 peer-reviewed papers were retrieved; of these, 53% were in journals mainly aimed at a neurosurgical audience. Amongst the retrieved articles, 386 used the NPH MeSH term (0.43%) and 186 the IIH MeSH term (0.21%). Over the years, there were clear oscillations in the number of papers published in each topic, but no definite trend. The proportion of publications on these topics was significantly higher in neurosurgery journals compared to neurology journals (Fisher’s exact test) for both NPH (p < 0.001) and IIH (p = 0.027).

**Conclusions**: Despite the fact that NPH and IIH are diseases managed in a multidisciplinary environment, these topics are less discussed in journals aimed at a neurology audience compared to the ones aimed at a neurosurgical readership.

## A145 Concussions and idiopathic normal pressure hydrocephalus: Is there a correlation?

### Mack J. Hancock, Gianluca Sorrento^2^, David Tang-Wai^2^, Carmela Tartaglia, Alfonso Fasano^1,2,3^

#### ^1^Edmond J. Safra Program in Parkinson’s Disease, Morton and Gloria Shulman Movement Disorders Clinic, Toronto Western Hospital, UHN, Toronto, Ontario; ^2^Krembil Brain Institute, Toronto, Ontario, Canada; ^3^Center for Advancing Neurotechnological Innovation to Application (CRANIA), Toronto, Ontario, Canada

##### **Correspondence**:Alfonso Fasano, alfonso.fasano@unh.ca

*Fluids and Barriers of the CNS* 2023, **20(Suppl 2)**: A145

**Introduction**: Traumatic brain injury (TBI) is a common cause of morbidity and mortality and some studies have also hypothesized a link between TBI and neurodegeneration. Normal Pressure Hydrocephalus (NPH) is a heterogeneous disorder with different etiology, including TBI. Some NPH patients have no apparent cause, thus are labelled as idiopathic (iNPH), a condition commonly underdiagnosed and poorly understood, especially with respect to its relation to neurodegeneration. Previous studies have shown that concussion or TBI can increase the risk of neurodegenerative diseases such as Alzheimers Disease (AD) and Parkinson’s (PD) (Fleminger et al., 2003; Bramlett & Dietrich., 2015) and the relationship between TBI and secondary NPH as well as the possible overlap between neurodegeneration and iNPH led us to the question of whether mild TBI plays a role in iNPH, an association unexpectedly not explored before. In this study we gathered data on the association between a history of TBI – even if mild – and later development of iNPH.

**Methods**: The history of head trauma was collected in a consecutive series of 54 iNPH patients by means of a case report form consisting of history of TBI assessment through the Ohio State University TBI-ID (OSU TBI-ID) (Corrigan, J. D., & Bogner, J. (2007) and Brain Injury Screening Questionnaire (BISQ) designed by the Icahn School of Medicine (Dams-O’Connor, K., et al., 2014) and compared with 50 patients diagnosed with Parkinson’s, as well as an age- and sex-matched control group of 40 healthy subjects.

**Results**: 74% of iNPH patients reported at least one minor head trauma throughout their lifetime, as opposed to 50% and 27.5% of patients diagnosed with Parkinson’s and healthy controls, respectively

**Conclusions**: Our preliminary findings indicate a possible association between TBI and iNPH, association needs to be further explored by future studies.

## A146 CSF biomarkers as predictive markers of outcome after CSF diversion in patients with normal pressure hydrocephalus

### Zeid A. Abussuud^1^, Jack Shepard^1^ Aleksandra B. Lasica^1^, Ahmed K. Toma^1^, Miles Chapman^2^

#### ^1^Victor Horsley Department of Neurosurgery, National Hospital for Neurology and Neurosurgery, University College London Hospitals, NHS Foundation Trust, Queen Square, London, UK; ^2^Department of Biochemistry, Institute of Neurology, University College London Hospitals, NHS Foundation Trust, Queen Square, London, UK.

##### **Correspondence**: Zeid A. Abussuud, z.abussuud@nhs.net

*Fluids and Barriers of the CNS* 2023, **20(Suppl 2)**: A146

**Introduction**: Biomarkers associated with neurodegeneration, obtained from CSF, may assist in the diagnosis of normal pressure hydrocephalus (NPH) as well as determining the prognostic benefit of CSF diversion treatment. We aim to assess the association of these CSF biomarkers with positive post-operative outcomes.

**Methods**: A retrospective analysis of patients that underwent CSF diversion at a single neurosurgical centre between 2009 and 2019 was conducted. CSF samples were obtained from either lumbar drainage (LD) or ventriculoperitoneal shunt (VPS) and were analysed for biomarkers, including Amyloid-β_1-42_ and Total-τ. Patients with insufficient documentation, incomplete biomarker data or no diagnosis of NPH were excluded. The associations between CSF biomarkers and post-operative outcomes were determined by Chi-Square test, Independent Sample T-test, and Pearson’s correlation.

**Results**: Our cohort comprised of 69 patients. 64 patients had LD and 52 had VPS insertion. The average value for Total-τ was 702pg/mL (range: 59–13,487pg/mL) and 29 (42.0%) patients had values outside the reference range. The average value for Amyloid-β_1-42_ was 595pg/mL (range 111–1339 pg/mL) and 13 patients (18.8%) had values outside the reference range. A strong positive correlation was found between the Total-τ value and the improvement of 10 metre walking test time post-LD at fast speed (*r* = 0.354, *n* = 41, *p* < 0.05), and post-VPS at normal speed (*r* = 0.329, *n* = 42, *p* < 0.05). Amyloid-β_1-42_ levels less than 450pg/mL were significantly associated with a greater improvement of 10 metre walking test time post-LD (t(54) = 2.098, *p* = 0.041) and subjective improvement in the patient’s symptoms (OR 6.790, *p* = 0.009).

**Conclusion**: Raised levels of CSF Total-τ and decreased levels of CSF Amyloid-β_1-42_ are significantly associated with post-operative improvement in mobility in NPH patients after CSF diversion. Further analysis is required to determine their association with positive neuropsychological and urological outcomes as well as their predictive value in clinical practice.

## A147 Non-Invasive ICP Waveform Monitoring for Assessment and Treatment Response in CSF Hypotension due to Spontaneous Occult CSF Fistulas: A Case Series

### Batista Savio^1^, Bertani Raphael^2^, Miranda Matheus^2^, Perret Caio^3^, Koester Stefan^4^, Guimarães Cavalcante Carlos de Carvalho Tamires^5^, A. S. Mendes Gabriel^2,6^, Oliveira Leonardo^7^, E. Bocanegra Jhon^8^, C. G. Pinto Fernando^2^

#### ^1^Faculty of Medicine, Federal University of Rio de Janeiro (UFRJ), Rio de Janeiro, RJ, Brazil; ^2^Cerebral Hydrodynamics Group, Department of Neurosurgery, Hospital das Clínicas, University of São Paulo, SP, Brazil; ^3^Department of Neurosurgery, Hospital Municipal Miguel Couto, Rio de Janeiro, RJ, Brazil; ^4^Medical School, Universidade Nove de Julho (UNINOVE), São Paulo, SP, Brazil; ^5^Medical School, University of Vanderbilt, Nashville, TN, USA; ^6^Physiotherapy Nucleous, Hospital of the State Public Servant of São Paulo, São Paulo, SP, Brazil; ^7^Faculty of Medicine, State University of Ponta Grossa, Ponta Grossa, Paraná, Brazil; ^8^Universidad Peruana Cayetano Heredia, Lima, Peru

##### **Correspondence**: Savio Batista, saviobatista360@gmail.com

*Fluids and Barriers of the CNS* 2023, **20(Suppl 2)**: A147

**Introduction**: Cerebrospinal fluid (CSF) hypotension due to spontaneous occult CSF fistulas is a condition that can cause debilitating symptoms such as headaches and neck pain. The non-invasive measurement of intracranial pressure waveform (nICPw) may provide a reliable way of assessing this condition. This study aims to evaluate the changes in P2/P1 ratios and time-to-peak (TTP) in different positions in patients with CSF hypotension due to spontaneous occult CSF fistulas and their response to treatment.

**Methods**: Three patients with CSF hypotension due to spontaneous occult CSF fistulas were evaluated using the P2/P1 ratio and TTP in different positions (supine, sitting, and standing) before and after treatment. All patients received specific treatment according to each case: one received an epidural blood patch, one received surgery and one received conservative treatment. The P2/P1 ratio was evaluated using a non-invasive device from Brain4care®.

**Results**: The results showed that the P2/P1 ratios in all three patients varied up to 1.5 and below 0.6 in different positions before treatment, with TTP varying from 0.06 to 0.40. After each patient received specific treatment, the P2/P1 ratios and TTP returned to closer-to-normal levels and less variation in all patients, in different positions, while symptoms also improved.

**Conclusions**: The present study suggests that the non-invasive ICP waveform and its associated parameters (P2/P1 ratio, TTP) may provide a reliable way of assessing CSF hypotension due to spontaneous occult CSF fistulas. The P2/P1 ratios and TTP in different positions varied before treatment but returned to closer-to-normal levels after treatment. These findings may help further understanding of other causes of CSF hypotension, such as shunt hyperdrainage. Further studies with larger sample sizes are needed to confirm these findings.

## A148 Exploring neurophysiology through monitoring the intracranial pressure (ICP) waveforms in shunted pregnant patients with a non-invasive device - a case series

### Gabriel A. S. Mendes^1,2^, Raphael Bertani^2^, Cintya Hayashi^2^, Gustavo H. Frigieri³, Rodolfo C. Reis^4^, Fernando C. G. Pinto^2^

#### ^1^Physiotherapy Nucleous,Hospital of the State Public Servant of São Paulo, São Paulo, SP, Brazil; ^2^Cerebral Hydrodynamics Group, Department of Neurosurgery, Hospital das Clínicas, University of São Paulo, SP, Brazil; ^3^Brain4Care, São Carlos, SP, Brazil; ^4^Neurosurgery Department, Hospital of the State Public Servant of São Paulo, São Paulo, SP, Brazil

##### **Correspondence**: Gabriel André da Silva Mendes, mendes1986@yahoo.com.br

*Fluids and Barriers of the CNS* 2023, **20(Suppl 2)**: A148

**Introduction**: Pregnancy can exacerbate the symptoms of idiopathic intracranial hypertension (IIH) and hydrocephalus due to increases in abdominal pressure. Non-invasive monitoring of intracranial pressure waveforms (ICPw) may provide insights into the neurophysiology of these conditions during pregnancy. The present case series describes two pregnant patients with IIH and hydrocephalus, respectively, who were monitored with a non-invasive device to assess changes in their P2/P1 ratios during pregnancy.

**Methods**: Two pregnant patients, one with IIH and one with hydrocephalus, were monitored with a non-invasive device to assess changes in their P2/P1 ratios during pregnancy. Symptoms were also evaluated.

**Results**: Both patients showed an increase in their P2/P1 ratios during pregnancy, which correlated with progressively worsening symptoms. After delivery, both patients showed improvement in symptoms, as well as a normalization of their P2/P1 ratios.

**Conclusions**: The present case series suggests that non-invasive monitoring of ICPw can provide insights into the neurophysiology of IIH and hydrocephalus during pregnancy. The increase in P2/P1 ratios during pregnancy may be indicative of worsening symptoms, and normalization of P2/P1 ratios after delivery may coincide with symptom improvement. Further studies with larger sample sizes are needed to confirm these findings and translate them into clinical use.

## A149 Patients with normal pressure hydrocephalus have fewer enlarged perivascular spaces in the centrum semiovale

### Aaron R. Switzer^1^, Jonathan Graff-Radford^1^, Jeffrey L. Gunter^2^, Benjamin D. Elder^3^, David T. Jones^1^, John Huston^4^, Clifford R. Jack^4^, Petrice M. Cogswell^4^

#### ^1^Department of Neurology, Mayo Clinic, Rochester, MN, 55905, USA; ^2^Department of Research Services, Rochester, MN, 55905, USA; ^3^Department of Neurosurgery, Mayo Clinic, Rochester, MN, 55905, USA; ^4^Department of Radiology, Mayo Clinic, Rochester, MN, 55905, USA

##### **Correspondence**: Aaron Ross Switzer, Switzer.aaron@mayo.edu

*Fluids and Barriers of the CNS* 2023, **20(Suppl 2)**: A149

**Introduction**: Enlarged perivascular spaces (ePVS) may be an indicator of glymphatic dysfunction. Limited studies have evaluated the role of ePVS in idiopathic normal pressure hydrocephalus (iNPH), with those studies showing conflicting results. We aimed to characterize the distribution and number of ePVS in iNPH compared to controls.

**Methods**: Thirty-eight patients with iNPH and a pre-shunt MRI were identified through clinical practice. Age- and sex-matched controls who had negative MRIs screening for intracranial metastases were identified through a medical record linkage system. Controls did not have cognitive impairment, gait disorder, or reported imaging features of iNPH. All patients had imaging performed on a 3T Siemens system. One reader counted ePVS in the basal ganglia (BG) and centrum semiovale (CS) on the T2 FSE sequence using the Wardlaw method blinded to clinical diagnosis. Imaging features of DESH, Fazekas white matter hyperintensity (WMH) grade, and the presence of microbleeds and lacunes were also evaluated. A second reader counted ePVS on a random sample of 20 participants to determine interrater agreement. The number of ePVS were compared between groups using an independent t-test. Linear regression models were performed in R.

**Results**: Each group had a mean age of 74±7 years and were 34% female with equal distributions of hypertension, dyslipidemia, diabetes, stroke, and history of smoking. Interrater agreement for ePVS grade was excellent (ICC 0.95, p < 0.001). There were fewer ePVS in the CS of patients with iNPH compared to controls (12.66 vs. 20.39, p < 0.001) but a similar number of ePVS in the BG (8.95 vs. 11.11, p = 0.08). This remained significant in models accounting for vascular risk factors (*p* = 0.005) and Evan’s index, HCTS, enlarged sylvian fissures, and WMH grade (*p* = 0.009).

**Conclusions**: Patients with iNPH had fewer ePVS in the CS but not in the BG suggesting that upward displacement of the brain may mechanically occlude PVS.

## A150 Beyond Antibiotics: The Therapeutic Benefits of Ventricular Irrigation for Ventriculitis

### Ahmed Al Menabbawy^1,2^, Ehab El Refaee^1,2^, Mohamed Elboradi^1^, Henry W. S. Schroeder^1,2^, Ahmed Zohdi^1^

#### ^1^Department of Neurosurgery, Cairo University, Cairo, Egypt; ^2^Department of Neurosurgery, University Medicine Greifswald, Greifswald, Germany

##### **Correspondence**: Ahmed Al Menabbawy, a.menabbawy@gmail.com

*Fluids and Barriers of the CNS* 2023, **20(Suppl 2)**: A150

**Introduction**: Ventriculitis poses a significant challenge in neurosurgical practice due to its unfavorable prognosis, protracted treatment duration, and extended hospitalization. The current standard of treatment has been limited to administering antibiotics and placement of external ventricular drains. However, while this approach is widely used, its efficacy is limited, and new treatment strategies are needed to improve outcomes for patients with ventriculitis. Here, we would like to introduce the different techniques of ventricular irrigation in cases of cerebral ventriculitis and the outcomes associated with this modality.

**Methods**: A total of 18 patients underwent ventricular irrigation for cerebral ventriculitis. Lavage/irrigation was performed endoscopically in 7 patients (3 patients required two burr holes while in 3 patients the irrigation was performed via one burr hole). In the remaining 12 patients, the irrigation was done without using the endoscope. The outcomes were compared regarding infection resolution and Hospitalization time and Glasgow Outcome Scale (GOS).

**Results**: Patients’ mean age was 116.7 months ± 17.9 [SE]. They were followed up to a mean duration of 8.6 months ± 4.5 [SE] months. GOS was more than 4 (good outcome) in (12/18) 66.7 % of the cases. Mean hospital stay duration was 23.6 days ± 4.8 [SE]. Infection resolution was achieved on average in 17 days ± 4.8 [SE].

**Conclusion**: In the management of cerebral ventriculitis, ventricular lavage has emerged as a valuable treatment modality, offering improved outcomes and shorter hospital stays compared to traditional therapies. These findings highlight the potential of ventricular lavage as a promising approach to the management of ventriculitis and underscore the need for further research to better understand its benefits and limitations.

## A151 Exteriorization of a ventriculoperitoneal shunt through the urethra in a pediatric patient: literature review and case report

### Silvio P. de O. Junior^1^, Willian W. B. Medeiros, Leonardo M. de Avellar^1^

#### ^1^Neurosurgery, Hospital Geral Roberto Santos, Salvador, Bahia, 40301-110, Brazil

##### **Correspondence**: Silvio Porto de Oliveira Junior; SilvioJr1212@gmail.com

*Fluids and Barriers of the CNS* 2023, **20(Suppl 2)**: A151

**Introduction**: Ventriculoperitoneal shunt surgery is a procedure indicated for hydrocephalus, in which the cerebrospinal fluid is diverted from the ventricles to the peritoneum. There are several complications related to this surgery, which are common in surgical practice. One of the rarest being the exteriorization of the “shunt” through the urethra.

**Methods**: To investigate the literature of exteriorization of a ventriculoperitoneal shunt through the urethra, a search was carried out in the MEDLINE/PUBMED database with the following descriptors: ((ventriculoperitoneal shunt[Title/Abstract])) AND (urethral)[Title/Abstract] ). Filters were not used, and 20 papers were found between 1992 and 2023, but after reviewing the papers, only 14 were selected for the study, ranging in depth in each one, from epidemiology, etiology, clinical presentation to surgical correction. Case presentation: Female patient, 03 years old, with myelomeningocele corrected at birth, with hydrocephalus, submitted to the ventriculoperitoneal shunt procedure, which complicates the exteriorization of the "shunt" to the urethra. 1 month after the complication, a ventriculoatrial shunt is performed as an alternative to treat hydrocephalus.

**Results**: The first work that brought urethral exteriorization as a complication of peritoneal diversion was published in 1995. This complication occurs more in female patients, in a ratio of 1.1:1, with a mean age of 6.8 years, in which it is more common during childhood, but can occur during adulthood, in addition to being more common. rare in neonates. Most common clinical presentation is hematuria, dysuria, abdominal pain and neurological symptoms, infection is the major complication in these cases. Surgical management consists of removing the catheter, preferably with disconnection of the drainage system.

**Conclusions**: As rare as such a complication may be, it is essential to understand its risks, so that measures and care are taken in order to reduce the chance of this occurring, aiming at a better prognosis for the patient.

## A152 Deaths due to hydrocephalus treatment in brazil from 2012 to 2022

### Silvio P. de O Junior^1^, João V. M. P. de Oliveira^2^ (Presenting author underlined)

#### ^1^Neurosurgery, Hospital Geral Roberto Santos, Salvador, Bahia, 40301-110, Brazil; ^2^Neurosurgery, Irmandade da Santa Casa de Misericórdia de São Paulo, São Paulo, São Paulo, 01221-020, Brazil

##### **Correspondence**: Silvio Porto de Oliveira Junior; SilvioJr1212@gmail.com

*Fluids and Barriers of the CNS* 2023, **20(Suppl 2)**: A152

**Introduction**: Scientifically, hydrocephalus is defined as the excess accumulation of cerebrospinal fluid in the intracranial spaces, generating enlargement of the ventricular system and intracranial hypertension with their respective consequences. This condition is responsible for both deaths and disability of individuals and can affect both adult and pediatric patients. The treatment has a good rate of positive outcome, but there are complications such as infection and obstruction of the system. This study aims to analyze the rate of deaths from hydrocephalus treatment in Brazil from 2012 to 2022 and seek to understand the variation in numbers over the years.

**Methods**: This is a quantitative, ecological, descriptive study, based on data reported in the Notifiable Diseases Information System (SINAN/SUS) of the Ministry of Health through DATASUS.

**Results**: In 2012, 3,589,149 patients with hydrocephalus were treated, while 170 died after treatment. In 2017, 6,575,832 patients underwent treatment for hydrocephalus, with the peak in the number of deaths, which reached 2015. In 2022, the number of treatments practically doubled, 6,819,129, but the number of deaths did not follow the same pattern , remaining at 196.

**Conclusion**: The number of records of treated patients increased over the years, most likely due to advances in patient recording techniques, and not due to a disease condition that increased over time. It is possible to verify that the death/treatment ratio decreased over time, which represents something positive and reflects advances in surgical techniques and measures to reduce complications, such as infection and obstructions.

## A153 Ventriculo-gallbladder shunt: literature review and case report

### Silvio P. de O Junior, Matheus da Paz, Joao Ramos, Beatriz da Cunha, Leonardo Avellar

#### ^1^Neurosurgery, Hospital Geral Roberto Santos, Salvador, Bahia, 40301-110, Brazil

##### **Correspondence**: Silvio Porto de Oliveira Junior; SilvioJr1212@gmail.com

*Fluids and Barriers of the CNS* 2023, **20(Suppl 2)**: A153

**Introduction**: Permanent liquor diversion is associated with high risk of failure and often needs re-intervention. Ventriculo-gallbladder shunt (VGS) has been recognized as a last-resort alternative to treat hydrocephalus when the peritoneum or other distal sites can no longer receive shunts. This article’s purpose is to report a case from a neurosurgery referral service in Brazil and review literature on that issue.

**Methods**: A search was carried out in the MEDLINE/PUBMED database with the following descriptors: ((ventriculo gallbladder shunt [Title/Abstract])) OR ((ventriculo-gallbladder shunt[Title/Abstract])). Filters were not used. Nine papers were found between 1997 and 2023, but, during the analysis of the papers, only 7 were selected for the study. Case presentation: G.B.S, male, 43 years old, no comorbidities, with a 12-year history of hydrocephalus, with post-surgical chronic fungal meningitis. He underwent a ventriculoatrial shunt (VAS) placement 2 years ago, as he had multiple ventriculoperitoneal shunt (VPS) failures. Endocarditis was suspected and the VAS was withdrawn. As an alternative, VGS was placed 6 months ago and, since then there has been no reason for a new system review.

**Results**: The first work was published in 1997 and brought the gallbladder as an alternative since it would serve as a temporary receptacle for liquor, just as the peritoneal cavity. The gallbladder has an absorptive capacity of 1500 cc liquid daily, which comprises more than the normal CSF daily production. Therefore, it’s a good alternative when the ventriculo-peritoneal shunt is not viable due to postsurgical peritoneal adhesions or when there are contraindications for ventriculoatrial or ventriculopleural Shunts.

**Conclusions**: VGS is an alternative to patients who cannot undergo the most common surgical interventions, such as VPS. The case reported here is a good illustration of that: VGS was placed as an alternative, with no complications since then, suggesting that it can be a viable option in some cases.

## A154 Managing Shunt Dysfunction with Non-invasive Intracranial Pressure Waveform Monitoring

### Bertani Raphael^1^, Batista Savio^2^, Miranda Matheus^1^, Perret Caio^3^, Koester Stefan^4^, Guimarães Cavalcante Carlos de Carvalho Tamires^5^, A. S. Mendes Gabriel^1,6^, Oliveira Leonardo^7^, Santa Maria Paulo^3^, C. G. Pinto Fernando^1^

#### ^1^Cerebral Hydrodynamics Group, Department of Neurosurgery, Hospital das Clínicas, University of São Paulo, SP, Brazil; ^2^Faculty of Medicine, Federal University of Rio de Janeiro (UFRJ), Rio de Janeiro, RJ, Brazil; ^3^Department of Neurosurgery, Hospital Municipal Miguel Couto, Rio de Janeiro, RJ, Brazil; ^4^Medical School, Universidade Nove de Julho (UNINOVE), São Paulo, SP, Brazil; ^5^Medical School, University of Vanderbilt, Nashville, TN, USA; ^6^Physiotherapy Nucleous, Hospital of the State Public Servant of São Paulo, São Paulo, SP, Brazil; ^7^State University of Ponta Grossa, Faculty of Medicine, Ponta Grossa, Paraná, Brazil

##### **Correspondence**: Savio Batista, saviobatista360@gmail.com

*Fluids and Barriers of the CNS* 2023, **20(Suppl 2)**: A154

**Introduction**: Shunt dysfunction is a potentially severe complication of ventriculoperitoneal shunt (VPS) surgery for hydrocephalus, and, while it can lead to debilitating and worsening symptoms, it may also present with vague or non-specific symptoms. Non-invasive intracranial pressure waveform monitoring (nICPw) is a method that can be used to help diagnose shunt dysfunction and assess patient improvement. The present case report describes a patient with previous hydrocephalus who presented with shunt dysfunction for which nICPw was used during all stages of management.

**Methods**: A 30-year-old female with a history of hydrocephalus presented with vision loss after encephalitis. CT scans showed mild increase in ventricle size, nICPw showed an increased P2/P1 ratio and a shunt replacement was performed. After surgery, the patient's vision improved but she began experiencing hyperdrainage symptoms. A programmable shunt was placed, and nICPw was used to assess shunt pressure adjustment and patient improvement.

**Results**: The nICPw was useful in diagnosing shunt dysfunction and determining a P2/P1 ratio baseline. After the placement of the programmable shunt, the patient's hyperdrainage symptoms improved, and nICPw was used to assess shunt pressure adjustment and patient improvement.

**Conclusions**: The present case report suggests that nICPw can be a useful tool in the management of shunt dysfunction in patients with hydrocephalus. It can aid in the diagnosis of shunt dysfunction, assessment of patient improvement, and shunt pressure adjustment. Informed consent to publish has been obtained by the patient.

## A155 Trends in hydrocephalus and CSF dynamics disorders research: A qualitative analysis of the Hydrocephalus Society abstracts

### Linda D’Antona^1,2^, Eleanor Moncour^1,2^, Kanza Tariq^1,2^, Lucia Darie^1^, Laurence Watkins^1,2^, Ahmed Toma^1,2^, Lewis Thorne^1^

#### ^1^Victor Horsley Department of Neurosurgery, National Hospital for Neurology and Neurosurgery, London, WC1N3BG, UK; ^2^Queen Square Institute of Neurology, University College London, London, WC1N3BG, UK

##### **Correspondence**: Linda D’Antona, linda.d’antona@nhs.net

*Fluids and Barriers of the CNS* 2023, **20(Suppl 2)**: A155

**Introduction**: Hydrocephalus and cerebrospinal fluid (CSF) dynamics disorders are a field of rapidly growing research interest. This qualitative research investigates the trends in this field through the meetings of the International Society for Hydrocephalus and Cerebrospinal Fluid Disorders (ISHCFD).

**Methods**: This is a retrospective qualitative analysis of the published abstracts of the ISHCFD meetings. The data collection covered the period between 2015 and 2022. Key terms were identified through the screening of the latest (2022) abstracts book and reviewed by the authors. A word frequency analysis of the preselected words/terms was conducted for each year and quantified (word frequency proportion = N. word was mentioned in the abstract book / N. abstracts published). Terms indicating similar concepts were consolidated (e.g., ‘NPH’ and ‘Normal Pressure Hydrocephalus’), while terms with partial overlap separated (e.g., ‘Hydrocephalus’ separated from ‘Normal Pressure Hydrocephalus’).

**Results**: A total of 774 abstracts, published for 7 ISHCFD meetings were analysed. 122 pre-selected terms were searched and only the most frequent selected for further analysis. The most represented diseases were Normal Pressure Hydrocephalus (NPH) (43.9%) and hydrocephalus (42.3%), followed by idiopathic intracranial hypertension (10.3%), Chiari malformation/syringomyelia (2.7%), LOVA (0.5%) and spontaneous intracranial hypotension (0.4%). There was a steady increase in the interest in Chiari malformation since 2017. Another interesting trend was the decrease in reports about hydrocephalus relative to NPH between 2015 and 2016. The analysis of investigations revealed a growing interest in biomarkers, optic nerve assessments and the DESH sign, in addition a steady decline of lumbar punctures was observed since 2019. The analysis of treatments confirmed ventriculoperitoneal shunts as the most popular (41%) followed by stenting (26%), lumboperitoneal shunts (20%) and ventriculoatrial shunts (11%); however, a significant steady decline was noted in the interest in stenting from 2016.

**Conclusions**: This qualitative research highlighted interesting trends in the research for hydrocephalus and CSF disorders.

## A156 How change the vestibulo-oculomotor reflex (VOR) in normal pressure hydrocephalus (NPH) shunted patients: study on the use the Video Head Impulse

### Gianpaolo Petrella, Silvia Ciarlo, Graziano Taddei, Angelo Pompucci, Alessandro Pesce

#### ^1^ “Santa Maria Goretti” Hospital, Neurosurgery Division, Latina, Italy

##### **Correspondence**: Gianpaolo Petrella, gianpaolo_p@hotmail.com

*Fluids and Barriers of the CNS* 2023, **20(Suppl 2)**: A156

**Introduction**: Normal pressure hydrocephalus is a progressive, chronic, extremely complex syndrome. It is the most common form of reversible dementia in the elderly, it is characterized by gait disturbance, dementia, and urinary incontinence.

**Methods**: We firstly subjected 9 patients, with symptoms of NPH, to a lumbar infusion test, which was positive documenting an elastance index greater than 0.3. The patients underwent a video Head Impulse Test before surgery, 7 days after and one month after surgery. The exam provides the objective measurement of the vestibulo-oculomotor reflex (VOR) at high frequencies and allows, by making small movements of the head in various directions, a study of the functionality of the pairs of semicircular canals. The software records the reflex eye movements originating from the VOR and compares them with the passive movements of the head, thus providing the gain of the VOR. The aim was to confirm the importance of the index of elastance as a positive predictive value of response to surgery, using the vHIT test.

**Results**: Our preliminary results showed a generally reduced gain in the preoperative phase and an increase in the postoperative gain, detectable already at the first check-up.

**Conclusions**: Firstly, it is conceivable that the chemical-physical variations of the CSF attributable to hydrocephalus and the subsequent variations induced following the peritoneal derivation can induce variations in the pressure and in the composition of the endolymph with consequent repercussions on the canal function. This data confirms the importance of the infusion test in the diagnosis of patients with normal pressure hydrocephalus.

## A157 The modified frailty index (mFI11) as a Predictor of Post-Operative Infections in Ventriculo-Peritoneal Shunt Surgery

### Francesca Marotta^1,2^, Daniele Piccolo^1,2,3^, Sara Fabbro^1,4^, Andrea Valenti^1,2^, Claudio Giuseppe Serio^1,2^, Daniele Bagatto^5^, Enrico Belgrado^6^, Miran Skrap^1^, Francesco Tuniz^1^

#### ^1^Neurosurgery Unit, Udine University Hospital, Udine (UD), 33100, Italy; ^2^Department of Neuroscience, University of Padua, Padova (PD), 35121, Italy; ^3^Department of Clinical, Diagnostic and Pediatric Sciences, University of Pavia, Pavia (PV), 27100, Italy; ^4^Neurosurgery Unit, Trieste University Hospital, Trieste (TS), 34128, Italy; ^5^Neuroradiology Unit, Udine University Hospital, Udine (UD), 33100, Italy; ^6^Neurology Unit, Udine University Hospital, Udine (UD), 33100, Italy

##### **Correspondence**: Daniele Piccolo, ing.daniele@gmail.com

*Fluids and Barriers of the CNS* 2023, **20(Suppl 2)**: A157

**Introduction**: This study aimed to investigate the correlation between the modified frailty index (mFI11) and the rate of postoperative infectious complications in patients with hydrocephalus who underwent ventriculoperitoneal shunt (VPS) placement.

**Methods**: We conducted a retrospective analysis of 367 consecutive patients with normal pressure or hydrocephalus who underwent VPS placement at our center between 2015 and 2022. Descriptive statistics were used to characterize the study population, and the correlation between mFI11 and the incidence of postoperative infectious complications was assessed using logistic regression analysis. The effects of age, surgical time, and diagnosis were also examined.

**Results**: Of the 367 patients, 360 did not experience any postoperative infectious complications, whereas 7 did (1.9%). The mean mFI11 score was significantly higher in patients who experienced postoperative infectious complications (mean 3, SD 1.7) compared to those who did not (mean 1.3, SD 1.2) (F = 12.353, p < 0.01). In the multivariate logistic regression analysis, mFI11 was found to be a significant predictor of postoperative infectious complications (OR = 2.18, 95% CI: 1.45–3.27, p < 0.01), while age, surgical time, and diagnosis were not.

**Conclusions**: Our study suggests that mFI11 is a valuable tool for predicting the incidence of postoperative infectious complications in patients who undergo VPS placement. It appears that mFI11 is a more important predictor than age, surgical time, or diagnosis in this regard. Further studies are needed to validate these findings and to determine if interventions to improve frailty may decrease the risk of postoperative infections in this patient population.

## A158 Callosal angle is more useful than Evans index for postoperative follow-up of normal pressure hydrocephalus

### Hisayuki Murai

#### ^1^Department of Neurosurgery, Saiseikai Narashino Hospital, Narashino City, Chiba, 275-8580, Japan

##### **Correspondence**: Hisayuki Murai, murai@chiba-saiseikai.com

*Fluids and Barriers of the CNS* 2023, **20(Suppl 2)**: A158

**Introduction**: When symptoms that had once improved after shunt surgery worsen again in iNPH patients, it is difficult to determine whether the cause is due to shunt dysfunction, progression of comorbidities, or development of a new disease. We examined the image findings of head CT or MRI to find shunt dysfunction more efficiently, based on changes in postoperative image findings and actual cases of shunt dysfunction.

**Methods**: From 2020 to 2021, 66 patients diagnosed with iNPH underwent shunt surgery at our hospital. Forty-five patients were included in the study, excluding eleven patients who were unable to evaluate neurological symptoms and MRI at 6 months after surgery. Before the tap test and 6 months after the shunt operation, Timed Up & Go Test (TUG), Evans index, and the callosal angle were evaluated. Those changes were also evaluated in a shunt malfunction case. **Results**: TUG showed significant improvement overall, but slight change in mild cases. There was also a significant decrease in the Evans index, but the amount of change was less than 1% in 47% of the cases. There was also a significant distinct angular enlargement of the callosal angle, with an average difference of 18.6 degrees. The change of callosal angle was also distinct in a shunt malfunction case.

**Conclusions**: The change in callosal angle was more obvious at postoperative follow-up. It was suggested that focusing on the callosal angle may make it easier to notice shunt malfunction.

## A159 Non-invasive P2/P1 Ratio Analysis as a Potential Auxiliary Diagnostic and Follow-up Criterion for Normal Pressure Hydrocephalus in Patients Undergoing Ventriculoperitoneal Shunt Surgery

### Raphael Bertani^1^, Savio Batista^2^, Matheus Miranda^1^, Caio Perret^3^, Stefan Koester^4^, Gustavo H Frigieri^5^, Fernando C G Pinto^1^, Jefferson Chen^6^

#### ^1^Cerebral Hydrodynamics Group, Department of Neurosurgery, Hospital das Clínicas, University of São Paulo, SP, Brazil; ^2^Medical School, Federal University of Rio de Janeiro (UFRJ), Rio de Janeiro, RJ, Brazil; ^3^Department of Neurosurgery, Hospital Municipal Miguel Couto, Rio de Janeiro, RJ, Brazil; ^4^Medical School, University of Vanderbilt, Nashville, TN, USA; ^5^brain4care, São Carlos, SP, Brazil; ^6^Neurosurgery Department, University of California Irvine (UCI) Irvine, CA, USA

##### **Correspondence**: Raphael Bertani, contato@rbertani.com

*Fluids and Barriers of the CNS* 2023, **20(Suppl 2)**: A159

**Introduction**: Normal Pressure Hydrocephalus (NPH) is a condition characterized by changes in intracranial compliance. Ventriculoperitoneal shunt (VPS) surgery is a common treatment for NPH, which involves the insertion of a shunt to drain excess cerebrospinal fluid. The present study aims to assess the performance of the P2/P1 ratio of the intracranial pressure waveform, obtained through a non-invasive device, as a diagnostic criterion for NPH in patients undergoing VPS surgery.

**Methods**: Seven patients with NPH were included in this study and were evaluated using the P2/P1 ratio before and after VPS surgery. The non-invasive evaluation of intracranial compliance was performed in the lying, sitting or 30 degrees inclination and upright positions, and the P2/P1 ratio was evaluated pre- and post-surgery.

**Results**: Most patients showed higher P2/P1 ratios, higher time to peak (TTP), smaller number of valid waveforms and a worse confidence interval before shunt surgery, with improvement of parameters correlating with symptom improvement, as well as initial symptoms. The lying position seems to be more consistent in showing highest P2/P1 values, especially in the post-shunt monitorization sessions.

**Conclusions**: The present study suggests that the P2/P1 ratio may be a valid non-invasive diagnostic and follow-up criterion for NPH, as well as a helpful adjunct for shunt pressure adjustment. Although differences in before and after shunt was found, optimization of monitorization routines is still necessary so that potential cut-off values can be found . Larger studies and clinical trials are required to further understand the diagnostic and follow-up potential of non-invasive waveform analysis for the diagnosis of NPH.

## A160 Do Evans index and callosal angle correlate with neurological improvement after shunt surgery in idiopathic normal pressure hydrocephalus?

### Rodolfo C. Reis^1^, Renata H. B. Yamashita^2^, Davi J. F. Solla^2^, Laís F. Ramin^3^, Tomás de A. L. Freddi^4^, Manoel J. Teixeira^2^, Fernando C. G. Pinto^2^.

#### ^1^Department of Neurosurgery, IAMSPE, São Paulo, SP, Brazil; ^2^Department of Neurosurgery, Hospital das Clínicas, São Paulo, SP, Brazil; ^3^Neuroradiology Section, Hospital das Clínicas, University of São Paulo, SP, Brazil; ^4^Neuroradiology Section, Hcor, São Paulo, SP, Brazil

##### **Correspondence**: Rodolfo Casimiro Reis, rodolfocr84@yahoo.com.br

*Fluids and Barriers of the CNS* 2023, **20(Suppl 2)**: A160

**Introduction**: Idiopathic normal pressure hydrocephalus (iNPH) is a syndrome characterized by gait disturbance, cognitive impairment, and urinary incontinence, affecting mainly the elderly. Although Evans index (EI) and callosal angle (CA) are important radiological markers for its diagnosis, little is known about their changes after ventriculoperitoneal shunt (VPS) surgery among these patients. Here we aim to investigate whether EI and CA change after VPS and if these differences correlate with clinical symptoms.

**Methods**: A prospective cohort of iNPH patients treated with Sphera Pro® programmable valve was followed for one year. Clinical improvement, measured by NPH Japanese Scale, EI and CA, measured by CT or MRI, were registered and compared pre- and postoperatively (3, 6 and 12 months).

**Results**: A total of 19 patients with probable iNPH were prospectively recruited. NPH Japanese Scale decreased over time (p < 0.001) from 6.0 (±2.0) preoperatively to 4.4 (±2.6), 4.0 (±2.2) and 4.1 (±2.2), 3, 6 and 12 months postoperatively (respectively) . CA was smaller (72±15°) preoperatively than postoperatively: 72±15° in 3, 91±18° in 6 and 91±18° in 12 months after surgery, p < 0.01. EI did not change with surgery (p = 0.495): 0.34 (±0.04) preoperatively, 0.34 ± 0.02 in 3, 0.33 ± 0.03 in 6 and 0.33 ± 0.04 in 12 months after VPS.

**Conclusions**: In this sample, EI did not change after surgery, and clinical improvement of NPH symptoms occurred concurrently with an increase in CA value. This indicates that CA value could be evaluated further as an indirect method to determine response to treatment and shunt function in patients with iNPH.

## A161 Development of web-apps for calculating Evans Index, iNPH Radscale, iNPH Scale, ETVSS

### Ganesalingam Narenthiran

#### ^1^Department of Neurosurgery, Manchester Centre for Clinical Neurosciences, Salford Royal Hospital, Greater Manchester, M6 8HD, United Kingdom

##### **Correspondence**: Ganesalingam Narenthiran: g_narenthiran@hotmail.com

*Fluids and Barriers of the CNS* 2023, **20(Suppl 2)**: A161

**Introduction**: Surgeons and physicians managing patients with hydrocephalus and in particular complex or normal pressure hydrocephalus often use Evans’ Index, iNPH Radscale, iNPH scale ETVSS. A calculator for these scales on the web would be useful and efficient in undertaking these calculations. Traditionally web-app development had required information technology (IT) specialists with expertise in this area. The aim of the project was for neurosurgeons to develop these web-apps without help from IT specialists.

**Methods**: Two softwares were used for web-app development. They were Xojo and Notepad (for JavaScript). The Evans Index, iNPH RadScale and iNPH scale calculators were developed with Xojo developer software. The developed softwares were deployed onto Xojocloud server. NotePad was used to write the code in JavaScript for the ETVSS score. The JavaScript was then published on the web using Adobe Dreamweaver software on a web-host.

**Results**: The Evans’ Index, iNPH Radscale, iNPH scale were successfully developed with Xojo and deployed in Xojocloud. The ETVSS calculator was successfully developed and deployed on a web-host. The development using Xojo and Xojo cloud is convenient, and the resultant app is appealing but, relatively expensive. The development of web-software with JavaScript has a more steeper learning curve but is economical. The web-apps can be accessed from https://neuro1.xojocloud.net

**Conclusions**: Web-apps useful for physicians involved in the management of complex hydrocephalus can be developed by physicians themselves using user friend software development platforms.

## A162 Verification of the Stroop test Application Software for patients of idiopathic Normal Pressure Hydrocephalus (NPH)

### Chihiro Kamohara^1^, Madoka Nakajima^1^, Kaito Kawamura^1,2^, Shigeki Yamada^3^, Yukihiko Aoyagi^4^, Chihiro Akiba^5^, Koichiro Sakamoto^1^, Kou Horikoshi^1,2^, Ryo Miyahara^1^, Ikuko Ogino^1^, Kostadin Karagiozov^1^, Masakazu Miyajima^5^, Akihide Kondo^1^

#### ^1^Department of Neurosurgery, Juntendo University School of Medicine, Tokyo, Japan; ^2^Department of Neurosurgery, Saiseikai Kawaguchi General Hospital, Saitama, Japan; ^3^Department of Neurosurgery, Nagoya City Hospital, Nagoya, Japan; ^4^Digital Standard Co., Ltd., Osaka, Japan; ^5^Department of Neurosurgery, Juntendo Koto Geriatric Medical Center, Tokyo, Japan

##### **Correspondence**: Chihiro Kamohara, c-kamohara@juntendo.ac.jp

*Fluids and Barriers of the CNS* 2023, **20(Suppl 2)**: A162

**Introduction**: The cognitive deterioration of iNPH includes memory impairment, frontal lobe dysfunction, psychomotor slowing, and attention deficits. Hellström’s EU-iNPH grading scale thoroughly includes neuropsychological tests that measure all these aspects. We have previously reported that the Stroop test (color naming and interference tasks) which targets psychomotor speed and executive function, was valid in detecting comorbidity of iNPH such as Alzheimer’s disease or Parkinson’s spectrum (PS) diseases. However, neuropsychological tests are time-consuming and could become a burden for both patients and examiners. This study aims to evaluate and compare the clinical application of a newly created Stroop test Application Software to the Hellström’s Swedish Stroop test.

**Methods**: The Application Software (Hacaro-Stroop test) that includes 20 items for each task was applied for testing on a tablet device. Patients diagnosed with iNPH at the Juntendo University Hospitals participated. In Study-1, a relationship between the two test methods was evaluated on 92 patients using correlation analysis. In Study-2, the prognostic value of the tap-test results from the Application Software was analyzed in 30 patients who underwent both tap-test and CSF shunt surgery through correlation analysis and ROC analysis.

**Results**: In Study-1, the Swedish Stroop Test and the Application Software had a strong correlation, especially in the color naming task (r = 0.77, p<.001). In Study-2, applying the Application Software, there was a strong correlation between the result of the tap test and the CSF shunting outcome from the color naming task (r = 0.75, p<.001). The ROC analysis indicated that improvement after the CSF shunting could be predicted by the tap test result (AUC = .76, p = .02, cut-off = 1.9, Sensitivity = .70, Specificity = .70).

**Conclusions**: The Application Software was successful in shortening the time taken for the test compared to the Swedish Stroop Test. The Application Software may be useful in the pre-tap test evaluation as a substitute for the Swedish Stroop Test.

## A163 Evaluation of non-invasive ICP waveform and intracranial compliance monitoring technology in a pediatric neurosurgical outpatient clinic

### Nelci Zanon^1^; Felipe O. Magalhães^1^; Rodrigo A. Watanabe^1^; Pedro J. R. Muiños^1^; Gustavo Frigieri^2^; Sávio Batsita^3^, Raphael Bertani^4^

#### ^1^Department of Neurosurgery, Universidade Federal de São Paulo (UNIFESP), São Paulo, SP, Brazil; ^2^Medical Investigation Laboratory 62, School of Medicine, University of São Paulo, São Paulo, Brazil; ^3^Medical Student, Federal University of Rio de Janeiro (UFRJ), Rio de Janeiro, RJ, Brazil; ^3^Cerebral Hydrodynamics Group, Department of Neurosurgery, Hospital das Clínicas, University of São Paulo, SP, Brazil

##### **Correspondence**: Nelci Zanon, nelcizanon@terra.com.br

*Fluids and Barriers of the CNS* 2023, **20(Suppl 2)**: A163

**Introduction**: Diagnosis and follow-up of neurosurgical patients is often challenging, particularly for children. This is a preliminary work of incorporating a non-invasive tool for monitoring the intracranial pressure waveform (nICPw) and therefore estimate intracranial compliance (IC). The study was conducted with 20 patients from the pediatric outpatient neurosurgical clinic at the São Paulo Hospital and Universidade Federal de São Paulo.

**Methods**: The study was observational and conducted blindly, without physicians evaluating the relationship between the new monitoring results, symptoms and the outcome of procedures performed. The indicators studied were consistency between the result obtained with non-invasive IC monitoring, symptoms and procedural results. The brain4care sensor was positioned in the frontotemporal region and the data were automatically analyzed by the company's system. In total, 20 patients were monitored before and after surgical procedures. All patients had hydrocephalus or other pathologies that, similarly, increase intracranial pressure. Out of this total 12 patients had hydrocephalus, 5 had arachnoid cysts, 1 had pseudotumor cerebri, 1 had an intradural lipoma and one had craniosynostosis. The results obtained were later compared with the clinical findings.

**Results**: The results showed a level of agreement of 94% with the symptoms and 75% with the results obtained after medical procedures through medical evaluation.

**Conclusions**: We conclude that the device may serve as an adjunct diagnostic method for several pathologies that cause increase of intracranial pressure, including hydrocephalus. It may be valuable for evaluating these patients both in immediate as well as long-term follow-up, as it allows for baseline measures of the nICPw. More studies with larger populations and optimized monitorization routines are required to better understand the added value of the method.

## A164 Epilepsy in Hydrocephalus Patients

### Mohammed Nooruldeen Jabbar^1^, Fatimah Nooruldeen^2^

#### ^1^Neurosurgery Department, Canadian Specialist Hospital, Dubai, United Arab Emirates; ^2^Faculty of Medicine, the University of Manchester, United Kingdom

##### **Correspondence**: Mohammed Nooruldeen Jabbar, Email: mohammed.nooraldeen@yahoo.com

*Fluids and Barriers of the CNS* 2023, **20(Suppl 2)**: A164

**Introduction**: Epilepsy is not a typical symptom of hydrocephalus but can be associated with hydrocephalus and be a complication of its treatment.

Hydrocephalus may be the consequence of a wide variety of congenital and acquired diseases affecting the brain. It commonly requires surgical treatment, which in turn places the patient at risk for a variety of mechanical and infectious complications. The causes of hydrocephalus, treatment, and the complication of treatment are all potential instigators of epilepsy. Furthermore, the onset of epilepsy may precede, coincide with, or follow the onset of hydrocephalus.

**Methods**: 347 patients were enrolled retrospectively who underwent surgical treatment for hydrocephalus in three different hospitals from January 2018 to January 2020 and which were identified by file search of diagnosis and treatment. 43 of them had seizures.

The date of the first shunt surgery and subsequent revision (if present), cause of hydrocephalus, age of the patients and the site of burr hole were recorded.

**Results**: 9 patients had epilepsy as one of the presenting features preoperatively. .

34 patients developed epilepsy following surgery: either single surgery or after revisions of failed shunt surgery

Out of the 347 patients, 87 patients had to have revisions. Out of these 87 patients, 29 developed postoperative epilepsy; while out of the patients with single surgery (260) only 5 had postoperative epilepsy.

Out of the 260 patients, 96 patients had frontal burr hole and of these, epilepsy developed in 4 patients.

Out of the 260 patients, 164 patients had parietal burr hole and of these, only one patient developed epilepsy.

About the age distribution, out of the 347 patients: 262 patients were under 20 years old. Out of the 262 patients, 38 patients developed epilepsy.

**Conclusions**: The risk of development of seizures in hydrocephalus patients is associated with shunt revision, age of the patient and site of burr hole.

## A165 Polyvinylpyrrolidone-coated Ventricular Catheter did not show Cellular Immunoreaction or Adhesion in Choroid Plexus Obstruction

### Bianca Romero^1^, Seunghyun Lee^1^, Scott Self^2^, Jordan Davies^1^, Jenna Ledbetter^1^, Victor Canales^2^, Michael Muhonen^2^, Leandro Castaneyra-Ruiz^1^

#### ^1^CHOC Children’s Research Institute, Children’s Hospital of Orange County, Orange, CA, 92868, USA; ^2^Neurosurgery Department, Children’s Hospital of Orange County, Orange, CA, 92868, USA

##### **Correspondence**: Leandro Castaneyra-Ruiz, Leandro.Castaneyra.Ruiz@choc.org

*Fluids and Barriers of the CNS* 2023, **20(Suppl 2)**: A165

**Introduction**: Ventricular shunting is the primary treatment of hydrocephalus. However, it has an unacceptably high rate of failure, with obstruction of the ventricular catheter as the leading cause of failure in pediatric patients. It is speculated that the catheter itself can induce an immune response cascade that triggers obstruction of the device. The richly vascular choroid plexus may play an active role in this immune response and is one of the primary causes of proximal catheter obstruction.

**Methods**: The case described herein is a patient with a functionally obstructed Polyvinylpyrrolidone-coated (PVP) catheter by in-growth of choroid plexus. It was explanted during shunt revision and analyzed for cellular occlusion as well as catheter pressure and flow in a ventricular simulator.

**Results**: There was no immunological response, and no fibrosis or macrophages detected as are generally seen in catheter obstructions from other materials. However, the choroid plexus was shown to obstruct the PVP proximal catheter without immune response, via a lattice of intertwined choroid plexus tissue drawn through individual catheter holes. Pressure differential across the catheter was elevated up to 15 cm H2O in order to sustain flow rate.

**Conclusions**: The catheter immunological response has been thought to be partially responsible for ventricular catheter malfunction but the PVP catheter did not induce cellular attachment or immune response in the brain. Catheter obstruction was from choroid plexus ingrowth instead. This report provides new insights to help design new catheters with non-immunogenic materials and different hole size and configuration to avoid fibrotic ingrowth into the catheter and avoid catheter obstruction.

## A166 Time course and Clinical Presentation variants for Cerebrospinal fluid diversion device failure in Slit ventricle syndrome. An observational study with 5 years follow up

### Ehab El Refaee, Mohamed Alsawy, Mohammad ElBaroody

#### ^1^Department of Neurosurgery - Cairo University - Egypt

##### **Correspondence**: Mohamed Alsawy, Email: Mohamed.elsawy@kasralainy.edu.eg

*Fluids and Barriers of the CNS* 2023, **20(Suppl 2)**: A166

**Introduction**: Prolonged over-drainage of the CSF can lead to slit ventricle syndrome that would cause multiple shunt obstructions. In this study, we present the variants of the clinical manifestations that can denote shunt obstruction and needing urgent intervention.

**Methods**: we retrospectively collected all clinical data of 6 successive cases that sought medical advice in the outpatient clinic during 2018 that suffered from slit ventricles due to ventriculoperitoneal shunt and started to observe the shunt obstruction episodes and the clinical presentation of such cases.

**Results**: the most common presentation was Projectile vomiting. History of ventriculitis and infection occurred in 2 out of the 6 cases with 4 cases free of any history of CNS infection. Clinical manifestations for failure varied in time course (escalating in one hour in one case , or two weeks. The incidence of shunt obstruction episodes were 3 times during the follow up period of 5 years. In all cases, proximal obstruction was the cause of failure, with only one case with shunt infection due to reservoir exposure. In two episodes in two different cases, the shunt obstruction episode was temporary, and resolved without the need for shunt revision. 5 of the 6 cases are doing well in education with accepted intellectual functions.

**Conclusion**: Slit ventricle syndrome is a critical condition that needs vast experience in congenital hydrocephalus, where the clinical signs are the most reliable to diagnose obstruction and proceed for surgical intervention.

## A167 Shift of blood and CSF volume between the intracranial and intraspinal compartments during positional change

### Andreas Spiegelberg^1^, Andrea Boraschi^1^, Vartan Kurtcuoglu^1,2^

#### ^1^University of Zurich, The Interface Group, Institute of Physiology, Switzerland; ^2^University of Zurich, Zurich Center for Integrative Human Physiology and Neuroscience Center Zurich, Switzerland

##### **Correspondence**: Vartan Kurtcuoglu, vartan.kurtcuoglu@uzh.ch

*Fluids and Barriers of the CNS* 2023, **20(Suppl 2)**: A167

**Introduction**: *Compliance *is a characteristic of the volume-pressure relation of the CSF system. With volume injections into the CSF space and concomitant pressure measurement, *craniospinal compliance* is determined. Separate compartments have their own respective compliance, which add up to the total: Intracranial CSF space compliance as a function of arterial and venous compartments, elasticity of the skull, and flexibility of the dura near the foramina. Intraspinal CSF space compliance as a function of intraspinal arterial and venous compartments, elasticity of the thecal sac, and elasticity of the vertebrae. The individual contributions of intracranial and intraspinal CSF space compliance appear characteristically changed in certain disorders, but there is no agreement on the relative contributions and quantitative changes with disease. One approach for probing changes in compliance distribution is tilt testing, which produces fluid shifts between intracranial and intraspinal compartments. However, dynamic measurement of blood and CSF volume redistribution is technically challenging.

**Methods**: We applied a recently developed method for capacitive measurement of dielectric properties of the head to healthy volunteers during body position changes. The measured signal was found to provide information on the intracranial blood-CSF relationship.

**Results**: Pilot data from three healthy volunteers show a decrease of the signal with head-up tilting at angles up to 45°. At larger tilt angles, no further signal decrease was observed.

**Conclusions**: We attribute the decrease of the signal at small tilt angles to a downward shift of blood, which is enabled by the open venous compartment with outflow towards the right heart. At larger angles, the collapse of the jugular veins prevents further shift of blood. Since no comparable mechanism exists for CSF, a spinal shift of CSF occurs.

We gratefully acknowledge partial funding by the Swiss National Science Foundation through project No. 205321_182683.

## A168 Volume changes in cerebral vascular and CSF compartments in hydrocephalus patients

### Kimi P. Owashi^1^, Cyrille Capel^1,2^, Serge Metanbou^3^, Zofia Czosnyka^5^, Marek Czosnyka^5^, Peter Smielewski^5^, Alexis Joannides^5^, Olivier Balédent^1,4^

#### ^1^CHIMERE UR7516, Jules Verne University, Amiens, France; ^2^Neurosurgery, University Hospital, Amiens, France; ^3^Radiology, University Hospital, Amiens, France; ^4^Image processing, University Hospital, Amiens, France; ^5^Department of Clinical Neurosciences, University of Cambridge, Cambridge, UK

##### **Correspondence**: Kimi Piedad Owashi, kimi.owashi@u-picardie.fr

*Fluids and Barriers of the CNS* 2023, **20(Suppl 2)**: A168

**Introduction**: While active reversible hydrocephalus (ARH) is usually associated with a cerebrospinal fluid (CSF) flow disorder, other factors, such as the arterial and venous compartments and the system’s compliance, may also contribute to its pathophysiology. During the short time of the cardiac cycle, CSF and venous volumes must flush out of the cranium to balance the arterial blood expansion. This study aims to estimate how CSF and venous blood flows respond to rapid systolic cerebral arterial inflow in suspected ARH patients.

**Methods**: Thirty patients (72±8 years) with suspected ARH were classified, by the value of resistance to CSF outflow (Rout), as suitable (ARH+: n = 14, 74±6 years) or unsuitable (ARH-: n = 16, 71±9 years) candidates for shunting. All patients underwent phase-contrast MRI to quantify arterial, venous, and CSF flows. We calculated the arteriovenous volume change during the cardiac cycle from cerebral arterial and venous flows measured at the intracranial (Vvasc,i) and extracranial levels (Vvasc,e). Additionally, we calculated a vascular volume ratio (VRvasc) between these two levels as: VRvasc = Vvasc,i/Vvasc,e. From the CSF acquisitions, CSF stroke volumes were calculated through the pontine cistern (SVpc), the aqueduct (SVaq), and the spinal canal at the C2-C3 level (SVc2c3). Similarly, a CSF volume ratio (VRcsf) between intracranial and spinal compartments was calculated as: VRcsf =  (SVpc+SVaq)/(SVc2c3).

**Results**: Individually, volume changes in vascular (Vvasc,i = 0.76±0.2; Vvasc,e = 1.05±0.4 ml) and CSF (SVpc = 0.36±0.2; SVaq = 0.18±0.1; SVc2c3 = 0.5±0.2 ml) compartments were not significantly different between the groups. Similar for VRvasc (ARH-: 0.94±0.4 vs. ARH+: 0.74±0.1). However, interestingly, VRcsf (ARH-: 0.75±0.4 vs. ARH+: 1.17±0.5) were significantly different between the groups.

**Conclusions**: These findings highlight the system’s complexity since a constant interplay exists between the three main neuro-fluids dependent on arterial inflow. These interactions are variable within the population, which explains the lack of consensus in several proposed parameters where the neuro-fluids are generally studied individually, rather than globally.

## A169 Analysis of transcranial Doppler pulse waveforms in the diagnosis of hydrocephalus

### Arkadiusz Ziółkowski^1^, Magdalena Kasprowicz^1^, Marek Czosnyka^2,3^, Zofia Czosnyka^2^

#### ^1^Department of Biomedical Engineering, Faculty of Fundamental Problems of Technology, Wroclaw University of Science and Technology, Wroclaw, Poland; ^2^Division of Neurosurgery, Department of Clinical Neurosciences, Addenbrooke’s Hospital, University of Cambridge, Cambridge, United Kingdom; ^3^Institute of Electronic Systems, Faculty of Electronics and Information Technology, Warsaw University of Technology, Warsaw, Poland

##### **Correspondence**: Arkadiusz Ziółkowski, arkadiusz.ziolkowski@pwr.edu.pl

*Fluids and Barriers of the CNS* 2023, **20(Suppl 2)**: A169

**Introduction**: Normal pressure hydrocephalus (NPH) is associated with alterations in cerebrospinal fluid circulation but often with reduced cerebral blood flow (CBF). It has been suggested that the shape of cardiac-induced cerebral arterial blood volume (C_a_BV) pulse waveform measured with ultrasound-based method may be altered in NPH patients. In this study we aim to compare the morphology of C_a_BV pulse waveforms estimated based on transcranial Doppler blood flow velocity (TCDFV) in groups of healthy volunteers and NPH patients.

**Methods**: TCDFV signal recorded in NPH patients and healthy individuals at rest and the global CBF model were used to assess the C_a_BV pulse waveforms. A total of 27 quantitative parameters were proposed to describe the shape of the C_a_BV pulse waveform. All parameters were compared between NPH patients and healthy volunteers with the U Mann-Whitney test. A decision tree classifier was applied to choose the most prominent parameter for NPH vs. healthy volunteer classification. The accuracy of the predictor was assessed by the area under the receiver operating characteristic curve (AUC).

**Results**: C_a_BV pulses from 31 patients diagnosed with NPH (age: 59 years (IQR 47–67 years), 14 females) and from 23 healthy volunteers (age: 54 years (IQR: 43–61 years), 18 females) were compared. There was no difference in age between these two groups. 18 of the 27 shape-related parameters were different between healthy individuals and NPH patients (p < 0.05). The most significant differences were found for the ascending slope of the C_a_BV pulse with the AUC of 0.87 (95% confidence interval: 0.77–0.97, p < 0.001), suggesting that in hydrocephalus arterial bed filling is generally faster than in volunteers.

**Conclusions**: The results suggest that the shape of the C_a_BV pulse waveform is altered in NPH patients. Usefulness of C_a_BV pulse shape analysis in NPH diagnosis needs to be confirmed in prospective study.

## A170 Pulsatility and CSF flow resistance in patients with suspected idiopathic hydrocephalus

### Olivier Balédent^1,2^, Alexis Joannides^3^, Kimi Owashi^1^, Serena Cole^3^, Serge Metanbou^2^, Zofia Czosnyka^3^, Pan Liu^2^, Marek Czosnyka^3^, Cyrille Capel^1,2^, Peter Smielewski^3^

#### ^1^Chimère UR 7516, Jules Verne University, Amiens, France; ^2^Jules Verne University hospital, Amiens, France; ^3^Department of Clinical Neurosciences, University of Cambridge, Cambridge, UK

##### **Correspondence**: Olivier Balédent, olivier.baledent@chu-amiens.fr

*Fluids and Barriers of the CNS* 2023, **20(Suppl 2)**: A170

**Study supported by**: Revert Project, Interreg, France (Channel Manche) England, funded by European Regional Development Fund.

**Introduction**: In 2022, the hydrocephalus conference discussed the existence of different potential subtypes of normal pressure hydrocephalus based on disturbances of CSF dynamics. Infusion studies can calculate alterations in CSF outflow resistance (Rout) and PC-MRI can determine alterations in CSF pulsatility. Within the REVERT project, we combined data from infusion tests with the measurement of CSF flows. The objective of this work is to highlight specific groups of alterations in CSF flow and ICP parameters to propose different classifications of CSF dynamics.

**Methods**: 54 patients (73±7 years) with suspected active reversible hydrocephalus (ARH) presenting with the symptoms of Hakim's triad underwent PC-MRI to study CSF cardiac oscillations. Aqueductal CSF and spinal CSF stroke volumes were determined from PC-MRI and combined with Rout calculated from infusion tests. Normal ranges for aqueductal and spinal CSF stroke volumes were between 35 and 115 microliters and 300 and 700 microliters respectively. The cut-off for elevated Rout was taken as 12 mmHg/(ml/min). We defined 9 classes function of CSF flows level and combined this with values for Rout.

**Results**: The majority of patients had an elevated aqueductal SV relative to spinal SV (Class 1, 2, and 4; 31/54; 57%), of which 19 (61%) had an elevated Rout. Out of 11 patients with normal SVs (Class 5), 5 (45%) had an elevated Rout. Only 3 patients had an elevated spinal SV relative to aqueductal SV (Class 6, 8, 9), of which 1 had an elevated Rout.

**Conclusions**: The distribution of elevated Rout amongst the CSF pulsatility classes does not seem to follow any particular pattern from our findings. Rout appears to be independent of CSF cardiac oscillations. This confirms the value of these two methods, which may independently describe pathophysiology of hydrocephalus. Both PC-MRI and infusion tests may contribute to improved diagnosis and treatment of hydrocephalus patients.

## A171 Two HTRA1-related autosomal dominant cerebral small vessel disease combined normal pressure hydrocephalus

### Qiong Yang, Yan Xing

#### ^1^Department of Neurology, Aviation General Hospital of China Medical University & Beijing Institute of Translational Medicine, Chinese Academy of Sciences, Beijing 100012, China

##### **Correspondence**: Yan Xing drxingyan@163.com

*Fluids and Barriers of the CNS* 2023, **20(Suppl 2)**: A171

**Introduction**: Cerebral autosomal recessive arteriopathy (CARASIL) is a hereditary cerebral small vessel disease (CSVD) caused by biallelic mutations in high-temperature requirement A serine peptidase 1 (HTRA1). However, heterozygous HTRA1 mutations were recently identified to be associated with autosomal dominant CSVD.

There are also some overlaps in clinical and imaging features between CSVD and normal pressure hydrocephalus (NPH). However, the relationship between the two diseases is rarely studied.

**Case ReportS**: We herein report two rare cases with genetically confirmed heterozygous missense variant in HTRA1.They also presented with the clinical features and laboratory results of NPH. After CSF tap test or shunt surgery, the two patients both improved by subjective and objective evaluation.

**Conclusion**: We report two Chinese patients with heterozygous HTRA1 mutation distinct from the typical CARASIL. They also co-exist with NPH suggesting an common pathogenesis between CSVD and NPH. Much more needs to be done to understand the relation and distinction between the two diseases.

**Keywords**: CARASIL, HTRA1, epilepsy, CSVD, NPH

## A172 Assessing the relationship between ventricular catheter obstruction and flow using computational fluid dynamics and a multicenter clinical biobank

### Cristopher Roberts, Prashant Hariharan, Carolyn Harris

#### ^1^Wayne State University, Michigan, USA

##### **Correspondence**: Cristopher Roberts bb9561@wayne.edu

*Fluids and Barriers of the CNS* 2023, **20(Suppl 2)**: A172

**Introduction**: Obstruction of flow through the ventricular catheter (VC) is a major contributor to shunt failure. The landmark study by Lin et al. analyzed flow characteristics in VCs using a 2D simulation paired with benchtop experiments on explanted VCs. Expanding on this, Galarza et al. (2014) modeled 3D flow through VC designs and suggested that equal flow across the VC holes would reduce the probability of obstruction. However, a relationship between flow through the VC holes and their obstruction remains to be established. We hypothesized that a higher flow rate through the VC holes would correlate with more significant obstruction. To test this hypothesis, we used computational fluid dynamics to model flow through VCs placed inside the ventricles and compared the flow patterns through the holes with the degree of obstruction in 208 failed VCs from our multicenter clinical biobank of failed VC catheters.

**Methods**: Enlarged lateral ventricles were extracted from MRIs to create computational 3D renders. Physiological boundary conditions were placed at the choroid plexus and catheter outlet and flow parameters, were quantified in the catheter drainage holes. A map of VC obstructions was generated using brightfield imaging. Flow distribution in the drainage holes of VCs were compared to distribution of obstructions in VCs collected from five centers.

**Results**: Preliminary data suggest that the mass flow rate through the VC holes follows a consistent distribution pattern from proximal towards the distal end, regardless of ventricular volume and surgical approach. The flow distribution pattern in our simulations matches the pattern of VC obstruction in 90 of 208 cases and not in 118 cases.

**Conclusions**: Preliminary data suggests that our simulations can represent flow through the VC, and flow distribution correlates with obstructions in a subset of catheters. Flow rate through the VC holes does not solely dictate degree of obstruction.

## A173 A Review of Psychiatric and Cognitive Morbidity in Adult Idiopathic Intracranial Hypertension

### Menaka P. Paranathala^1^, Katie Yoganathan^2^, Lois Gorley^1^, Ian C. Coulter^1^

#### ^1^Department of Neurosurgery, Royal Victoria Infirmary, Newcastle, UK; ^2^Department of Neurology, Royal London Hospital, London, UK

##### **Correspondence**: Ian Coulter, ian.coulter@doctors.org.uk

*Fluids and Barriers of the CNS* 2023, **20(Suppl 2)**: A173

**Introduction**: Psychiatric symptoms, such as depression and anxiety, and cognitive impairment are frequently seen in association with Idiopathic Intracranial Hypertension (IIH) and can negatively impact quality of life (QoL). As patients are predominantly young women of working age, the economic consequences are significant. We undertook a review to assess the psychiatric and cognitive burden of the disease.

**Methods**: We performed a narrative systematic review of relevant literature on adult patients, identified in the OVID, EMBASE, Medline, PsycINFO, CINAHL and PubMed databases in April 2023. Articles were limited to those written in English, and published between January 2000 and March 2023. This study is registered with PROSPERO ID CRD42023399410. Review of titles and abstracts was carried out by two authors and relevant papers were selected for full paper analysis as per PRISMA guidelines.

**Results**: From a total of 454 papers, 24 relevant articles were identified from a range of countries including the UK, USA, Israel, Egypt, Canada, Germany, and Brazil. There is considerable comorbidity of psychiatric conditions including anxiety, depression and eating disorders with IIH, and an increased risk of suicide, when compared to age-, sex- and BMI- matched controls. In addition there are various cognitive impairments seen within IIH. These can significantly impact quality of life of patients with IIH. These can have an impact outwith those of the primary symptoms and can occur alongside and throughout the course of the condition.

**Conclusions**: Aside from optimising symptom control including reducing the severity of headaches and protecting visual function, clinicians should also be mindful of the psychosocial difficulties faced by patients with IIH. Assistance may be required for managing concurrent depression, and anxiety, as well as optimising cognitive functioning. Appropriate neuropsychological assessment may be needed as well as support at the point of diagnosis and throughout the illness to improve QoL.

## A174 Shunting in normal pressure hydrocephalus (NPH): Are we reaching the full potential?

### David Rowland, Zeid Abussuud, Lucia Darie, Lewis Thorne, Laurence Watkins, Ahmed Toma

#### ^1^Department of Neurosurgery, National Hospital for Neurology and Neurosurgery, London, WC1N 3BG, United Kingdom

##### **Correspondence**: David Rowland, david.rowland3@nhs.net

*Fluids and Barriers of the CNS* 2023, **20(Suppl 2)**: A174

**Introduction**: Shunting is the mainstay of treatment for normal pressure hydrocephalus. The maximum potential of improvement in gait after VP shunt is not well understood. We aimed to assess if post extended lumbar drainage walking test should be used as a benchmark to guide shunt valve adjustments in the post operative period.

**Methods**: Single centre, retrospective case series of patients undergoing ventriculoperitoneal (VP) shunt for normal pressure hydrocephalus over a 2-year period from 2019 to 2021. Patients were included who had undergone extended lumbar drainage investigation, with subsequent insertion of a VP shunt and had shown objective improvement on follow up 10m walking tests. Patients without documented 10m walking tests on follow up were excluded.

**Results**: 23 patients were included, 87% were male with an average age of 76 years. All patients had gait disturbance prior to shunting and 43% suffered from Hakim’s triad.

At follow up after VP shunt, 15/23 (65%) of patients achieved greater walking test speeds than the post extended lumbar drainage walking speed. Of these patients 73% of patients achieved this at their first follow up, 20% at second follow up and 7% achieved at their fourth follow up. For those that did not reach the post extended lumbar drain walking speed at their first follow up, reduction in shunt valve settings was utilised in all patients to achieve this.

8/23 (35%) did not achieve post extended lumbar drain walking speed at follow up. This was not achieved despite a reduction in shunt valve settings in 7/8 patients.

**Conclusions**: The majority of patients with NPH who have undergone VP shunt achieve improvement in their gait that is better than after extended lumbar drainage. For those that do not achieve it, shunt valve adjustments are recommended to target greater than the post extended lumbar drainage walking test results.

## A175 The importance of hydrostatic compensation in lumboperitoneal shunts in Hydrocephalus communicans

### August V. Hardenberg, Hans-Joachim Crawack, Jan Mügel, Christoph Miethke

#### ^1^Christoph Miethke GmbH & Co. KG, Potsdam, 14469, Germany

##### **Correspondence**: August v. Hardenberg, august.hardenberg@miethke.com

*Fluids and Barriers of the CNS* 2023, **20(Suppl 2)**: A175

**Introduction**: The use of lumboperitoneal shunts (LPS) has increased in the last decade, especially in Japan and extensive clinical experience is available. The rise in publications since 1999 reflects and highlights the increasing use of LPS. But recent publications still state the siphoning effect in an LPS is negligible, as the lumbar exit and peritoneal entry for LPS are generally at the same level when patients are upright (Ho et al 2023, Miyake 2016). But as Mirone et al (2019) state LPS may cause similar posture-related problems.

**Methods**: The aim of the work is a contribution to the difference between ventriculoperitoneal (VP) and lumboperitoneal (LP) shunts as long-term implants. The flow along the two systems is entirely different when the patient is in vertical position. There is a reversal of flow with different compliance in cranial and spinal derivation. So what is important from clinical perspective?

**Results**: From an engineering point of view hydrostatic compensation is in LP-shunts as important as it is in VP-shunts. Accordingly, Nakajima et al (2018) confirmed good outcomes with the use of gravitational valves (GV) in LPS and stated, “outcomes were improved in the LPS group with the GV”.

**Conclusions**: Although recent studies conclude “LP shunt surgery is equally as effective as the VP shunt surgery” (Wang et al 2019), LPS “might be beneficial” (Kazui et al 2015) or could be “the treatment of choice because of its minimal invasiveness” (Miyajima et al 2016), a broader understanding regarding hydrostatic forces and hydrodynamics in LPS is needed. Moreover, more clinical research is desirable regarding the need of a compensation of the siphoning effect in LPS.

**References**: Ho Y. J., Chiang W. C., Huang H. Y., Lin S. Z. and Tsai S. T.: Effectiveness and safety of ventriculoperitoneal shunt versus lumboperitoneal shunt for communicating hydrocephalus: A systematic review and meta-analysis with trial sequential analysis. CNS Neurosci Ther, 2023, Jan 17

Kazui H, Miyajima M, Mori E, Ishikawa M; SINPHONI-2 Investigators. Lumboperitoneal shunt surgery for idiopathic normal pressure hydrocephalus (SINPHONI-2): an open-label randomised trial. Lancet Neurol. 2015 Jun;14(6):585–94. 10.1016/S1474-4422(15)00046-0. Epub 2015 Apr 28. PMID: 25934242.

Mirone G., Spina D. and Sainte-Rose C.: Shunt hardware, in Pediatric Hydrocephalus, p. edited by G. Cinalli, Publisher:Springer, 2019

Miyajima M, Kazui H, Mori E, Ishikawa M; , on behalf of the SINPHONI-2 Investigators. One-year outcome in patients with idiopathic normal-pressure hydrocephalus: comparison of lumboperitoneal shunt to ventriculoperitoneal shunt. J Neurosurg. 2016 Dec;125(6):1483–1492. 10.3171/2015.10.JNS151894. Epub 2016 Feb 12. PMID: 26871203.

Miyake H.: Shunt Devices for the Treatment of Adult Hydrocephalus: Recent Progress and Characteristics. Neurol Med Chir (Tokyo) 56(5): 274–283, 2016, May 15

Wang Z, Wang K, Qian Z, Zeng L, Gao L. Lumboperitoneal and Ventriculoperitoneal Shunt Surgery for Posthemorrhagic Communicating Hydrocephalus: A Comparison. World Neurosurg. 2019 Jul;127:e638-e643. 10.1016/j.wneu.2019.03.235. Epub 2019 Apr 1. PMID: 30947009.

## A176 Craniometric points for ventricular access, analytical study between history and current trends

### Ehab El Refaee

#### ^1^Professor of Neurosurgery, Cairo University, Giza, Egypt

##### **Correspondence**: Ehab El Refaee: e.elrefaee@googlemail.com

*Fluids and Barriers of the CNS* 2023, **20(Suppl 2)**: A176

**Introduction**: Various Indications are present to gain ventricular access before, during, or after certain neurosurgical procedures. In accordance to the variable pathologies, multiple access points have been designed.

**Methods**: Gathering of data of ventricular access steps during the operative management through the past 2 years in the current institute. Analysis of the role of navigation, years of experience , and wrong implantation of the ventricular catheter has been performed.

**Results**: The ventricle was accessed through 5 different access points in 350 successive cases. The incidence of malposition or tapping failure was 10 %. Years of experience was a good prognostic factor for well positioning while neuronavigation, intra operative ultrasound were secondly beneficial.

**Conclusion**: Correct orientation of all possible access points for the cerebral ventricles is mandatory in neurosurgical practice. Choice of the access point needs correct orientation with the current pathology, postoperative consequences, and the anatomical features.

## A177 Impact Of Global Warming On Brain Temperature

### Kanza Tariq^1^, Sanjay Sissodiya^1^, Ahmed Toma^1^, Lewis Thorne^1^,Laurence Watkins^1^

#### ^1^National Hospital for Neurology and Neurosurgery, Queen Square, London, U.K

##### **Correspondence**: Kanza Tariq, kanza.tariq@nhs.net

*Fluids and Barriers of the CNS* 2023, **20(Suppl 2)**: A177

**Introduction**: The direct impact of global warming on human brain temperature, behaviour and disease burden was studied in the National Hospital for Neurology and Neurosurgery from June 2022 onwards.

**Methods**: In this single-centre prospective-observational study, data was collected from patients with an indwelling M.Scio-ventricular-shunt-sensor-reservoir for various illnesses, at out-patient clinic appointments during summers. Demographic data, past medical history, history of incidents of ill health/relapses requiring medical assistance, history of medication, neurological assessment and baseline vitals were recorded. Only healthy patients with no acute illness were included. The temperature, humidity and carbon-dioxide (CO2) concentration of the clinic-room was recorded using HOBO-MX-CO2-data logger. The brain temperature and ICP were recorded in lying, sitting and standing positions using M.Scio-ventricular-shunt-sensor-reader unit. Patients were asked survey questions focusing on subjective feelings of wellness, heat-tolerance, noticeable behaviour changes and objective incidents of illness requiring medical attention. Same patients were seen again during winters and same measurements were repeated. Results were compared for the different seasons in individual patients. Comparison of two groups of patients (rise in brain temperature vs maintained core-brain temperature) was done by two sample t-test using SPSS (version25.0, IBM).

**Results**: After exclusions, brain temperature was measured in 54 patients during both seasons. Room temperature during summer (average 27.5–29.5°C), was an average 2–3 °C higher than during winter (average 24.5–26.5 °C). Room humidity and CO2-concentration were comparable in both seasons. 34 patients had an increase in brain temperature of 0.7–2 °C during summer as compared to winter (p < 0.0001). The average ICP was higher in all individuals during summer (average 2–6 mmHg). The patient cohort depicting rise in brain temperature with environmental temperature also had  > 3 incidents of ill-health/disease-relapse/hospital admissions during summers and rise in anger/aggression.

**Conclusion**: Global warming is likely to have a direct effect on human brain temperature and the presentation of neurological diseases. Validation of the results is required in larger cohorts.

## A178 Machine Learning-led Exploration of Imaging Biomarkers distinguishing normal pressure hydrocephalus (NPH) vs. controls: Pilot and Test Perturbations Reveal the Robustness of Feature Extraction

### Christine Lock^1^, Nicole C. H. Keong^1,2^, for the Alzheimer's Disease Neuroimaging Initiative*

#### ^1^Department of Neurosurgery, National Neuroscience Institute, 308433, Singapore; ^2^Duke-NUS Medical School, 169857, Singapore

*Data used in preparation of this article were obtained from the Alzheimer's Disease Neuroimaging Initiative (ADNI) database (adni.loni.usc.edu). As such, the investigators within the ADNI contributed to the design and implementation of ADNI and/or provided data but did not participate in analysis or writing of this report. A complete listing of ADNI investigators can be found at: http://adni.loni.usc.edu/wp-content/uploads/how_to_apply/ADNI_Acknowledgement_List.pdf

##### **Correspondence**: Nicole CH Keong, nchkeong@cantab.net

*Fluids and Barriers of the CNS* 2023, **20(Suppl 2)**: A178

**Introduction**: In normal pressure hydrocephalus, NPH, contradictory results in radiological biomarkers reflect concurrent but conflicting changes occurring in brain tissues in response to progressive ventriculomegaly. The interrogation of multi-modality variables may be challenging. We aimed to use machine learning (ML) to - i) characterize structural volumetric and diffusion tensor imaging (DTI) metrics that distinguish patients with NPH vs. healthy controls and ii) test for fairness in such ML derivations.

**Methods**: The study cohort consisted of 12 patients with Complex NPH and 45 healthy volunteers drawn from the ADNI study. We performed volumetric segmentations on all grey and white matter brain regions (Freesurfer 7.1.1) and generated a full profile of DTI metrics (FSL and MRTrix3; FA, MD, AD and RD) for structural regions-of-interest. We applied ML methodology to interrogate the dataset for best model and top feature predictions. Furthermore, we performed perturbations on the NPH dataset to test for robustness of the measures described in the context of small datasets, missing data and range of metrics.

**Results**: Using the test perturbations above, we created four training models. The most robust features that distinguished NPH vs. controls were the left inferior ventricular volume, corpus callosum anterior volume, left pallidum RD and left hippocampus AD. Independent statistical analyses confirmed that these measures significantly differed between cohorts (p < 0.001, < 0.001,  = 0.014 and < 0.001 respectively). In tests of full performance, all models demonstrated high accuracy in classifying cohorts (AUC, AUC-weighted and average precision scores of 1.0, log loss 0.007 - 0.009). The feature most impacted from perturbations was the left thalamus proper AD (p < 0.001), downgraded from top to fifth. The MaxAbsScaler, LightGBM classifier was the best performing ML algorithm.

**Conclusions**: ML approaches may aid in the interpretation of multi-modality/-variable imaging biomarker results in the context of widespread significant but contradictory changes.

## A179 Cerebrospinal fluid oscillations and cerebral blood flows during breathing

### Olivier Balédent^1,2^, Pan Liu^1,2^, Heimiri Monnier^2^, Serge Metanbou^1^, Cyrille Capel^1,2^

#### ^1^University Hospital, Amiens, France; ^2^CHIMERE UR7516, Jules Verne University, Amiens, France

##### **Correspondence**: Olivier Balédent, olivier.baledent@chu-amiens.fr

*Fluids and Barriers of the CNS* 2023, **20(Suppl 2)**: A179

**Introduction**: Real-time phase contrast MRI (RT-PCMRI) can generate velocity map in less than 100 ms. Therefore, it is possible to continuously quantify the oscillations of these neurological fluid flows directly during several respiratory cycles without any cardiac or respiratory synchronization. In this work, we applied RT-PCMRI to quantify the influence of free breathing on neurofluid dynamics.

**Methods**: 10 healthy volunteers were examined using a 3T clinical MRI. Volunteers maintained free breathing during acquisition. The main parameters of RT-PCMRI were as follows TE/TR/flip = 4.9 ms/9.4/10°, SENSE = 2.5, EPI factor = 7, VENC = 60 cm/s, 5 cm/s, and 10 cm/s, acquisition pixel size = 2*2 mm^2^, temporal resolution = 75 ms/frame to 96 ms/frame for 30 s or 45 s.

We applied RT-PCMRI to quantify blood flow in the straight and superior sagittal sinuses, internal and basilar arteries, but also CSF flows in the aqueduct and spinal spaces. A new in-house post processing software segmented all cardiac cycles and labeled them according to their position in the respiratory cycle. We then calculated the influence of thoracic pressure change during breathing on the neurofluid flow dynamics and cardiac period durations.

**Results**: During the elevated pressure of the respiratory period, cardiac period duration (0.84s±0.12s) decreased by (7%). Stroke volumes of cerebral arterial inflow (9800±1200 mm^3^) and output measured venous outflow (6750±1310 mm^3^) decreased by 10% and 8%, respectively. Mean stroke volumes of CSF in the aqueduct (61±33 mm^3^) and in the spinal canal (657±163 mm^3^) decreased by 20% and 18% respectively. CSF net flow presented caudal direction during elevated thoracic pressure and cranial direction during low thoracic pressure but upward and downward movements of CSF flows during cardiac cycle were still present. Net CSF flow of the total acquisition was null.

**Conclusions**: RT-PCMRI can assess the influence of respiration on the dynamics of neurofluid flows in free breathing, neurofluids are slightly influenced by variations of the respiratory thoracic pressure.

## A180 Monitoring patients with CSF disorders – potential use of phase-contrast MRI

### Niklas Lützen^1^, Marco Reisert^2^, Hansjörg Mast^1^, Florian Volz^3^, Amir El Rahal^3^, Christian Fung^3^, Jürgen Beck^3^, Horst Urbach^1^, Katharina Wolf^3^

#### ^1^Department of Neuroradiology, Medical Center, University of Freiburg, 79106, Germany; ^2^Department of Radiology, Medical Physics, Medical Center, University of Freiburg, 79106, Germany; ^3^Department of Neurosurgery, Medical Center, University of Freiburg, D-79106, Germany

##### **Correspondence**: Dr. Katharina Wolf, katharina.wolf@uniklinik-freiburg.de

*Fluids and Barriers of the CNS* 2023, **20(Suppl 2)**: A180

**Introduction**: The spinal cord at the upper cervical spine is moved alongside pulsatile shifts of blood- and CSF volumes. This cardiac-related motion can be depicted by non-invasive phase-contrast MRI. While the detailed physiological mechanisms are yet to be revealed, it has been demonstrated that increased spinal cord motion can be found among patients with spontaneous intracranial hypotension (SIH); thus, in a disorder of spinal CSF leakage. A high reliability and feasibility of this short (1.5 min) sequence has been reported. Also, there is first evidence that reduced spinal cord motion can be found in patients with idiopathic intracranial hypertension (IIH) (preliminary data).

**Methods**: Case reports on patients with SIH and IIH who received axial, ECG-triggered phase-contrast MRI measurements before and after treatment. Analysis was fully automated (www.nora-imaging.org). The velocity range (mm/s) of the time-resolved velocity curve over the cardiac cycle was used as the main parameter. Additionally, time-resolved curves were interpolated to reflect one standardized heartbeat for visual, qualitative comparison.

**Results**: Case A, IIH, female, 37 years: before treatment 3.6 mm/s, after Stenting 5.0 mm/s. Case B, IIH, female, 44 years: before treatment 3.1 mm/s, after Stenting 5.3 mm/s. Case C, SIH (CSF venous fistula), male, 35 years: before treatment 11.9 mm/s, after surgery 6.2 mm/s. Case D, SIH (CSF venous fistula), female, 67 years: before treatment 9.5 mm/s, after embolization 4.6 mm/s and highly reduced area under the curve (Figure 1)

**Conclusions**: The reported cases show that monitoring of patients with CSF volume disorders might be possible by using a fast and non-invasive MRI technique. It might help to discern rebound intracranial hypertension from recurrent leaks, and it might be of use in clinical trials monitoring the effect of the intervention. Informed consent to publish has been obtained by the patients.

**Figure:**
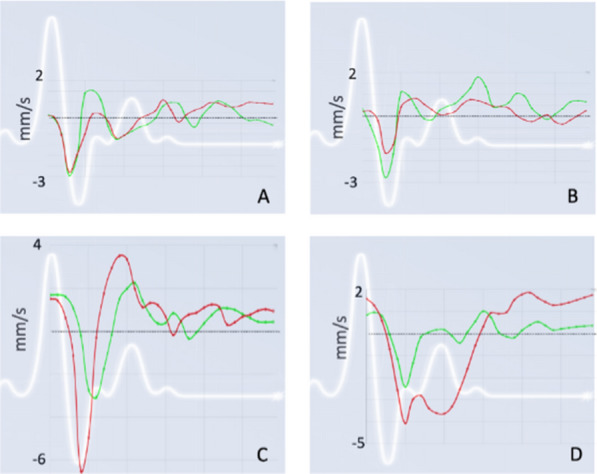
Figure 1: time-resolved phase-contrast measurements of the spinal cord at C2/C3 over one heartbeat in 4 cases. Velocities in mm/2 in cranial direction are positive on the y-axis, velocities in caudal direction are negative. The x-axis resembles one heartbeat. Measurements before treatment are red, after treatment green. Cases A and B are patients with idiopathic intracranial hypertension, treated with stenting. Cases C and D are patients with CSF venous fistula treated by surgery (C), and embolization (D).

## A181 Negative Pressure hydrocephalus and its great therapeutic challenge: Experience in our center

### Cristian Leonardo Ortiz Alonso, Patricia Barrio Fernandez, Jesus Rodrigues Vera, Jenny Leon Rivera, Noelia Miron Jimenez, Juan Rial Basalo, Belen Alvarez Fernandez, Maria Garcia Pallero

#### Hospital Universitario Central de Asturias, Oviedo, Spain

##### **Correspondence**: Cristian Leonardo Ortiz Alonso cristian.or9202@gmail.com

*Fluids and Barriers of the CNS* 2023, **20(Suppl 2)**: A181

**Introduction**: Negative pressure hydrocephalus (NePH) is characterized by ventriculomegaly and symptoms of high intracranial pressure (HICP), despite low or even negative intracranial pressure (ICP) monitoring. Its pathophysiology involves alterations in transmantle pressure, cerebral compliance, and turgor. Diagnostic studies such as elastography have been implemented, and various therapeutic algorithms have been proposed.

**Objectives**: To present our center's experience with NePH, describe our results, and review the literature.

**Materials and Methods**: We reviewed the medical records of four patients treated in our center between 2017 and 2022. To diagnose NePH, each patient had a prior history of an intracranial alteration that could affect the normal properties of the brain, clinical signs of HICP, ventriculomegaly on CT scan, and prolonged ventricular drainage with ICP < 5 cmH2O.

**Results**: The mean age at diagnosis was 42 years, with a 50% female predominance. Most cases were secondary to tumor resection (75%). The mean hospitalization duration was 165.8 days, with nine surgeries and four readmissions. A mean of 3.5 external ventricular drains (EVD) were required, and two patients required third ventriculostomy endoscopy (TVE). As for complications, three patients presented with CNS infections, and two of them had an isolated fourth ventricle. Finally, one patient died, and the remaining three have adjustable low-pressure valves.

**Conclusions**: NePH and its treatment remain a significant challenge, requiring multiple interventions, prolonged hospitalizations, and frequent readmissions, with CNS infection being the most serious complication. Our results are consistent with the available literature and suggest the need to implement protocols or therapeutic algorithms in our center to guide decision-making for these patients.

## A182 Posterior reversible encephalopathy syndrom (PRES) secondary to two cases of intracranial hypotension

### Patricia Barrio, Maria Angeles Garcia Pallero, Cristian Leonardo Ortiz Alonso, Jenny Karina Leon Rivera, Noelia Miron Jimenez, Celia Gonzalez Moldes, Joce Ignacio Gimeno Calabuig, Jesus Antonio Rodrigues Vera

#### ^1^Hospital Universitario Central de Asturias, Oviedo, Spain

##### **Correspondence**: Patricia Barrio patribf@hotmail.com

*Fluids and Barriers of the CNS* 2023, **20(Suppl 2)**: A182

**Introduction**: PRES is a neurological syndrome with specific imaging findings. The clinical manifestations are characterized by headache, visual disturbance, seizures and impaired consciousness.

PRES is associated with many clinical entities, the most common of which are eclampsia, hypertension and immunosuppresive treatment.

PRES secondary to cerebrospinal fluid (CSF) hypovolemia, also called intracranial hypotension, is not a well - recognized cause of this condition.

Two cases of PRES with features of intracranial hypotension are reported.

**Methods**: Medical history and updated literature were rewiewed.

**Results**: CASE 1: A 52-year-old woman was diagnosed with idiopathic intracranial hypertension, after presenting with papilledema and decreased visual acuity.

After a programmable lumboperitoneal shunt was implanted, she complainted of severe headache due to intracranial hypotension despite the valve – opening pressure modification.

CASE 2: A 64-year-old woman with posthemorragic hydrocephalus caused by thalamic bleeding with intraventricular extension, showed excessive drainage after a ventriculoperitoneal shunt was placed.

In both cases, cranial CT revealed widespread low – density areas in cerebral white matter.

We suspected PRES after performing cranial MRI.

Both patients improved the symptoms and radiological findings, after removing the lumboperitoneal shunt and increasing the set pressure of the valve.

**Conclusions**: In this work we aim to draw attention to the possible association of PRES with intracranial hypotension after CSF drainage. Informed consent to publish has been obtained by the patients.

## A183 Bone Regrowth After Burr Hole Craniostomy: Establishing natural history and implications for post-operative trans-burr hole ultrasound

### Albert Antar, Ryan P. Lee, Shahab Aldin Sattari, Michael Meggyesy, Mark Luciano

#### ^1^Department of Neurosurgery, Johns Hopkins University, Baltimore, MD, 21287, USA

##### **Correspondence**: Mark Luciano, markluciano@jhmi.edu

*Fluids and Barriers of the CNS* 2023, **20(Suppl 2)**: A183

**Introduction**: Burr hole craniostomy is performed for ventriculoperitoneal (VP) shunt insertion or endoscopic third ventriculostomy (ETV) in patients with hydrocephalus or other cerebrospinal fluid (CSF) disorders. Interest is growing in trans-burr hole ultrasound for longitudinal post-operative monitoring of ventricular caliber, but formal study is needed to determine rate and degree of bony regrowth that would prohibit this technique. This study evaluates bone regrowth patterns and implications for transcranial ultrasound monitoring.

**Methods**: We retrospectively analyzed CT imaging for 62 adult patients (mean age 61, range 21–85; 81% normal pressure hydrocephalus) with 20mm burr hole and sonolucent PMMA burr hole cover (Longeviti) who underwent VP shunt insertion or ETV, and 39 historical controls (mean age 59, range 19–90; 62% normal pressure hydrocephalus) with standard 14mm perforator burr holes (Acra-Cut) without cover. Bone regrowth was assessed using serial CT scans over up to 1100 days. Linear and logistic regression analyses examined bone regrowth correlations with patient characteristics.

**Results**: The 20mm sonolucent cover cohort experienced initial bone regrowth at a rate of 1-mm per 40 days, with 33% closure after 1 year. The matched-control cohort had 1-mm regrowth per 50 days with 33% closure after 2 years. Regrowth slowed almost completely 6–12 months post-craniostomy in both groups. Bone regrowth was not associated with age, sex, skull thickness, etiology, or implant size.

**Conclusions**: Bone regrowth after burr hole craniostomy is common in adults, occurring rapidly within the first 6–12 months and stabilizing thereafter. This pattern translates to a decreased and frequently unusable ultrasound field-of-view for follow-up imaging in VP shunt or ETV patients. New techniques and materials, such as full-thickness sonolucent implants, inhibited burr hole edges, or larger burr holes, are needed to limit bone regrowth after burr hole craniostomy to enable ultrasound to act as a viable long-term imaging alternative in this population.

## A184 Preclinical quality testing for the early feasibility study of telemetric intracranial pressure monitoring in spaceflight

### Andreas Bunge^1^, Michael A. Williams^2^, Christoph Miethke^1^

#### ^1^Christoph Miethke GmbH & Co. KG, Potsdam, Germany; ^2^University of Washington School of Medicine, Seattle, USA

##### **Correspondence**: Andreas Bunge, andreas.bunge@miethke.com

*Fluids and Barriers of the CNS* 2023, **20(Suppl 2)**: A184

**Introduction**: After 3–6 months in space, approximately 70% of astronauts develop vision problems, known as Spaceflight-Associated-Neuro-ocular-Syndrome (SANS). Its symptoms are similar to those of Idiopathic Intracranial Hypertension (IIH). It is hypothesized that shift of fluid toward the head in zero G increases the intracranial pressure (ICP).

**Methods**: The conventional methods for direct measurement of ICP are too complex for use in space. Instead, the CE-approved telemetric ICP sensor M.scio is planned to be used in an FDA-approved early feasibility study to collect ICP data before, during and after spaceflight. Preclinical quality testing of the devices was performed to ensure that the equipment withstands the challenging environmental conditions encountered during a space mission.

**Results**: The influence of thermal, mechanical and barometric parameters on the performance of the M.scio and the Reader Unit Set was investigated. The characteristics of the devices were compared before and after exposure to these environmental conditions. First results suggest that the Reader Unit Set functions normally after testing.

**Conclusions**: The preclinical quality tests marked an important milestone in the feasibility study, which will be the first to investigate whether and how the ICP changes before, during, and after spaceflight. This study aims to gain a better understanding of how SANS develops and could also provide useful insights for patients with IIH on Earth.

We wish to thank our SpaceX collaborators Marissa Rosenberg and Jaime Mateus.

## A185 Telemetric intracranial pressure recordings in patients with shunts for complex hydrocephalus

### Hassan Bin Ajmal^1^, Louis Taylor^1^, Marian Byrne^1^, Athanasios Zisakis^1^, Georgios Tsermoulas^1,2^

#### ^1^Department of Neurosurgery, Queen Elizabeth Hospital Birmingham, United Kingdom; ^2^Institute of Metabolism and Systems Research, University of Birmingham, United Kingdom

##### **Correspondence**: Georgios Tsermoulas, georgios.tsermoulas@nhs.net

*Fluids and Barriers of the CNS* 2023, **20(Suppl 2)**: A185

**Introduction**: The use of telemetric intracranial pressure (ICP) sensors in people with shunts is expanding, but there are limited data to help interpretation of the telemetric recordings. People with complex hydrocephalus are likely to benefit more with the extra information that telemetric systems provide, regarding the function of the shunt. In this study we determined reference values in patients with a shunt using the M.scio system, in order to assist the interpretation of telemetric ICP data.

**Methods**: This was a single centre cohort study of patients with complex hydrocephalus of various aetiology that underwent cerebrospinal fluid (CSF) shunt insertion or revision with integration of an M.scio telesensor, between November 2019 and September 2022. The first post-operative telemetric measurements in the sitting and supine position were recorded in order to obtain reference values of functioning shunts. The morphology, mean pressure and amplitude (AMP) of the ICP wave were determined.

**Results**: Twelve patients with complex hydrocephalus underwent shunt insertion/ revision with insertion of M.scio telesensor over 35 months. The hydrocephalus aetiology included transition, malabsorption, obstructive and Chiari-related hydrocephalus. All patients had a functioning shunt at the time of the recording. The intracranial pressure (ICP) curve demonstrated pulsatility in all patients in the sitting position and in all but one patient in the supine position. In the sitting position, the mean ICP was − 8.5 mmHg (SD = 2.5) and the AMP was 4.7 mmHg. In the supine position, the mean ICP was 12.5 mmHg (SD = 5.3) and the AMP was 5.1 mmHg.

**Conclusions**: Telemetric ICP recordings provided clinically meaningful information on the function of CSF shunts. Baseline recording should be obtained and can be used for future comparison to exclude shunt malfunction and guide valve adjustment. Larger studies are required to assess the clinical effectiveness of telemetric ICP measurement.

## A186 Changes in the shape of intracranial pressure pulse waveform correlate with cerebrospinal compliance during lumbar infusion tests

### Agnieszka Kazimierska^1^, Magdalena Kasprowicz^1^, Alexandra Vallet^2^, Zofia Czosnyka^3^, Marek Czosnyka^3^, Eric Schmidt^4^

#### ^1^Department of Biomedical Engineering, Faculty of Fundamental Problems of Technology, Wroclaw University of Science and Technology, Poland; ^2^Department of Mathematics, University of Oslo, Norway; ^3^Brain Physics Laboratory, Department of Clinical Neurosciences, Addenbrooke’s Hospital, University of Cambridge, United Kingdom; ^4^Department of Neurosurgery, University Hospital of Toulouse, France

##### **Correspondence**: Agnieszka Kazimierska, agnieszka.kazimierska@pwr.edu.pl

*Fluids and Barriers of the CNS* 2023, **20(Suppl 2)**: A186

**Introduction**: Analysis of intracranial pressure (ICP) pulse waveform has long been suggested as an indirect method of assessing cerebrospinal compliance (C). The ratio of characteristic peaks P1/P2 of the ICP pulse seems to correlate with estimates of C. Frailty, as a shift from normal to pathological brain ageing, appears in turn to be correlated to cerebrospinal elastance (E = 1/C). It was demonstrated that reduction in C results in increasing prominence of peak P2 and progressive rounding of the pulse. Hence baseline ICP pulse characteristics could be meaningful to depict the shift from normal to pathological brain biomechanical aging. But it remained unclear whether lumbar ICP pulse profile actually reflects changes in C.

**Methods**: 41 ICP recordings collected during lumbar infusion tests were selected from a database of 100 normal pressure hydrocephalus patients enrolled in the Proliphyc project (exclusion criteria: insufficient signal quality or peak P1 undetectable over the entire recording period). Peaks P1 and P2 were annotated in individual waveforms using an automated algorithm based on detection of local maxima and pulse curvature. Pulses with indistinguishable peaks were excluded manually. C was estimated using E coefficient calculated from the infusion test. Time courses of mean ICP and C in each patient were compared with peak heights and ratios using Spearman correlation coefficient.

**Results**: The height of P1 and P2 augmented with increasing mean ICP (median group-averaged correlation coefficient over 0.9). Peak ratio P1/P2 was strongly correlated with model-based C (correlation coefficient median [first-third quartile]: 0.69 [0.44–0.83]).

**Conclusions**: Analysis of the relationship between characteristic peaks of lumbar ICP pulse waveform provides an indirect measure of changes in cerebrospinal compliance. Although peak ratios cannot be expressed in absolute units of compliance, this approach could help identify patients with reduced cerebrospinal pressure compensation.

## A187 Chiari malformation type 1 without hydrocephalus and third ventriculostomy

### Ivona Nemeiko, Oliver Runge Skovbo Hansen, Arnar Astradsson, Marianne Juhler, Torben Skovbo Hansen

#### ^1^Department of Neurosurgery, Aarhus University Hospital, Aarhus, Denmark

##### **Correspondence**: Ivona Nemeiko, niemejko@yahoo.com

*Fluids and Barriers of the CNS* 2023, **20(Suppl 2)**: A187

**Introduction**: Third ventriculostomy is described as a treatment option in patients with Chiari malformation type 1 and hydrocephalus (1,2). There is just one case report, describing third ventriculostomy as primary treatment option of increased intracranial hypertension due to Chiari malformation without hydrocephalus (3).

We present our series of 6 cases with symptomatic Chiari malformation type 1 without hydrocephalus treated primarily with endoscopic third ventriculostomy.

**Methods**: We retrospectively reviewed and analyzed the clinical and radiological data of 6 patients with Chiari malformation type 1 who underwent endoscopic treatment at our clinic between January 2020 and December 2022, and reviewed cases reported in the literature.

**Results**: All patients presented with headaches, and none with radiological hydrocephalus. One patient had coexisting small cervical syringomyelia. Preoperative ICP monitoring was abnormal in all cases with increased amplitude and B-waves high but without increased average ICP values consistent with reduced compliance was found in all cases.

5/6 cases experienced clinical improvement of headaches after primary endoscopic ventriculostomy. Only one patient underwent secondary operation with fossa posterior decompression.

**Conclusions**: Third ventriculostomy can be considered as a treatment with acceptable improvement in some patients with Chiari malformation.

## A188 Trends in intracranial pressure (ICP) waveform in the early postoperative period: Does sedation play a relevant role?

### Linda D’Antona^1,2^, Eleanor Moncour^1,2^, Sobiya Bilal^1^, Kanza Tariq^1,2^, Lucia Darie^1^, Lewis Thorne^1^, Laurence Watkins^1,2^, Astri M. V. Luoma^3^, Ahmed Toma^1,2^

#### ^1^Victor Horsley Department of Neurosurgery, National Hospital for Neurology and Neurosurgery, London, WC1N3BG, UK; ^2^Queen Square Institute of Neurology, University College London, London, WC1N3BG, UK; ^3^Department of Neuroanaesthesia and Neurocritical Care, National Hospital for Neurology and Neurosurgery, London, WC1N3BG, UK

##### **Correspondence**: Linda D’Antona, linda.d’antona@nhs.net

*Fluids and Barriers of the CNS* 2023, **20(Suppl 2)**: A188

**Introduction**: Intraparenchymal intracranial pressure monitoring (ICPM) is used to inform decisions in a variety of clinical environments going from the elective to the acute settings. This procedure is often performed under sedation. There is uncertainty on the effects of sedative agents on ICP and their duration, complete clearance of the sedatives from the body is desirable and can lead clinicians to perform prolonged periods of ICPM. This study investigates the trends in postoperative ICP waveform and their possible associations with sedation.

**Methods**: Prospective observational pilot study. Patients admitted for elective intraparenchymal ICPM performed under local anaesthesia with or without sedation were included. Self-calibrating Raumedic ICPM were used. Continuous ICP, pulse amplitude (PA), pCO^2^ and pO^2^ were simultaneously recorded and their trends analysed (60–100 Hz). PCO^2^ and pO^2^ were measured through a noninvasive transcutaneous monitor.

**Results**: Six patients (5F, 44±19 years) were included. Four patients underwent ICPM insertion under sedation (propofol, alfentanil and midazolam), while the remaining 2 only received local anaesthetic. From the moment of insertion of the ICP probe, ICP declined until an ICP steady state was achieved. This decline occurred in all the patients, but was steeper for the patients who received sedation. The ICP steady state was achieved on average 30 min after insertion. The difference between pre- and post-steady state ICP was on average 4±1 mmHg and was statistically significant (t-test p < 0.001). There was no significant difference between pre- and post-steady state pCO^2^ and pO^2^.

**Conclusions**: These preliminary results demonstrate that immediate post-insertion ICP is higher than subsequent recordings, this phenomenon does not seem to be associated with the use of sedative agents and could be the result of local inflammation. Larger studies will be needed to confirm these findings.

## A189 Exchange of Fixed Pressure Gravitational Unit to an Adjustable Gravitational Valve is Safe and Effective Procedure

### Ivans Matvejevs, Michael J. Fritsch

#### ^1^Department of Neurosurgery, Dietrich-Bonhoeffer-Klinikum, Neubrandenburg, Germany

##### **Correspondence**: Ivans Matvejevs, ivansmatvejevs@gmail.com

*Fluids and Barriers of the CNS* 2023, **20(Suppl 2)**: A189

**Introduction**: The broadly accepted, predefined indications for implantation of an adjustable gravitational valve (AGV) are prevention and treatment of over-drainage. The purpose of this study was to evaluate the safety and efficacy of exchanging the fixed pressure unit to AGV to optimize drainage.

**Methods**: We retrospectively analyzed 19 consecutive patients who underwent shunt exchange procedure from 2012 to 2021 in a single centre at the age of 5–79 years (mean 60) with different etiologies of hydrocephalus for 4 indications: 1) optimization of drainage excluding patients with idiopathic intracranial hypertension (IIH), (n = 14). 2) prevention of over-drainage, (n = 2). 3) IIH, (n = 1). 4) treatment for over-drainage,(n = 2). In patients with idiopathic normal pressure hydrocephalus (iNPH), (n = 7), special parameters were used (Evans ratio, NPH-Recovery-Rate and Black-Score). All the patients received a AGV proSA®, Miethke shunt, with different adjustments (16 to 40 cmH_2_O, mean 22,84) depending on individual clinical situation. The mean follow-up was 30 months.

**Results**: In 14 cases (74%) the clinical state improved and in 5 (26%) it was constant, with no deterioration. In patients with iNPH the Evans ratio analyzed with Spearman correlation decreased significantly (r(5) = 0.76, p = .049). There were no statistically significant changes in NPH-Recovery-Rate and Black-Score. There were no device-related shunt failures and no mortality. Over-drainage was observed in 8 (42%) of cases. It could be managed non-invasively in 88% of these cases with improvement of over-drainage in 57% cases. The underdrainage was initially present in 14 cases (74%) and was treated non-invasively in 10 cases (71%).

**Conclusions**: The exchange of fixed pressure unit to an AGV is a safe and efficient procedure leading to improvement of hydrocephalus symptoms with low complication rates.

## A190 Cerebrospinal fluid tau biomarkers show strong potential in early selection for shunt surgery

### Yan Xing^*^, Chunyan Liu^a^, Jijing Wang^b^, Hongliang Li^a^, Qiong Yang^a^, Yanfeng Li^c^

#### ^a^Department of Neurology, Aviation General Hospital, 100012 Beijing, China; ^b^Department of Medical Biochemistry and Biophysics, Karolinska Institutet, 17165 Stockholm, Sweden; ^c^Department of Neurology, Peking Union Medical College Hospital, Beijing, China 100730

##### **Correspondence**: Yan Xing drxingyan@163.com

*Fluids and Barriers of the CNS* 2023, **20(Suppl 2)**: A190

**Introduction**: The idiopathic normal pressure hydrocephalus (iNPH) is a type of neurodegenerative disorder of gait impairment, dementia and incontinence. Here we investigated the correlation between lumbar cerebrospinal fluid (CSF) biomarkers and the CSF tap test (CSF-TT) responsiveness in patients with iNPH.

**Method**: A total of 163 iNPH patients were prospectively enrolled and subjected to CSF-TT. The CSF levels of Aβ_42_, phosphorylated tau 181 (P-tau_181_) and total tau (T-tau) were analyzed.

**Results**: The CSF P-tau_181_and T-tau levels were significantly lower in the CSF-TT responders compared to the non-responders (*P* < 0.05). The lower levels of CSF P-tau_181_were discovered in the CSF-TT responders with the improvement of gait + cognition or cognition only compared to the non-responders (*P* < 0.05).

**Conclusion**: The CSF tau biomarkers show a significantly negative correlation with the responsiveness to CSF-TT, and the CSF biomarkers exhibit strong potential in early selection for shunt surgery.

**Keywords**: cerebrospinal fluid (CSF) biomarkers; CSF tap test (CSF-TT); idiopathic normal pressure hydrocephalus (iNPH); shunt surgery.

## A191 Effect of shunt treatment on Epworth sleepiness scale score in normal pressure hydrocephalus (max 153 characters)

### Simon Lidén^1,2^, Anna Lindam^3^, Dan Farahmand^4^, Anne-Marie Landtblom^1^, Katarina Laurell^4^

#### ^1^Department of Medical Sciences, Neurology, Uppsala University, Sweden; ^2^Department of Neurology, Östersund Hospital, Region Jämtland Härjedalen, Sweden; ^3^Department of Public Heal th and Clinical Medicine, Unit of Research, Education and Development Östersund Hospital, Umeå University, Umeå, Sweden; ^4^Department of Clinical Neuroscience, Institute of Neuroscience and Physiology, Sahlgrenska Academy, University of Gothenburg, Sweden; ^5^Department of Biomedical and Clinical Sciences, Neurology, Linköping University, Sweden

##### **Correspondence**: Simon Lidén, simon.liden@neuro.uu.se

*Fluids and Barriers of the CNS* 2023, **20(Suppl 2)**: A191

**Introduction**: Sleepiness and apathy, symptoms often reported in patients with Normal Pressure Hydrocephalus (NPH), probably contribute to their disability. However, research on outcomes after shunt surgery has mainly focused on other symptoms. This study aimed to be an initial investigation into the effects of shunt treatment on daytime sleepiness and whether there was a relation to changes in ventricular volume (VV).

**Methods**: Pre- and postsurgical daytime sleepiness was investigated using the Epworth Sleepiness Scale (ESS) in a sample of 32 patients with NPH. All participants received Strata® shunts at surgery set at an initial performance lever (PL) of 1.5. Data was gathered before surgery and at follow up one month after surgery. Participants answered the questionnaire with the aid of clinical investigators and/or accompanying family members as needed. The ventricular volume of the lateral as well as third ventricles was measured using quantitative MRI.

**Results**: ESS improved by a median of 1.5 points one month after surgery, p = .026. A subgroup analysis dividing the group by presurgical ESS according to a commonly used cut-off of ESS > 12 revealed a significant effect only in the group with pathological ESS. That group had a median improvement of 12 points (n = 6, p = .035) versus a median change of 0 points in the non-pathological group (n = 26, p = .47). While VV decreased by a median of 10.25 mL post-surgery (interquartile range 5.75 to 15.9, p<.001), there was no significant correlation between change in VV and ESS.

**Conclusions**: Daytime sleepiness seems to be another domain of NPH symptomatology that is responsive to treatment. Although developed for narcolepsy, ESS is a quick test to administer and could be a valuable addition to pre-surgical screening for treatable symptoms.

## A192 Cerebral Blood Flow and Autoregulation in Normal Pressure Hydrocephalus (NPH)

### Afroditi D. Lalou^1,2,3^, Zofia H. Czosnyka^1^, Marek Czosnyka^1^, John D. Pickard^1^

#### ^1^Department of Clinical Neurosciences, Division of Neurosurgery, Brain Physics Laboratory, University of Cambridge & Cambridge University Hospital NHS Foundation Trust, United Kingdom; ^2^Department of Radiation Sciences, Umea University, Sweden; ^3^Department of Neurology, King’s College Hospital NHS Foundation Trust, London, United Kingdom

##### **Correspondence**: Afroditi Lalou, adl43@cam.ac.uk

*Fluids and Barriers of the CNS* 2023, **20(Suppl 2)**: A192

**Introduction**: The available evidence, as summarised by a 2001 review, has indicated the necessity of studying the interaction between CSF circulation and cerebral blood flow (CBF). We aimed to review the research on the topic after 2001, adding cerebral autoregulation. We aimed to summarise the knowledge in CBF and autoregulation in NPH and to report potential directions for investigations and clinical applications.

**Methods**: A systematic review of English and non-English original research papers using PubMed, Cochrane, Scopus, Embase and Web of Knowledge for autoregulation in hydrocephalus published after 2001 was carried out. Search terms included autoregulation/cerebral autoregulation/cerebrovascular reactivity and hydrocephalus / normal pressure hydrocephalus. We included studies of adult humans under investigation for idiopathic or secondary NPH with measurements of CBF and/or autoregulation.

**Results**: 436 articles were assessed for eligibility, out of which 59 met our criteria. Global and regional CBF, cerebrovascular reactivity and autoregulation, as well as CBF before and after shunting and temporary CSF withdrawal were reported.

**Conclusions**: CBF is most probably not reduced globally, showing regional patterns. There was no evidence for CBF measurement as a prognostication tool for shunting. Shunting probably restores CBF patterns to normal. Autoregulation and reactivity show diagnostic as well as prognostic promise in NPH, however this will require further study, in conjunction with cerebral metabolism.

## A193 Exploring the relationship between global white matter and idiopathic normal pressure hydrocephalus triad

### Hongliang Li, Chunyan Liu, Qiong Yang, Yan Xing

#### Department of Neurology, Aviation General Hospital, Beijing, China

##### **Correspondence**: Yan Xing, Email: drxingyan@163.com

*Fluids and Barriers of the CNS* 2023, **20(Suppl 2)**: A193

**Introduction**: Idiopathic normal pressure hydrocephalus (INPH) triad includes cognitive impairment, gait disorders, and urinary incontinence. Few imaging studies have reported the relationship between global white matter (WM) alterations and the hydrocephalus triad. This study explores the relationship between the hydrocephalus triad and global WM in patients with INPH through structural image analysis.

**Methods**: 42 patients with INPH and 24 healthy control (HC) subjects were included in this study. The cerebrospinal fluid tap test (CSF TT) was performed on INPH patients, and gait parameters, cognition, and urinary function were assessed CSF TT by using the 3-meter timed up and go test (3-mTUG), the 10-meter walking test (10-MWT), the Mini-Mental State Examination (MMSE) scale and Montreal Cognitive Assessment (MoCA), and urinary frequency, respectively. All subjects underwent 3D T1-weighted MRI. Statistical parametric mapping 12 was used for preprocessing images, statistical analysis, and voxel-based morphometry for white matter volume analyses. Pearson's correlation analysis and Bonferroni's statistic corrected one-way ANOVA were used to determine the relationship among demographic variables.

**Results**: INPH patients had lower cognitive impairment, more gait disorders, and higher urinary frequency compared to the HCs (p < 0.001). Compared to the HCs, the INPH patients had significantly reduced WM volumes in the bilateral temporal gyrus, precentral gyrus, inferior occipital gyrus, cerebellum (VIII), and right frontal gyrus, left lingual gyrus, right parahippocampal gyrus (p < 0.001). The INPH group had worse MMSE and MoCA scores, and urinary frequency was associated with bilateral temporal gyrus, precentral gyrus, inferior occipital gyrus, cerebellum (VIII), right middle frontal gyrus, left lingual gyrus, right parahippocampal gyrus (p < 0.001). The gait disorders were associated with bilateral temporal gyrus, precentral gyrus, left inferior occipital gyrus, lingual gyrus (p < 0.001).

**Conclusion**: Compared to the HCs, the INPH patient triad was significantly associated with global white matter alterations.

